# BCL-2 Inhibitor ABT-737 Effectively Targets Leukemia-Initiating Cells with Differential Regulation of Relevant Genes Leading to Extended Survival in a NRAS/BCL-2 Mouse Model of High Risk-Myelodysplastic Syndrome

**DOI:** 10.3390/ijms221910658

**Published:** 2021-09-30

**Authors:** Petra Gorombei, Fabien Guidez, Saravanan Ganesan, Mathieu Chiquet, Andrea Pellagatti, Laure Goursaud, Nilgun Tekin, Stephanie Beurlet, Satyananda Patel, Laura Guerenne, Carole Le Pogam, Niclas Setterblad, Pierre de la Grange, Christophe LeBoeuf, Anne Janin, Maria-Elena Noguera, Laure Sarda-Mantel, Pascale Merlet, Jacqueline Boultwood, Marina Konopleva, Michael Andreeff, Robert West, Marika Pla, Lionel Adès, Pierre Fenaux, Patricia Krief, Christine Chomienne, Nader Omidvar, Rose Ann Padua

**Affiliations:** 1 INSERM UMR-S1131, Université de Paris, Institut de la Recherche Saint-Louis, Assistance Publique-Hôpitaux de Paris (AP-HP), Hôpital Saint-Louis Hôpital, 75010 Paris, France; petra.gorombei@gmail.com (P.G.); fabien.guidez@inserm.fr (F.G.); saravanan.mgtian@gmail.com (S.G.); mathieu.c@live.fr (M.C.); laure.goursaud@inserm.fr (L.G.); tekinnilgun@gmail.com (N.T.); stephanie.beurlet@free.fr (S.B.); patelsatyananda@gmail.com (S.P.); lguerenne@gmail.com (L.G.); clp_5@hotmail.com (C.L.P.); marika.pla@inserm.fr (M.P.); patricia.krief@inserm.fr (P.K.); christine.chomienne@inserm.fr (C.C.); 2 Blood Cancer UK Molecular Haematology Unit, Nuffield Division of Clinical Laboratory Sciences, Radcliffe Department of Medicine, University of Oxford, and BRC Haematology Theme, Oxford OX3 9DU, UK; andreapellagatti@yahoo.co.uk (A.P.); jacqueline.boultwood@ndcls.ox.ac.uk (J.B.); 3 Imagerie Département, Université de Paris, Institut de la Recherche Saint-Louis, 75010 Paris, France; niclas.setterblad@univ-paris-diderot.fr; 4 GenoSplice Technology, Paris Biotech Santé, 29 Rue du Faubourg Saint-Jacques, 75014 Paris, France; pierre.delagrange@genosplice.com; 5 INSERM UMR-S942, Université de Paris, Assistance Publique-Hôpitaux de Paris (AP-HP), Hôpital Saint-Louis, 75010 Paris, France; christophe.leboeuf@univ-paris-diderot.fr (C.L.); anne.janin@yahoo.fr (A.J.); 6 Department of Cytology, Assistance Publique-Hôpitaux de Paris (AP-HP), Hôpital Saint-Louis, 75010 Paris, France; maria-elena.noguera@aphp.fr; 7 Radiopharmacie AP-HP, Hôpital Saint-Louis, Service Medicine Nuclear, AP-HP Lariboisiere, 75010 Paris, France; laure.sarda@inserm.fr; 8 Nuclear Medicine, Assistance Publique-Hôpitaux de Paris (AP-HP), Hôpital Saint-Louis, 75010 Paris, France; pascal.merlet@aphp.fr; 9 M. D. Anderson Cancer Center, The University of Texas, Houston, TX 77030, USA; mkonople@mdanderson.org (M.K.); mandreef@mdanderson.org (M.A.); 10 Department of Public Health, Cardiff University School of Medicine, Cardiff CF14 4XN, UK; westrr@cardiff.ac.uk; 11 INSERM UMR-S944, Université de Paris, Institut de la Recherche Saint-Louis, Assistance Publique-Hôpitaux de Paris (AP-HP), Hôpital Saint-Louis, 75010 Paris, France; lionel.ades@aphp.fr (L.A.); pierre.fenaux@aphp.fr (P.F.); 12 Department of Haematology, Cardiff University School of Medicine, Cardiff CF14 4XN, UK; omidvarn@cf.ac.uk

**Keywords:** HR-MDS, BCL-2, ABT-737, gene regulation

## Abstract

During transformation, myelodysplastic syndromes (MDS) are characterized by reducing apoptosis of bone marrow (BM) precursors. Mouse models of high risk (HR)-MDS and acute myelogenous leukemia (AML) post-MDS using mutant NRAS and overexpression of human BCL-2, known to be poor prognostic indicators of the human diseases, were created. We have reported the efficacy of the BCL-2 inhibitor, ABT-737, on the AML post-MDS model; here, we report that this BCL-2 inhibitor also significantly extended survival of the HR-MDS mouse model, with reductions of BM blasts and lineage negative/Sca1+/KIT+ (LSK) cells. Secondary transplants showed increased survival in treated compared to untreated mice. Unlike the AML model, BCL-2 expression and RAS activity decreased following treatment and the RAS:BCL-2 complex remained in the plasma membrane. Exon-specific gene expression profiling (GEP) of HR-MDS mice showed 1952 differentially regulated genes upon treatment, including genes important for the regulation of stem cells, differentiation, proliferation, oxidative phosphorylation, mitochondrial function, and apoptosis; relevant in human disease. Spliceosome genes, found to be abnormal in MDS patients and downregulated in our HR-MDS model, such as Rsrc1 and Wbp4, were upregulated by the treatment, as were genes involved in epigenetic regulation, such as DNMT3A and B, upregulated upon disease progression and downregulated upon treatment.

## 1. Introduction

Myelodysplastic syndromes (MDS) are clonal stem cell disorders characterized by ineffective hematopoiesis leading to blood cytopenias, bone marrow (BM) dysplasia, and an increased risk of developing acute myelogenous leukemia (AML). In high-risk (HR)-MDS (HR-MDS) as defined by the international prognostic scoring system (IPSS), median overall survival is poor. However, prognosis has improved due to the advent of hypomethylating agents and, in a minority of patients, allogeneic stem cell transplantation (alloSCT) [1].

The *RAS* family of genes encodes for GTP-binding proteins located in the plasma membrane, where they play a role in the transduction of signals from membrane receptors. Activating mutations of the *NRAS* oncogene, found in a wide range of human hematological malignancies, are observed in 5 to 30% of HR-MDS patients, often as an early event. There is a predominance of *NRAS* mutations at codon 12 [2], and overall, 78% of patients had one or more oncogene mutations, including *K* and *N RAS* [3]. The mutation renders the active RAS-GTP conformation insensitive to the GTPase activating protein (GAP) and results in constitutive signaling cascades, such as the MAP kinase pathway.

While low-risk (LR)-MDS is characterized by excessive apoptosis of marrow hematopoietic precursors, HR-MDS is associated with a significant apoptosis reduction regulated by the BCL-2 family of apoptotic regulators [4]. In this family, both anti-apoptotic members such as BCL-2, BCL-xL, and MCL-1, and pro-apoptotic members such as BAX and BAK. contain at least one of the four BCL-2 Homology domains (BH1, BH2, BH3, BH4). The ratio between pro and anti-apoptotic BCL-2 family members determines cellular sensitivity to death-inducing signals, including chemotherapeutic agents; around 50% of AML have increased BCL-2 expression, which correlates with poor prognosis [5,6].

A mouse model of human HR-MDS-like disease was created with the mutant *NRASD12* gene under the regulation of the myeloid promoter MRP8 and the human BCL-2 gene under the control of the doxycycline regulatable tetracycline minimal promoter, requiring a transactivator under the control of the MMTV LTR, for expression of the transgene. The mice had blood cytopenias, an excess of BM blasts of around 15%, dysplastic features and blastic infiltration of the liver and spleen [7]. Infiltration by apoptotic myeloid cells was detected by the terminal deoxynucleotidyl transferase (TdT)-mediated dUTP nick end-labeling (TUNEL) assay in mouse liver sections. This finding shows that the pro-apoptotic features of LR-MDS are retained in this model, but increased BM blasts classify it as HR-MDS. [7] The stem cell feature of the disease of this animal model is defined by the ability to repopulate immuno-deficient mice using BM or spleen cells. Furthermore, the Lineage-/Sca-1+/c-Kit+ (LSK) primitive cell compartments of the BM are expanded, correlating with an abnormal myeloid colony growth [7]. This model allowed the first demonstration of an in vivo complex of NRAS and BCL-2 proteins in a pathological context. This complex was subsequently identified in HR-MDS patients correlating with the international prognostic scoring system (IPSS) and percentage of BM blasts [8].

ABT-737 is a BH3 domain mimetic small molecule that can bind the hydrophobic pocket of BCL-2, BCL-x and BCL-w with high affinity and MCL-1 and BFL-1 with low affinity. This binding prevents BCL-2 from blocking the formation of the BAX/BAK complex at the mitochondria. We have previously shown that ABT-737 is efficacious in our mouse model of AML post-MDS [9]; in this present study, we assessed the effect of ABT-737 on survival and leukemia-initiating cells (LICs) in our preclinical mouse model of HR-MDS. In order to obtain molecular insights into the mechanism of action, gene expression profiling (GEP) was undertaken.

## 2. Results

### 2.1. ABT-737 Treatment Prolongs Survival in HR-MDS Transgenic Mice

**Figure 1:**
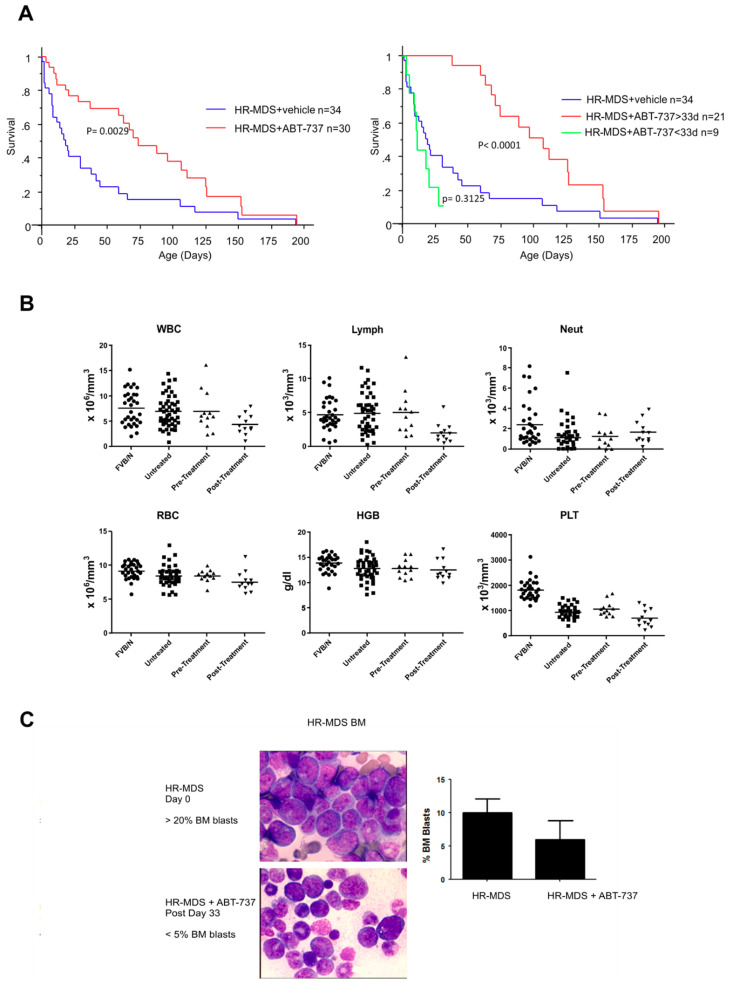

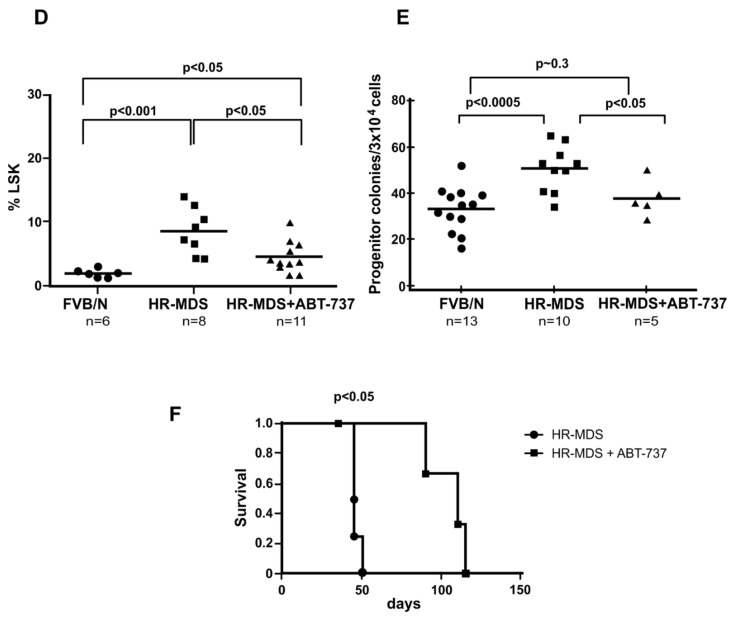
ABT-737 rescues clinical features of HR-MDS mice. (**A**) Kaplan–Meier survival curves showing prolonged survival of ABT-737 treated HR-MDS mice (*n* = 30), and untreated HR-MDS mice (*n* = 34) plotted from date of diagnosis (*p* = 0.0029) (left) and with the treatment group split into completers (*n* = 21) and non-completers (*n* = 9) showing a significant difference between the completers and the untreated control group (*p* < 0.0001) and no significant difference between the non–completers and the controls (right) (*p* = 0.3125); (**B**) White blood cell (WBC) counts; no significant differences were observed between normal FVB/N (*n* = 32), untreated HR-MDS (*n* = 47), pretreated (*n* = 12) and post-treated (*n* = 12) diseased mice; (**C**) Percentage of HR-MDS BM blast pretreatment vs. post-treatment 33 days after the start of the treatment (*n* = 3 mice in untreated HR-MDS group, *n* = 4 mice in treated HR-MDS group). Giemsa stained BM smears showing decreased marrow blasts after treatment (shown in low power [LP 50×] magnification; (**D**) Lin-/Sca-1 + /c-Kit+ (LSK) cell population of normal FVB/N (*n* = 6) vs. untreated HR-MDS (*n* = 8) vs. treated HR-MDS (*n* = 11). Significant increase of untreated HR-MDS mice compared to normal FVB/N (*p* < 0.001), a decrease of treated mice compared to untreated mice (*p* < 0.05) and increase of treated mice compared to normal FVB/N (*p* < 0.05); (**E**) Dot plots showing numbers of day 7 colony-forming unit granulocyte-macrophage per 3 × 10^4^ cells plated dish from BMs of normal FVB/N mice (*n* = 13), untreated HR-MDS mice (*n* = 10), treated HR-MDS mice (*n* = 5). Normal FVB/N vs. untreated HR-MDS *p* < 0.0005, normal FVB/N vs. treated HR-MDS *p* ≈ 0.3, untreated HR-MDS vs. treated HR-MDS *p* < 0.05; (**F**) Differential survival of lethally irradiated syngeneic FVB/N mice transplanted each with 10^7^ spleen cells from untreated HR-MDS or treated HR-MDS mice (*n* = 4 in each group, log-rank χ^2^ = 4.966, *p* < 0.05). One mouse in the treated group was censored due to non-leukemic death at day 35 due to failure of engraftment and two untreated mice died on day 45.

ABT-737 treatment was initiated after confirming the presence of transgenes by genotyping, expression of hBCL-2 by flow cytometry and disease monitoring by measuring blood counts; 64 mice with thrombocytopenia were recruited (platelet counts <1000 × 10^3^/mm^3^) and followed for survival. Thirty were treated with ABT-737 at 75 mg/kg, 3 times per week for 33 days (a total of 15 injections) and 34 were left untreated as controls. Median survival was 15 days for the untreated mice compared to 61 days for the treated mice (*p* = 0.0029) (Figure 1A). When the curves of the treated group were split into mice which completed the treatment (completers, *n* = 21, median 74 days) and those that failed to complete treatment (non-completers, *n* = 9, median 12 days), the completers differed from the controls with increased significance (*p* < 0.0001) whereas the non-completers did not differ from the untreated controls (*p* = 0.3125). No significant improvements in blood parameters were observed in treated mice, with persisting thrombocytopenia (Figure 1B). This may reflect persisting disease or toxicity of ABT-737 on platelets in mice, as was also observed in humans with the orally bioactive, Navitoclax (ABT-263) [10,11,12]. A significant reduction in BM blast counts to a median of 6± 2.8% was obtained, vs. 15± 2.1% in untreated mice (*p* < 0.05) (Figure 1C), with a clearance of tissue infiltration by blast cells in treated mice (Figure 1C).

### 2.2. ABT-737 Treatment Targets Leukemia Initiating Cells (LICs) and Primitive Progenitors

We have previously reported that our HR-MDS mice show an expansion of the myeloid immature cell compartment described as LSK+ cells and an increase of myeloid progenitors with features resembling human RAEB [7]. After 33 days of ABT-737 treatment, the proportion of BM LSK cell population decreased to nearly normal levels (1.8 ± 0.6% normal; 8.5 ± 3.6% untreated vs. 4.5 ± 2.4 in treated and remained significantly different (*p* < 0.05 between the treated and untreated mice and between treated and normal mice) (Figure 1D). The colony growth was restored to near normal range (33 ± 10 in normal FVB/N mice, 51 ± 10 in untreated vs. 38± 8 in treated mice colony-forming unit-granulocyte macrophage—CFU-GM progenitors per 3 × 10^4^ BM cells) (Figure 1E, *p* < 0.05).

Lethally irradiated syngeneic mice with secondary transplants of untreated HR-MDS spleen cells all died within 50 days, whilst those transplanted with cells from 33-day ABT-737 treated animals, 3 of 4 remained alive for 90 to 115 days (Figure 1F *p* < 0.05). One mouse died at day 35 with no evidence of leukemia due to failure of engraftment with no expression of human BCL-2 (hBCL-2) and was censored. All the engrafted mice demonstrated hBCL-2 expression measured by flow cytometry (data not shown).

### 2.3. ABT-737 Treatment Induced Reduced Apoptosis in the BM, Increased Apoptosis and Inhibition of Cell Proliferation in the Liver and Spleen of HR-MDS Transgenic Mice

**Figure 2:**
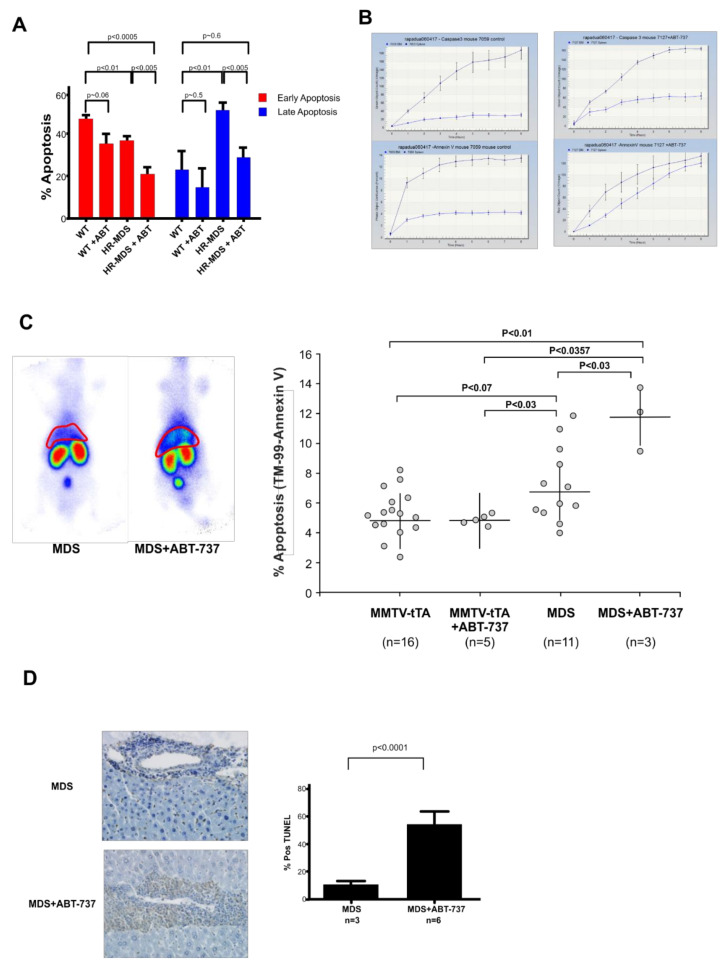
ABT-737 treatment of HR-MDS cells reduced apoptosis of BM cells and induces apoptosis in the liver and spleen of HR-MDS mice. (**A**) Histogram of mean ± SD showing a decrease of early and late apoptotic cells (*n* = 3); (**B**) Representative incucyte profiles of normal healthy untreated normal and HR-MDS treated mice showing reduced BM and increased spleen apoptosis; (**C**) Paired untreated and treated radioisotope heat maps of ^99m^Tc labeled annexin-V, which targets apoptotic cells and is metabolized by kidneys and bladder, shows greater intensity around the localization of the liver in the treated HR-MDS mouse. Dot plot showing increase of ^99m^Tc labeled annexin-V uptake in liver of the treated HR-MDS mice (*n* = 3) compared to untreated HR-MDS mice (*n* = 11), *p* < 0.03, compared to treated single transgenic mice (*n* = 5), *p* ≈ 0.03, and compared to untreated single transgenic mice (*n* = 16), *p* < 0.01 after 23 days of treatment. Untreated HR-MDS mice show an increase of ^99m^Tc labeled annexin-V uptake compared to treated single transgenic mice, *p* < 0.03; (**D**) Representative DNA fragmentation as demonstrated by TUNEL-positive myeloid cells is significantly greater in the liver of treated HR-MDS mice (*n* = 6) compared to untreated HR-MDS mice (*n* = 3) after 33 days of treatment, *p* < 0.0001. The nuclei of the hepatocytes are staining in blue and the brown staining apoptotic cells are in the sinusoid spaces.

Treatment with ABT-737 reduced early and late apoptosis in the bone marrow (Figure 2A). Paired BM and spleen samples assayed using the incucyte showed reduced BM and increased spleen cell death (Figure 2B), similar to the pattern seen in normal cells (Figure 2B). Liver and spleen were studied by in vivo imaging using SPECT; Post day 33 analyses of paired pre- and post-treated wild type and HR-MDS mice were imaged (Figure 2C). In contrast to the BM, ABT-737 induced a significant increase of the ^99m^Tc AnnexinV uptake in the liver and spleen area (Figure 2C). The single transgenic MMTV mouse showed no increase in uptake of ^99m^Tc AnnexinV post-treatment compared to pre-treatment profiles (3.9 ± 1.0 vs. 4.6 ± 0.2), while the HR-MDS mice had a nearly twofold increase in the uptake of radioactivity upon treatment (6.7 ± 2.1 vs. 11.8 ± 2.1 *p* < 0.02) showing increased apoptosis. These results were corroborated by increased apoptosis in the treated HR-MDS mice assayed by TUNEL staining of paraffin-embedded liver sections (12% untreated and 58% treated) (Figure 2D).

### 2.4. ABT-737 Induced Inhibition of BCL-2 Reduces RAS Activity in Sca1+ Cells

**Figure 3:**
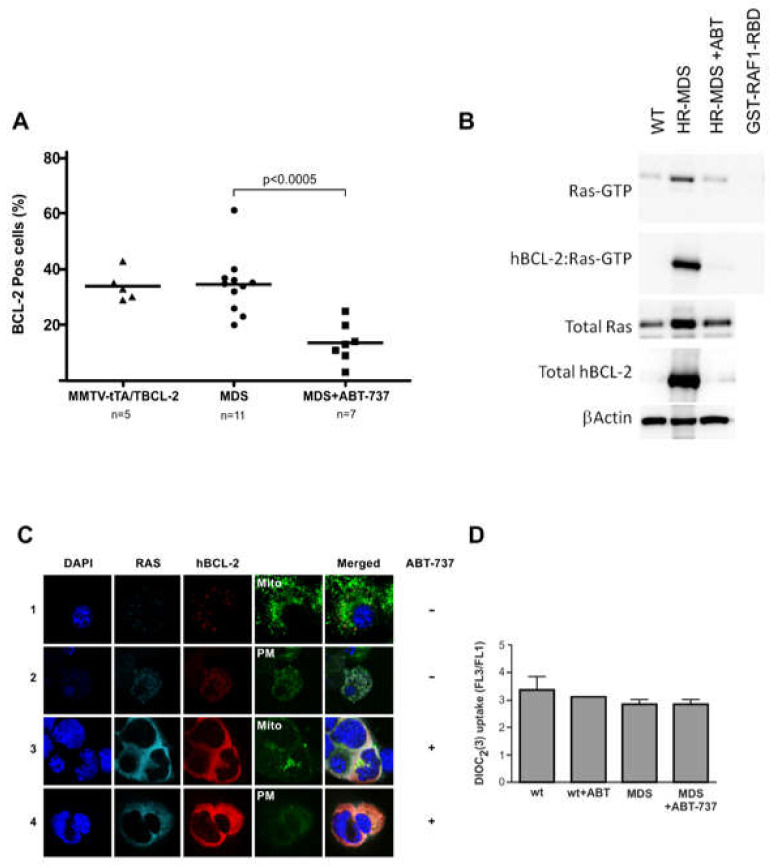
Inhibition of BCL-2 reduces BCL-2 expression and RAS activity in Sca-1+ cells. (**A**) Percentage of peripheral blood hBCL-2 positive (pos) cells in MMTV-tTA/TBCL-2 mice (*n* = 5) compared to untreated HR-MDS mice (*n* = 11), compared to treated HR-MDS mice (*n* = 7), showing a significant decrease of BCL-2 positive cells after treatment *p* < 0.0005; (**B**) Representative Western blot analysis of protein lysates from Sca-1+ enriched spleen cells of untreated, 75 mg/kg ABT-737 treated mice after day 33 of treatment and assessed for total RAS and hBCL-2 expression showing a decrease in RAS-GTP. Blots were reprobed with anti β-Actin antibody to assess protein loading. *n* = 3 mice were assayed; (**C**) Confocal microscopy of stained BM cells showing subcellular localization of the NRAS:BCL-2 complex of HR-MDS mice in the plasma membrane in untreated HR-MDS mice (rows 1 & 2) and in treated HR-MDS mice (rows 3 & 4). Mitochondria stained with Tom20 antibody, plasma membrane stained with anti-Ezrin antibody. *n* = 2 mice were assayed. (**D**) Mitochondrial membrane potential measurements of splenocytes by DiOC2(3) show no difference of dye uptake in ABT-737–treated normal (FVB/N) and HR-MDS mice relative to untreated samples (minimum in each group, *n* = 3 mice).

ABT-737 treatment decreased the percentage of cells expressing BCL-2 in the peripheral blood (Figure 3A), as assayed by flow cytometry. The RAF1-RBD pull-down assay of the ABT-737 treated mice confirmed the reduction of BCL-2 from the complex, which led to a reduction of the RAS activity seen by the RAF1-RBD pull-down assay (Figure 3B). The localization of the complex remained in the plasma membrane (Figure 3C). Furthermore, the MMP was not changed (Figure 3D).

### 2.5. ABT-737 Treatment Induces Regulation of Pathways Implicated in Cell Survival, Proliferation and Stem Cell Regulation in HR-MDS Mice

**Figure 4:**
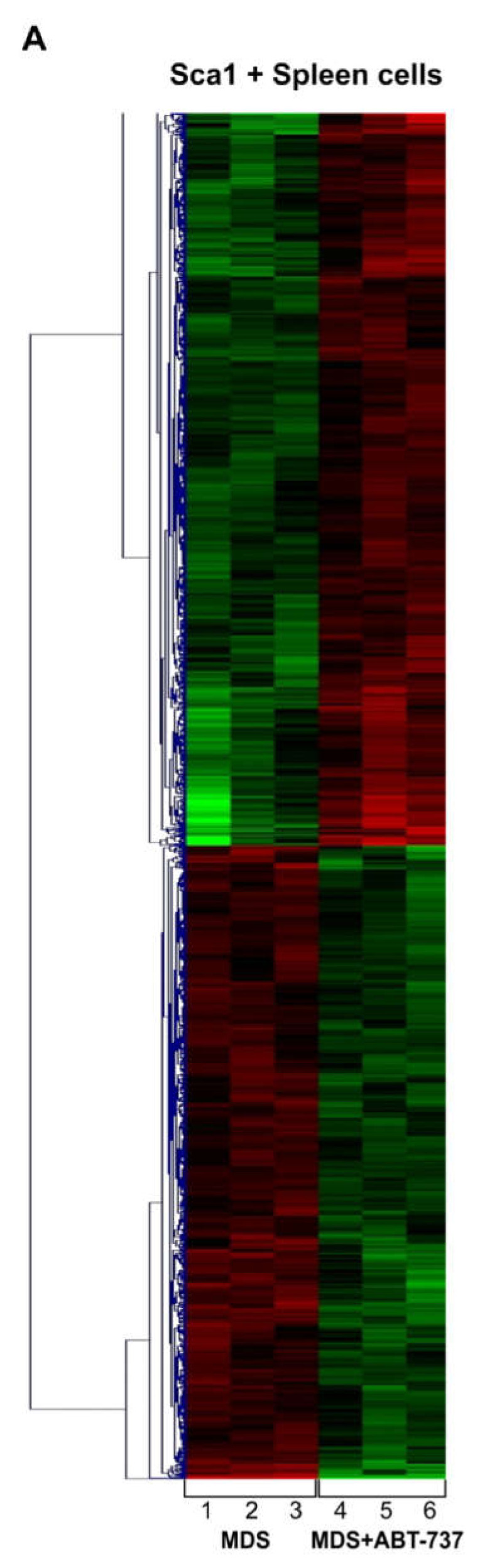

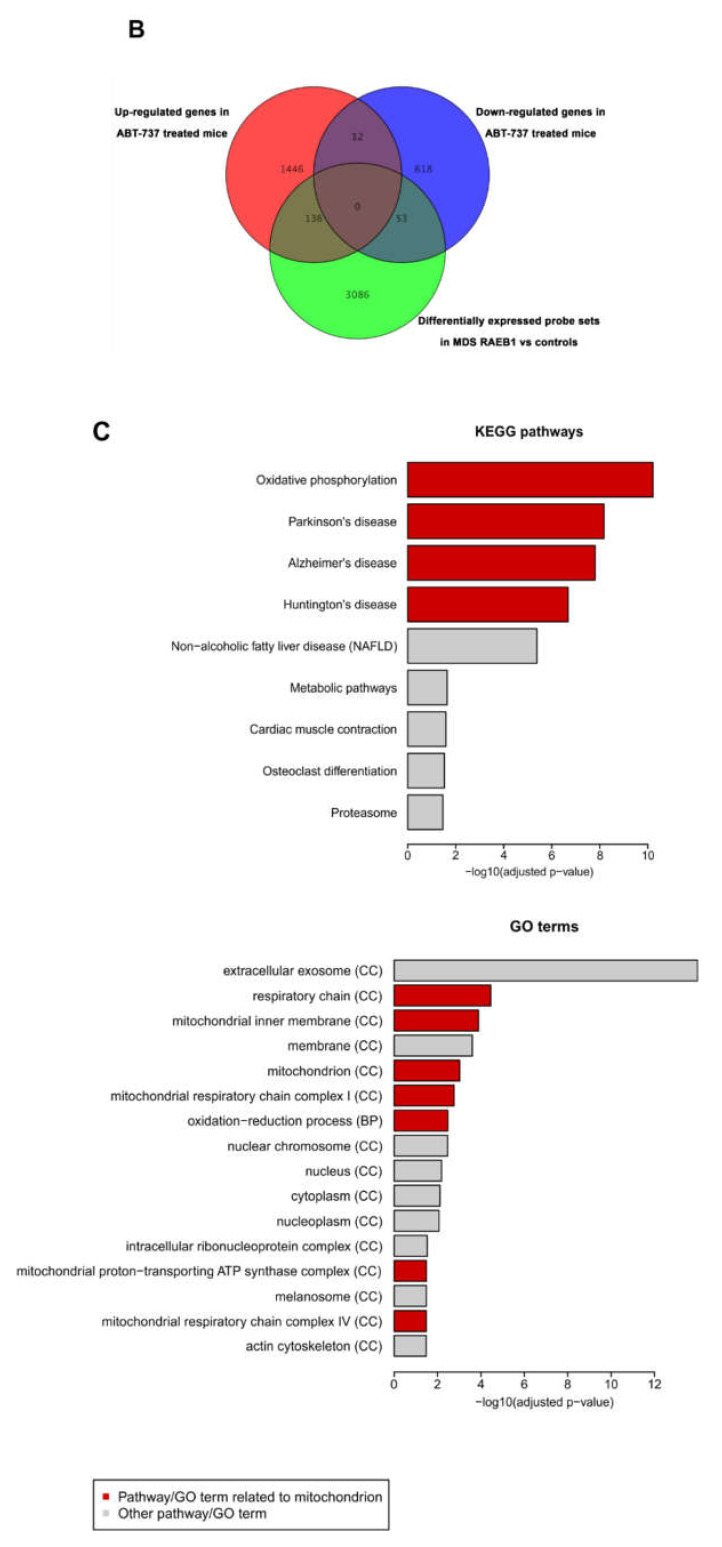

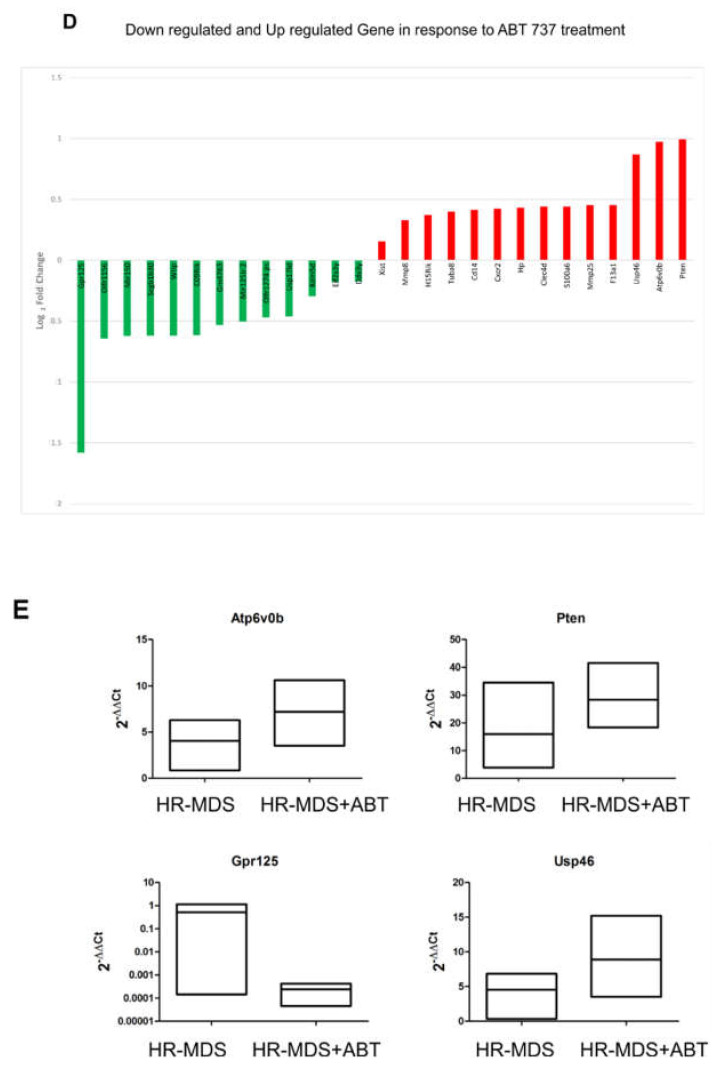
Gene expression profiling reveals differential gene expression signatures after ABT-737 treatment. (**A**) Exon-specific microarray heat map of Sca-1+ enriched spleen cells from untreated HR-MDS mice (*n* = 3) compared to treated HR-MDS mice (*n* = 3) relative to normal FVB/N. Each row represents an independent mouse in each group; (**B**) Venn diagram showing common and unique differentially expressed genes between ABT-737 treated/control HR-MDS model and human MDS RAEB1 v control samples [13]; (**C**) Diagram of significant KEGG Pathways with *p*-value; (**D**) Significantly dysregulated genes identified between ABT-737 treated and control HR-MDS cells (Fold-Change > 1.5; *p* < 0.05); (**E**) RQ-PCR *ATP6v0b* (upregulated), *Pten* (upregulated), *Gpr125* (downregulated) and *Usp46* (upregulated), representative genes regulated in GEPs.

Differential exon-specific gene expression profiling (GEP) in Sca-1+ splenocytes after treatment (*n* = 3) compared to untreated HR-MDS mice (*n* = 3) revealed 1952 genes differentially expressed upon treatment, distributed into two distinct cluster groups (Figure 4A). The full gene list represents 1048 upregulated and 904 downregulated genes (Supplementary Table S2).

In order to convey the importance to the human disease, we next compared our list of differentially expressed genes between the ABT-737 treated and untreated HR-MDS model with the significantly differentially expressed probe sets (*p* < 0.05, Benjamini-Hochberg multiple testing correction) between RAEB1 and healthy control bone marrow CD34+ cells from an existing MDS gene expression study [13]. Clear selective over-lap exists between the probe datasets, where 53 sets are uniquely downregulated and 136 sets are uniquely upregulated (Figure 4B).

The most biologically relevant genes are shown in Supplementary Table S3A,B. KEGG pathway analysis of the gene expression array showed oxidative phosphorylation (Figure 4C, Supplementary Table S4A,B), cell cycle (*Cdks*, *Cdcs*, *Ccns*, *Ccnds*) and certain pro-apoptosis genes (*Apaf1, Bak1, Casp1*) upregulated by ABT-737 treatment (Table 1). Other apoptosis-related genes were found to be downregulated, such as *Lck* and *Fas/FasL*. Tumor suppressor genes such as *Map3k14* were upregulated upon treatment. Stem cell and differentiation gene expression were also regulated upon treatment. Among others, a very important regulator of stem cell development, *Aldh 2* and *3* (aldehyde dehydrogenase) were found to be upregulated, and *Lck* downregulated. Splice related genes were also found to be regulated (Table 1) with 10 genes upregulated, including argenine/serine-rich coil-coil-1 (Rsrc1, 1.55-fold, *p* = 0.0359), WW domain-binding protein 4 (Wbp4, 1.85-fold, *p* = 0.0167) as well as the MDS relevant Zrsr2 spliceosome gene (1.71-fold, *p* = 0.0437). Seven genes were downregulated, including a putative RNA binding protein, Rbm20 and Tut1, which functions as both a terminal uridyl transferase and a nuclear poly(A) polymerase, which may control gene expression and cell proliferation (1.73-fold, *p* = 0.0136 and *p* = 0.2 respectively).

In our HR-MDS model, ABT-737 modulated the expression of 238 genes associated with epigenetic regulation (Supplementary Table S5A). These include important epigenetic regulators involved in gene silencing by DNA methylation (Tet methycytosine dioxygenase (*Tet*), DNA methyl transferases (*Dnmt*) and DNA methyl binding protein (*Mbd*) and histone modifications (Histone deacetylases (*Hdac*) (Supplementary Table S5B).

Genes regulated with ABT-737, but consistent within control samples were selected when ordered with the following parameters: Fold-Change > 1.5; *p* < 0.05. The probe sets representing important functional pathways, such as cell cycle, hematopoiesis and cell signaling were shown to be down- or up-regulated in response to ABT-737 (Figure 4D). Four representative genes shown to be regulated by GEP have been confirmed by RQ-PCR (Figure 4E). These genes code for *ATP6v0b* (upregulated, ATPase subunit), *Pten* (upregulated, phosphatase and tensin homolog), *Gpr125* (downregulated, G-protein) and *Usp46* (upregulated, ubiquitin apoptosis regulator).

## 3. Discussion

ABT-737 has already shown its efficacy in vitro on the apoptosis of carcinoma and AML cell lines [14], ex vivo on patient blasts, [15] in vivo on a murine model of lymphoma [16], on a xenograft model of small cell lung cancer [17] and our own mouse model of MRP8[NRASD12/BCL-2] AML post-MDS [9].

In the present study, we found that ABT-737 significantly improved survival in our HR-MDS mouse model. The survival advantage was corroborated with a significant reduction of the BM blasts and clearance of the infiltrating myeloid cells in the liver and spleen.

As HR-MDS is a clonal disorder of hematopoietic stem cells, effective treatment may have to target the leukemic stem cell population. After treatment, the significant reduction of progenitor numbers and LSK cells to near normal FVB/N levels, suggests the treatment of the disease. The extended lifespan of the secondary transplanted mice injected with cells from treated mice suggests the reduction of LICs by ABT-737. Moreover, we show with SPECT that there is differential induction of apoptosis of the diseased cells by ABT-737 whilst normal cells are spared. Indeed the restoration of nearly normal LSK, progenitor growth, delay of death in the secondary transplant experiments, reduction of BCL-2 expression and RAS activity and rescue of blood parameters suggest a reduction of disease. Overall, these findings indicate that ABT-737 targets leukemia-initiating cells.

BM apoptosis measurements by flow cytometry show a reduction in early and late apoptosis after treatment. Paired BM and spleen samples assayed using the incucyte showed reduced BM and increased spleen cell death. Invaded organs had apoptotic cells detectable in the liver and spleen areas measured by SPECT and TUNEL. These findings suggest that the BM cells have cleared the diseased cells after treatment, whereas the organs still have diseased apoptotic cells.

Studies showed that ABT-737 induces apoptosis through *Apaf1* in mouse embryos [18], which is consistent with our finding of upregulation of *Apaf1* in our arrays from Sca1+ spleen cells, stressing the capacity of this drug to promote apoptosis specifically in the Sca1+ cell compartment. Downregulation of BCL-2 induces apoptosis and is consistent with the reduced expression of BCL-2 measured by flow cytometry. *FasL* expression was found upregulated in HR-MDS [19] and is downregulated after treatment. Interestingly, *Usp46,* a gene in the ubiquitin pathway and confirmed by RQ-PCR is upregulated after treatment. The kinetics of apoptosis of paired samples may reflect the differences in gene expression of apoptotic genes, but in general, ABT-737 is associated with reduced BM apoptosis and increased organ apoptosis, the same as the pattern seen in normal cells, suggesting the restoration of a nearly normal state at the end of treatment.

*Csf2rb* encodes a common beta chain of the receptors of interleukin 3, interleukin 5, and granulocyte-macrophage colony-stimulating factor, and its partners’ expression has been described in specifically marking leukemia stem cells [20]. As deregulation of spliceosome, ribosomal and epigenetic genes are ways in which global gene expression changes can be initiated, it is pertinent that ABT-737 targets these genes to reverse the disease. The role of the calcineurin homologous protein, *Chp1*, as an inhibitor of ribosomal RNA transcription by repressing the nucleolar *Ubf1* transcriptional activity has been described, but its role in normal and leukemic hematopoiesis has yet to be elucidated. *Aldh* is found to be upregulated in our HR-MDS mouse model; it is an important hallmark of leukemic stem cells in the bone marrow of patients with AML [21]. Abnormalities of the spliceosome are frequently found in MDS patients, and these genes were found to be regulated after treatment with ABT-737.

Among the epigenetic genes downregulated by ABT-737 treatment, we mostly found factors associated with gene repression, including genes either involved in DNA methylation (*Dnmt3A* and *B*, *Tet 1* and *3*), histone methylation (*Dotl1, Ehmt2, Kmt2*) and histone deacetylation (*Hdac 1, 2,4,5* and *10* and *Sirt1*), features usually associated with HR-MDS pathogenesis. On the other hand, numerous co-activators implicated in gene activation by histone acetylation (*Ncoa1* and *2, Kat 2* and *7, Ep300*) are upregulated. The differential expression of these epigenetic regulators upon the ABT-737 treatment could explain the broad number of upregulated genes found in this HR-MDS model.

In this HR-MDS model, BCL-2 is also complexed with the constitutively activated NRAS in the plasma membrane and already has increased apoptosis compared to normal cells. After ABT-737 treatment, we found a reduction of this complex, but it remained detectable in the plasma membrane, where RAS normally resides with increased apoptosis in the liver and spleen. ABT-737 was not able to disrupt the complex physically due to its different binding site on BCL-2 (BH3), whereas the RAS binding site on BCL-2 is the BH4 domain [22], but nevertheless, treatment diminished the complex. The synergy of ABT-737 with farnesyltransferase inhibitors (such as tipifarnib) may further disrupt the complex with additionally improved survival. There is also a reduction in BCL-2 expression (Figure 3A,B). Although RAS is not selectively targeted by ABT-737, several downstream targets were found to be downregulated as measured by GEPs, such as *Erk* and *Akt*, probably due to their association with BCL-2. In contrast with our AML model where the RAS:BCL-2 complex localizes in the mitochondria where the cells have reduced apoptosis, after ABT-737 treatment, the complex is found in the plasma membrane with an increase in MMP [9], in the HR-MDS model, there was no change in MMP between diseased cells and cells from wild type healthy animals and between pre and post-treatment with the RAS:BCL-2 complex remaining in the plasma membrane. However, pathway analyses showed that the mitochondrial genes were regulated.

Shared and unique transcripts with human RAEB supports that both the model reflects the human disease and that ABT-737 treatment regulates these relevant surrogate markers of disease. Several cell cycle-related genes were found to be upregulated by ABT-737. The upregulation of cell cycle-related genes could permit the restoration and repopulation of the organs with normal cells; this contrasts with the downregulation of cell cycle genes in our AML model [9]. The observed increased expression of cell cycle-related genes following treatment could provide a mechanism by which cells progress G1 to S phase, leading to expansion of normal myeloid hematopoiesis, thus extending survival of the treated mice [23].

*Pik3cg*, a phosphoinositide 3-kinase, was found to share common sets of upstream and downstream receptors and targets with the RAS signaling pathway, including Akt and Erk [24]. The co-activation of these pathways has frequently been found in several tumor types, including AML with NRAS mutations [25]. *Pik3c* activation was also demonstrated to be necessary for RAS-induced transformation. The regulation of this gene may be a very important factor in the effectiveness of ABT-737 in our mouse model and, therefore, in human HR-MDS. A lymphocyte-specific protein tyrosine kinase (*Lck*), already found to be important in the evolution of chronic lymphocytic leukemia (CLL) [26], regulating Erk and Akt phosphorylation, is targeted by ABT-737 in our mouse model. Erk and Akt are two components of the RAS-mediated signaling pathway involved in the regulation of differentiation and proliferation. ABT-737 also regulated the expression of genes involved in the Toll-like receptor signaling pathway. The upregulation of TLR7/8, an activator of MyD88, leads to an upregulation of the NFkB mediated immune response. *Tlr4* and CD14 are also regulated, activating the Erk signaling pathway with pro-inflammatory effects. Thus, ABT-737 seems to upregulate the immune system.

A related orally-bioavailable derivative, ABT-263 (Navitoclax) has been tested in phase 1 clinical trial in patients with both solid tumors or hematological malignancies and despite mild myelotoxicity, promising efficacy has been reported [27,28,29]. Another derivative, ABT-199 (Venetoclax), has been promising in clinical trials in CLL [30,31], lymphoma [32] and AML [33]. Compared to ABT-263, AML cases treated with a combination of ABT-199 and hypomethylating agents has resulted in complete remission with reduced side effects (such as myelotoxicity), thus leading to multi-centre phase 3 trials. 

## 4. Materials and Methods

### 4.1. Transgenic Mice

The *MMTVtTA* [34]*/TBCL-2* [7]*/MRP8NRASD12* [35] mice were generated from the cross of *MMTVtTA/TBCL-2* with *MRP8NRASD12/MMTVtTA* hemizygote mice, as previously described [7]. As this is a Tet-off system, BCL-2 is constitutively switched on in the absence of doxycycline. Mice were classified according to the Bethesda method [35] as HR-MDS-like. Mice were maintained in the barrier facilities of our institution, under the appropriate animal project licenses. All procedures complied with national regulations on the use of animals for experimentation. Mice were sacrificed when they were moribund or, upon veterinary advice, “blinded” to treatment status. Mice were bred and genotyped using standard husbandry and PCR techniques as described. HR-MDS mice develop disease after 3 to 6 months as measured by a decrease in platelet count (<1000 × 10^3^/mm^3^), with marrow blasts of around 15%.

### 4.2. ABT-737

ABT-737 (kindly provided by MDA, Anderson, TX, USA and Abbott Laboratories, Abbott Park, Illinois, IL, USA) was used in a final formulation of 30% propylene glycol, 5% Tween 80, 65% double distilled water, pH 4–5. An amount of 75 mg/kg was administered three times weekly for 15 injections for 33 days as described previously [9].

### 4.3. Tissue and Cell Preparation, Flow Cytometry, Incucyte

The BM and spleen were prepared as previously described [7]. Blood was obtained from anesthetized animals (with isoflurane) by venipuncture of retro-orbital venus plexus into EDTA tubes. Differential blood counts were obtained using an automated hematology analyzer (Cell Dyn, Abbott Diagnostics, Abott Park, IL, USA or Medonica, Kitvia, Labarthe-Inard, France). White peripheral blood (PB) cells were analyzed by flow cytometry as previously described [7] (FACSCalibur, Becton Dickinson, San Jose, CA, USA); PB BCL-2 expression was performed by flow cytometry using the human-specific BCL-2 (hBCL-2) antibody (BD Pharmingen, San Diego, CA, USA). Bone marrow (BM) was obtained by flushing long bones. PB and BM smears were prepared according to standard hematological techniques, stained and examined by a cytologist of Hôpital Saint-Louis. The tissue sections were reviewed by the Head of Histopathology of Hôpital Saint-Louis; and classified according to the Bethesda proposal [35], where blast equivalents are designated as “Immature Forms/Blasts”, which for convenience, are referred to as blasts herein. 

Percentage blasts were determined from the BM smears by counting 100–200 cells. Lineage negative (Lin-) fractions were separated using an AutoMacs separator (Miltenyi, Auburn, CA). The lineage depletion kit contained a mixture of specific biotinylated antibodies to CD5 (T-cell antigen), CD45R (lymphocyte antigen), Mac-1, Gr-1(Lys-6G) (granulo-macrophagic differentiation antigens) and Ter119 (early erythroid antigen). BM Lin-/Sca1+/Kit+ (LSK) cells were estimated using Sca-1 conjugated antibodies with fluorescent isothiocyanate (FITC) and KIT conjugated with phycoerythrin (PE) (Becton Dickinson, San Jose, CA, USA). Livers and spleens were fixed overnight in buffered formalin and embedded in paraffin, sectioned and stained by the Hôpital Saint-Louis Histopathology department. Splenocytes were obtained by soft dilaceration of the spleen, washed in PBS, filtered through a 40 μm nylon mesh. Then density centrifugation was conducted using Lymphoprep (Eurobio, Paris, France) to isolate mononuclear splenocytes. Cell death of BM and spleen cells were measured using Annexin V/7-aminoactinomycin D (7-AAD) staining and flow cytometry. Briefly, single-cell suspensions were prepared from spleen cells and cultured in a low serum/cytokine mixture (IMDM/2% FCS/IL-3/SCF/G-CSF/GM-CSF (5 ng/mL)) for 24 h as recommended by the manufacturer (BD Biosciences, Oxford, UK). Three × 10^5^ cells were collected by centrifugation and labeled with Annexin V-Cy5 (BioVision Inc, Milpitas, CA, USA) and 7-AAD (BD Biosciences, Oxford, UK) according to manufacturers’ instructions. Apoptosis was followed using the Incucyte^®^ Zoom System (Essen Bioscience, Ann Arbour, MICH, USA) using manufacturer’s protocols; reagents for apoptosis, caspase 3 conjugated with a fluorescent green dye and annexin V conjugated with a cyanine fluorescent red dye.

### 4.4. Secondary Transplantation

Spleen cells (10^7^) from untreated and 33-day ABT-737 treated mice were injected intravenously into lethally irradiated (5 Grays + 5 Grays with an interval of 4 h) syngeneic FVB/N mice. These recipient mice were not treated. Three weeks after transplantation BCL-2 expression was measured by flow cytometry. Donor Sca1+ spleen cells have the LICs, as secondary transplants show that the spleen alone can give rise to the disease [36].

### 4.5. Progenitor Colony Assay

Colony assays were performed using the Methocult^®^ media as recommended by the manufacturer (Stem Cell Technologies, Vancouver, BC, Canada) and as described previously [7]. This kit contained rm-Stem Cell Factor, rmIL-3, rhIL-6 growth factors and insulin and transferrin. Briefly, 10^6^ bone marrow cells were centrifuged and resuspended in 3.3 mL Iscove’s media supplemented with 2% heat-inactivated fetal calf serum (FCS), 2 mM glutamine, 5 UI/mL penicillin, 300 mg/mL streptomycin. 0.3 mL of cells was added to 3 mL of Methocult^®^ and 1 mL (3 × 10^4^ cells) was plated per 35 mm dish (in triplicate). Cultures were incubated for 7 days at 37 °C, 5% CO2 in air and >95% humidity. Identification and counts of colonies were undertaken (according to the manufacturer’s instructions). Colonies were counted on day 7 and the mean of the first two dishes plated was scored.

### 4.6. Immunofluorescence and Confocal Microscopy

Immunofluorescence and microscopy were undertaken as previously described [8]. Briefly, a TRITC directly conjugated hBCL-2 (Santa Cruz Biotechnology, Dallas, TX, USA), an anti-NRAS monoclonal antibody was visualized with a goat anti-mouse Alexa 647 secondary antibody and anti-mitochondria antibody Tom 20 (Santa Cruz Biotechnology, Dallas, TX, USA) visualized with a goat anti-rabbit Alexa 488 secondary antibody. The fluorescent lectin (Alexa Fluor 488) wheat germ agglutinin (Molecular Probes, Invitrogen, Paisley, OR, USA) was used as a plasma membrane marker for mouse cells. Slides were analyzed by confocal microscopy on a Zeiss LSM 510 META confocal laser microscope (Zeiss, Jena, Germany).

### 4.7. Mitochondrial Membrane Potential (MMP)

MMP was measured using DiOC2(3) according to the manufacturer’s instructions (Invitrogen, Carlsbad, CA, USA) as we described previously [9]. Briefly, splenocytes were resuspended at 1 × 10^6^/mL in 1 × PBS with 50 nM DiOC2(3) (Invitrogen, Carlsbad, CA, USA) and incubated at 37 °C for 30 min. Cells were washed in 1 × PBS and analyzed by flow cytometry using the 488 nm excitation laser and 530/30 nm bandpass and 670 nm long pass filters. The accumulation of the DiOC2(3) dye [37] within the mitochondria measured by emission in the green/red channels following excitation reflects the membrane potential. Background corrected mean fluorescence intensity (MFI) using unstained cells was used to calculate the FL3/FL1 ratios of DiOC2(3) test samples as previously described [37].

### 4.8. SPECT

#### 4.8.1. ANX-Labeling

^99m^Tc-Annexin-V (ANX), which allows non-invasive in vivo detection of apoptosis, was prepared by injecting sodium pertechnetate (400 ± 20 Mega Becquerel [MBq] drawn up from a ^99m^Tc generator, freshly eluted) with stannous tricine in a sterile vial containing annexin V (Theseus Imaging Corp, Cambridge, Boston, MA, USA). After shaking, the preparation was left to stand 15 min at room temperature. The quality control was performed with instant thin-layer chromatography, using citric acid/dextrose solution as eluant. The radiolabeling yield was always greater than 88%.

#### 4.8.2. ANX-Scintigraphy

Scintigraphic imaging or Single-Photon Emission Computed Tomography (SPECT) was performed under pentobarbital anesthesia (4 mg/100 g body weight; Ceva Santé Animale, Libourne, France) in mice, 10 min after intravenous injection of ANX, as previously described [9]. Three consecutive mod-list planar acquisitions were performed, centered on the abdomen. In addition, mice that had previously undergone planar imaging underwent abdominal X/tomoscintigraphy acquisition; mod-list tomographic acquisition was performed during continuous rotation of the animal placed between 2 parallel collimators (360° rotation per minute, acquisition duration: 60 min from 1 h to 2 h after ANX injection). All acquisitions were performed using a dedicated small animal IMAGER-S/CT system (Biospace Mesures, Paris, France) equipped with parallel low-energy high-resolution collimators (matrix 128 × 128, 15% energy window centred on 140 KeV). ANX uptake in the hepato-splenic area was visually assessed, and activity (mean counts per pixel) ratios between pre- and post-treatment (determined on early dynamic images) and underlying background areas were computed on planar images.

### 4.9. TUNEL

Apoptosis was assessed by in situ detection of fragmented DNA, using the TUNEL assay on 5-μm paraffin-embedded liver sections as previously described [7,9]. Briefly, quantitative data on tissue sections were assessed blindly by two pathologists (AJ, CL) on an Olympus ProvisAX-70 microscope (Olympus, Tokyo, Japan), with wide-field eyepiece number 26.5, providing a field size of 0.344 mm^2^ at 400× magnification. Cell counts were performed on three different fields per section, and expressed as the mean number of cells per field (400× magnification) using the Olympus SIS software system.

### 4.10. RAS Activation Assays and Western Blotting

Briefly, 5 × 10^5^ cells were lysed in 50 mmol/L Tris (pH 7.4), 1% NP40, 15% glycerol, 200 mmol/L NaCl, 5 mmol/L MgCl_2_, 5 mmol/L NaF, 1 µmol/L leupeptin, 0.1 µmol/L aprotinin, and 1 mmol/L phenylmethylsulfonyl fluoride. Detergent-insoluble material was removed by centrifugation (16,000× *g* at 4 °C for 20 min) and assayed for RAS activation using the GSTRaf1-Ras Binding Domain protein as previously described [7,9].

### 4.11. Cell Preparation and RNA Extraction

Spleen cells were labeled with anti-Sca-1+ antibodies coupled with microbeads from Miltenyi and then sorted using an AutoMacs separator (Miltenyi Biotec, Bergisch Gladbach, Germany). 5 × 10^6^ to 10^7^ Sca-1+ sorted cells were used to extract total RNA using TRIzol (Invitrogen, Carlsbad, CA, USA). Quantification and quality of the RNAs were assessed using a Nanodrop (Thermo Fisher Scientific, Waltham, MA, USA).

### 4.12. Affymetrix Exon Array Hybridization

Affymetrix Mouse Exon 1.0 ST arrays were hybridized by GenoSplice technology (www.genosplice.com, accessed on 13 August 2010) according to the Ambion WT protocol (Life Technologies, Paris, France) and Affymetrix (Santa Clara, CA, USA) labeling and hybridization recommendations as previously described [9,36]. Targets were finally prepared according to Affymetrix recommendations for hybridization of exon arrays. Microarrays were hybridized, washed and scanned using Affymetrix instruments. Total RNAs RIN values were between 8.3 and 9. Raw data were controlled with the Expression Console (Affymetrix).

### 4.13. Array Data

Affymetrix Mouse Exon 1.0 ST Array dataset analysis and visualization were made using EASANA^®^ (GenoSplice Technology, Paris, France), which is based on the GenoSplice’s FAST DB^®^ annotations as previously described [9,36]. Exon Array data were normalized using quantile normalization. Background correction was made by using the antigenomic probes and probe selection was made as described previously [38]. Only probes targeting exons annotated from FAST DB^®^ transcripts were selected in order to focus on well-annotated genes, whose mRNA sequences are in public databases [39]. Among these selected probes, bad-quality probes (e.g., probes identified by Affymetrix as “cross-hybridizing”) and probes with too low of an intensity signal compared to antigenomic background probes with the same GC content were removed from the analysis. Only probes with a DABG *p*-value ≤ 0.05 in at least half of the arrays were considered for statistical analysis [39]. Only genes expressed in at least one compared condition were analyzed (i.e., untreated and ABT-737 treated mice). To be considered as expressed, the DABG *p*-value had to be ≤0.05 for at least half of the gene probes. We performed an unpaired Student’s t-test to compare gene intensities in the different biological replicates. Significant KEGG pathways [40] were retrieved using DAVID [41].

### 4.14. RQ-PCR

Primers were designed to *ATP6v0b*, *Pten*, *Usp46* and *Gpr125* (Supplementary Table S1).

### 4.15. Statistical Analysis

Survival from date of diagnosis (reduction of platelet count to below 1000 × 10^3^/mm^2^ at three to six months from birth) of treated mice (*n* = 34) was compared with untreated controls (*n* = 30) by the Kaplan Meier method with the log-rank test of significance. Mice were killed under veterinary advice, blinded to treatment status (at the end of treatment and due to disease; all shown to have advanced disease at necropsy). The combined endpoint (died naturally or killed due to advanced disease) of untreated and treated mice was used for comparisons.

Blood counts, LSK, progenitor, apoptosis and MMP assays were presented as means (±standard deviation) and between-group differences as mean difference (95% confidence interval of difference) and the unpaired Student’s *t*-test was used where >10 mice per group were assayed. Where <10 mice per group were assayed, the Mann–Whitney test was used.

## 5. Conclusions

The combination of ABT-737 with chemotherapeutic agents [42,43,44] or other targeted therapy molecules [45,46] has synergistic efficacy and is a promising molecule for treating BCL-2 dependent solid tumors and hematologic malignancies [47]. ABT-737 targets leukemia-initiating cells of our HR-MDS mouse model with biomarkers of response showing the rescue of disease, with implications in the stratification and treatment strategies of malignant haematopoietic syndromes with BH3 mimetics.

## Figures and Tables

**Table 1:** Target genes regulated after ABT-737 of HR-MDS mice. Fold change of treated compared to untreated mice was considered significant when ≥1.5 and *p*-value ≤ 0.05. The arrays were normalized to FVB/N, and the treated HR-MDS group was compared to the untreated HR-MDS.

Apoptosis related genes regulated			
Gene symbol	Regulation	Fold change	*p* value
Sgk1	up	1.67	2.05 × 10^−2^
E2f2	up	2.06	2.12 × 10^−2^
Osm	up	2.26	3.18 × 10^−2^
Rybp	up	1.85	3.48 × 10^−2^
Ripk3	up	1.68	3.62 × 10^−2^
Rnf130	up	1.57	4.72 × 10^−3^
Gpx1	up	1.76	7.96 × 10^−3^
Bcl2l12	up	1.79	1.63 × 10^−2^
App	up	2.46	3.79 × 10^−2^
Tnfrsf21	up	2.02	4.42 × 10^−2^
Pdcd5	up	1.65	1.82 × 10^−2^
Apaf1	up	1.62	3.56 × 10^−2^
Mfsd10	up	1.53	1.33 × 10^−2^
Pdcl3	up	1.50	2.26 × 10^−3^
Ckap2	up	1.91	2.50 × 10^−2^
Casp1	up	1.77	2.25 × 10^−3^
Bcl2a1a	up	2.00	2.85 × 10^−2^
G2e3	up	1.89	1.74 × 10^−3^
Birc5	up	2.45	2.10 × 10^−2^
C1d	up	1.52	1.07 × 10^−2^
Sgpl1	up	1.56	3.36 × 10^−2^
Bak1	up	1.97	1.49 × 10^−2^
Hipk2	up	1.51	3.38 × 10^−2^
Naip2	up	1.81	2.77 × 10^−3^
Rnf144b	up	1.51	1.28 × 10^−2^
Pten	up	1.98	4.07 × 10^−3^
Csf2rb	up	2.48	1.18 × 10^−2^
Pik3cg	up	1.62	3.48 × 10^−3^
Chp1	up	1.85	5.80 × × 10^−4^
Csf2rb2	up	1.73	2.07 × 10^−2^
Cxcr2	up	4.98	1.37 × 10^−2^
Wwox	down	1.60	5.05 × 10^−3^
Eif2ak3	down	1.54	9.38 × 10^−3^
Fasl	down	2.33	3.27 × 10^−2^
Lck	down	1.68	3.46 × 10^−2^
Dyrk2	down	1.63	8.96 × × 10^−4^
Trib3	down	1.51	1.59 × 10^−2^
Sod1	down	1.54	4.83 × × 10^−4^
Fas	down	1.54	2.01 × 10^−2^
Csrnp2	down	1.7	2.16 × 10^−2^
**Splice related genes regulated**			
**Gene Symbol**	**Regulation**	**Fold-Change**	***p*-Value**
Rsrc1	up	1.55	3.59 × 10^−2^
Gemin6	up	1.97	2.50 × 10^−2^
Gemin7	up	1.66	5.08 × 10^−3^
Lgals3	up	4.29	1.75 × 10^−2^
Lsm6	up	1.62	3.69 × 10^−2^
Zrsr2	up	1.71	4.37 × 10^−2^
Snrnp27	up	1.59	3.39 × 10^−2^
Snrpd2	up	1.76	4.59 × 10^−2^
Lsm10	up	1.90	1.28 × 10^−3^
Wbp4	up	1.85	1.67 × 10^−2^
Isy1	down	1.62	3.51 × 10^−2^
Pnn	down	1.91	1.19 × 10^−2^
Prpf38b	down	1.54	1.56 × 10^−2^
Rbm20	down	1.73	1.36 × 10^−2^
Rbfox1	down	1.50	6.72 × 10^−3^
Snrnp48	down	1.50	3.85 × 10^−2^
Tut1	down	1.73	2.19 × 10^−2^
**Cell cycle related genes regulated**			
**Gene symbol**	**Regulation**	**Fold change**	***p* value**
Cdk2	up	1.72	1.10 × 10^−2^
Ccne2	up	1.76	2.18 × 10^−2^
Cdc6	up	1.51	4.07 × 10^−2^
E2f2	up	2.06	2.12 × 10^−2^
Mapk13	up	3.06	3.22 × 10^−2^
Ccnb2	up	2.25	3.62 × 10^−2^
Cdca8	up	1.90	4.16 × 10^−2^
Ccna2	up	2.51	2.04 × 10^−2^
Cdk1	up	2.27	9.20 × 10^−3^
Cdkn2d	up	2.01	5.12 × 10^−3^
Cdkn2c	up	2.08	1.20 × 10^−2^
Ccnd3	up	1.88	2.09 × 10^−2^
Cdca3	up	2.24	1.91 × 10^−2^
Cdc25a	up	1.92	1.95 × 10^−2^
Cdkn3	up	2.43	2.91 × 10^−2^
Mapk3	up	1.89	2.36 × 10^−2^

## Data Availability

The data discussed in this publication have been deposited in NCBI’s Gene Expression Omnibus and are accessible through GEO Series accession number GSE19429.

## Supplementary Materials

The following are available online at https://www.mdpi.com/article/10.3390/ijms221910658/s1.
