# Supplementary material for: BCL-2 Inhibitor ABT-737 Effectively Targets Leukemia-Initiating Cells with Differential Regulation of Relevant Genes Leading to Extended Survival in a NRAS/BCL-2 Mouse Model of High Risk-Myelodysplastic Syndrome
Source: Int J Mol Sci. 2021 Sep 30;22(19):10658. doi: 10.3390/ijms221910658 (PMC8508829; doi:10.3390/ijms221910658)
Supplement: Supplementary file 1 [file ijms-22-10658-s001.zip › ijms-1334103-supplementary.pdf]

Table S1. Primer sequences for RQ-PCR validations

Target Gene	Foward	Reverse
Abl	GAAGACCTTGAAGGAGGACACCATG	GGGTACACACCCCTAGCAGCT
Pten	GAAAGGGACGGACTGGTGTA	TCTTGTGAAACAGCAGTGCC
Atp6v0b	GGCCATCGAAACTACCATGC	ACTGCCAAAGATCTCCACGA
Gpr125	CATGCTTGTGAACCTGTGCT	CTAGCAGTGACTCCAACCCA
Usp46	ATGTTGCAGCAAACAGGAGG	CAACCAGGTCATACATGCGG

Supplementary Table S2. 1952 Genes regulated post ABT-737 treatment of HR-MDS mice (Fold-change ≥ 1.5; P-Value ≤ 0.05)

FAST DB STABLE ID	Gene Symbol	Regulation	Fold-Change	P-Value	Gene Coordinates (mm10)	UCSC Link	Gene Name	Representative Transcript ID	Entrez Gene ID	EnsEMBL ID	log^2 intensity of untreated	log^2 intensity of treated	Low-specificity probe included
GSMG0051096	Xist	up	91.01	8.06E-08	chrX(-):103460373-103483233	UCSC	inactive X specific transcripts	NR_001570	213742	ENSMUSG00000086503	5.4	11.9	No
GSMG0041991	---	up	21.4	1.04E-03	chrX(+):103469447-103473001	UCSC	---	AK145665	---	---	5.55	9.97	Yes
GSMG0039124	Mmp8	up	8.27	3.45E-02	chr9(+):7558429-7568486	UCSC	matrix metallopeptidase 8	NM_008611	17394	ENSMUSG00000005800	8.57	11.62	No
GSMG0014944	2010005H15Rik	up	6.46	1.68E-03	chr16(+):36221562-36257427	UCSC	RIKEN cDNA 2010005H15 gene	NM_029733	76770	ENSMUSG00000095620	7.13	9.83	Yes
GSMG0032427	Tuba8	up	5.68	8.52E-03	chr6(+):121210770-121226854	UCSC	tubulin, alpha 8	AK032157	53857	ENSMUSG00000030137	5.79	8.29	No
GSMG0018859	Cd14	up	5.34	2.15E-02	chr18(-):36725067-36726742	UCSC	CD14 antigen	AK149744	12475	ENSMUSG00000051439	7.69	10.1	Yes
GSMG0000416	Cxcr2	up	5.14	1.46E-02	chr1(+):74153988-74161246	UCSC	chemokine (C-X-C motif) receptor 2	NM_009909	12765	ENSMUSG00000026180	9.24	11.61	No
GSMG0038900	Hp	up	4.98	4.34E-02	chr8(-):109575128-109579172	UCSC	haptoglobin	NM_017370	15439	ENSMUSG00000031722	9.13	11.44	No
GSMG0032455	Clec4d	up	4.83	8.16E-03	chr6(+):123262107-123275268	UCSC	C-type lectin domain family 4, member d	NM_010819	17474	ENSMUSG00000030144	7.77	10.04	No
GSMG0024587	S100a6	up	4.82	2.97E-02	chr3(+):90612894-90614414	UCSC	S100 calcium binding protein A6 (calcyclin)	NM_011313	20200	ENSMUSG00000001025	9.59	11.86	Yes
GSMG0017195	Mmp25	up	4.61	4.55E-02	chr17(-):23629179-23645270	UCSC	matrix metallopeptidase 25	BC059059	240047	ENSMUSG00000023903	8.16	10.37	No
GSMG0010552	F13a1	up	4.61	4.10E-03	chr13(-):36867178-37050244	UCSC	coagulation factor XIII, A1 subunit	NM_001166391	74145	ENSMUSG00000039109	7.19	9.39	No
GSMG0016285	Fpr3 // Fpr2	up	4.59	2.87E-02	chr17(+):17887822-17971677	UCSC	formyl peptide receptor 3 // formyl peptide receptor 2	U78299	14289 // 14294	USG00000052270 // ENSMUSG00000	9.94	12.13	Yes
GSMG0035940	Saa3	up	4.57	4.64E-03	chr7(-):46711998-46715676	UCSC	serum amyloid A 3	NM_011315	20210	ENSMUSG00000040026	6.9	9.09	No
GSMG0025365	Slc7a11	up	4.49	4.35E-02	chr3(-):49892525-50443614	UCSC	solute carrier family 7 (cationic amino acid transporter, y+ system), member	AY766237	26570	ENSMUSG00000027737	7.64	9.81	No
GSMG0011536	Ear6	up	4.47	1.56E-02	chr14(+):51853768-51854640	UCSC	eosinophil-associated, ribonuclease A family, member 6	NM_053111	93719	ENSMUSG00000062148	5.91	8.07	No
GSMG0014941	Stfa2l1	up	4.39	3.68E-02	chr16(+):36156810-36161952	UCSC	stefin A2 like 1	AK050597	268885	ENSMUSG00000059657	8.21	10.35	No
GSMG0036953	1810033B17Rik	up	4.37	4.89E-02	chr8(+):3665754-3669259	UCSC	RIKEN cDNA 1810033B17 gene	AK087121	69189	ENSMUSG00000013974	8.59	10.72	Yes
GSMG0014943	Gm5416	up	4.32	2.44E-02	chr16(+):36210403-36217788	UCSC	predicted gene 5416	NM_001082542	408196	ENSMUSG00000094733	5.24	7.35	No
GSMG0011490	Lgals3	up	4.29	1.75E-02	chr14(+):47373860-47386167	UCSC	lectin, galactose binding, soluble 3	NM_010705	16854	ENSMUSG00000050335	8.79	10.89	No
GSMG0012402	Cebpe	up	4.28	3.01E-02	chr14(-):54710361-54712175	UCSC	CCAAT/enhancer binding protein (C/EBP), epsilon	AK143561	110794	ENSMUSG00000052435	6.92	9.01	No
GSMG0007122	Ccl6	up	4.23	3.07E-02	chr11(-):83587886-83593087	UCSC	chemokine (C-C motif) ligand 6	NM_009139	20305	ENSMUSG00000018927	7.91	9.99	No
GSMG0014577	Nfe2	up	4.14	1.05E-02	chr15(-):103248220-103255428	UCSC	nuclear factor, erythroid derived 2	NM_008685	18022	ENSMUSG00000058794	7.25	9.3	No
GSMG0005639	Ccl2	up	4.14	2.27E-02	chr11(+):82035575-82037452	UCSC	chemokine (C-C motif) ligand 2	AK153520	20296	ENSMUSG00000035385	6.07	8.12	No
GSMG0033770	Klra2	up	4.07	3.24E-03	chr6(-):131219235-131247362	UCSC	killer cell lectin-like receptor, subfamily A, member 2	NM_008462	16633	ENSMUSG00000030187	6.41	8.43	No
GSMG0010105	---	up	4.06	4.03E-02	chr13(+):97751111-97760965	UCSC	---	AK144398	---	---	8.19	10.21	No
GSMG0003712	Cd63	up	4.05	1.46E-02	chr10(+):128908919-128912825	UCSC	CD63 antigen	NM_001042580	12512	ENSMUSG00000025351	6.95	8.96	No
GSMG0043598	Mrgpra2b // Mrgpra2a	up	4	2.65E-02	chr7(-):47426331-47489588	UCSC	MAS-related GPR, member A2B // MAS-related GPR, member A2A	NM_001172588	235712 // 668727	USG00000093973 // ENSMUSG00000	8.5	10.5	Yes
GSMG0014713	Cebpd	up	3.98	1.58E-03	chr16(+):15887006-15891031	UCSC	CCAAT/enhancer binding protein (C/EBP), delta	AK149723	12609	ENSMUSG00000071637	7.31	9.3	No
GSMG0020586	Csf2ra	up	3.94	8.54E-03	chr19(-):61223957-61228418	UCSC	colony stimulating factor 2 receptor, alpha, low-affinity (granulocyte-macroph	NM_009970	12982	ENSMUSG00000059326	9.45	11.43	No
GSMG0021673	---	up	3.92	1.70E-02	chr2(+):121778615-121779421	UCSC	---	AK015054	---	---	5.22	7.19	No
GSMG0021700	AA467197	up	3.87	2.50E-02	chr2(+):122637887-122641084	UCSC	expressed sequence AA467197	NM_001004174	433470	ENSMUSG00000033213	7.38	9.34	No
GSMG0009465	Inhba	up	3.78	4.04E-02	chr13(+):16011851-16027260	UCSC	inhibin beta-A	AK134991	16323	ENSMUSG00000041324	7.38	9.3	No
GSMG0004336	Uqcr11	up	3.73	1.27E-02	chr10(-):80402997-80406831	UCSC	ubiquinol-cytochrome c reductase, complex III subunit XI	AK003033	66594	ENSMUSG00000020163	6.64	8.54	No
GSMG0007503	5330430P22Rik	up	3.72	1.26E-03	chr11(-):106065086-106065932	UCSC	RIKEN cDNA 5330430P22 gene	AK019365	---	ENSMUSG00000085255	6.9	8.79	No
GSMG0052112	Gm15931	up	3.67	2.42E-03	chr7(+):4274143-4282933	UCSC	predicted gene 15931	NM_008848	18729	ENSMUSG00000081723	6.4	8.28	No
GSMG0043179	---	up	3.64	4.85E-02	chr13(+):21809791-21810171	UCSC	---	BC119519	---	---	8.61	10.47	No
GSMG0021315	Prg2	up	3.6	4.98E-02	chr2(+):84980459-84983632	UCSC	proteoglycan 2, bone marrow	AK010901	19074	ENSMUSG00000027073	8.82	10.66	Yes
GSMG0021177	Dhrs9	up	3.59	3.01E-02	chr2(+):69380443-69404533	UCSC	dehydrogenase/reductase (SDR family) member 9	NM_175512	241452	ENSMUSG00000027068	7.14	8.98	No
GSMG0000982	F5	up	3.57	1.84E-02	chr1(+):164151831-164220274	UCSC	coagulation factor V	AK137537	14067	ENSMUSG00000026579	6.9	8.74	No
GSMG0020184	Gda	up	3.53	4.48E-02	chr19(-):21391307-21473448	UCSC	guanine deaminase	NM_010266	14544	ENSMUSG00000058624	8.44	10.26	No
GSMG0038337	Mtus1	up	3.49	1.94E-02	chr8(-):40990912-41133727	UCSC	mitochondrial tumor suppressor 1	NM_001005863	102103	ENSMUSG00000045636	7.97	9.77	No
GSMG0037297	Scrg1	up	3.49	1.35E-04	chr8(+):57455923-57477586	UCSC	scrapie responsive gene 1	NM_009136	20284	ENSMUSG00000031610	4.8	6.6	Yes
GSMG0050187	---	up	3.48	2.12E-02	chr9(+):72170127-72175780	UCSC	---	DQ072390	---	---	9.74	11.54	Yes
GSMG0011530	Ear5	up	3.46	1.58E-03	chr14(+):51162260-51163018	UCSC	eosinophil-associated, ribonuclease A family, member 5	NM_019398	54159	ENSMUSG00000059606	6.69	8.48	Yes
GSMG0007196	Epx	up	3.45	3.73E-02	chr11(-):87863998-87875536	UCSC	eosinophil peroxidase	NM_007946	13861	ENSMUSG00000052234	6.86	8.65	No
GSMG0035854	---	up	3.39	4.06E-02	chr7(-):43524216-43526379	UCSC	---	AK149443	---	---	8.2	9.96	No
GSMG0023045	---	up	3.39	1.60E-02	chr2(-):80836946-80838042	UCSC	---	AK019820	---	---	8.52	10.28	Yes
GSMG0036829	Adam8	up	3.37	4.26E-02	chr7(-):139978933-139992538	UCSC	a disintegrin and metallopeptidase domain 8	BC025584	11501	ENSMUSG00000025473	8.25	10	No
GSMG0017852	Xdh	up	3.36	3.17E-02	chr17(-):73883899-73950198	UCSC	xanthine dehydrogenase	AK160010	22436	ENSMUSG00000024066	8.33	10.07	No
GSMG0015484	Fgd4	up	3.34	4.15E-02	chr16(-):16416917-16600552	UCSC	FYVE, RhoGEF and PH domain containing 4	BC129903	224014	ENSMUSG00000022788	7.67	9.41	No
GSMG0039746	Anxa2	up	3.33	1.63E-02	chr9(+):69453681-69491793	UCSC	annexin A2	AK012563	12306	ENSMUSG00000032231	8.82	10.56	No
GSMG0035647	Ceacam1	up	3.3	7.44E-03	chr7(-):25461702-25489494	UCSC	carcinoembryonic antigen-related cell adhesion molecule 1	AK085408	26365	ENSMUSG00000074272	10.23	11.95	No
GSMG0026746	BC042782	up	3.28	3.23E-02	chr4(+):95075669-95167318	UCSC	cDNA sequence BC042782	BC042782	---	ENSMUSG00000087366	5.11	6.82	Yes
GSMG0027198	---	up	3.27	2.83E-02	chr4(+):134003363-134004885	UCSC	---	AF285579	---	---	5.74	7.45	No
GSMG0016446	Mapk13	up	3.25	3.15E-02	chr17(+):28769317-28778704	UCSC	mitogen-activated protein kinase 13	NM_011950	26415	ENSMUSG00000004864	7.83	9.53	No
GSMG0037896	Crispld2	up	3.24	1.62E-02	chr8(+):119992470-120052792	UCSC	cysteine-rich secretory protein LCCL domain containing 2	NM_030209	78892	ENSMUSG00000031825	6.85	8.55	No
GSMG0005663	1700020L24Rik	up	3.23	4.12E-02	chr11(+):83437693-83441232	UCSC	RIKEN cDNA 1700020L24 gene	AK006174	66330	ENSMUSG00000035085	7.69	9.38	No
GSMG0003800	Rab32	up	3.23	6.82E-04	chr10(-):10545011-10558234	UCSC	RAB32, member RAS oncogene family	AK088661	67844	ENSMUSG00000019832	7.91	9.61	No
GSMG0032595	Mgst1	up	3.21	4.09E-02	chr6(+):138140536-138156752	UCSC	microsomal glutathione S-transferase 1	AK005122	56615	ENSMUSG00000008540	7.92	9.6	No
GSMG0032452	Clec4a2	up	3.2	2.38E-02	chr6(+):123122680-123143999	UCSC	C-type lectin domain family 4, member a2	NM_001170332	26888	ENSMUSG00000030148	8.14	9.82	No
GSMG0034305	Tyrobp	up	3.18	1.10E-02	chr7(+):30413788-30417577	UCSC	TYRO protein tyrosine kinase binding protein	NM_011662	22177	ENSMUSG00000030579	9.5	11.18	No
GSMG0003412	Hal	up	3.18	3.19E-02	chr10(+):93488768-93516761	UCSC	histidine ammonia lyase	NM_010401	15109	ENSMUSG00000020017	7.19	8.86	No
GSMG0036850	Ifitm2	up	3.17	1.02E-02	chr7(-):140954837-140955989	UCSC	interferon induced transmembrane protein 2	AK141392	80876	ENSMUSG00000060591	7.34	9.01	No
GSMG0002654	Hsd11b1	up	3.16	3.28E-03	chr1(-):193221633-193264047	UCSC	hydroxysteroid 11-beta dehydrogenase 1	NM_008288	15483	ENSMUSG00000016194	9.17	10.83	No
GSMG0032460	C1rl	up	3.16	3.36E-03	chr6(+):124493112-124510643	UCSC	complement component 1, r subcomponent-like	NM_181344	232371	ENSMUSG00000038527	6.6	8.26	No
GSMG0002446	Fcer1g	up	3.14	1.29E-02	chr1(-):171229574-171234349	UCSC	Fc receptor, IgE, high affinity I, gamma polypeptide	NM_010185	14127	ENSMUSG00000058715	10.01	11.67	No
GSMG0001931	Slco4c1	up	3.14	4.19E-02	chr1(-):96817731-96872171	UCSC	solute carrier organic anion transporter family, member 4C1	AK049944	227394	ENSMUSG00000040693	6.18	7.83	Yes
GSMG0028480	Lin28a	up	3.13	4.57E-02	chr4(-):134003330-134018816	UCSC	lin-28 homolog A (C. elegans)	NM_145833	83557	ENSMUSG00000050966	7.39	9.04	No
GSMG0043816	Pstpip2	up	3.12	3.49E-02	chr18(+):77794544-77882879	UCSC	proline-serine-threonine phosphatase-interacting protein 2	NM_013831	19201	ENSMUSG00000025429	7.5	9.14	No
GSMG0033561	---	up	3.12	1.68E-03	chr6(-):116101924-116103149	UCSC	---	AK079568	---	---	6.1	7.75	No
GSMG0024785	Adora3	up	3.07	1.67E-02	chr3(+):105870858-105924041	UCSC	adenosine A3 receptor	NM_027025	11542	ENSMUSG00000000562	6.17	7.78	No
GSMG0007084	Myo1d	up	3.06	1.42E-02	chr11(-):80482127-80780026	UCSC	myosin ID	NM_177390	338367	ENSMUSG00000035441	6.97	8.59	No
GSMG0029197	G6pd2	up	3.06	2.84E-02	chr5(+):61808843-61810477	UCSC	glucose-6-phosphate dehydrogenase 2	NM_019468	14380	ENSMUSG00000089992	9.76	11.37	Yes
GSMG0001035	Fcgr4	up	3.05	4.74E-02	chr1(+):171018920-171029761	UCSC	Fc receptor, IgG, low affinity IV	NM_144559	246256	ENSMUSG00000059089	8.06	9.67	No
GSMG0043894	Scarna6	up	3.05	6.62E-03	chr1(+):87784571-87784818	UCSC	small Cajal body-specific RNA 6	NR_028519	100217415	ENSMUSG00000089281	9.33	10.95	No
GSMG0017498	Rpp21	up	3.02	3.22E-03	chr17(-):36255645-36257862	UCSC	ribonuclease P 21 subunit	AY251017	67676	ENSMUSG00000024446	6.7	8.29	Yes
GSMG0030436	Prom1	up	3.02	3.48E-02	chr5(-):43993621-44102032	UCSC	prominin 1	BC028286	19126	ENSMUSG00000029086	6.12	7.71	Yes
GSMG0027128	Marcksl1	up	3.02	3.38E-02	chr4(+):129513581-129515981	UCSC	MARCKS-like 1	NM_010807	17357	ENSMUSG00000047945	8.42	10.02	Yes
GSMG0031852	Tmem176a	up	3.01	4.82E-02	chr6(+):48841483-48847048	UCSC	transmembrane protein 176A	AK079378	66058	ENSMUSG00000023367	7.76	9.34	No
GSMG0051073	Pira2	up	2.98	7.04E-03	chr7(-):3836810-3845053	UCSC	paired-Ig-like receptor A2	NM_011089	18725	ENSMUSG00000089942	6.15	7.73	Yes
GSMG0029122	Bst1	up	2.98	1.71E-02	chr5(+):43818893-43843468	UCSC	bone marrow stromal cell antigen 1	NM_009763	12182	ENSMUSG00000029082	7.28	8.86	No
GSMG0014232	Gm10865	up	2.95	4.48E-02	chr15(-):78994955-79011042	UCSC	predicted gene 10865	AK139534	100038602	---	7.54	9.1	No
GSMG0006964	---	up	2.95	4.93E-02	chr11(-):74515765-74520614	UCSC	---	AK141360	---	---	6.12	7.68	No
GSMG0012441	Mcpt8	up	2.94	4.68E-02	chr14(-):56082159-56085273	UCSC	mast cell protease 8	BC144934	17231	ENSMUSG00000022157	6.26	7.82	No
GSMG0034471	---	up	2.93	2.76E-03	chr7(+):45459469-45460300	UCSC	---	AK158602	---	---	10.38	11.93	Yes
GSMG0032691	Bet1	up	2.93	1.67E-02	chr6(-):4076904-4086949	UCSC	blocked early in transport 1 homolog (S. cerevisiae)	NM_009748	12068	ENSMUSG00000032757	7.11	8.65	No
GSMG0023520	Ckap2l	up	2.93	1.37E-02	chr2(-):129268211-129297212	UCSC	cytoskeleton associated protein 2-like	NM_181589	70466	ENSMUSG00000048327	6.99	8.54	No
GSMG0035423	Lilra6	up	2.92	2.28E-03	chr7(-):3908280-3915505	UCSC	leukocyte immunoglobulin-like receptor, subfamily A (with TM domain), memt	AK030846	18726	ENSMUSG00000030427	7.6	9.15	No
GSMG0022932	Spc25	up	2.91	2.60E-03	chr2(-):69193896-69206190	UCSC	SPC25, NDC80 kinetochore complex component, homolog (S. cerevisiae)	NM_025565	66442	ENSMUSG00000005233	7.46	9	No
GSMG0000665	Serpinb10	up	2.9	4.63E-02	chr1(+):107529001-107549271	UCSC	serine (or cysteine) peptidase inhibitor, clade B (ovalbumin), member 10	NM_198028	241197	ENSMUSG00000092572	6.86	8.4	No
GSMG0031251	Pilrb2	up	2.9	3.83E-02	chr5(-):137865829-137871759	UCSC	paired immunoglobin-like type 2 receptor beta 2	AK036467	545812	ENSMUSG00000066682	7.64	9.17	No
GSMG0031249	Pilra	up	2.9	1.70E-02	chr5(-):137821952-137836278	UCSC	paired immunoglobin-like type 2 receptor alpha	NM_153510	231805	ENSMUSG00000046245	9.09	10.63	No
GSMG0044801	---	up	2.89	3.52E-02	chr12(-):73946408-73947527	UCSC	---	AY279360	---	---	5.75	7.28	No
GSMG0006232	Slc16a3	up	2.89	3.92E-02	chr11(+):120948484-120960868	UCSC	solute carrier family 16 (monocarboxylic acid transporters), member 3	AK166048	80879	ENSMUSG00000025161	8.64	10.17	No
GSMG0009532	Hist1h3f	up	2.87	1.56E-03	chr13(+):23544052-23545314	UCSC	histone cluster 1, H3f	BC101956	260423	ENSMUSG00000059309	9.16	10.68	No
GSMG0007616	Cd300lf	up	2.85	4.34E-03	chr11(-):115116214-115133992	UCSC	CD300 antigen like family member F	NM_145634	246746	ENSMUSG00000047798	7.84	9.35	No
GSMG0036506	1600010M07Rik	up	2.83	3.85E-02	chr7(-):109998377-110151202	UCSC	RIKEN cDNA 1600010M07 gene	AK005418	69781	---	5.43	6.93	Yes
GSMG0017829	Emilin2	up	2.8	4.02E-02	chr17(-):71252173-71311557	UCSC	elastin microfibril interfacer 2	AK171303	246707	ENSMUSG00000024053	8.29	9.78	Yes
GSMG0039983	---	up	2.8	2.62E-03	chr9(+):100778789-100780692	UCSC	---	AK082274	---	---	5.05	6.54	Yes
GSMG0009542	Hist1h2ab	up	2.79	2.20E-02	chr13(+):23751088-23751593	UCSC	histone cluster 1, H2ab	NM_175660	319172	ENSMUSG00000061615	7.83	9.31	No
GSMG0038338	---	up	2.77	1.12E-02	chr8(-):41015505-41016553	UCSC	---	AK086990	---	---	6.09	7.56	No
GSMG0043178	Hist1h2ao	up	2.76	2.56E-03	chr13(+):21810367-21810885	UCSC	histone cluster 1, H2ao	BC147162	665433	ENSMUSG00000094248	7.62	9.08	No
GSMG0052223	Pira1	up	2.76	1.29E-02	chr7(-):3731634-3739870	UCSC	paired-Ig-like receptor A1	NM_011087	18722	---	8.3	9.76	Yes
GSMG0043152	Rpl26	up	2.76	4.03E-02	chr11(+):68901566-68904538	UCSC	ribosomal protein L26	NM_009080	19941	ENSMUSG00000060938	7.86	9.32	No
GSMG0006393	Ugp2	up	2.75	3.78E-03	chr11(-):21321144-21371207	UCSC	UDP-glucose pyrophosphorylase 2	NM_139297	216558	ENSMUSG00000001891	8.98	10.44	No
GSMG0030087	Alox5ap	up	2.75	3.91E-02	chr5(+):149265048-149288045	UCSC	arachidonate 5-lipoxygenase activating protein	AK004002	11690	ENSMUSG00000060063	10.6	12.06	No
GSMG0010522	---	up	2.74	2.12E-02	chr13(-):33562486-33569318	UCSC	---	AK146381	---	---	5.42	6.88	Yes
GSMG0009370	D430020J02Rik	up	2.73	1.78E-02	chr12(-):116401947-116405162	UCSC	RIKEN cDNA D430020J02 gene	NR_028421	319545	---	8.66	10.11	No
GSMG0019204	---	up	2.72	4.76E-02	chr18(-):82336250-82354011	UCSC	---	AK139683	---	---	9.05	10.49	No
GSMG0043657	Gm15832	up	2.72	3.08E-03	chr1(-):39547588-39552003	UCSC	predicted gene 15832	AK040602	---	ENSMUSG00000085894	5.16	6.61	Yes
GSMG0009954	Glrx	up	2.72	4.70E-02	chr13(+):75839886-75850151	UCSC	glutaredoxin	NM_053108	93692	ENSMUSG00000021591	9.43	10.87	No
GSMG0017686	Lrg1	up	2.71	2.54E-02	chr17(-):56119677-56121946	UCSC	leucine-rich alpha-2-glycoprotein 1	NM_029796	76905	ENSMUSG00000037095	7.97	9.41	No
GSMG0002194	Rgs18	up	2.7	2.35E-02	chr1(-):144752839-144775428	UCSC	regulator of G-protein signaling 18	AB042807	64214	ENSMUSG00000026357	8.15	9.58	No
GSMG0043594	---	up	2.7	4.27E-02	chr5(-):129622946-129623313	UCSC	---	AK191883	---	---	6.12	7.55	Yes
GSMG0051074	Gm14548	up	2.7	1.85E-02	chr7(-):3884211-3898125	UCSC	predicted gene 14548	U96688	100038909	ENSMUSG00000074417	8.75	10.19	Yes
GSMG0034055	Tmem160	up	2.7	5.06E-03	chr7(+):16452779-16455494	UCSC	transmembrane protein 160	NM_026938	69094	ENSMUSG00000019158	8.24	9.67	No
GSMG0033131	---	up	2.7	1.03E-02	chr6(-):69775744-69776036	UCSC	---	S60864	---	---	10.9	12.33	Yes
GSMG0016372	Prss34	up	2.68	2.30E-02	chr17(+):25298391-25300161	UCSC	protease, serine, 34	AK081986	328780	ENSMUSG00000056399	6.86	8.28	No
GSMG0029437	---	up	2.68	4.69E-02	chr5(+):93187453-93189231	UCSC	---	AK038292	---	---	6.93	8.35	No
GSMG0027858	Ptgr1	up	2.68	3.98E-03	chr4(-):58965440-58987115	UCSC	prostaglandin reductase 1	AK134440	67103	ENSMUSG00000028378	7.04	8.46	No
GSMG0032062	St3gal5	up	2.65	2.17E-02	chr6(+):72097606-72154571	UCSC	ST3 beta-galactoside alpha-2,3-sialyltransferase 5	NM_011375	20454	ENSMUSG00000056091	8.59	10	No
GSMG0032168	Nfu1	up	2.63	4.48E-03	chr6(+):87009835-87028460	UCSC	NFU1 iron-sulfur cluster scaffold homolog (S. cerevisiae)	NM_020045	56748	ENSMUSG00000029993	7.6	8.99	Yes
GSMG0025321	Ccna2	up	2.62	1.99E-02	chr3(-):36564866-36571999	UCSC	cyclin A2	AK044924	12428	ENSMUSG00000027715	8.77	10.16	No
GSMG0035420	Pirb	up	2.61	6.62E-03	chr7(-):3711409-3720391	UCSC	paired Ig-like receptor B	AK161081	18733	ENSMUSG00000058818	8.1	9.48	No
GSMG0043176	Hist1h2br	up	2.59	1.48E-03	chr13(+):21833741-21837530	UCSC	histone cluster 1 H2br	NM_001110555	665622	ENSMUSG00000069303	7.22	8.6	No
GSMG0025181	Sirpb1a	up	2.59	3.26E-03	chr3(-):15371827-15426427	UCSC	signal-regulatory protein beta 1A	NM_001002898	320832	ENSMUSG00000095788	9.01	10.38	No
GSMG0009540	Hist1h1c	up	2.58	2.45E-02	chr13(+):23738804-23740366	UCSC	histone cluster 1, H1c	AK155502	50708	ENSMUSG00000036181	9.53	10.9	No
GSMG0003541	---	up	2.58	1.11E-02	chr10(+):112837751-112845807	UCSC	---	AK036450	---	---	5.46	6.82	No
GSMG0003471	---	up	2.58	1.63E-02	chr10(+):99443704-99447007	UCSC	---	AK137453	---	---	6.64	8.01	Yes
GSMG0013349	Csf2rb	up	2.57	1.19E-02	chr15(+):78325798-78353847	UCSC	colony stimulating factor 2 receptor, beta, low-affinity (granulocyte-macropha	AK133420	12983	ENSMUSG00000071713	8.9	10.26	No
GSMG0012140	---	up	2.57	2.52E-02	chr14(-):25841434-25842484	UCSC	---	AK038929	---	---	6.45	7.81	Yes
GSMG0010374	Gpr141	up	2.57	6.86E-03	chr13(-):19749682-19824259	UCSC	G protein-coupled receptor 141	AK154688	353346	ENSMUSG00000053101	6.77	8.13	No
GSMG0006164	Birc5	up	2.57	2.09E-02	chr11(+):117849237-117855755	UCSC	baculoviral IAP repeat-containing 5	NM_009689	11799	ENSMUSG00000017716	7.37	8.73	No
GSMG0013007	---	up	2.55	5.74E-03	chr15(+):20449189-20979940	UCSC	---	AK084059	---	---	6.49	7.84	No
GSMG0036231	Fah	up	2.54	1.12E-02	chr7(-):84585162-84605942	UCSC	fumarylacetoacetate hydrolase	NM_010176	14085	ENSMUSG00000030630	7.05	8.4	No
GSMG0034068	Ptgir	up	2.54	4.75E-02	chr7(+):16906489-16910905	UCSC	prostaglandin I receptor (IP)	AK033567	19222	ENSMUSG00000043017	7.12	8.46	No
GSMG0011480	Cdkn3	up	2.53	2.91E-02	chr14(+):46760541-46771525	UCSC	cyclin-dependent kinase inhibitor 3	NM_028222	72391	ENSMUSG00000037628	7.37	8.71	Yes
GSMG0006265	Tcn2	up	2.53	1.23E-02	chr11(-):3917064-3932078	UCSC	transcobalamin 2	NM_001130458	21452	ENSMUSG00000020432	9.14	10.48	No
GSMG0004386	---	up	2.53	3.26E-02	chr10(-):82587408-82590263	UCSC	---	AK140463	---	---	5.85	7.18	No
GSMG0033649	C3ar1	up	2.53	4.06E-02	chr6(-):122847140-122856160	UCSC	complement component 3a receptor 1	NM_009779	12267	ENSMUSG00000040552	6.22	7.56	No
GSMG0058024	---	up	2.52	3.03E-02	chr6(-):8596926-8596946	UCSC	---	AB335256	---	---	7.77	9.1	Yes
GSMG0020318	Acta2	up	2.51	1.24E-03	chr19(-):34240336-34255373	UCSC	actin, alpha 2, smooth muscle, aorta	NM_007392	11475	ENSMUSG00000035783	7.01	8.34	No
GSMG0017538	---	up	2.51	4.50E-02	chr17(-):39848081-39848675	UCSC	---	BC127072	---	---	9.53	10.86	No
GSMG0014158	Gm10872	up	2.51	3.83E-02	chr15(-):76266787-76269840	UCSC	predicted gene 10872	NR_045747	100038659	---	7.04	8.36	Yes
GSMG0022279	Fam217b	up	2.51	2.42E-02	chr2(+):178422568-178424424	UCSC	family with sequence similarity 217, member B	AK018518	71532	ENSMUSG00000070476	6.83	8.16	No
GSMG0021633	Nusap1	up	2.5	1.56E-02	chr2(+):119618298-119651553	UCSC	nucleolar and spindle associated protein 1	AK042697	108907	ENSMUSG00000027306	7.45	8.77	No
GSMG0013871	Cox6c	up	2.5	1.32E-03	chr15(-):35931978-35938246	UCSC	cytochrome c oxidase subunit VIc	NM_053071	12864	ENSMUSG00000014313	8.55	9.87	No
GSMG0013456	Tspo	up	2.5	2.10E-03	chr15(+):83563573-83574202	UCSC	translocator protein	NM_009775	12257	ENSMUSG00000041736	8.85	10.18	No
GSMG0036573	Itpripl2	up	2.5	2.92E-02	chr7(-):118485114-118491979	UCSC	inositol 1,4,5-triphosphate receptor interacting protein-like 2	NM_001033380	319622	ENSMUSG00000095115	7.17	8.49	No
GSMG0030974	Cox6a1	up	2.5	1.28E-02	chr5(-):115345654-115348961	UCSC	cytochrome c oxidase subunit VIa polypeptide 1	AK019318	12861	ENSMUSG00000041697	8.75	10.07	No
GSMG0034162	Ceacam10	up	2.48	1.89E-02	chr7(+):24777204-24784653	UCSC	carcinoembryonic antigen-related cell adhesion molecule 10	NM_007675	26366	ENSMUSG00000054169	7.73	9.04	Yes
GSMG0031250	Pilrb1	up	2.48	3.11E-02	chr5(-):137852147-137858049	UCSC	paired immunoglobin-like type 2 receptor beta 1	NM_133209	170741	ENSMUSG00000066684	8.56	9.87	Yes
GSMG0010427	Hist1h2be	up	2.47	1.88E-02	chr13(-):23583670-23621127	UCSC	histone cluster 1, H2be	NM_178194	319179	ENSMUSG00000047246	6.97	8.27	No
GSMG0007120	Ccl9	up	2.47	4.29E-02	chr11(-):83572917-83578636	UCSC	chemokine (C-C motif) ligand 9	NM_011338	20308	ENSMUSG00000019122	8.4	9.71	Yes
GSMG0042305	---	up	2.47	2.97E-02	chrX(-):6068458-6071451	UCSC	---	AK030640	---	---	9.94	11.25	Yes
GSMG0022635	Swi5	up	2.47	7.08E-03	chr2(-):32278816-32288068	UCSC	SWI5 recombination repair homolog (yeast)	NM_175190	72931	ENSMUSG00000044627	8.2	9.51	No
GSMG0019253	1810055G02Rik	up	2.46	1.28E-02	chr19(+):3708331-3717881	UCSC	RIKEN cDNA 1810055G02 gene	AK007868	72056	ENSMUSG00000035372	6.03	7.33	No
GSMG0015570	Dgkg	up	2.46	1.87E-02	chr16(-):22466571-22657231	UCSC	diacylglycerol kinase, gamma	NM_138650	110197	ENSMUSG00000022861	7.38	8.69	No
GSMG0022154	Ube2c	up	2.43	4.68E-02	chr2(+):164769896-164778820	UCSC	ubiquitin-conjugating enzyme E2C	BC085107	68612	ENSMUSG00000001403	9.28	10.56	No
GSMG0016518	Myo1f	up	2.43	3.25E-02	chr17(+):33555707-33607764	UCSC	myosin IF	NM_053214	17916	ENSMUSG00000024300	8.71	9.99	No
GSMG0013362	Triobp	up	2.43	3.46E-03	chr15(+):78947724-79005869	UCSC	TRIO and F-actin binding protein	NM_138579	110253	ENSMUSG00000033088	8.46	9.74	No
GSMG0037822	---	up	2.43	4.36E-02	chr8(+):111540028-111542294	UCSC	---	AK154039	---	---	6.59	7.87	No
GSMG0021815	Smox	up	2.42	1.08E-02	chr2(+):131491862-131525922	UCSC	spermine oxidase	AK166350	228608	ENSMUSG00000027333	7.65	8.92	No
GSMG0000184	Il18rap	up	2.42	3.55E-02	chr1(+):40515362-40549305	UCSC	interleukin 18 receptor accessory protein	AF077347	16174	ENSMUSG00000026068	8.47	9.74	Yes
GSMG0013121	---	up	2.42	3.73E-02	chr15(+):38518785-38520066	UCSC	---	AK007010	---	---	7.2	8.47	No
GSMG0040456	Rab3d	up	2.42	4.70E-02	chr9(-):21907492-21918184	UCSC	RAB3D, member RAS oncogene family	AK048395	19340	ENSMUSG00000019066	6.95	8.23	No
GSMG0025488	Lxn	up	2.41	1.49E-02	chr3(-):67457997-67463908	UCSC	latexin	AK032170	17035	ENSMUSG00000047557	6.65	7.92	No
GSMG0033021	---	up	2.4	2.87E-02	chr6(-):51909747-51911184	UCSC	---	AK086100	---	---	6.5	7.76	Yes
GSMG0013608	Tuba1c	up	2.39	3.48E-03	chr15(+):99029891-99038110	UCSC	tubulin, alpha 1C	NM_009448	22146	ENSMUSG00000043091	10.27	11.53	No
GSMG0001517	Tbc1d8	up	2.39	1.79E-02	chr1(-):39371499-39478747	UCSC	TBC1 domain family, member 8	NM_018775	54610	ENSMUSG00000003134	8.35	9.6	No
GSMG0008590	Adssl1	up	2.39	1.79E-02	chr12(+):112620044-112641357	UCSC	adenylosuccinate synthetase like 1	NM_007421	11565	ENSMUSG00000011148	7.76	9.02	No
GSMG0033475	Prok2	up	2.38	1.64E-02	chr6(-):99711299-99726392	UCSC	prokineticin 2	NM_015768	50501	ENSMUSG00000030069	6.59	7.84	No
GSMG0003908	Arg1	up	2.38	2.62E-02	chr10(-):24915208-24927484	UCSC	arginase, liver	NM_007482	11846	ENSMUSG00000019987	6.03	7.29	Yes
GSMG0042485	Olfr1324	up	2.37	1.18E-02	chrX(-):50425431-50426582	UCSC	olfactory receptor 1324	BC120627	258289	ENSMUSG00000048173	5.95	7.19	No
GSMG0004222	Cdk1	up	2.37	9.36E-03	chr10(-):69336635-69352939	UCSC	cyclin-dependent kinase 1	AK030231	12534	ENSMUSG00000019942	7.84	9.08	No
GSMG0021383	Spi1	up	2.36	1.88E-02	chr2(+):91096096-91115756	UCSC	spleen focus forming virus (SFFV) proviral integration oncogene	X17463	20375	ENSMUSG00000002111	9.23	10.47	No
GSMG0013088	---	up	2.36	1.26E-02	chr15(+):34442979-34444812	UCSC	---	AK029389	---	---	9.92	11.16	No
GSMG0012532	Msra	up	2.36	2.92E-03	chr14(-):64122621-64455903	UCSC	methionine sulfoxide reductase A	NM_026322	110265	ENSMUSG00000054733	8.11	9.34	No
GSMG0043351	---	up	2.36	1.78E-05	chr7(+):136812434-136812560	UCSC	---	AK207713	---	---	7.31	8.55	Yes
GSMG0035094	Lyrm1	up	2.36	7.66E-03	chr7(+):119895858-119956963	UCSC	LYR motif containing 1	AK157931	73919	ENSMUSG00000030922	7.69	8.93	No
GSMG0032996	Tmem176b	up	2.36	4.64E-02	chr6(-):48833811-48841376	UCSC	transmembrane protein 176B	NM_001164208	65963	ENSMUSG00000029810	10.18	11.42	No
GSMG0027227	Ifnlr1	up	2.36	3.57E-02	chr4(+):135686456-135708181	UCSC	interferon lambda receptor 1	NM_174851	242700	ENSMUSG00000062157	6.43	7.67	No
GSMG0025180	Gm9733	up	2.36	6.58E-03	chr3(-):15296551-15332303	UCSC	predicted gene 9733	DQ055452	751864	ENSMUSG00000078783	5.93	7.17	Yes
GSMG0016182	Dynlt1b	up	2.35	1.36E-02	chr17(+):6430112-6436293	UCSC	dynein light chain Tctex-type 1B	NM_009342	21648	ENSMUSG00000096255	10.49	11.72	Yes

FAST DB STABLE ID	Gene Symbol	Regulation	Fold-Change	P-Value	Gene Coordinates (mm10)	UCSC Link	Gene Name	Representative Transcript ID	Entrez Gene ID	EnsEMBL ID	log2 intensity of untreated	log2 intensity of treated	Low-specificity probe included
GSMG0033686	Cd9	up	2.35	3.62E-03	chr6(-):125460266-125494760	UCSC	CD9 antigen	AK012793	12527	ENSMUSG00000030342	10.22	11.45	No
GSMG0033338	1700031F10Rik	up	2.35	4.40E-02	chr6(-):88222602-88225679	UCSC	RIKEN cDNA 1700031F10 gene	AK006579	---	ENSMUSG00000047046	8.15	9.38	Yes
GSMG0016686	Pla2g7	up	2.34	7.38E-03	chr17(+):43568449-43612193	UCSC	phospholipase A2, group VII (platelet-activating factor acetylhydrolase, plasn	NM_013737	27226	ENSMUSG00000023913	9.84	11.06	No
GSMG0010923	Vcan	up	2.34	1.42E-02	chr13(-):89655310-89742512	UCSC	versican	NM_019389	13003	ENSMUSG00000021614	6.48	7.71	No
GSMG0042616	G6pdx	up	2.34	4.83E-02	chrX(-):74409486-74429161	UCSC	glucose-6-phosphate dehydrogenase X-linked	NM_008062	14381	ENSMUSG00000031400	10.59	11.82	No
GSMG0024830	Slc25a24	up	2.34	1.15E-02	chr3(+):109123148-109168453	UCSC	solute carrier family 25 (mitochondrial carrier, phosphate carrier), member 24	AK146034	229731	ENSMUSG00000040322	8.69	9.92	No
GSMG0040970	Ccnb2	up	2.33	3.67E-02	chr9(-):70407689-70421554	UCSC	cyclin B2	NM_007630	12442	ENSMUSG00000032218	8.89	10.11	No
GSMG0004402	Ckap4	up	2.33	2.31E-02	chr10(-):84526305-84534036	UCSC	cytoskeleton-associated protein 4	NM_175451	216197	ENSMUSG00000046841	8.71	9.93	No
GSMG0032469	Cdca3	up	2.33	1.93E-02	chr6(+):124830176-124833701	UCSC	cell division cycle associated 3	NM_013538	14793	ENSMUSG00000023505	7.32	8.54	No
GSMG0000884	Fam129a	up	2.32	1.77E-02	chr1(+):151571373-151719346	UCSC	family with sequence similarity 129, member A	NM_022018	63913	ENSMUSG00000026483	8.78	9.99	No
GSMG0006996	Fam101b	up	2.32	5.88E-03	chr11(-):76019203-76027785	UCSC	family with sequence similarity 101, member B	NM_029658	76566	ENSMUSG00000020846	8.34	9.56	No
GSMG0025913	Gstm3	up	2.32	4.58E-02	chr3(-):107963696-107969174	UCSC	glutathione S-transferase, mu 3	NM_010359	14864	ENSMUSG00000004038	8.01	9.22	Yes
GSMG0024623	S100a11	up	2.32	5.52E-03	chr3(+):93520494-93526288	UCSC	S100 calcium binding protein A11 (calgizzarin)	NM_016740	20195	ENSMUSG00000027907	10.78	11.99	No
GSMG0019588	Trpd52l3	up	2.31	2.79E-02	chr19(+):30003790-30006020	UCSC	tumor protein D52-like 3	NM_025741	66745	ENSMUSG00000024815	7.03	8.24	Yes
GSMG0011280	Vcl	up	2.31	4.56E-02	chr14(+):20929397-21033673	UCSC	vinculin	NM_009502	22330	ENSMUSG00000021823	8.97	10.17	Yes
GSMG0034155	Ethe1	up	2.31	1.27E-02	chr7(+):24587543-24608923	UCSC	ethylmalonic encephalopathy 1	NM_023154	66071	ENSMUSG00000064254	8.04	9.24	No
GSMG0031745	Mgam	up	2.3	2.69E-02	chr6(+):40628831-40769123	UCSC	maltase-glucoamylase	NM_001171003	232714	ENSMUSG00000068587	6.84	8.04	No
GSMG0023476	Atp8b4	up	2.3	2.86E-02	chr2(-):126320973-126500669	UCSC	ATPase, class I, type 8B, member 4	AK155448	241633	ENSMUSG00000060131	8.23	9.44	No
GSMG0007612	Cd300ld	up	2.29	1.57E-02	chr11(-):114982274-114989922	UCSC	CD300 molecule-like family member d	AK028662	217305	ENSMUSG00000034641	7.4	8.6	No
GSMG0053369	Gm2012	up	2.29	6.04E-03	chrX(-):26200882-26231323	UCSC	predicted gene 2012	NM_001104946	100039030	ENSMUSG00000082639	7.08	8.27	Yes
GSMG0035835	---	up	2.29	2.48E-03	chr7(-):41060617-41089843	UCSC	---	AK007160	---	---	6.17	7.36	Yes
GSMG0027932	Megf9	up	2.29	4.82E-02	chr4(-):70428853-70534929	UCSC	multiple EGF-like-domains 9	AK122378	230316	ENSMUSG00000039270	8.52	9.72	No
GSMG0034268	Rasgrp4	up	2.28	4.84E-02	chr7(+):29134931-29153961	UCSC	RAS guanyl releasing protein 4	NR_045676	233046	ENSMUSG00000030589	7.69	8.89	No
GSMG0032451	Clec4b1	up	2.28	6.66E-03	chr6(+):123049962-123071555	UCSC	C-type lectin domain family 4, member b1	NM_027218	69810	ENSMUSG00000030147	5.98	7.17	Yes
GSMG0027584	Cpne3	up	2.28	1.44E-02	chr4(-):19519252-19570109	UCSC	copine III	NM_027769	70568	ENSMUSG00000028228	9.56	10.75	No
GSMG0035422	Gm10693	up	2.27	1.05E-02	chr7(-):3860681-3869153	UCSC	predicted pseudogene 10693	AK137448	675749	ENSMUSG00000074420	7.63	8.81	Yes
GSMG0051539	Gm6034	up	2.26	3.68E-02	chr17(+):36042961-36058645	UCSC	predicted gene 6034	NM_001034909	547347	ENSMUSG00000073407	8.3	9.47	No
GSMG0039583	Cox5a	up	2.26	4.92E-03	chr9(+):57521232-57532424	UCSC	cytochrome c oxidase subunit Va	NM_007747	12858	ENSMUSG00000000088	8.96	10.14	No
GSMG0036875	---	up	2.26	3.33E-02	chr7(-):141516368-141518603	UCSC	---	BC025551	---	---	6.36	7.53	Yes
GSMG0019310	Ap5b1	up	2.25	1.97E-02	chr19(+):5568022-5571261	UCSC	adaptor-related protein complex 5, beta 1 subunit	AK171710	381201	ENSMUSG00000049562	7.13	8.3	No
GSMG0029955	Lamtor4	up	2.25	5.04E-04	chr5(+):138255482-138259394	UCSC	late endosomal/lysosomal adaptor, MAPK and MTOR activator 4	NM_001081108	66096	ENSMUSG00000050552	8.34	9.52	No
GSMG0012816	Ednrb	up	2.24	2.70E-03	chr14(-):103814615-103844476	UCSC	endothelin receptor type B	NM_001136061	13618	ENSMUSG00000022122	6.19	7.36	No
GSMG0012780	---	up	2.24	1.46E-02	chr14(-):98064815-98066901	UCSC	---	AK143255	---	---	5.98	7.15	Yes
GSMG0011330	Anxa11	up	2.24	3.00E-03	chr14(+):25842155-25886804	UCSC	annexin A11	NM_013469	11744	ENSMUSG00000021866	8.89	10.06	No
GSMG0031123	Chchd2	up	2.24	6.06E-03	chr5(-):129881161-129887471	UCSC	coiled-coil-helix-coiled-coil-helix domain containing 2	AK088374	14004	ENSMUSG00000070493	8.8	9.96	Yes
GSMG0010813	Uqcrb	up	2.23	2.48E-03	chr13(-):66900617-66905350	UCSC	ubiquinol-cytochrome c reductase binding protein	NM_026219	67530	ENSMUSG00000021520	8.31	9.47	Yes
GSMG0042320	Plp2	up	2.23	4.42E-03	chrX(-):7667943-7671373	UCSC	proteolipid protein 2	AK012816	18824	ENSMUSG00000031146	10.91	12.06	No
GSMG0035957	Mrgpra3	up	2.23	4.04E-02	chr7(-):47588950-47601372	UCSC	MAS-related GPR, member A3	NM_153067	233222	ENSMUSG00000078698	6.14	7.29	Yes
GSMG0025182	Sirpb1c	up	2.23	2.29E-02	chr3(-):15795147-15848512	UCSC	signal-regulatory protein beta 1C	NM_001173459	100038947	ENSMUSG00000074677	7.62	8.77	Yes
GSMG0023660	Cd93	up	2.23	1.96E-02	chr2(-):148436651-148444026	UCSC	CD93 antigen	AF081789	17064	ENSMUSG00000027435	7.84	9	No
GSMG0012929	Dab2	up	2.22	9.96E-03	chr15(+):6299789-6440712	UCSC	disabled 2, mitogen-responsive phosphoprotein	NM_001008702	13132	ENSMUSG00000022150	6.29	7.44	No
GSMG0012702	---	up	2.22	7.06E-03	chr14(-):78564355-78569193	UCSC	---	AK083897	---	---	6.64	7.8	No
GSMG0010168	Depdc1b	up	2.22	3.02E-02	chr13(+):108316333-108407782	UCSC	DEP domain containing 1B	AK043283	218581	ENSMUSG00000021697	7.58	8.73	No
GSMG0009624	Nqo2	up	2.22	3.67E-02	chr13(+):33964659-33988465	UCSC	NAD(P)H dehydrogenase, quinone 2	NM_020282	18105	ENSMUSG00000046949	7.87	9.02	No
GSMG0030627	Usp46	up	2.22	9.06E-03	chr5(-):73998641-74068431	UCSC	ubiquitin specific peptidase 46	AK145298	69727	ENSMUSG00000054814	7.69	8.84	No
GSMG0017632	A530064D06Rik	up	2.21	3.51E-02	chr17(-):48149126-48167271	UCSC	RIKEN cDNA A530064D06 gene	AK171433	328830	ENSMUSG00000043939	6.92	8.06	No
GSMG0013360	Lgals1	up	2.21	1.17E-02	chr15(+):78926725-78930466	UCSC	lectin, galactose binding, soluble 1	AK004298	16852	ENSMUSG00000068220	9.76	10.9	No
GSMG0010400	Prss16	up	2.21	3.18E-03	chr13(-):22002176-22009770	UCSC	protease, serine, 16 (thymus)	AK088019	54373	ENSMUSG00000006179	7.52	8.67	No
GSMG0010082	F2rl2	up	2.21	2.96E-04	chr13(+):95696853-95702768	UCSC	coagulation factor II (thrombin) receptor-like 2	NM_010170	14064	ENSMUSG00000021675	6.7	7.85	No
GSMG0042077	---	up	2.21	2.94E-03	chrX(+):130009911-130011556	UCSC	---	AK047731	---	---	6.34	7.49	No
GSMG0040904	2300009A05Rik	up	2.21	2.44E-03	chr9(-):63394447-63399244	UCSC	RIKEN cDNA 2300009A05 gene	NM_027090	69478	ENSMUSG00000032403	7.55	8.7	No
GSMG0016148	Tiam2	up	2.2	4.35E-02	chr17(+):3326573-3531344	UCSC	T cell lymphoma invasion and metastasis 2	NM_001122998	24001	ENSMUSG00000023800	6.42	7.56	No
GSMG0014311	---	up	2.2	1.22E-02	chr15(-):82993370-82995706	UCSC	---	AK159337	---	---	7.35	8.48	No
GSMG0010773	Gm3604	up	2.2	4.44E-02	chr13(-):62367716-62383174	UCSC	predicted gene 3604	NM_001162910	100041979	ENSMUSG00000094942	6.41	7.55	Yes
GSMG0007569	Wipi1	up	2.2	2.22E-02	chr11(-):109573332-109611433	UCSC	WD repeat domain, phosphoinositide interacting 1	AK159608	52639	ENSMUSG00000041895	6.93	8.06	No
GSMG0027848	Txn1	up	2.2	2.66E-03	chr4(-):57943373-57956411	UCSC	thioredoxin 1	NM_011660	22166	ENSMUSG00000028367	10.62	11.75	No
GSMG0022688	Stom	up	2.2	1.51E-02	chr2(-):35313986-35337009	UCSC	stomatin	U17297	13830	ENSMUSG00000026880	9.09	10.23	No
GSMG0011367	---	up	2.19	9.88E-03	chr14(+):29903768-29907580	UCSC	---	BC066010	---	---	7.78	8.92	Yes
GSMG0025915	Gstm1	up	2.19	4.41E-02	chr3(-):108012251-108017975	UCSC	glutathione S-transferase, mu 1	NM_010358	14862	ENSMUSG00000058135	8.46	9.59	Yes
GSMG0020337	Cpeb3	up	2.18	3.39E-02	chr19(-):37021291-37207509	UCSC	cytoplasmic polyadenylation element binding protein 3	NM_198300	208922	ENSMUSG00000039652	6.67	7.8	No
GSMG0019669	Cyp2c50	up	2.18	8.02E-03	chr19(+):40089679-40113955	UCSC	cytochrome P450, family 2, subfamily c, polypeptide 50	NM_134144	107141	ENSMUSG00000054827	6.64	7.76	No
GSMG0016324	Hcfc1r1	up	2.18	4.67E-02	chr17(+):23673608-23675524	UCSC	host cell factor C1 regulator 1 (XPO1-dependent)	NM_181821	353502	ENSMUSG00000023904	8.49	9.61	No
GSMG0001399	---	up	2.18	3.21E-02	chr1(-):23391749-23397774	UCSC	---	AK090150	---	---	6.46	7.58	Yes
GSMG0011612	Ltb4r1	up	2.18	4.02E-02	chr14(+):55765960-55768492	UCSC	leukotriene B4 receptor 1	NM_008519	16995	ENSMUSG00000046908	7.5	8.62	No
GSMG0005668	Wfdc17	up	2.18	3.29E-02	chr11(+):83704016-83706269	UCSC	WAP four-disulfide core domain 17	FJ007372	100034251	ENSMUSG00000069792	7.15	8.27	No
GSMG0041619	---	up	2.18	9.72E-03	chrX(+):18226851-18228940	UCSC	---	AK079313	---	---	6.99	8.12	No
GSMG0040947	---	up	2.18	2.58E-03	chr9(-):66804732-66806011	UCSC	---	AK015709	---	---	7.22	8.35	Yes
GSMG0010865	Ndufs6	up	2.17	9.16E-03	chr13(-):73319876-73328482	UCSC	NADH dehydrogenase (ubiquinone) Fe-S protein 6	NR_033306	407785	ENSMUSG00000021606	8.83	9.94	No
GSMG0008984	Dhrs7	up	2.17	3.23E-02	chr12(-):72650354-72664828	UCSC	dehydrogenase/reductase (SDR family) member 7	NM_025522	66375	ENSMUSG00000021094	8.98	10.1	No
GSMG0007696	Timp2	up	2.17	3.99E-02	chr11(-):118301065-118355506	UCSC	tissue inhibitor of metalloproteinase 2	AK081522	21858	ENSMUSG00000017466	8.23	9.35	No
GSMG0038818	Gm10631	up	2.17	1.01E-02	chr8(-):104177780-104184600	UCSC	predicted gene 10631	AK135158	---	ENSMUSG00000074128	6.77	7.89	Yes
GSMG0035856	Siglece	up	2.17	2.37E-02	chr7(-):43651070-43660164	UCSC	sialic acid binding Ig-like lectin E	NM_031181	83382	ENSMUSG00000030474	8.55	9.67	No
GSMG0032189	Rpn1	up	2.17	3.72E-02	chr6(+):88084473-88105304	UCSC	ribophorin I	NM_133933	103963	ENSMUSG00000030062	9.16	10.28	No
GSMG0017235	Ndufb10	up	2.16	3.43E-02	chr17(-):24722063-24724423	UCSC	NADH dehydrogenase (ubiquinone) 1 beta subcomplex, 10	AK151404	68342	ENSMUSG00000040048	6.84	7.95	No
GSMG0036253	---	up	2.16	6.64E-03	chr7(-):90207695-90208839	UCSC	---	AK171074	---	---	7.23	8.34	No
GSMG0051850	Gm6525	up	2.16	2.83E-02	chr3(+):84174638-84175193	UCSC	predicted pseudogene 6525	NR_036654	624713	---	7.85	8.97	Yes
GSMG0043007	Bmx	up	2.15	2.96E-02	chrX(-):164192842-164258194	UCSC	BMX non-receptor tyrosine kinase	NM_009759	12169	ENSMUSG00000031377	6.37	7.47	No
GSMG0028798	Steap4	up	2.15	3.64E-02	chr5(+):7960455-7982213	UCSC	STEAP family member 4	AJ319746	117167	ENSMUSG00000012428	6.67	7.77	No
GSMG0027237	E2f2	up	2.15	2.13E-02	chr4(+):136172392-136196057	UCSC	E2F transcription factor 2	NM_177733	242705	ENSMUSG00000018983	7.68	8.78	No
GSMG0002987	---	up	2.15	1.88E-02	chr10(+):52450368-52452515	UCSC	---	AK076568	---	---	8.43	9.54	Yes
GSMG0019805	Mxi1	up	2.14	2.25E-02	chr19(+):53310506-53375810	UCSC	Max interacting protein 1	NM_001008542	17859	ENSMUSG00000025025	8.57	9.67	No
GSMG0012394	---	up	2.14	1.20E-02	chr14(-):54463044-54464744	UCSC	---	AK144204	---	---	6.05	7.15	Yes
GSMG0010705	Pdlim7	up	2.14	3.40E-03	chr13(-):55495795-55513439	UCSC	PDZ and LIM domain 7	AK033091	67399	ENSMUSG00000021493	7.49	8.59	No
GSMG0007014	Cpd	up	2.14	3.70E-02	chr11(-):76777208-76847008	UCSC	carboxypeptidase D	NM_007754	12874	ENSMUSG00000020841	7.89	8.98	No
GSMG0003313	---	up	2.14	3.07E-02	chr10(+):82064017-82066713	UCSC	---	AK132466	---	---	8.93	10.02	Yes
GSMG0043177	Hist1h4m	up	2.13	6.20E-03	chr13(+):21811746-21812150	UCSC	histone cluster 1, H4m	NM_001195421	100041230	ENSMUSG00000069306	6.85	7.94	No
GSMG0043021	Tmsb4x	up	2.13	1.65E-04	chrX(-):167207094-167209218	UCSC	thymosin, beta 4, X chromosome	NM_021278	19241	ENSMUSG00000049775	11.29	12.38	No
GSMG0041610	Gpr82	up	2.13	2.69E-02	chrX(+):13661306-13667433	UCSC	G protein-coupled receptor 82	AK033761	319200	ENSMUSG00000047678	4.73	5.82	Yes
GSMG0051052	2810417H13Rik	up	2.13	3.76E-02	chr9(+):65890318-65903551	UCSC	RIKEN cDNA 2810417H13 gene	NM_026515	68026	ENSMUSG00000040204	9	10.09	No
GSMG0031858	Gpnmb	up	2.13	1.88E-03	chr6(+):49036518-49070929	UCSC	glycoprotein (transmembrane) nmb	AK079220	93695	ENSMUSG00000029816	5.91	7	Yes
GSMG0013534	Lrrk2	up	2.12	4.83E-02	chr15(+):91673223-91816124	UCSC	leucine-rich repeat kinase 2	NM_025730	66725	ENSMUSG00000036273	8.46	9.55	Yes
GSMG0012174	Spcs1	up	2.12	1.53E-02	chr14(-):30999827-31001666	UCSC	signal peptidase complex subunit 1 homolog (S. cerevisiae)	NM_026911	69019	ENSMUSG00000021917	8.87	9.96	Yes
GSMG0051115	---	up	2.12	1.45E-02	chr1(-):13785685-13786929	UCSC	---	NR_004447	---	---	9.67	10.75	No
GSMG0010584	Adtrp	up	2.12	3.16E-02	chr13(-):41763148-41847617	UCSC	androgen dependent TFPI regulating protein	NM_001145875	109254	ENSMUSG00000058022	6.09	7.17	No
GSMG0006259	Smtn	up	2.12	4.78E-02	chr11(-):3517523-3539294	UCSC	smoothelin	NM_013870	29856	ENSMUSG00000020439	6.53	7.61	No
GSMG0035738	Sdhaf1	up	2.12	1.40E-02	chr7(-):30321411-30322375	UCSC	succinate dehydrogenase complex assembly factor 1	NM_001033140	68332	ENSMUSG00000074211	9.3	10.38	No
GSMG0033917	---	up	2.12	1.43E-02	chr6(-):148915215-148920535	UCSC	---	AK082007	---	---	6.42	7.5	No
GSMG0032709	Shfm1	up	2.12	6.20E-04	chr6(-):6557298-6578658	UCSC	split hand/foot malformation (ectrodactyly) type 1	AK135462	20422	ENSMUSG00000042541	9.44	10.52	No
GSMG0015077	Cd200r4	up	2.11	1.02E-02	chr16(+):44811782-44839150	UCSC	CD200 receptor 4	AK089262	239849	ENSMUSG00000062082	6.02	7.1	No
GSMG0014969	Cox17	up	2.11	3.84E-03	chr16(+):38346999-38352763	UCSC	cytochrome c oxidase assembly protein 17	NM_001017429	12856	ENSMUSG00000046516	8.33	9.4	No
GSMG0041661	Il13ra1	up	2.11	2.75E-02	chrX(+):36112106-36171262	UCSC	interleukin 13 receptor, alpha 1	AK154675	16164	ENSMUSG00000017057	7.72	8.8	No
GSMG0033132	---	up	2.11	2.33E-02	chr6(-):69823352-69823641	UCSC	---	M34528	---	---	11.42	12.5	Yes
GSMG0032030	---	up	2.11	1.66E-02	chr6(+):67896356-67896648	UCSC	---	X53355	---	---	10.37	11.45	Yes
GSMG0024151	Pld1	up	2.11	4.21E-02	chr3(+):27938680-28133362	UCSC	phospholipase D1	NM_001164056	18805	ENSMUSG00000027695	6.56	7.64	No
GSMG0004836	Osm	up	2.1	2.92E-02	chr11(+):4236785-4241026	UCSC	oncostatin M	NM_001013365	18413	ENSMUSG00000058755	6.87	7.94	No
GSMG0041447	Cx3cr1	up	2.1	1.78E-03	chr9(-):120048683-120068297	UCSC	chemokine (C-X3-C) receptor 1	NM_009987	13051	ENSMUSG00000052336	7.17	8.24	No
GSMG0041096	Snx14	up	2.1	3.01E-02	chr9(-):88376747-88438951	UCSC	sorting nexin 14	NM_172926	244962	ENSMUSG00000032422	8.62	9.69	No
GSMG0026500	---	up	2.1	4.46E-03	chr4(+):44831658-44834010	UCSC	---	AK046982	---	---	7.26	8.33	No
GSMG0016682	Tnfrsf21	up	2.09	4.50E-02	chr17(+):43016551-43089188	UCSC	tumor necrosis factor receptor superfamily, member 21	AK043823	94185	ENSMUSG00000023915	8.36	9.42	No
GSMG0013013	---	up	2.09	4.12E-02	chr15(+):22592223-22596093	UCSC	---	AK137113	---	---	5.57	6.64	Yes
GSMG0005751	4930405P13Rik	up	2.09	4.12E-03	chr11(+):88905927-88936061	UCSC	RIKEN cDNA 4930405P13 gene	AK015102	73921	ENSMUSG00000087338	8.13	9.2	Yes
GSMG0041250	Alas1	up	2.09	3.09E-02	chr9(-):106233455-106247954	UCSC	aminolevulinic acid synthase 1	NM_020559	11655	ENSMUSG00000032786	9.12	10.18	No
GSMG0003399	Ikbip	up	2.09	1.74E-02	chr10(+):91083039-91102612	UCSC	IKBKB interacting protein	NM_027078	67454	ENSMUSG00000019975	6.46	7.52	Yes
GSMG0043151	Gm15772	up	2.09	4.29E-02	chr5(+):3236385-3236909	UCSC	predicted gene 15772	X80699	100034726	ENSMUSG00000062353	10.06	11.13	Yes
GSMG0023958	Aurka	up	2.09	1.13E-02	chr2(-):172356191-172370536	UCSC	aurora kinase A	AK145968	20878	ENSMUSG00000027496	8.58	9.64	No
GSMG0019562	C030016D13Rik	up	2.08	4.03E-04	chr19(+):27430037-27432631	UCSC	RIKEN cDNA C030016D13 gene	NR_027987	107372	---	4.6	5.66	Yes
GSMG0040441	Cdkn2d	up	2.08	5.36E-03	chr9(-):21288410-21291209	UCSC	cyclin-dependent kinase inhibitor 2D (p19, inhibits CDK4)	NM_009878	12581	ENSMUSG00000096472	8.48	9.54	No
GSMG0039896	Bcl2a1a	up	2.08	2.87E-02	chr9(+):88956920-88962416	UCSC	B cell leukemia/lymphoma 2 related protein A1a	NM_009742	12044	ENSMUSG00000093809	8.33	9.39	Yes
GSMG0031260	BC037034	up	2.08	2.72E-03	chr5(-):138259657-138264052	UCSC	cDNA sequence BC037034	NM_153161	231807	ENSMUSG00000091964	8.82	9.87	No
GSMG0029142	Ncapg	up	2.08	2.50E-02	chr5(+):45669925-45700547	UCSC	non-SMC condensin I complex, subunit G	NM_019438	54392	ENSMUSG00000015880	8.07	9.12	No
GSMG0008690	---	up	2.07	3.37E-02	chr12(-):8921312-8922287	UCSC	---	AK142786	---	---	7.18	8.24	No
GSMG0038521	---	up	2.07	2.18E-03	chr8(-):69112971-69113719	UCSC	---	AK180407	---	---	6.97	8.01	No
GSMG0021174	Cers6	up	2.06	3.45E-04	chr2(+):69112737-69114280	UCSC	ceramide synthase 6	AK136463	241447	ENSMUSG00000027035	8.4	9.44	Yes
GSMG0001987	Ralb	up	2.06	4.79E-02	chr1(-):119470305-119504782	UCSC	v-ral simian leukemia viral oncogene homolog B (ras related)	NM_022327	64143	ENSMUSG00000004451	8.59	9.63	No
GSMG0015964	---	up	2.06	3.70E-02	chr16(-):75893346-75895898	UCSC	---	AK155543	---	---	8.5	9.54	No
GSMG0010120	Serf1	up	2.06	3.57E-02	chr13(+):100108019-100114231	UCSC	small EDRK-rich factor 1	NM_011353	20365	ENSMUSG00000021643	5.68	6.73	Yes
GSMG0000514	---	up	2.06	2.00E-02	chr1(+):86826100-86829655	UCSC	---	AK142992	---	---	7.1	8.14	Yes
GSMG0036522	Rnf141	up	2.06	8.08E-05	chr7(-):110813532-110844421	UCSC	ring finger protein 141	AK154417	67150	ENSMUSG00000030788	8.68	9.73	No
GSMG0035354	Taldo1	up	2.06	3.15E-02	chr7(+):141392160-141402976	UCSC	transaldolase 1	NM_011528	21351	ENSMUSG00000025503	9.21	10.25	No
GSMG0018355	Ap3s1	up	2.05	2.64E-02	chr18(+):46741904-46790826	UCSC	adaptor-related protein complex 3, sigma 1 subunit	AK137489	11777	ENSMUSG00000024480	10.11	11.14	Yes
GSMG0017283	Bak1	up	2.05	1.52E-02	chr17(-):27019810-27028627	UCSC	BCL2-antagonist/killer 1	Y13231	12018	ENSMUSG00000057789	8.04	9.08	No
GSMG0010284	---	up	2.05	1.91E-02	chr13(-):5860326-5861199	UCSC	---	AK020996	---	---	7.78	8.82	No
GSMG0007337	Top2a	up	2.05	4.04E-02	chr11(-):98937373-99024189	UCSC	topoisomerase (DNA) II alpha	U01919	21973	ENSMUSG00000020914	9.24	10.27	Yes
GSMG0004486	Plxnc1	up	2.05	3.06E-02	chr10(-):94790869-94944578	UCSC	plexin C1	NM_018797	54712	ENSMUSG00000074785	9.54	10.57	No
GSMG0026546	Msantd3	up	2.05	1.21E-02	chr4(+):48539933-48561919	UCSC	Myb/SANT-like DNA-binding domain containing 3	AK075956	66665	ENSMUSG00000039693	6.81	7.84	No
GSMG0022272	Gm14403	up	2.04	2.41E-02	chr2(+):177498226-177512309	UCSC	predicted gene 14403	NR_036450	433520	ENSMUSG00000094786	7.93	8.96	Yes
GSMG0019809	---	up	2.04	8.58E-04	chr19(+):53460621-53462723	UCSC	---	AK014661	---	---	6.73	7.76	No
GSMG0009529	Hist1h4h	up	2.04	1.13E-02	chr13(+):23531044-23531519	UCSC	histone cluster 1, H4h	NM_153173	69386	ENSMUSG00000060981	8.55	9.58	No
GSMG0035847	Gm5595	up	2.04	3.38E-02	chr7(-):42660110-42692742	UCSC	predicted gene 5595	NM_001008427	434179	ENSMUSG00000069727	5.74	6.77	No
GSMG0029921	Myl10	up	2.04	3.04E-02	chr5(+):136693146-136701094	UCSC	myosin, light chain 10, regulatory	NM_021611	59310	ENSMUSG00000005474	6.67	7.7	Yes
GSMG0028259	Atp6v0b	up	2.04	1.24E-04	chr4(-):117884327-117887334	UCSC	ATPase, H+ transporting, lysosomal V0 subunit B	AF356006	114143	ENSMUSG00000033379	10.23	11.26	No
GSMG0018513	Sec11c	up	2.03	1.75E-02	chr18(+):65800548-65817657	UCSC	SEC11 homolog C (S. cerevisiae)	AK003027	66286	ENSMUSG00000024516	10.37	11.39	No
GSMG0038271	Rbpms	up	2.03	3.07E-02	chr8(-):33782644-33929841	UCSC	RNA binding protein gene with multiple splicing	NM_019733	19663	ENSMUSG00000031586	7.61	8.64	No
GSMG0038023	---	up	2.03	3.33E-02	chr8(+):128367916-128369990	UCSC	---	AK080781	---	---	5.14	6.15	Yes
GSMG0037255	Acsl1	up	2.03	3.32E-02	chr8(+):46471042-46536050	UCSC	acyl-CoA synthetase long-chain family member 1	NM_007981	14081	ENSMUSG00000018796	8.59	9.62	No
GSMG0020063	---	up	2.02	2.87E-02	chr19(-):8756368-8757021	UCSC	---	AK043475	---	---	5.64	6.66	Yes
GSMG0017336	Ccdc167	up	2.02	3.97E-02	chr17(-):29695978-29717017	UCSC	coiled-coil domain containing 167	NM_001163741	68597	ENSMUSG00000024018	7.18	8.19	No
GSMG0014131	Ly6c1	up	2.02	1.77E-02	chr15(-):75044027-75048837	UCSC	lymphocyte antigen 6 complex, locus C1	NM_001252056	17067	ENSMUSG00000079018	10.82	11.83	No
GSMG0052730	Hba-a1	up	2.02	4.98E-02	chr11(+):32283671-32284489	UCSC	hemoglobin alpha, adult chain 1	AK011018	15122	ENSMUSG00000069919	11.69	12.71	Yes
GSMG0004499	Nudt4	up	2.02	1.03E-02	chr10(-):95547007-95564167	UCSC	nudix (nucleoside diphosphate linked moiety X)-type motif 4	NM_027722	71207	ENSMUSG00000020029	10.03	11.05	No
GSMG0037518	Ndufb7	up	2.02	1.39E-02	chr8(+):83566756-83571623	UCSC	NADH dehydrogenase (ubiquinone) 1 beta subcomplex, 7	AK003300	66916	ENSMUSG00000033938	8.76	9.77	No
GSMG0035228	---	up	2.02	7.62E-03	chr7(+):128644435-128646953	UCSC	---	AK083879	---	---	6.24	7.25	Yes
GSMG0030307	Tyms	up	2.02	1.16E-02	chr5(-):30058200-30073625	UCSC	thymidylate synthase	NR_033402	22171	ENSMUSG00000025747	7.78	8.79	Yes
GSMG0023897	Sulf2	up	2.02	2.30E-02	chr2(-):166073089-166155683	UCSC	sulfatase 2	AK133336	72043	ENSMUSG00000006800	7.19	8.21	No
GSMG0021967	Srxn1	up	2.01	2.20E-02	chr2(+):152105524-152111375	UCSC	sulfiredoxin 1 homolog (S. cerevisiae)	NM_029688	76650	ENSMUSG00000032802	7.4	8.41	Yes
GSMG0021316	Prg3	up	2.01	1.72E-02	chr2(+):84988195-84993886	UCSC	proteoglycan 3	AK158110	53856	ENSMUSG00000027072	6.63	7.63	No
GSMG0019601	Pten	up	2.01	4.14E-03	chr19(+):32757497-32826159	UCSC	phosphatase and tensin homolog	NM_008960	19211	ENSMUSG00000013663	10.06	11.07	Yes
GSMG0016804	Ebi3	up	2.01	3.43E-02	chr17(+):55952623-55957024	UCSC	Epstein-Barr virus induced gene 3	NM_015766	50498	ENSMUSG00000003206	7.96	8.97	No
GSMG0012052	Psmd6	up	2.01	5.60E-03	chr14(-):14112185-14120940	UCSC	proteasome (prosome, macropain) 26S subunit, non-ATPase, 6	AJ252147	66413	ENSMUSG00000021737	9.51	10.52	No
GSMG0000997	Mpc2	up	2.01	4.26E-03	chr1(+):165461201-165481214	UCSC	mitochondrial pyruvate carrier 2	AK011889	70456	ENSMUSG00000026568	8.78	9.79	No
GSMG0036414	Trim30b	up	2.01	5.82E-03	chr7(-):104355380-104369885	UCSC	tripartite motif-containing 30B	AK080092	244183	ENSMUSG00000052749	5.84	6.85	No
GSMG0035805	Nudt19	up	2.01	1.99E-02	chr7(-):35547185-35555928	UCSC	nudix (nucleoside diphosphate linked moiety X)-type motif 19	NM_033080	110959	ENSMUSG00000034875	7.67	8.67	No
GSMG0023455	Gatm	up	2.01	2.91E-02	chr2(-):122594473-122611278	UCSC	glycine amidinotransferase (L-arginine:glycine amidinotransferase)	AK146111	67092	ENSMUSG00000027199	8.33	9.35	No
GSMG0019914	Ndufs8	up	2	3.16E-03	chr19(-):3908864-3912775	UCSC	NADH dehydrogenase (ubiquinone) Fe-S protein 8	NM_144870	225887	ENSMUSG00000059734	9.05	10.05	Yes
GSMG0042792	Cysltr1	up	2	2.13E-02	chrX(-):106576517-106603600	UCSC	cysteinyl leukotriene receptor 1	AK033476	58861	ENSMUSG00000052821	5.81	6.81	No
GSMG0022294	Rps21	up	2	8.26E-03	chr2(+):180257378-180258444	UCSC	ribosomal protein S21	NM_025587	66481	ENSMUSG00000039001	10.21	11.21	No
GSMG0021358	Olfr1188	up	1.99	1.18E-02	chr2(+):88559451-88560418	UCSC	olfactory receptor 1188	NM_146919	258921	ENSMUSG00000068809	4.74	5.73	Yes
GSMG0013695	Prr13	up	1.99	1.14E-03	chr15(+):102459170-102462809	UCSC	proline rich 13	NM_025385	66151	ENSMUSG00000023048	10.25	11.24	No
GSMG0051357	Tmem170b	up	1.99	4.08E-02	chr13(+):41606216-41641357	UCSC	transmembrane protein 170B	NM_001163572	621976	ENSMUSG00000087370	7.79	8.78	Yes
GSMG0038228	---	up	1.99	6.04E-03	chr8(-):26062515-26066052	UCSC	---	AK137862	---	---	6.67	7.66	Yes
GSMG0033679	Ncapd2	up	1.99	4.07E-02	chr6(-):125168009-125191616	UCSC	non-SMC condensin I complex, subunit D2	AK156143	68298	ENSMUSG00000038252	8.89	9.88	Yes
GSMG0029681	Pxn	up	1.99	2.02E-02	chr5(+):115506687-115555986	UCSC	paxillin	NM_133915	19303	ENSMUSG00000029528	9.72	10.72	No
GSMG0003225	Tpgs1	up	1.99	1.27E-02	chr10(+):79669369-79676126	UCSC	tubulin polyglutamylase complex subunit 1	NM_148934	110012	ENSMUSG00000020308	7.8	8.79	Yes
GSMG0020031	Nudt22	up	1.98	1.82E-02	chr19(-):6993019-6996038	UCSC	nudix (nucleoside diphosphate linked moiety X)-type motif 22	AK002252	68323	ENSMUSG00000037349	7.78	8.76	No
GSMG0017306	Fkbp5	up	1.98	2.12E-02	chr17(-):28398753-28517524	UCSC	FK506 binding protein 5	AK159389	14229	ENSMUSG00000024222	8.54	9.52	No
GSMG0040122	Cdc25a	up	1.98	1.92E-02	chr9(+):109875577-109893888	UCSC	cell division cycle 25A	NM_007658	12530	ENSMUSG00000032477	8.84	9.82	Yes
GSMG0002685	Syne1	up	1.98	4.15E-02	chr10(-):5020201-5194707	UCSC	spectrin repeat containing, nuclear envelope 1	NM_001079686	64009	ENSMUSG00000019769	9.38	10.36	No
GSMG0027930	---	up	1.98	4.89E-02	chr4(-):69922521-69926244	UCSC	---	AK030082	---	---	9.6	10.59	Yes
GSMG0023978	Atp5e	up	1.98	9.88E-03	chr2(-):174461075-174464101	UCSC	ATP synthase, H+ transporting, mitochondrial F1 complex, epsilon subunit	NM_025983	67126	ENSMUSG00000016252	8.25	9.23	No
GSMG0016961	Gemin6	up	1.97	2.50E-02	chr17(+):80224449-80228497	UCSC	gem (nuclear organelle) associated protein 6	AK167920	67242	ENSMUSG00000055760	6.58	7.56	Yes
GSMG0004451	---	up	1.97	4.79E-04	chr10(-):89743617-89745821	UCSC	---	AK087890	---	---	6.64	7.62	No
GSMG0038180	Ckap2	up	1.97	2.73E-02	chr8(-):22168152-22185819	UCSC	cytoskeleton associated protein 2	NM_001004140	80986	ENSMUSG00000037725	7.04	8.01	No
GSMG0037790	Zfhx3	up	1.97	1.57E-02	chr8(+):108714644-108961636	UCSC	zinc finger homeobox 3	NM_007496	11906	ENSMUSG00000038872	7.27	8.24	No
GSMG0033117	---	up	1.97	1.12E-02	chr6(-):68768550-68768846	UCSC	---	X59097	---	---	11.77	12.75	No
GSMG0028600	Efhd2	up	1.97	6.16E-03	chr4(-):141858142-141874952	UCSC	EF hand domain containing 2	AK048475	27984	ENSMUSG00000040659	10.12	11.1	No
GSMG0028343	Cdca8	up	1.97	4.06E-02	chr4(-):124918465-124936917	UCSC	cell division cycle associated 8	NM_026560	52276	ENSMUSG00000028873	7.93	8.9	No

FAST DB STABLE ID	Gene Symbol	Regulation	Fold-Change	P-Value	Gene Coordinates (mm10)	UCSC Link	Gene Name	Representative Transcript ID	Entrez Gene ID	EnsEMBL ID	log^2 intensity of untreated	log^2 intensity of treated	Low-specificity probe included
GSMG0020118	Ms4a6d	up	1.96	3.57E-02	chr19(-):11586605-11604804	UCSC	membrane-spanning 4-domains, subfamily A, member 6D	NM_026835	68774	ENSMUSG00000024679	7.7	8.67	No
GSMG0020021	Prdx5	up	1.96	2.85E-02	chr19(-):6906698-6910077	UCSC	peroxiredoxin 5	AK009858	54683	ENSMUSG00000024953	10.25	11.23	Yes
GSMG0019311	Rnaseh2c	up	1.96	6.01E-04	chr19(+):5601873-5602959	UCSC	ribonuclease H2, subunit C	NM_026616	68209	ENSMUSG00000024925	8.05	9.02	Yes
GSMG0006356	Sec61g	up	1.96	1.53E-02	chr11(-):16501630-16508484	UCSC	SEC61, gamma subunit	NM_011343	20335	ENSMUSG00000078974	8.51	9.48	Yes
GSMG0035172	Mapk3	up	1.96	2.34E-02	chr7(+):126759626-126765816	UCSC	mitogen-activated protein kinase 3	NM_011952	26417	ENSMUSG00000063065	8.11	9.08	No
GSMG0034447	Pnkp	up	1.96	1.47E-02	chr7(+):44857150-44862929	UCSC	polynucleotide kinase 3'- phosphatase	NM_021549	59047	ENSMUSG00000002963	8.59	9.56	No
GSMG0003174	---	up	1.96	3.10E-03	chr10(+):76078617-76080502	UCSC	---	AK139596	---	---	9.68	10.65	Yes
GSMG0018137	Mocos	up	1.95	1.31E-02	chr18(+):24653691-24701573	UCSC	molybdenum cofactor sulfurase	NM_026779	68591	ENSMUSG00000039616	6.87	7.84	No
GSMG0012868	---	up	1.95	4.57E-02	chr14(-):120429206-120429630	UCSC	---	AK180994	---	---	6.27	7.23	No
GSMG0008046	G2e3	up	1.95	1.80E-03	chr12(+):51348230-51376986	UCSC	G2/M-phase specific E3 ubiquitin ligase	NM_001167964	217558	ENSMUSG00000035293	7.94	8.9	No
GSMG0007760	---	up	1.95	6.72E-03	chr11(-):120984407-120987804	UCSC	---	AK141798	---	---	7.38	8.34	No
GSMG0004994	Psme4	up	1.95	4.30E-03	chr11(+):30771775-30880353	UCSC	proteasome (prosome, macropain) activator subunit 4	NM_134013	103554	ENSMUSG00000040850	9.22	10.19	No
GSMG0041710	Stag2	up	1.95	3.81E-02	chrX(+):42149412-42277179	UCSC	stromal antigen 2	NM_001077712	20843	ENSMUSG00000025862	9	9.96	Yes
GSMG0041695	Mcts1	up	1.95	3.78E-02	chrX(+):38600654-38613522	UCSC	malignant T cell amplified sequence 1	AK005292	68995	ENSMUSG00000000355	7.51	8.47	Yes
GSMG0039434	---	up	1.95	3.48E-02	chr9(+):44336284-44338280	UCSC	---	AK079124	---	---	7.11	8.08	No
GSMG0043360	---	up	1.95	7.74E-03	chr7(-):27470167-27470343	UCSC	---	AK192398	---	---	8.49	9.45	Yes
GSMG0029446	Cxcl13	up	1.95	8.79E-04	chr5(+):95956924-95961068	UCSC	chemokine (C-X-C motif) ligand 13	AK160241	55985	ENSMUSG00000023078	6.09	7.05	Yes
GSMG0025653	Zbtb7b	up	1.95	2.60E-02	chr3(-):89377647-89393203	UCSC	zinc finger and BTB domain containing 7B	NM_009565	22724	ENSMUSG00000028042	8.55	9.52	No
GSMG0025206	Agtr1b	up	1.95	1.98E-03	chr3(-):20314473-20367177	UCSC	angiotensin II receptor, type 1b	NM_175086	11608	ENSMUSG00000054988	6.01	6.97	Yes
GSMG0024272	---	up	1.95	4.68E-02	chr3(+):50001971-50010783	UCSC	---	AK144912	---	---	7.47	8.44	No
GSMG0022099	Top1	up	1.94	1.03E-02	chr2(+):160645887-160722761	UCSC	topoisomerase (DNA) I	NM_009408	21969	ENSMUSG00000070544	9.27	10.22	Yes
GSMG0018166	---	up	1.94	1.11E-02	chr18(+):31859076-31861764	UCSC	---	AK143384	---	---	6.35	7.31	No
GSMG0015989	---	up	1.94	2.14E-02	chr16(-):84949685-84954038	UCSC	---	AK132225	---	---	6.06	7.01	Yes
GSMG0001398	Ogfrl1	up	1.94	4.62E-03	chr1(-):23366424-23383175	UCSC	opioid growth factor receptor-like 1	NM_001081079	70155	ENSMUSG00000026158	8.16	9.11	No
GSMG0004768	Myl6	up	1.94	1.78E-04	chr10(-):128490860-128493876	UCSC	myosin, light polypeptide 6, alkali, smooth muscle and non-muscle	AK168450	17904	ENSMUSG00000090841	11.12	12.08	No
GSMG0052421	Cmc2	up	1.94	6.50E-03	chr8(-):116888685-116911245	UCSC	COX assembly mitochondrial protein 2	NM_026844	66531	ENSMUSG00000014633	9.04	10	No
GSMG0043340	---	up	1.94	1.05E-02	chr5(-):5781530-5781642	UCSC	---	X05838	---	---	10.34	11.29	Yes
GSMG0020040	Cox8a	up	1.93	4.08E-03	chr19(-):7215158-7217616	UCSC	cytochrome c oxidase subunit VIIIa	NM_007750	12868	ENSMUSG00000035885	9.8	10.75	Yes
GSMG0017615	---	up	1.93	1.77E-02	chr17(-):46852284-46853896	UCSC	---	AK161970	---	---	8.27	9.22	Yes
GSMG0015080	Ccdc80	up	1.93	4.51E-02	chr16(+):45093672-45128077	UCSC	coiled-coil domain containing 80	AK144183	67896	ENSMUSG00000022665	5.75	6.7	Yes
GSMG0010639	1110007C09Rik	up	1.93	1.89E-02	chr13(-):49202951-49216027	UCSC	RIKEN cDNA 1110007C09 gene	AK003526	68480	ENSMUSG00000037960	9.1	10.05	Yes
GSMG0008155	Daam1	up	1.93	6.72E-03	chr12(+):71831078-71992367	UCSC	dishevelled associated activator of morphogenesis 1	NM_172464	208846	ENSMUSG00000034574	7.01	7.96	No
GSMG0005818	Snf8	up	1.93	1.38E-02	chr11(+):96034914-96047429	UCSC	SNF8, ESCRT-II complex subunit, homolog (S. cerevisiae)	AK013058	27681	ENSMUSG00000006058	7.45	8.4	No
GSMG0042423	Ndufa1	up	1.93	1.24E-02	chrX(-):37187591-37191238	UCSC	NADH dehydrogenase (ubiquinone) 1 alpha subcomplex, 1	NM_019443	54405	ENSMUSG00000016427	8.74	9.69	No
GSMG0035928	Emp3	up	1.93	2.00E-04	chr7(-):45918023-45921426	UCSC	epithelial membrane protein 3	NM_010129	13732	ENSMUSG00000040212	10.1	11.05	No
GSMG0029686	Cit	up	1.93	2.89E-02	chr5(+):115845277-116006343	UCSC	citron	AF086824	12704	ENSMUSG00000029516	7.5	8.45	No
GSMG0052731	Hba-a2	up	1.92	3.05E-02	chr11(+):32296488-32297309	UCSC	hemoglobin alpha, adult chain 2	AK010422	110257	ENSMUSG00000069917	11.69	12.63	Yes
GSMG0040112	Pfkfb4	up	1.92	3.74E-03	chr9(+):108991902-109032197	UCSC	6-phosphofructo-2-kinase/fructose-2,6-biphosphatase 4	NM_173019	270198	ENSMUSG00000025648	8.16	9.1	No
GSMG0038431	Neil3	up	1.92	3.85E-02	chr8(-):53586867-53639065	UCSC	nei like 3 (E. coli)	NM_146208	234258	ENSMUSG00000039396	7.41	8.35	No
GSMG0031847	Gimap7	up	1.92	4.23E-02	chr6(+):48718621-48724634	UCSC	GTPase, IMAP family member 7	NM_146167	231932	ENSMUSG00000043931	8.09	9.03	Yes
GSMG0021719	Dut	up	1.91	2.10E-02	chr2(+):125247240-125259049	UCSC	deoxyuridine triphosphatase	NM_001159646	110074	ENSMUSG00000027203	8.3	9.23	No
GSMG0002635	Nenf	up	1.91	2.68E-03	chr1(-):191306790-191318119	UCSC	neuron derived neurotrophic factor	AK155319	66208	ENSMUSG00000037499	7.15	8.09	Yes
GSMG0009972	---	up	1.91	2.17E-02	chr13(+):78153634-78157137	UCSC	---	AK040215	---	---	5.75	6.68	No
GSMG0009203	Efcab11	up	1.91	1.76E-02	chr12(-):99693047-99883442	UCSC	EF-hand calcium binding domain 11	AK011836	78767	ENSMUSG00000021176	6.07	7	Yes
GSMG0005037	---	up	1.91	1.34E-02	chr11(+):35320128-35325045	UCSC	---	AK132357	---	---	6.79	7.72	Yes
GSMG0042406	Gm5935	up	1.91	1.86E-02	chrX(-):24753163-24775164	UCSC	predicted gene 5935	NM_001081657	546282	ENSMUSG00000093923	7.66	8.6	Yes
GSMG0037426	Pgls // Fam129c	up	1.91	1.17E-02	chr8(+):71592182-71608149	UCSC	6-phosphogluconolactonase // family with sequence similarity 129, member C	NM_001166213	100037278 // 66171	USG00000031807 // ENSMUSG00000	7.84	8.77	No
GSMG0037145	Eif4ebp1	up	1.91	2.06E-02	chr8(+):27260327-27276668	UCSC	eukaryotic translation initiation factor 4E binding protein 1	AK013033	13685	ENSMUSG00000031490	7.55	8.48	Yes
GSMG0004052	Prdm1	up	1.91	6.38E-03	chr10(-):44437175-44528502	UCSC	PR domain containing 1, with ZNF domain	AK077622	12142	ENSMUSG00000038151	8.48	9.41	No
GSMG0033478	Rybp	up	1.91	3.50E-02	chr6(-):100228565-100287441	UCSC	RING1 and YY1 binding protein	BC080287	56353	ENSMUSG00000072872	8.16	9.09	No
GSMG0032638	Lyrm5	up	1.91	1.93E-02	chr6(+):145211134-145216939	UCSC	LYR motif containing 5	AF412298	67636	ENSMUSG00000040370	7.31	8.24	Yes
GSMG0025523	Golim4	up	1.91	2.45E-02	chr3(-):75876183-75956949	UCSC	golgi integral membrane protein 4	AK078928	73124	ENSMUSG00000034109	8.18	9.12	No
GSMG0022040	0610038P03Rik	up	1.9	1.34E-02	chr2(+):155592318-155594955	UCSC	RIKEN cDNA 0610038P03 gene	AK002817	---	ENSMUSG00000085010	7.53	8.46	Yes
GSMG0011893	---	up	1.9	4.02E-03	chr14(+):103346585-103360478	UCSC	---	AK007026	---	---	7.15	8.07	Yes
GSMG0009506	Hist1h4j	up	1.9	2.42E-02	chr13(+):21735034-21735837	UCSC	histone cluster 1, H4j	AK010085	319159	ENSMUSG00000067455	10.35	11.27	Yes
GSMG0040949	Rab8b	up	1.9	1.80E-04	chr9(-):66843664-66919705	UCSC	RAB8B, member RAS oncogene family	NM_173413	235442	ENSMUSG00000036943	10.28	11.2	Yes
GSMG0040551	Tirap	up	1.9	1.75E-02	chr9(-):35184391-35200293	UCSC	toll-interleukin 1 receptor (TIR) domain-containing adaptor protein	NM_001177845	117149	ENSMUSG00000032041	7.57	8.49	No
GSMG0028192	Cdkn2c	up	1.9	1.82E-02	chr4(-):109660183-109666658	UCSC	cyclin-dependent kinase inhibitor 2C (p18, inhibits CDK4)	AK157209	12580	ENSMUSG00000028551	8.17	9.1	No
GSMG0027069	Lsm10	up	1.9	1.28E-03	chr4(+):126096653-126098583	UCSC	U7 snRNP-specific Sm-like protein LSM10	NM_138721	116748	ENSMUSG00000050188	7.07	8	No
GSMG0024776	Rhoc	up	1.9	6.70E-03	chr3(+):104789033-104794459	UCSC	ras homolog gene family, member C	AK011599	11853	ENSMUSG00000002233	6.61	7.53	No
GSMG0012724	Diap3	up	1.89	3.30E-02	chr14(-):86655362-87141237	UCSC	diaphanous homolog 3 (Drosophila)	BC085191	56419	ENSMUSG00000022021	7.8	8.72	No
GSMG0011120	Gapt	up	1.89	4.08E-02	chr13(-):110352615-110357178	UCSC	Grb2-binding adaptor, transmembrane	AK155882	238875	ENSMUSG00000046006	9.43	10.35	No
GSMG0008453	Rin3	up	1.89	2.89E-02	chr12(+):102283047-102390854	UCSC	Ras and Rab interactor 3	NM_177620	217835	ENSMUSG00000044456	8.07	8.99	No
GSMG0008202	---	up	1.89	2.99E-02	chr12(+):75630289-75637748	UCSC	---	AK044480	---	---	8.55	9.47	No
GSMG0038554	Uba52	up	1.89	2.14E-03	chr8(-):70508265-70510367	UCSC	ubiquitin A-52 residue ribosomal protein fusion product 1	BC086924	22186	ENSMUSG00000090137	11.21	12.13	No
GSMG0043486	4933413G19Rik	up	1.89	1.61E-02	chr6(+):128375455-128385144	UCSC	RIKEN cDNA 4933413G19 gene	AK161342	71149	ENSMUSG00000079304	7.73	8.64	Yes
GSMG0030404	Msx1	up	1.89	2.68E-02	chr5(-):37820491-37824585	UCSC	msh homeobox 1	NM_010835	17701	ENSMUSG00000048450	7.3	8.22	Yes
GSMG0043844	6530402F18Rik	up	1.89	1.32E-03	chr2(-):29243238-29252993	UCSC	RIKEN cDNA 6530402F18 gene	AK220426	76220	ENSMUSG00000079499	8.46	9.38	No
GSMG0021630	Chp1	up	1.88	6.17E-04	chr2(+):119547703-119587020	UCSC	calcineurin-like EF hand protein 1	NM_019769	56398	ENSMUSG00000014077	9.79	10.7	No
GSMG0018894	---	up	1.88	2.17E-02	chr18(-):39468446-39469661	UCSC	---	AK137987	---	---	7.08	7.99	Yes
GSMG0010367	Stard3nl	up	1.88	4.42E-02	chr13(-):19357677-19395796	UCSC	STARD3 N-terminal like	AK152116	76205	ENSMUSG00000003062	6.52	7.44	Yes
GSMG0009765	Cks2	up	1.88	2.89E-02	chr13(+):51645232-51650720	UCSC	CDC28 protein kinase regulatory subunit 2	NM_025415	66197	ENSMUSG00000062248	7.75	8.67	Yes
GSMG0038034	Fcor	up	1.88	4.55E-02	chr8(-):3671188-3672325	UCSC	Foxo1 corepressor	AK019114	100503924	ENSMUSG00000089665	6.42	7.33	Yes
GSMG0033774	Ybx3	up	1.88	2.99E-02	chr6(-):131364855-131388456	UCSC	Y box protein 3	BC027785	56449	ENSMUSG00000030189	9	9.91	Yes
GSMG0031727	Ndufb2	up	1.88	4.10E-02	chr6(+):39592574-39603382	UCSC	NADH dehydrogenase (ubiquinone) 1 beta subcomplex, 2	AK028334	68198	ENSMUSG00000002416	5.96	6.87	Yes
GSMG0031038	Aldh2	up	1.88	8.46E-03	chr5(-):121566028-121593825	UCSC	aldehyde dehydrogenase 2, mitochondrial	AK163452	11669	ENSMUSG00000029455	10.37	11.27	No
GSMG0027184	Cd164l2	up	1.88	1.08E-02	chr4(+):133212373-133224554	UCSC	CD164 sialomucin-like 2	AK158529	69655	ENSMUSG00000028865	7.11	8.03	Yes
GSMG0018212	Kif20a	up	1.87	4.50E-02	chr18(+):34624613-34633277	UCSC	kinesin family member 20A	NM_001166407	19348	ENSMUSG00000003779	8.06	8.97	Yes
GSMG0015548	Camk2n2	up	1.87	4.44E-03	chr16(-):20619215-20621278	UCSC	calcium/calmodulin-dependent protein kinase II inhibitor 2	NM_028420	73047	ENSMUSG00000051146	7.55	8.45	Yes
GSMG0051443	Ly6f	up	1.87	1.98E-02	chr15(+):75268421-75272234	UCSC	lymphocyte antigen 6 complex, locus F	NM_008530	17071	ENSMUSG00000022583	7.25	8.15	No
GSMG0011028	Naip2	up	1.87	2.66E-03	chr13(-):100144063-100202092	UCSC	NLR family, apoptosis inhibitory protein 2	NM_010872	17948	ENSMUSG00000078945	7.78	8.68	No
GSMG0001070	Tagln2	up	1.87	2.64E-02	chr1(+):172500047-172507379	UCSC	transgelin 2	BC049861	21346	ENSMUSG00000026547	9.33	10.23	Yes
GSMG0028325	Akirin1	up	1.87	1.94E-03	chr4(-):123735196-123750345	UCSC	akirin 1	NM_023423	68050	ENSMUSG00000023075	9.46	10.36	No
GSMG0027554	Ints8	up	1.87	3.80E-02	chr4(-):11199158-11254259	UCSC	integrator complex subunit 8	NM_178112	72656	ENSMUSG00000040738	7.38	8.29	Yes
GSMG0043255	---	up	1.87	2.90E-02	chr16(-):46152958-46153381	UCSC	---	AK219959	---	---	8.91	9.82	Yes
GSMG0022631	---	up	1.87	1.26E-03	chr2(-):31886932-31892418	UCSC	---	AK005985	---	---	7.92	8.83	No
GSMG0019922	BC021614	up	1.86	8.94E-03	chr19(-):4057478-4059444	UCSC	cDNA sequence BC021614	BC034269	225884	ENSMUSG00000058216	7.43	8.32	No
GSMG0019915	Aldh3b1	up	1.86	5.52E-03	chr19(-):3913493-3929810	UCSC	aldehyde dehydrogenase 3 family, member B1	AK154500	67689	ENSMUSG00000024885	7.81	8.7	No
GSMG0019249	---	up	1.86	9.36E-03	chr19(+):3371925-3374092	UCSC	---	AK143406	---	---	7.84	8.74	Yes
GSMG0010072	Tbca	up	1.86	1.72E-02	chr13(+):94788919-94842922	UCSC	tubulin cofactor A	BC051475	21371	ENSMUSG00000042043	8.81	9.7	No
GSMG0052774	-54 // Ighv10-3 // Ighv14-2 //	up	1.86	3.71E-02	chr12(-):112896981-116000321	UCSC	immunoglobulin heavy constant gamma 1 (G1m marker) // immunoglobulin he	AK007826	609 // 544896 // 6199	4546 // ENSMUSG00000094787 // ENS	11.04	11.93	Yes
GSMG0006414	Commd1 // B3gnt2	up	1.86	1.49E-02	chr11(-):22834739-22982284	UCSC	COMM domain containing 1 // UDP-GlcNAc:betaGal beta-1,3-N-acetylglucos	BC098462	17846 // 53625	USG00000051355 // ENSMUSG00000	7.34	8.23	No
GSMG0035631	Atp1a3	up	1.86	3.49E-02	chr7(-):24978171-25005937	UCSC	ATPase, Na+/K+ transporting, alpha 3 polypeptide	NM_144921	232975	ENSMUSG00000040907	7.56	8.45	No
GSMG0032070	Capg	up	1.86	5.68E-03	chr6(+):72544391-72562983	UCSC	capping protein (actin filament), gelsolin-like	NM_007599	12332	ENSMUSG00000056737	10.07	10.97	No
GSMG0029862	Vkorc1l1	up	1.86	2.98E-02	chr5(+):129942109-129984745	UCSC	vitamin K epoxide reductase complex, subunit 1-like 1	BC085133	69568	ENSMUSG00000066735	8.72	9.62	No
GSMG0022693	---	up	1.86	2.36E-02	chr2(-):35455483-35457933	UCSC	---	AK037474	---	---	8.21	9.1	No
GSMG0016487	Ndufv3	up	1.85	4.34E-02	chr17(+):31520115-31531323	UCSC	NADH dehydrogenase (ubiquinone) flavoprotein 3	NM_030087	78330	ENSMUSG00000024038	7.66	8.54	No
GSMG0012714	Wbp4	up	1.85	1.67E-02	chr14(-):79459937-79481520	UCSC	WW domain binding protein 4	NM_018765	22380	ENSMUSG00000022023	7.92	8.81	No
GSMG0011799	Esd	up	1.85	7.30E-03	chr14(+):74732289-74750765	UCSC	esterase D/formylglutathione hydrolase	AK163074	13885	ENSMUSG00000021996	8.72	9.6	No
GSMG0011569	Mrpl52	up	1.85	4.29E-02	chr14(+):54426908-54429753	UCSC	mitochondrial ribosomal protein L52	AK150806	68836	ENSMUSG00000010406	8.83	9.72	No
GSMG0008007	Gm7008	up	1.85	2.56E-03	chr12(+):40222757-40229183	UCSC	predicted gene 7008	NR_045157	629820	ENSMUSG00000035983	8.41	9.3	Yes
GSMG0042520	Mospd1	up	1.85	2.52E-03	chrX(-):53344600-53370503	UCSC	motile sperm domain containing 1	BC085100	70380	ENSMUSG00000023074	7.6	8.48	Yes
GSMG0042367	Cask	up	1.85	1.46E-02	chrX(-):13517080-13846557	UCSC	calcium/calmodulin-dependent serine protein kinase (MAGUK family)	NM_009806	12361	ENSMUSG00000031012	7.35	8.24	No
GSMG0037953	Rpl13	up	1.85	3.96E-03	chr8(+):123102350-123105248	UCSC	ribosomal protein L13	NM_016738	270106	ENSMUSG00000000740	11.52	12.4	Yes
GSMG0035883	Bcl2l12	up	1.85	1.65E-02	chr7(-):44991223-44998712	UCSC	BCL2-like 12 (proline rich)	BC039807	75736	ENSMUSG00000003190	8.02	8.9	No
GSMG0034600	Igf1r	up	1.85	4.84E-02	chr7(+):67952257-68233667	UCSC	insulin-like growth factor I receptor	NM_010513	16001	ENSMUSG00000005533	8.29	9.18	No
GSMG0006559	Pttg1	up	1.84	1.33E-02	chr11(-):43420244-43426248	UCSC	pituitary tumor-transforming gene 1	NM_001131054	30939	ENSMUSG00000020415	8.42	9.3	Yes
GSMG0006249	---	up	1.84	1.60E-02	chr11(-):3233986-3234721	UCSC	---	AK020879	---	---	6.93	7.81	No
GSMG0041459	Higd1a	up	1.84	1.17E-02	chr9(-):121848560-121858000	UCSC	HIG1 domain family, member 1A	NM_019814	56295	ENSMUSG00000038412	7.11	7.99	No
GSMG0043330	Gm8203	up	1.84	2.56E-03	chr6(+):116174096-116174527	UCSC	predicted pseudogene 8203	AK210116	666634	ENSMUSG00000060214	11.16	12.04	Yes
GSMG0027067	Mrps15	up	1.84	1.77E-04	chr4(+):126046924-126055537	UCSC	mitochondrial ribosomal protein S15	AK050396	66407	ENSMUSG00000028861	9.77	10.65	No
GSMG0026541	Sec61b	up	1.84	3.88E-02	chr4(+):47474656-47483239	UCSC	Sec61 beta subunit	AK004505	66212	ENSMUSG00000053317	9.19	10.07	No
GSMG0024702	Fmo5	up	1.84	2.72E-03	chr3(+):97628803-97655287	UCSC	flavin containing monooxygenase 5	NM_001161763	14263	ENSMUSG00000028088	8.03	8.91	No
GSMG0023008	Prkra	up	1.84	3.09E-02	chr2(-):76629937-76648020	UCSC	protein kinase, interferon inducible double stranded RNA dependent activator	AK155112	23992	ENSMUSG00000002731	6.03	6.92	Yes
GSMG0022373	---	up	1.84	1.33E-02	chr2(-):6711739-6713806	UCSC	---	AK081424	---	---	7.62	8.5	No
GSMG0021913	---	up	1.83	2.43E-02	chr2(+):145865245-145870127	UCSC	---	AK047536	---	---	6.35	7.23	Yes
GSMG0047294	---	up	1.83	4.97E-02	chr2(+):125088329-125091794	UCSC	---	AK148201	---	---	6.09	6.96	No
GSMG0021594	---	up	1.83	1.62E-02	chr2(+):117124966-117126843	UCSC	---	AK044288	---	---	6.81	7.68	No
GSMG0021165	Cers6	up	1.83	1.97E-02	chr2(+):68861456-69111290	UCSC	ceramide synthase 6	NM_172856	241447	ENSMUSG00000027035	9.02	9.89	No
GSMG0041617	---	up	1.83	1.60E-02	chrX(+):18192031-18195802	UCSC	---	AK085079	---	---	6.65	7.52	No
GSMG0004601	Glipr1	up	1.83	8.12E-03	chr10(-):111985448-111997264	UCSC	GLI pathogenesis-related 1 (glioma)	NM_028608	73690	ENSMUSG00000056888	9.19	10.06	No
GSMG0036599	Pdzd9	up	1.83	4.26E-02	chr7(-):120658731-120670344	UCSC	PDZ domain containing 9	AK017019	67983	ENSMUSG00000030887	5.7	6.57	Yes
GSMG0034703	Pde8a	up	1.83	9.90E-03	chr7(+):81213593-81334531	UCSC	phosphodiesterase 8A	AK148238	18584	ENSMUSG00000025584	8.67	9.54	No
GSMG0034165	Rps19	up	1.83	7.70E-03	chr7(+):24884630-24889804	UCSC	ribosomal protein S19	AK012052	20085	ENSMUSG00000040952	10.39	11.26	No
GSMG0003528	Gm5176	up	1.83	9.76E-04	chr10(+):111500739-111503850	UCSC	predicted gene 5176	AK048998	382421	---	6.24	7.11	No
GSMG0025746	Ecm1	up	1.83	5.56E-03	chr3(-):95734148-95739571	UCSC	extracellular matrix protein 1	NM_007899	13601	ENSMUSG00000028108	7.58	8.45	No
GSMG0050987	Casc5	up	1.82	4.76E-02	chr2(+):119047118-119104121	UCSC	cancer susceptibility candidate 5	NM_029617	76464	ENSMUSG00000027326	7.57	8.43	No
GSMG0012506	Dleu2	up	1.82	2.63E-02	chr14(-):61602837-61682373	UCSC	deleted in lymphocytic leukemia, 2	NR_028264	668253	---	8.55	9.41	No
GSMG0012296	Ero1l	up	1.82	1.78E-02	chr14(-):45283088-45318572	UCSC	ERO1-like (S. cerevisiae)	NM_015774	50527	ENSMUSG00000021831	8.96	9.82	No
GSMG0011374	Tkt	up	1.82	3.18E-02	chr14(+):30549144-30574722	UCSC	transketolase	AK030446	21881	ENSMUSG00000021957	10.23	11.09	No
GSMG0010518	Serpinb1a	up	1.82	3.69E-02	chr13(-):32842093-32851185	UCSC	serine (or cysteine) peptidase inhibitor, clade B, member 1a	NM_025429	66222	ENSMUSG00000044734	10.41	11.27	No
GSMG0009444	Lyst	up	1.82	1.29E-02	chr13(+):13590394-13778800	UCSC	lysosomal trafficking regulator	U70015	17101	ENSMUSG00000019726	10.4	11.26	No
GSMG0008871	---	up	1.82	2.34E-02	chr12(-):53303352-53309849	UCSC	---	AK006560	---	---	6.38	7.24	Yes
GSMG0005657	Slfn3	up	1.82	7.14E-03	chr11(+):83191330-83215143	UCSC	schlafen 3	NM_011409	20557	ENSMUSG00000018986	7.57	8.43	No
GSMG0040252	Deb1	up	1.82	5.54E-03	chr9(+):121710346-121713388	UCSC	differentially expressed in B16F10 1	AK078209	26901	ENSMUSG00000032526	7.86	8.73	No
GSMG0039109	Casp1	up	1.82	2.26E-03	chr9(+):5298517-5307289	UCSC	caspase 1	NM_009807	12362	ENSMUSG00000025888	8.79	9.65	No
GSMG0032014	Gng12	up	1.82	3.64E-02	chr6(+):66896397-67021361	UCSC	guanine nucleotide binding protein (G protein), gamma 12	NM_025278	14701	ENSMUSG00000036402	8.85	9.71	No
GSMG0030158	---	up	1.82	1.17E-02	chr5(-):7428172-7432355	UCSC	---	AK145349	---	---	7.64	8.5	Yes
GSMG0010435	Hist1h3a	up	1.82	1.47E-02	chr13(-):23761853-23762387	UCSC	histone cluster 1, H3a	AK018952	360198	ENSMUSG00000069265	8.46	9.32	Yes
GSMG0002490	Ifi204	up	1.81	1.41E-02	chr1(-):173747290-173766944	UCSC	interferon activated gene 204	NM_008329	15951	ENSMUSG00000073489	6.94	7.8	Yes
GSMG0018914	---	up	1.81	5.38E-03	chr18(-):43314763-43318030	UCSC	---	AK035189	---	---	6.9	7.76	Yes
GSMG0015806	5330426P16Rik	up	1.81	1.88E-02	chr16(-):50719297-50732773	UCSC	RIKEN cDNA 5330426P16 gene	AK151508	68190	ENSMUSG00000022639	7.93	8.79	No
GSMG0007866	---	up	1.81	1.17E-02	chr12(+):17757942-17760441	UCSC	---	AK136551	---	---	5.53	6.38	Yes
GSMG0006335	Tns3	up	1.81	4.29E-02	chr11(-):8431652-8664535	UCSC	tensin 3	NM_001083587	319939	ENSMUSG00000020422	7.61	8.46	Yes
GSMG0006320	---	up	1.81	2.50E-02	chr11(-):6415208-6416226	UCSC	---	AK076662	---	---	9.23	10.08	Yes
GSMG0039871	Ttk	up	1.81	4.89E-02	chr9(+):83834689-83872390	UCSC	Ttk protein kinase	NM_009445	22137	ENSMUSG00000038379	7	7.86	Yes
GSMG0000356	Gm11602	up	1.81	2.81E-02	chr1(+):63114471-63158295	UCSC	predicted gene 11602	NR_045915	100380944	ENSMUSG00000084799	5.42	6.28	No
GSMG0035517	Ube2m	up	1.81	6.82E-03	chr7(-):13035120-13038275	UCSC	ubiquitin-conjugating enzyme E2M	NM_001168469	22192	ENSMUSG00000005575	9.19	10.04	No
GSMG0031352	Atp5j2	up	1.81	8.00E-03	chr5(-):145183707-145191593	UCSC	ATP synthase, H+ transporting, mitochondrial F0 complex, subunit F2	AB030192	57423	ENSMUSG00000038690	10.39	11.25	No
GSMG0030368	Mxd4	up	1.81	1.42E-02	chr5(-):34173888-34174927	UCSC	Max dimerization protein 4	BC025016	17122	ENSMUSG00000037235	9.1	9.96	Yes
GSMG0022292	Osbpl2	up	1.81	5.32E-03	chr2(+):180119333-180162680	UCSC	oxysterol binding protein-like 2	AK145292	228983	ENSMUSG00000039050	9.33	10.18	No
GSMG0019264	Cdk2ap2	up	1.8	5.61E-04	chr19(+):4097346-4099017	UCSC	CDK2-associated protein 2	AK018035	52004	ENSMUSG00000024856	8.99	9.84	No
GSMG0007108	Slfn9	up	1.8	4.64E-03	chr11(-):82978412-82991832	UCSC	schlafen 9	AK036579	237886	ENSMUSG00000069793	6.74	7.59	Yes
GSMG0041780	F9	up	1.8	4.51E-02	chrX(+):59999458-60030759	UCSC	coagulation factor IX	AK149372	14071	ENSMUSG00000031138	6.06	6.91	Yes
GSMG0035283	Ptpre	up	1.8	1.72E-02	chr7(+):135537481-135686294	UCSC	protein tyrosine phosphatase, receptor type, E	NM_011212	19267	ENSMUSG00000041836	8.78	9.62	No
GSMG0033660	Emg1	up	1.8	4.30E-03	chr6(-):124704371-124712178	UCSC	EMG1 nucleolar protein homolog (S. cerevisiae)	NM_013536	14791	ENSMUSG00000004268	8.75	9.6	No
GSMG0030942	Selplg	up	1.8	3.04E-03	chr5(-):113817798-113830501	UCSC	selectin, platelet (p-selectin) ligand	NM_009151	20345	ENSMUSG00000048163	10.28	11.13	No
GSMG0030559	Rbm47	up	1.8	1.31E-02	chr5(-):66016557-66151924	UCSC	RNA binding motif protein 47	NM_001127382	245945	ENSMUSG00000070780	8.13	8.98	No
GSMG0003069	2210417K05Rik	up	1.8	9.45E-04	chr10(+):62340255-62349866	UCSC	RIKEN cDNA 2210417K05 gene	AK008969	70162	ENSMUSG00000085347	6.16	7.01	Yes
GSMG0026280	Ccne2	up	1.8	2.20E-02	chr4(+):11190925-11204779	UCSC	cyclin E2	AK158720	12448	ENSMUSG00000028212	6.91	7.76	No
GSMG0025670	S100a1	up	1.8	4.86E-02	chr3(-):90511034-90514392	UCSC	S100 calcium binding protein A1	BC005590	20193	ENSMUSG00000044080	7.81	8.66	No
GSMG0025317	Anxa5	up	1.8	1.22E-02	chr3(-):36448925-36475887	UCSC	annexin A5	NM_009673	11747	ENSMUSG00000027712	9.06	9.91	No
GSMG0003015	Smpdl3a	up	1.8	7.98E-03	chr10(+):57794544-57811830	UCSC	sphingomyelin phosphodiesterase, acid-like 3A	NM_020561	57319	ENSMUSG00000019872	8.79	9.64	No
GSMG0023392	Ino80	up	1.8	1.27E-02	chr2(-):119373042-119477630	UCSC	INO80 homolog (S. cerevisiae)	NM_026574	68142	ENSMUSG00000034154	8.53	9.38	No
GSMG0013348	Ncf4	up	1.79	3.27E-02	chr15(+):78244811-78262580	UCSC	neutrophil cytosolic factor 4	NM_008677	17972	ENSMUSG00000071715	9.84	10.68	No
GSMG0001440	---	up	1.79	3.87E-02	chr1(-):32967484-32972490	UCSC	---	AK155586	---	---	4.99	5.83	No
GSMG0012196	---	up	1.79	1.27E-02	chr14(-):31739154-31741458	UCSC	---	AK153672	---	---	5.48	6.33	No
GSMG0010930	---	up	1.79	3.27E-02	chr13(-):90030626-90035677	UCSC	---	AK157241	---	---	6.21	7.05	Yes
GSMG0007730	Actg1	up	1.79	3.67E-02	chr11(-):120345689-120348499	UCSC	actin, gamma, cytoplasmic 1	AK152202	11465	ENSMUSG00000062825	11.95	12.8	Yes
GSMG0041616	Kdm6a	up	1.79	5.23E-04	chrX(+):18162633-18279935	UCSC	lysine (K)-specific demethylase 6A	NM_009483	22289	ENSMUSG00000037369	9.18	10.02	No
GSMG0053277	---	up	1.79	2.04E-03	chrUn_JH584304(-):17987-60823	UCSC	---	AK036146	---	---	9.66	10.5	Yes
GSMG0034470	Gys1	up	1.79	3.54E-03	chr7(+):45434839-45456629	UCSC	glycogen synthase 1, muscle	NM_030678	14936	ENSMUSG00000003865	8.38	9.22	No
GSMG0000290	Ndufb3	up	1.79	1.42E-02	chr1(+):58586443-58595962	UCSC	NADH dehydrogenase (ubiquinone) 1 beta subcomplex 3	BC028669	66495	ENSMUSG00000026032	9.03	9.87	No
GSMG0025765	---	up	1.79	1.20E-02	chr3(-):96267075-96270290	UCSC	---	AK134918	---	---	6.81	7.65	No
GSMG0025079	Gng5	up	1.79	1.03E-02	chr3(+):146499836-146505543	UCSC	guanine nucleotide binding protein (G protein), gamma 5	NM_010318	14707	ENSMUSG00000068523	9.69	10.53	No
GSMG0021079	Arl6ip6	up	1.78	1.84E-03	chr2(+):53191430-53219221	UCSC	ADP-ribosylation factor-like 6 interacting protein 6	NM_022989	65103	ENSMUSG00000026960	8.27	9.1	Yes
GSMG0020163	Ostf1	up	1.78	8.16E-04	chr19(-):18579328-18631813	UCSC	osteoclast stimulating factor 1	AK149175	20409	ENSMUSG00000024725	10.72	11.55	No
GSMG0019694	Ubtd1	up	1.78	2.05E-02	chr19(+):41981763-42034643	UCSC	ubiquitin domain containing 1	NM_145500	226122	ENSMUSG00000025171	8.38	9.21	No
GSMG0017624	Tomm6	up	1.78	9.32E-03	chr17(-):47686646-47688386	UCSC	translocase of outer mitochondrial membrane 6 homolog (yeast)	NM_025365	66119	ENSMUSG00000033475	8.95	9.78	No
GSMG0016827	Ndufa11	up	1.78	9.64E-03	chr17(+):56717762-56724248	UCSC	NADH dehydrogenase (ubiquinone) 1 alpha subcomplex 11	NM_027244	69875	ENSMUSG00000002379	8.7	9.54	Yes
GSMG0014929	Ptplb	up	1.78	1.47E-02	chr16(+):35022421-35109177	UCSC	protein tyrosine phosphatase-like (proline instead of catalytic arginine), memt	NM_023587	70757	ENSMUSG00000035376	7.01	7.84	No
GSMG0013238	1700065I16Rik	up	1.78	5.58E-03	chr15(+):63817185-63819540	UCSC	RIKEN cDNA 1700065I16 gene	AK018879	78462	---	8.89	9.72	Yes
GSMG0012393	Slc7a7	up	1.78	4.08E-02	chr14(-):54369442-54417702	UCSC	solute carrier family 7 (cationic amino acid transporter, y+ system), member	NM_011405	20540	ENSMUSG00000000958	8.28	9.11	No
GSMG0012078	Gng2	up	1.78	4.55E-02	chr14(-):19872559-19977251	UCSC	guanine nucleotide binding protein (G protein), gamma 2	NM_010315	14702	ENSMUSG00000043004	8.62	9.46	No
GSMG0005391	2310047M10Rik	up	1.78	3.99E-02	chr11(+):69059732-69061576	UCSC	RIKEN cDNA 2310047M10 gene	AK009886	71923	ENSMUSG00000045176	7.78	8.61	No
GSMG0053393	Gm12070	up	1.78	2.72E-03	chr11(+):26785108-26787814	UCSC	predicted gene 12070	NR_002890	654472	ENSMUSG00000069939	12.17	13	Yes

FAST DB STABLE ID	Gene Symbol	Regulation	Fold-Change	P-Value	Gene Coordinates (mm10)	UCSC Link	Gene Name	Representative Transcript ID	Entrez Gene ID	EnsEMBL ID	log^2 intensity of untreated	log^2 intensity of treated	Low-specificity probe included
GSMG0042495	---	up	1.78	1.08E-02	chrX(-):51194877-51198845	UCSC	---	AK039980	---	---	6.65	7.48	No
GSMG0041163	Acpl2	up	1.78	3.64E-02	chr9(-):96823343-96889475	UCSC	acid phosphatase-like 2	AK084605	235534	ENSMUSG00000043587	7.43	8.27	No
GSMG0041060	Cox7a2	up	1.78	2.15E-02	chr9(-):79755241-79759853	UCSC	cytochrome c oxidase subunit VIIa 2	NM_009945	12866	ENSMUSG00000032330	9.82	10.64	No
GSMG0036358	Rhog	up	1.78	8.96E-03	chr7(-):102239123-102250118	UCSC	ras homolog gene family, member G	NM_019566	56212	ENSMUSG00000073982	9.83	10.66	No
GSMG0035419	Mboat7	up	1.78	1.34E-02	chr7(-):3677789-3693525	UCSC	membrane bound O-acyltransferase domain containing 7	NM_029934	77582	ENSMUSG00000035596	8.14	8.98	No
GSMG0034584	Vimp	up	1.78	5.46E-03	chr7(+):66079649-66089405	UCSC	VCP-interacting membrane protein	NM_024439	109815	ENSMUSG00000075701	7.75	8.58	No
GSMG0003469	Dusp6	up	1.78	5.92E-03	chr10(+):99263228-99267488	UCSC	dual specificity phosphatase 6	NM_026268	67603	ENSMUSG00000019960	10.58	11.41	No
GSMG0043318	---	up	1.78	2.93E-02	chr5(+):95861155-95861802	UCSC	---	AY618199	---	---	10.8	11.63	Yes
GSMG0027214	Rhd	up	1.78	1.87E-02	chr4(+):134864505-134896171	UCSC	Rh blood group, D antigen	NM_011270	19746	ENSMUSG00000028825	6.85	7.68	Yes
GSMG0027167	---	up	1.78	2.32E-02	chr4(+):132351772-132353382	UCSC	---	AK133170	---	---	6.9	7.73	No
GSMG0023559	1700037H04Rik	up	1.78	3.54E-03	chr2(-):131146325-131160081	UCSC	RIKEN cDNA 1700037H04 gene	BC071212	67326	ENSMUSG00000027327	7.92	8.76	No
GSMG0015099	C330027C09Rik	up	1.77	3.29E-02	chr16(+):48994180-49019705	UCSC	RIKEN cDNA C330027C09 gene	NM_172616	224171	ENSMUSG00000033031	8.43	9.25	No
GSMG0014212	Csf2rb2	up	1.77	2.20E-02	chr15(-):78282510-78305647	UCSC	colony stimulating factor 2 receptor, beta 2, low-affinity (granulocyte-macroph	NM_007781	12984	ENSMUSG00000071714	8.41	9.24	Yes
GSMG0013246	---	up	1.77	2.74E-03	chr15(+):66607475-66609362	UCSC	---	AK040209	---	---	7.79	8.62	No
GSMG0011696	---	up	1.77	3.40E-03	chr14(+):63837421-63841254	UCSC	---	AK132557	---	---	6.09	6.91	Yes
GSMG0001253	---	up	1.77	2.12E-02	chr1(+):193281271-193283843	UCSC	---	AK031888	---	---	8.39	9.21	No
GSMG0033237	Wbp1	up	1.77	9.22E-03	chr6(-):83119044-83121557	UCSC	WW domain binding protein 1	BC005666	22377	ENSMUSG00000030035	8.61	9.44	No
GSMG0031767	Zyx	up	1.77	4.73E-02	chr6(+):42346652-42360146	UCSC	zyxin	AK031415	22793	ENSMUSG00000029860	9.17	9.99	No
GSMG0030211	Ptpn12	up	1.77	4.28E-02	chr5(-):20986645-21055889	UCSC	protein tyrosine phosphatase, non-receptor type 12	AK154107	19248	ENSMUSG00000028771	7.92	8.74	No
GSMG0027682	Ndufb6	up	1.77	3.58E-04	chr4(-):40270663-40279370	UCSC	NADH dehydrogenase (ubiquinone) 1 beta subcomplex, 6	AK146762	230075	ENSMUSG00000071014	9.6	10.42	No
GSMG0024539	Krtcap2	up	1.77	1.51E-02	chr3(+):89245966-89249725	UCSC	keratinocyte associated protein 2	BC043030	66059	ENSMUSG00000042747	10.07	10.89	No
GSMG0022126	Ywhab	up	1.76	2.35E-02	chr2(+):163995134-164018587	UCSC	tyrosine 3-monooxygenase/tryptophan 5-monooxygenase activation protein,	AK004872	54401	ENSMUSG00000018326	10.59	11.4	Yes
GSMG0016574	Clic1	up	1.76	3.31E-02	chr17(+):35049964-35058720	UCSC	chloride intracellular channel 1	AK153509	114584	ENSMUSG00000007041	9.94	10.76	No
GSMG0016430	Snrpc	up	1.76	1.14E-02	chr17(+):27840087-27851965	UCSC	U1 small nuclear ribonucleoprotein C	NM_011432	20630	ENSMUSG00000024217	8.02	8.84	Yes
GSMG0015988	Atp5j	up	1.76	1.20E-02	chr16(-):84827867-84835574	UCSC	ATP synthase, H+ transporting, mitochondrial F0 complex, subunit F	NM_016755	11957	ENSMUSG00000022890	9.5	10.31	Yes
GSMG0013883	Gm10384	up	1.76	1.58E-02	chr15(-):36870501-36879820	UCSC	predicted gene 10384	AK142674	100038735	ENSMUSG00000072618	6.7	7.52	Yes
GSMG0001505	---	up	1.76	3.21E-02	chr1(-):38417704-38421336	UCSC	---	AK041222	---	---	6.85	7.67	No
GSMG0012430	Dhrs1	up	1.76	4.20E-03	chr14(-):55739022-55745684	UCSC	dehydrogenase/reductase (SDR family) member 1	NM_026819	52585	ENSMUSG00000002332	8.69	9.5	No
GSMG0011666	Arl11	up	1.76	2.50E-02	chr14(+):61309753-61311936	UCSC	ADP-ribosylation factor-like 11	NM_177337	219144	ENSMUSG00000043157	7.84	8.65	No
GSMG0008248	Rdh12	up	1.76	4.96E-02	chr12(+):79208911-79222663	UCSC	retinol dehydrogenase 12	AK039233	77974	ENSMUSG00000021123	7.98	8.79	Yes
GSMG0038572	Haus8	up	1.76	8.68E-03	chr8(-):71248561-71272590	UCSC	4HAUS augmin-like complex, subunit 8	AK161850	76478	ENSMUSG00000035439	7.58	8.39	No
GSMG0036985	Tnfsf13b	up	1.76	1.53E-02	chr8(+):10006465-10037845	UCSC	tumor necrosis factor (ligand) superfamily, member 13b	AK034121	24099	ENSMUSG00000031497	6.55	7.37	No
GSMG0043359	---	up	1.76	1.56E-02	chr7(-):27469074-27469500	UCSC	---	AK177942	---	---	10.37	11.19	Yes
GSMG0035170	Bola2	up	1.76	5.63E-04	chr7(+):126695971-126696696	UCSC	bolA-like 2 (E. coli)	BC061197	66162	ENSMUSG00000047721	8.73	9.54	No
GSMG0034104	Snrpd2	up	1.76	4.59E-02	chr7(+):19149838-19152727	UCSC	small nuclear ribonucleoprotein D2	NM_026943	107686	ENSMUSG00000040824	8.48	9.3	No
GSMG0032369	March8	up	1.76	2.72E-02	chr6(+):116338021-116409540	UCSC	membrane-associated ring finger (C3HC4) 8	AK154976	71779	ENSMUSG00000025702	7.15	7.96	No
GSMG0029815	Setd8	up	1.76	2.74E-02	chr5(+):124439930-124462307	UCSC	SET domain containing (lysine methyltransferase) 8	NM_030241	67956	ENSMUSG00000049327	9.04	9.86	Yes
GSMG0025651	Adam15	up	1.76	4.50E-02	chr3(-):89338542-89350010	UCSC	a disintegrin and metallopeptidase domain 15 (metargidin)	AK149796	11490	ENSMUSG00000028041	7.96	8.77	No
GSMG0015760	Slc35a5	up	1.75	1.36E-02	chr16(-):45139573-45158673	UCSC	solute carrier family 35, member A5	NM_028756	74102	ENSMUSG00000022664	8.4	9.21	No
GSMG0015251	Jam2	up	1.75	1.37E-02	chr16(+):84774123-84826356	UCSC	junction adhesion molecule 2	NM_023844	67374	ENSMUSG00000053062	5.93	6.74	Yes
GSMG0001524	Gm16894	up	1.75	1.86E-02	chr1(-):40076212-40085853	UCSC	predicted gene, 16894	NR_037980	100047133	ENSMUSG00000097899	6.52	7.33	Yes
GSMG0012731	---	up	1.75	3.94E-03	chr14(-):90082514-90085239	UCSC	---	AK145750	---	---	6.72	7.53	Yes
GSMG0004780	Cdk2	up	1.75	1.10E-02	chr10(-):128697939-128705051	UCSC	cyclin-dependent kinase 2	NM_183417	12566	ENSMUSG00000025358	9.37	10.18	No
GSMG0034907	Numa1	up	1.75	4.51E-02	chr7(+):101934110-102014958	UCSC	nuclear mitotic apparatus protein 1	AK168399	101706	ENSMUSG00000066306	7	7.81	No
GSMG0034531	---	up	1.75	4.20E-02	chr7(+):50387360-50388818	UCSC	---	AK081603	---	---	5.21	6.02	Yes
GSMG0032793	---	up	1.75	4.30E-02	chr6(-):28211008-28214806	UCSC	---	AK034112	---	---	7.69	8.5	Yes
GSMG0032750	Tfec	up	1.75	3.33E-02	chr6(-):16833381-16898441	UCSC	transcription factor EC	NM_031198	21426	ENSMUSG00000029553	7.1	7.91	No
GSMG0030018	Kdelr2	up	1.75	1.06E-02	chr5(+):143403820-143421900	UCSC	KDEL (Lys-Asp-Glu-Leu) endoplasmic reticulum protein retention receptor 2	NM_025841	66913	ENSMUSG00000079111	8.93	9.74	Yes
GSMG0025625	Sema4a	up	1.75	4.42E-02	chr3(-):88435962-88461182	UCSC	sema domain, immunoglobulin domain (Ig), transmembrane domain (TM) and	X85991	20351	ENSMUSG00000028064	9.01	9.82	Yes
GSMG0023261	---	up	1.75	3.46E-03	chr2(-):104658584-104664741	UCSC	---	AK136931	---	---	6.59	7.4	Yes
GSMG0022698	Ndufa8	up	1.75	9.23E-04	chr2(-):36036329-36049309	UCSC	NADH dehydrogenase (ubiquinone) 1 alpha subcomplex, 8	AK088463	68375	ENSMUSG00000026895	9.29	10.09	No
GSMG0002368	Dcaf6	up	1.74	1.08E-02	chr1(-):165329500-165460463	UCSC	DDB1 and CUL4 associated factor 6	NM_028759	74106	ENSMUSG00000026571	8.34	9.14	No
GSMG0013296	Grina	up	1.74	2.54E-02	chr15(+):76246787-76249909	UCSC	glutamate receptor, ionotropic, N-methyl D-aspartate-associated protein 1 (g	BC019157	66168	ENSMUSG00000022564	10.64	11.44	No
GSMG0000111	---	up	1.74	2.44E-02	chr1(+):32496444-32497988	UCSC	---	AK052137	---	---	6	6.8	Yes
GSMG0005594	Unc119	up	1.74	2.36E-02	chr11(+):78343507-78349156	UCSC	unc-119 homolog (C. elegans)	AK131955	22248	ENSMUSG00000002058	9.01	9.81	No
GSMG0043022	Tlr8	up	1.74	3.29E-02	chrX(-):167242693-167263809	UCSC	toll-like receptor 8	AY035890	170744	ENSMUSG00000040522	7.01	7.81	No
GSMG0040083	Gpx1	up	1.74	7.74E-03	chr9(+):108339080-108340340	UCSC	glutathione peroxidase 1	NM_008160	14775	ENSMUSG00000063856	10.99	11.79	No
GSMG0035734	Capns1	up	1.74	4.98E-03	chr7(-):30186936-30195048	UCSC	calpain, small subunit 1	NM_009795	12336	ENSMUSG00000001794	10.65	11.45	No
GSMG0035028	Ampd3	up	1.74	2.46E-02	chr7(+):110768206-110812409	UCSC	adenosine monophosphate deaminase 3	NM_001276301	11717	ENSMUSG00000005686	8.78	9.58	No
GSMG0033942	Rps9	up	1.74	1.17E-02	chr7(+):3704037-3706897	UCSC	ribosomal protein S9	AK151621	76846	ENSMUSG00000006333	11.22	12.02	No
GSMG0033504	---	up	1.74	1.14E-02	chr6(-):108521518-108523166	UCSC	---	AK137713	---	---	5.35	6.15	Yes
GSMG0029867	Tpst1	up	1.74	2.46E-02	chr5(+):130073326-130135732	UCSC	protein-tyrosine sulfotransferase 1	NM_013837	22021	ENSMUSG00000034118	8.47	9.27	No
GSMG0051027	Gm6623	up	1.74	4.88E-03	chr17(+):36178777-36181364	UCSC	predicted gene 6623	NR_033619	625785	ENSMUSG00000092374	8.01	8.81	Yes
GSMG0050825	Dnah7b	up	1.73	2.79E-04	chr1(+):46066738-46373550	UCSC	dynein, axonemal, heavy chain 7B	NM_001160386	227058	ENSMUSG00000041144	6.7	7.5	No
GSMG0017770	Vapa	up	1.73	7.16E-04	chr17(-):65578327-65613556	UCSC	vesicle-associated membrane protein, associated protein A	NM_013933	30960	ENSMUSG00000024091	9.87	10.66	No
GSMG0017424	---	up	1.73	6.20E-03	chr17(-):34211312-34214459	UCSC	---	AK007825	---	---	8.49	9.29	No
GSMG0017139	Phf10	up	1.73	1.53E-02	chr17(-):14944994-14961260	UCSC	PHD finger protein 10	NM_024250	106740	ENSMUSG00000023883	8	8.79	No
GSMG0016607	Flot1	up	1.73	5.30E-03	chr17(+):35823335-35832787	UCSC	flotillin 1	AK150692	14251	ENSMUSG00000059714	8.15	8.94	Yes
GSMG0012694	Dnajc15	up	1.73	9.26E-03	chr14(-):77826215-77874927	UCSC	DnaJ (Hsp40) homolog, subfamily C, member 15	NM_025384	66148	ENSMUSG00000022013	7.45	8.24	No
GSMG0012433	Ripk3	up	1.73	3.64E-02	chr14(-):55784996-55788857	UCSC	receptor-interacting serine-threonine kinase 3	NM_019955	56532	ENSMUSG00000022221	8.73	9.52	No
GSMG0051418	Olfr726	up	1.73	4.32E-02	chr14(-):50083714-50084679	UCSC	olfactory receptor 726	NM_146316	258313	ENSMUSG00000060523	6.16	6.95	Yes
GSMG0000958	Cenpl	up	1.73	3.88E-02	chr1(+):161070890-161086722	UCSC	centromere protein L	BC063765	70454	ENSMUSG00000026708	7.82	8.61	No
GSMG0008331	Eif2b2	up	1.73	5.92E-03	chr12(+):85219481-85226628	UCSC	eukaryotic translation initiation factor 2B, subunit 2 beta	AK049731	217715	ENSMUSG00000004788	9.39	10.18	No
GSMG0040987	---	up	1.73	3.48E-02	chr9(-):71910496-71911656	UCSC	---	AK017987	---	---	6.95	7.74	Yes
GSMG0039980	---	up	1.73	4.96E-03	chr9(+):100665839-100669799	UCSC	---	AK153844	---	---	6.05	6.84	No
GSMG0027359	Gm13242	up	1.73	4.22E-02	chr4(+):145670694-145704435	UCSC	predicted gene 13242	BC096593	100041379	ENSMUSG00000058186	6.64	7.43	Yes
GSMG0026986	Med8	up	1.73	5.56E-03	chr4(+):118409335-118415782	UCSC	mediator of RNA polymerase II transcription, subunit 8 homolog (yeast)	NM_173719	80509	ENSMUSG00000006392	8.21	9	Yes
GSMG0025871	---	up	1.73	3.05E-02	chr3(-):105782875-105785027	UCSC	---	AK141336	---	---	7.96	8.75	No
GSMG0019477	Gnaq	up	1.72	8.38E-03	chr19(+):16132829-16387453	UCSC	guanine nucleotide binding protein, alpha q polypeptide	AK147393	14682	ENSMUSG00000024639	8.89	9.67	No
GSMG0017670	Sgol1	up	1.72	3.02E-02	chr17(-):53674787-53689334	UCSC	shugoshin-like 1 (S. pombe)	NM_028232	72415	ENSMUSG00000023940	7.05	7.82	No
GSMG0016286	Fpr-rs4	up	1.72	2.31E-02	chr17(+):18021733-18022704	UCSC	formyl peptide receptor, related sequence 4	NM_008041	14291	ENSMUSG00000048062	6.72	7.5	Yes
GSMG0015526	Mrpl40	up	1.72	1.02E-02	chr16(-):18872018-18876637	UCSC	mitochondrial ribosomal protein L40	NM_010922	18100	ENSMUSG00000022706	8.15	8.93	No
GSMG0015346	---	up	1.72	1.24E-03	chr16(+):96036088-96038743	UCSC	---	AK133374	---	---	6.43	7.21	Yes
GSMG0011852	Kbtbd7	up	1.72	3.24E-02	chr14(+):79426392-79429253	UCSC	kelch repeat and BTB (POZ) domain containing 7	AK135854	211255	ENSMUSG00000043881	8.44	9.22	Yes
GSMG0010402	Hist1h4i	up	1.72	1.71E-02	chr13(-):22040638-22041363	UCSC	histone cluster 1, H4i	AK011560	319158	ENSMUSG00000060639	8.46	9.25	Yes
GSMG0009541	Hist1h2bb	up	1.72	3.82E-02	chr13(+):23746734-23747202	UCSC	histone cluster 1, H2bb	NM_175664	319178	ENSMUSG00000075031	7.46	8.25	No
GSMG0005925	Atp6v0a1	up	1.72	9.96E-03	chr11(+):101009452-101063715	UCSC	ATPase, H+ transporting, lysosomal V0 subunit A1	NM_016920	11975	ENSMUSG00000019302	9.06	9.85	No
GSMG0038629	Ednra	up	1.72	2.56E-02	chr8(-):77663029-77724465	UCSC	endothelin receptor type A	NM_010332	13617	ENSMUSG00000031616	7.24	8.02	Yes
GSMG0037176	Gtf2e2	up	1.72	2.27E-02	chr8(+):33731831-33777173	UCSC	general transcription factor II E, polypeptide 2 (beta subunit)	AK158783	68153	ENSMUSG00000031585	8.18	8.97	No
GSMG0004163	---	up	1.72	4.14E-02	chr10(-):62505859-62506910	UCSC	---	AK040025	---	---	9.44	10.22	Yes
GSMG0028451	Atpif1	up	1.72	9.76E-03	chr4(-):132530555-132535415	UCSC	ATPase inhibitory factor 1	AK152389	11983	ENSMUSG00000054428	9.94	10.72	Yes
GSMG0021173	---	up	1.71	2.42E-02	chr2(+):69051118-69054254	UCSC	---	AK083066	---	---	7.49	8.27	No
GSMG0020211	Fam122a	up	1.71	1.68E-02	chr19(-):24475777-24477474	UCSC	family with sequence similarity 122, member A	AK033646	68034	ENSMUSG00000074922	7.98	8.75	No
GSMG0020116	Ms4a7	up	1.71	4.74E-02	chr19(-):11321039-11336148	UCSC	membrane-spanning 4-domains, subfamily A, member 7	NM_027836	109225	ENSMUSG00000024672	6.66	7.44	No
GSMG0018929	---	up	1.71	6.27E-05	chr18(-):44827746-44828925	UCSC	---	AK052308	---	---	7	7.77	No
GSMG0043812	Eif4ebp3 // Ankhd1	up	1.71	4.20E-02	chr18(+):36560603-36666324	UCSC	eukaryotic translation initiation factor 4E binding protein 3 // ankyrin repeat ar	NM_175375	108112 // 108857	USG00000024483 // ENSMUSG00000	7.92	8.69	Yes
GSMG0017776	Ralbp1	up	1.71	2.58E-02	chr17(-):65848431-65885799	UCSC	ralA binding protein 1	NM_009067	19765	ENSMUSG00000024096	9.13	9.9	No
GSMG0014876	Cep19	up	1.71	1.88E-02	chr16(+):32099802-32108055	UCSC	centrosomal protein 19	NM_025892	66994	ENSMUSG00000035790	7.63	8.41	No
GSMG0010777	6720489N17Rik	up	1.71	1.94E-03	chr13(-):62603015-62624185	UCSC	RIKEN cDNA 6720489N17 gene	NM_173381	211378	ENSMUSG00000072066	6.43	7.2	No
GSMG0009343	2010107E04Rik	up	1.71	5.34E-03	chr12(-):111961376-111966978	UCSC	RIKEN cDNA 2010107E04 gene	AK008333	70257	ENSMUSG00000021290	9.06	9.84	No
GSMG0009091	Dpf3	up	1.71	3.01E-02	chr12(-):83213751-83487738	UCSC	D4, zinc and double PHD fingers, family 3	NM_001267625	70127	ENSMUSG00000021221	7.44	8.21	Yes
GSMG0005555	---	up	1.71	2.86E-02	chr11(+):77227876-77229732	UCSC	---	AK140603	---	---	6.95	7.72	No
GSMG0043001	Zrsr2	up	1.71	4.37E-02	chrX(-):163935443-163958666	UCSC	zinc finger (CCCH type), RNA binding motif and serine/arginine rich 2	NM_009453	22184	ENSMUSG00000031370	7.53	8.31	No
GSMG0036762	Chst15	up	1.71	3.38E-03	chr7(-):132231501-132317221	UCSC	carbohydrate (N-acetylgalactosamine 4-sulfate 6-O) sulfotransferase 15	AK083614	77590	ENSMUSG00000030930	9.08	9.85	No
GSMG0032729	Ndufa4	up	1.71	2.32E-02	chr6(-):11900373-11907451	UCSC	NADH dehydrogenase (ubiquinone) 1 alpha subcomplex, 4	AK005084	17992	ENSMUSG00000029632	9.45	10.23	Yes
GSMG0025290	Mrpl47	up	1.71	8.32E-03	chr3(-):32725403-32736755	UCSC	mitochondrial ribosomal protein L47	BC029173	74600	ENSMUSG00000037531	7.47	8.25	Yes
GSMG0021610	Knstrn	up	1.7	1.42E-02	chr2(+):118814002-118836250	UCSC	kinetochore-localized astrin/SPAG5 binding	NM_026412	51944	ENSMUSG00000027331	7.99	8.75	No
GSMG0000488	---	up	1.7	3.24E-02	chr1(+):85326729-85343516	UCSC	---	BC108341	---	---	6.96	7.72	Yes
GSMG0019244	---	up	1.7	1.58E-02	chr19(+):3205605-3206258	UCSC	---	AK007267	---	---	8.32	9.08	Yes
GSMG0015426	Emp2	up	1.7	3.15E-02	chr16(-):10281749-10313970	UCSC	epithelial membrane protein 2	NM_007929	13731	ENSMUSG00000022505	6.8	7.57	Yes
GSMG0011009	---	up	1.7	1.77E-02	chr13(-):98806492-98809572	UCSC	---	AK046946	---	---	7.14	7.91	Yes
GSMG0008357	Slirp	up	1.7	2.41E-02	chr12(+):87443896-87452208	UCSC	SRA stem-loop interacting RNA binding protein	AK028746	380773	ENSMUSG00000021040	7.58	8.35	No
GSMG0005137	Mgat4b	up	1.7	7.82E-03	chr11(+):50225333-50235103	UCSC	mannoside acetylglucosaminyltransferase 4, isoenzyme B	NM_145926	103534	ENSMUSG00000036620	7.36	8.13	No
GSMG0041156	Rasa2	up	1.7	7.00E-03	chr9(-):96539300-96631537	UCSC	RAS p21 protein activator 2	NM_053268	114713	ENSMUSG00000032413	8.74	9.5	Yes
GSMG0040477	Rp9	up	1.7	2.86E-02	chr9(-):22411047-22468359	UCSC	retinitis pigmentosa 9 (human)	AK147912	55934	ENSMUSG00000032239	8.46	9.23	No
GSMG0043349	---	up	1.7	2.14E-02	chr7(+):72674599-72674883	UCSC	---	AK217757	---	---	8.88	9.65	Yes
GSMG0002778	Sgk1	up	1.7	2.03E-02	chr10(+):21882184-21999900	UCSC	serum/glucocorticoid regulated kinase 1	NM_001161845	20393	ENSMUSG00000019970	9.56	10.32	No
GSMG0019780	---	up	1.69	8.10E-03	chr19(+):47020117-47021540	UCSC	---	AK039077	---	---	5.82	6.57	Yes
GSMG0002037	---	up	1.69	2.90E-02	chr1(-):126106922-126108293	UCSC	---	AK086957	---	---	5.47	6.23	Yes
GSMG0015075	Cd200r1	up	1.69	1.13E-02	chr16(+):44765736-44794977	UCSC	CD200 receptor 1	NM_021325	57781	ENSMUSG00000022667	7.35	8.11	No
GSMG0011795	---	up	1.69	3.28E-02	chr14(+):74338388-74349636	UCSC	---	AY139115	---	---	6.38	7.14	Yes
GSMG0040946	Aph1b	up	1.69	3.21E-02	chr9(-):66775209-66795491	UCSC	anterior pharynx defective 1b homolog (C. elegans)	AK162585	208117	ENSMUSG00000032375	7.73	8.49	No
GSMG0039943	---	up	1.69	1.79E-02	chr9(+):96119989-96122127	UCSC	---	AK078564	---	---	7.6	8.36	Yes
GSMG0039432	Dpagt1	up	1.69	1.02E-02	chr9(+):44326845-44333894	UCSC	dolichyl-phosphate (UDP-N-acetylglucosamine) acetylglucosaminephosphotra	AK008317	13478	ENSMUSG00000032123	8.72	9.48	No
GSMG0004403	C330046G13Rik	up	1.69	3.68E-02	chr10(-):84547417-84553339	UCSC	RIKEN cDNA C330046G13 gene	AK082870	442847	ENSMUSG00000097076	6.4	7.15	Yes
GSMG0036856	Rnh1	up	1.69	2.77E-04	chr7(-):141160322-141172851	UCSC	ribonuclease/angiogenin inhibitor 1	NM_001172100	107702	ENSMUSG00000038650	9.16	9.92	No
GSMG0035808	Pdcd5	up	1.69	1.84E-02	chr7(-):35641982-35647497	UCSC	programmed cell death 5	NM_019746	56330	ENSMUSG00000030417	9.19	9.95	Yes
GSMG0032434	Gm10319	up	1.69	2.27E-02	chr6(+):122136628-122150958	UCSC	predicted pseudogene 10319	NR_003624	381806	ENSMUSG00000071204	6.18	6.94	Yes
GSMG0029771	Arpc3	up	1.69	2.66E-03	chr5(+):122391897-122406178	UCSC	actin related protein 2/3 complex, subunit 3	AK003485	56378	ENSMUSG00000029465	9.74	10.49	No
GSMG0028512	Lypla2	up	1.69	4.82E-04	chr4(-):135968225-135972620	UCSC	lysophospholipase 2	BC068120	26394	ENSMUSG00000028670	8.75	9.5	No
GSMG0025759	Bola1	up	1.69	1.22E-02	chr3(-):96196588-96198488	UCSC	bolA-like 1 (E. coli)	JX420286	69168	ENSMUSG00000015943	7.12	7.88	No
GSMG0002657	G0s2	up	1.69	3.06E-02	chr1(-):193272160-193273188	UCSC	G0/G1 switch gene 2	NM_008059	14373	ENSMUSG00000009633	8.09	8.85	No
GSMG0043250	---	up	1.68	1.01E-02	chr19(-):42378611-42378855	UCSC	---	AK207983	---	---	6.56	7.31	Yes
GSMG0002533	---	up	1.68	1.11E-02	chr1(-):177792033-177795509	UCSC	---	AK053488	---	---	8.88	9.63	Yes
GSMG0002407	Pbx1	up	1.68	2.00E-02	chr1(-):168119365-168432258	UCSC	pre B cell leukemia homeobox 1	NM_008783	18514	ENSMUSG00000052534	7.78	8.53	No
GSMG0019998	Cdc42ep2	up	1.68	2.46E-02	chr19(-):5916128-5924817	UCSC	CDC42 effector protein (Rho GTPase binding) 2	AK083004	104252	ENSMUSG00000045664	7.53	8.27	No
GSMG0019861	Atrnl1	up	1.68	2.91E-02	chr19(+):57611033-58133338	UCSC	attractin like 1	AK147492	226255	ENSMUSG00000054843	8.27	9.02	No
GSMG0019516	---	up	1.68	2.42E-02	chr19(+):21992580-22021025	UCSC	---	AK077070	---	---	6.02	6.76	Yes
GSMG0016833	Alkbh7	up	1.68	4.33E-02	chr17(+):56997338-56999340	UCSC	alkB, alkylation repair homolog 7 (E. coli)	NM_027372	66400	ENSMUSG00000002661	7.36	8.11	No
GSMG0011970	Rap2a	up	1.68	3.81E-02	chr14(+):120478444-120507194	UCSC	RAS related protein 2a	BC067194	76108	ENSMUSG00000051615	7.71	8.46	No
GSMG0001263	---	up	1.68	3.20E-02	chr1(+):194993260-194996195	UCSC	---	AK148519	---	---	8.48	9.23	No
GSMG0001059	Copa	up	1.68	1.40E-02	chr1(+):172082529-172122332	UCSC	coatomer protein complex subunit alpha	NM_009938	12847	ENSMUSG00000026553	9.88	10.63	No
GSMG0008083	Psma6	up	1.68	1.62E-03	chr12(+):55398825-55418459	UCSC	proteasome (prosome, macropain) subunit, alpha type 6	NM_011968	26443	ENSMUSG00000021024	9.9	10.65	No
GSMG0005595	Ift20	up	1.68	1.97E-02	chr11(+):78536400-78541512	UCSC	intraflagellar transport 20	AK139942	55978	ENSMUSG00000001105	10.05	10.8	No
GSMG0042240	---	up	1.68	4.02E-02	chrX(+):159301731-159304435	UCSC	---	AK041476	---	---	7.8	8.55	No
GSMG0040893	Gm10653	up	1.68	1.80E-02	chr9(-):62841477-62862025	UCSC	predicted gene 10653	AK131930	677044	ENSMUSG00000074250	11.52	12.28	Yes
GSMG0040144	Ccdc12	up	1.68	9.18E-03	chr9(+):110656502-110711592	UCSC	coiled-coil domain containing 12	NM_028312	72654	ENSMUSG00000019659	7.8	8.55	No
GSMG0034906	Lamtor1	up	1.68	5.19E-04	chr7(+):101905837-101911901	UCSC	late endosomal/lysosomal adaptor, MAPK and MTOR activator 1	NM_025605	66508	ENSMUSG00000030842	8.25	9	Yes
GSMG0033347	Abtb1	up	1.68	1.67E-02	chr6(-):88835915-88841935	UCSC	ankyrin repeat and BTB (POZ) domain containing 1	NM_030251	80283	ENSMUSG00000030083	8.75	9.5	No
GSMG0032725	---	up	1.68	3.58E-02	chr6(-):8725128-8728731	UCSC	---	AK086162	---	---	7.24	7.99	No
GSMG0032348	Atg7	up	1.68	1.69E-02	chr6(+):114643097-114860612	UCSC	autophagy related 7	NM_028835	74244	ENSMUSG00000030314	8.92	9.67	No
GSMG0026960	Prdx1	up	1.68	6.28E-03	chr4(+):116685597-116700820	UCSC	peroxiredoxin 1	AK145138	18477	ENSMUSG00000028691	9.9	10.64	Yes
GSMG0024275	4930577N17Rik	up	1.68	1.78E-02	chr3(+):51276758-51278407	UCSC	RIKEN cDNA 4930577N17 gene	NR_073429	---	ENSMUSG00000087440	7.1	7.85	No
GSMG0021668	Serf2	up	1.67	8.30E-03	chr2(+):121449228-121453426	UCSC	small EDRK-rich factor 2	NM_011354	378702	ENSMUSG00000074884	9.75	10.49	No
GSMG0043803	A330069E16Rik	up	1.67	2.79E-02	chr2(+):91237146-91238355	UCSC	RIKEN cDNA A330069E16 gene	NR_015464	606735	ENSMUSG00000097868	5.97	6.71	Yes
GSMG0019877	1810007D17Rik	up	1.67	2.62E-03	chr19(+):58606325-58629753	UCSC	RIKEN cDNA 1810007D17 gene	AK007359	69055	---	8.07	8.81	Yes
GSMG0019514	Tmem2	up	1.67	1.68E-02	chr19(+):21778342-21858327	UCSC	transmembrane protein 2	NM_031997	83921	ENSMUSG00000024754	8.22	8.96	No
GSMG0019480	---	up	1.67	2.92E-03	chr19(+):16242302-16243798	UCSC	---	AK047890	---	---	7.39	8.12	Yes
GSMG0001847	A530032D15Rik	up	1.67	4.33E-02	chr1(-):85083077-85109854	UCSC	RIKEN cDNA A530032D15Rik gene	BC094285	381287	ENSMUSG00000089844	8.11	8.85	Yes
GSMG0040617	4931429I11Rik	up	1.67	7.86E-03	chr9(-):40894849-40964112	UCSC	RIKEN cDNA 4931429I11 gene	NM_001081121	70989	ENSMUSG00000032023	7.53	8.26	No
GSMG0040219	Acaa1a	up	1.67	2.77E-02	chr9(+):119341294-119350298	UCSC	acetyl-Coenzyme A acyltransferase 1A	NM_130864	113868	ENSMUSG00000036138	8.11	8.85	No
GSMG0039897	Bcl2a1b	up	1.67	2.80E-02	chr9(+):89199273-89207838	UCSC	B cell leukemia/lymphoma 2 related protein A1b	NM_007534	12045	ENSMUSG00000089929	8.28	9.02	Yes
GSMG0034593	---	up	1.67	2.20E-02	chr7(+):67030911-67033053	UCSC	---	AK086977	---	---	7.52	8.26	Yes
GSMG0003164	Ggt1	up	1.67	3.35E-02	chr10(+):75568662-75586190	UCSC	gamma-glutamyltransferase 1	AK079235	14598	ENSMUSG00000006345	7.37	8.12	No
GSMG0026998	Ccdc23	up	1.67	3.47E-02	chr4(+):119195310-119201299	UCSC	coiled-coil domain containing 23	NM_024462	69216	ENSMUSG00000028643	6.95	7.69	Yes
GSMG0026607	Gng10	up	1.67	3.20E-02	chr4(+):59035156-59041899	UCSC	guanine nucleotide binding protein (G protein), gamma 10	NM_025277	14700	ENSMUSG00000038607	9.33	10.07	No
GSMG0026325	Ggh	up	1.67	7.02E-03	chr4(+):20042052-20066111	UCSC	gamma-glutamyl hydrolase	NM_010281	14590	ENSMUSG00000073987	7.05	7.79	No
GSMG0025876	Chi3l7	up	1.67	2.27E-02	chr3(-):106016951-106032897	UCSC	chitinase 3-like 7	AB081756	229687	ENSMUSG00000043873	6.78	7.52	No
GSMG0024568	Rab13	up	1.67	4.52E-02	chr3(+):90213714-90226385	UCSC	RAB13, member RAS oncogene family	AK080805	68328	ENSMUSG00000027935	6.06	6.8	Yes
GSMG0002181	Cfh	up	1.66	1.63E-02	chr1(-):140085855-140183411	UCSC	complement component factor h	NM_009888	12628	ENSMUSG00000026365	5.78	6.51	Yes
GSMG0013770	Rai14	up	1.66	1.44E-02	chr15(-):10568979-10714631	UCSC	retinoic acid induced 14	NM_001166408	75646	ENSMUSG00000022246	8.11	8.84	Yes
GSMG0011276	Chchd1	up	1.66	7.74E-03	chr14(+):20703027-20704417	UCSC	coiled-coil-helix-coiled-coil-helix domain containing 1	NM_025366	66121	ENSMUSG00000063787	8.03	8.76	No
GSMG0011109	Ndufaf2	up	1.66	1.05E-02	chr13(-):108002715-108158627	UCSC	NADH dehydrogenase (ubiquinone) 1 alpha subcomplex, assembly factor 2	AK080810	75597	ENSMUSG00000068184	6.6	7.33	No
GSMG0008544	Ppp2r5c	up	1.66	1.75E-02	chr12(+):110447179-110583061	UCSC	protein phosphatase 2, regulatory subunit B (B56), gamma isoform	NM_001135001	26931	ENSMUSG00000017843	9.69	10.42	No
GSMG0006037	Milr1	up	1.66	9.54E-03	chr11(+):106751226-106768794	UCSC	mast cell immunoglobulin like receptor 1	NR_073165	380732	ENSMUSG00000040528	8.77	9.5	No
GSMG0005401	Trappc1	up	1.66	8.52E-03	chr11(+):69323986-69325793	UCSC	trafficking protein particle complex 1	NM_001024206	245828	ENSMUSG00000049299	9.09	9.82	No
GSMG0041475	Ano10	up	1.66	9.40E-03	chr9(-):122175874-122294423	UCSC	anoctamin 10	NM_133979	102566	ENSMUSG00000037949	8.94	9.67	No
GSMG0040790	Etfa	up	1.66	3.98E-03	chr9(-):55454432-55512244	UCSC	electron transferring flavoprotein, alpha polypeptide	NM_145615	110842	ENSMUSG00000032314	9.15	9.88	No
GSMG0004452	Apaf1	up	1.66	3.51E-02	chr10(-):90989311-91082770	UCSC	apoptotic peptidase activating factor 1	NM_009684	11783	ENSMUSG00000019979	8.64	9.38	No
GSMG0035586	Gemin7	up	1.66	5.08E-03	chr7(-):19564947-19573394	UCSC	gem (nuclear organelle) associated protein 7	AK010303	69731	ENSMUSG00000044709	8.33	9.07	No
GSMG0035211	Bckdk	up	1.66	4.13E-02	chr7(+):127904073-127909664	UCSC	branched chain ketoacid dehydrogenase kinase	NM_009739	12041	ENSMUSG00000030802	9.27	10	No
GSMG0030431	---	up	1.66	1.65E-02	chr5(-):43752909-43754271	UCSC	---	AK041730	---	---	7.93	8.66	No
GSMG0027870	Inip	up	1.66	3.36E-03	chr4(-):59769665-59783855	UCSC	INTS3 and NABP interacting protein	NM_001013577	66209	ENSMUSG00000038544	7.25	7.98	No
GSMG0026762	---	up	1.66	2.70E-03	chr4(+):98021516-98025483	UCSC	---	AK085001	---	---	5.24	5.97	Yes
GSMG0024984	---	up	1.66	1.52E-02	chr3(+):136683826-136686421	UCSC	---	AK084872	---	---	6.67	7.4	No
GSMG0001840	Trip12	up	1.65	1.75E-02	chr1(-):84721189-84840517	UCSC	thyroid hormone receptor interactor 12	NM_133975	14897	ENSMUSG00000026219	9.18	9.9	No

FAST DB STABLE ID	Gene Symbol	Regulation	Fold-Change	P-Value	Gene Coordinates (mm10)	UCSC Link	Gene Name	Representative Transcript ID	Entrez Gene ID	EnsEMBL ID	log^2 intensity of untreated	log^2 intensity of treated	Low-specificity probe included
GSMG0001541	Kdelc1	up	1.65	2.64E-02	chr1(-):44106546-44118802	UCSC	KDEL (Lys-Asp-Glu-Leu) containing 1	AK035899	72050	ENSMUSG00000026047	7.6	8.33	No
GSMG0012399	Psmb5	up	1.65	7.22E-03	chr14(-):54614120-54618018	UCSC	proteasome (prosome, macropain) subunit, beta type 5	AK133839	19173	ENSMUSG00000022193	8.83	9.55	No
GSMG0012291	---	up	1.65	3.65E-02	chr14(-):44546193-44549485	UCSC	---	AK004571	---	---	10.76	11.49	Yes
GSMG0011743	Kctd9	up	1.65	9.18E-03	chr14(+):67716095-67742310	UCSC	potassium channel tetramerisation domain containing 9	NM_134073	105440	ENSMUSG00000034327	8.28	9	No
GSMG0010685	4833439L19Rik	up	1.65	2.93E-02	chr13(-):54550812-54565382	UCSC	RIKEN cDNA 4833439L19 gene	AK145025	97820	ENSMUSG00000025871	9.26	9.98	No
GSMG0035844	9830147E19Rik	up	1.65	1.14E-02	chr7(-):42609526-42642604	UCSC	RIKEN cDNA 9830147E19 gene	NM_001242388	208111	ENSMUSG00000074158	5.85	6.57	Yes
GSMG0032890	---	up	1.65	1.28E-02	chr6(-):37425432-37429582	UCSC	---	AK143927	---	---	6.9	7.63	Yes
GSMG0031239	Mospd3	up	1.65	7.02E-04	chr5(-):137596649-137601044	UCSC	motile sperm domain containing 3	BC034167	68929	ENSMUSG00000037221	7.82	8.54	Yes
GSMG0029824	Zfp664	up	1.65	1.99E-02	chr5(+):124862692-124902693	UCSC	zinc finger protein 664	AK035022	269704	ENSMUSG00000079215	7.55	8.28	Yes
GSMG0026492	---	up	1.65	2.87E-02	chr4(+):44664414-44685347	UCSC	---	AK029677	---	---	6.78	7.5	Yes
GSMG0022198	Cebpb	up	1.64	4.62E-02	chr2(+):167688915-167690418	UCSC	CCAAT/enhancer binding protein (C/EBP), beta	X62600	12608	ENSMUSG00000056501	9.01	9.72	No
GSMG0021393	Arhgap1	up	1.64	4.74E-03	chr2(+):91650118-91672318	UCSC	Rho GTPase activating protein 1	NR_027373	228359	ENSMUSG00000027247	8.63	9.34	No
GSMG0020471	Arl3	up	1.64	2.40E-03	chr19(-):46531109-46573093	UCSC	ADP-ribosylation factor-like 3	AK048644	56350	ENSMUSG00000025035	8.41	9.13	No
GSMG0019400	Tmem258	up	1.64	1.44E-03	chr19(+):10204202-10207824	UCSC	transmembrane protein 258	NM_026919	69038	ENSMUSG00000036372	8.28	8.99	No
GSMG0017105	---	up	1.64	1.44E-02	chr17(-):10299777-10302221	UCSC	---	AK053557	---	---	7.84	8.56	Yes
GSMG0011230	3830406C13Rik	up	1.64	2.48E-02	chr14(+):12284209-12303231	UCSC	RIKEN cDNA 3830406C13 gene	NM_178141	218734	ENSMUSG00000033111	8.18	8.89	No
GSMG0009626	Bphl	up	1.64	1.21E-02	chr13(+):34037596-34074074	UCSC	biphenyl hydrolase-like (serine hydrolase, breast epithelial mucin-associated a	AK078232	68021	ENSMUSG00000038286	6.81	7.52	No
GSMG0000758	Rab7l1	up	1.64	1.39E-02	chr1(+):131867277-131872885	UCSC	RAB7, member RAS oncogene family-like 1	NM_144875	226422	ENSMUSG00000026433	8.11	8.83	No
GSMG0006819	Map2k4	up	1.64	4.68E-03	chr11(-):65688244-65788302	UCSC	mitogen-activated protein kinase kinase 4	AK036928	26398	ENSMUSG00000033352	9.02	9.73	No
GSMG0005209	Gpx3	up	1.64	6.80E-03	chr11(+):54902854-54910382	UCSC	glutathione peroxidase 3	NM_008161	14778	ENSMUSG00000018339	7.35	8.06	Yes
GSMG0039869	Sh3bgrl2	up	1.64	3.38E-02	chr9(+):83548325-83638801	UCSC	SH3 domain binding glutamic acid-rich protein like 2	AK032092	212531	ENSMUSG00000032261	7.07	7.78	Yes
GSMG0039022	Aprt	up	1.64	9.32E-03	chr8(-):122574637-122576911	UCSC	adenine phosphoribosyl transferase	NM_009698	11821	ENSMUSG00000006589	10.13	10.84	No
GSMG0037908	Cox4i1	up	1.64	3.73E-02	chr8(+):120668286-120674209	UCSC	cytochrome c oxidase subunit IV isoform 1	AK012583	12857	ENSMUSG00000031818	9.69	10.4	No
GSMG0029937	---	up	1.64	1.67E-02	chr5(+):137612852-137615247	UCSC	---	AK134703	---	---	7	7.71	Yes
GSMG0029602	---	up	1.64	1.30E-02	chr5(+):110896218-110898650	UCSC	---	AK034968	---	---	5.72	6.44	Yes
GSMG0028188	Rnf11	up	1.64	1.40E-02	chr4(-):109452857-109476983	UCSC	ring finger protein 11	AB024427	29864	ENSMUSG00000028557	8.55	9.26	No
GSMG0025184	Gm5150	up	1.64	4.61E-02	chr3(-):15948070-16006332	UCSC	predicted gene 5150	NM_001081687	381484	ENSMUSG00000078780	7.9	8.62	No
GSMG0024996	H2afz	up	1.64	1.00E-02	chr3(+):137864540-137866922	UCSC	H2A histone family, member Z	AK088704	51788	ENSMUSG00000037894	10.97	11.68	No
GSMG0024958	Ints12	up	1.64	3.66E-02	chr3(+):133091933-133110983	UCSC	integrator complex subunit 12	NM_027927	71793	ENSMUSG00000028016	9.44	10.15	No
GSMG0000027	Sgk3	up	1.64	5.94E-03	chr1(+):9798130-9902570	UCSC	serum/glucocorticoid regulated kinase 3	NM_177547	170755	ENSMUSG00000025915	8.96	9.67	Yes
GSMG0021766	---	up	1.63	1.14E-02	chr2(+):129082743-129084444	UCSC	---	AK134618	---	---	6.21	6.92	Yes
GSMG0017654	---	up	1.63	1.96E-02	chr17(-):51126711-51129160	UCSC	---	AK136422	---	---	6.63	7.33	No
GSMG0014050	---	up	1.63	1.44E-03	chr15(-):64255166-64257070	UCSC	---	AK089241	---	---	6.72	7.43	No
GSMG0011853	Zbtbd6	up	1.63	4.96E-02	chr14(+):79451840-79454700	UCSC	kelch repeat and BTB (POZ) domain containing 6	AK163295	432879	ENSMUSG00000075502	7.21	7.92	No
GSMG0011100	---	up	1.63	2.30E-02	chr13(-):107011621-107012737	UCSC	---	AK086649	---	---	7.63	8.33	No
GSMG0009759	Gm904	up	1.63	1.83E-02	chr13(+):50643228-50645838	UCSC	predicted gene 904	NM_001033770	380845	ENSMUSG00000096641	6.69	7.39	No
GSMG0005278	Med9	up	1.63	8.44E-03	chr11(+):59948214-59963306	UCSC	mediator of RNA polymerase II transcription, subunit 9 homolog (yeast)	NM_138675	192191	ENSMUSG00000061650	7.84	8.55	Yes
GSMG0005208	Hint1	up	1.63	2.73E-02	chr11(+):54866393-54870496	UCSC	histidine triad nucleotide binding protein 1	BC070415	15254	ENSMUSG00000020267	9.43	10.13	No
GSMG0004788	Bloc1s1	up	1.63	3.65E-02	chr10(-):128919914-128923525	UCSC	biogenesis of lysosome-related organelles complex-1, subunit 1	AK003035	14533	ENSMUSG00000090247	8.16	8.87	No
GSMG0035070	Nucb2	up	1.63	9.96E-03	chr7(+):116504369-116540586	UCSC	nucleobindin 2	NM_001130479	53322	ENSMUSG00000030659	9.09	9.79	No
GSMG0029927	Plod3	up	1.63	4.30E-02	chr5(+):136987019-136996646	UCSC	procollagen-lysine, 2-oxoglutarate 5-dioxygenase 3	NM_011962	26433	ENSMUSG00000004846	8.32	9.02	No
GSMG0028562	---	up	1.63	5.42E-03	chr4(-):139376549-139380380	UCSC	---	AK155929	---	---	6.49	7.19	Yes
GSMG0024181	Gpr160	up	1.63	4.57E-02	chr3(+):30855947-30897192	UCSC	G protein-coupled receptor 160	NM_027965	71862	ENSMUSG00000037661	7.04	7.74	Yes
GSMG0022985	Atp5g3	up	1.63	6.96E-03	chr2(-):73908449-73911303	UCSC	ATP synthase, H+ transporting, mitochondrial F0 complex, subunit C3 (subur	AK087955	228033	ENSMUSG00000018770	10.05	10.76	No
GSMG0022855	Ly75	up	1.63	2.94E-02	chr2(-):60292098-60383296	UCSC	lymphocyte antigen 75	U19271	17076	ENSMUSG00000026980	8.95	9.65	No
GSMG0002712	---	up	1.63	8.75E-04	chr10(+):10017379-10021406	UCSC	---	AK044056	---	---	9.14	9.84	Yes
GSMG0014603	Hmox2	up	1.62	1.12E-02	chr16(+):4726361-4766739	UCSC	heme oxygenase (decycling) 2	AK134536	15369	ENSMUSG00000004070	9.55	10.24	No
GSMG0051328	Gm17821	up	1.62	1.30E-02	chr12(-):67656742-67669271	UCSC	predicted gene, 17821	NR_033146	100316870	---	8.79	9.48	Yes
GSMG0008789	Pik3cg	up	1.62	3.40E-03	chr12(-):32173397-32208650	UCSC	phosphoinositide-3-kinase, catalytic, gamma polypeptide	NM_001146200	30955	ENSMUSG00000020573	10.48	11.18	No
GSMG0008122	Mgat2	up	1.62	6.82E-03	chr12(+):69184195-69186702	UCSC	mannoside acetylglucosaminyltransferase 2	AK159185	217664	ENSMUSG00000043998	10.1	10.79	No
GSMG0006597	Trim41	up	1.62	2.84E-03	chr11(-):48806404-48817391	UCSC	tripartite motif-containing 41	NM_145377	211007	ENSMUSG00000040365	8.53	9.22	Yes
GSMG0038633	Lsm6	up	1.62	3.69E-02	chr8(-):78804868-78821152	UCSC	LSM6 homolog, U6 small nuclear RNA associated (S. cerevisiae)	NM_030145	78651	ENSMUSG00000031683	8.53	9.22	Yes
GSMG0036575	Tmc7	up	1.62	2.33E-02	chr7(-):118535844-118584747	UCSC	transmembrane channel-like gene family 7	AK083189	209760	ENSMUSG00000042246	6.64	7.34	No
GSMG0032677	2810474O19Rik	up	1.62	1.14E-02	chr6(+):149309414-149335662	UCSC	RIKEN cDNA 2810474O19 gene	NM_026054	67246	ENSMUSG00000032712	10.23	10.93	No
GSMG0029860	2410018M08Rik	up	1.62	1.97E-02	chr5(+):129895722-129903623	UCSC	RIKEN cDNA 2410018M08 gene	NM_183088	71970	ENSMUSG00000034173	9.44	10.14	Yes
GSMG0028619	Pdpn	up	1.62	3.23E-02	chr4(-):143267422-143299564	UCSC	podoplanin	AK158855	14726	ENSMUSG00000028583	6.66	7.36	Yes
GSMG0026557	Smc2	up	1.62	2.88E-02	chr4(+):52439242-52488365	UCSC	structural maintenance of chromosomes 2	AK145341	14211	ENSMUSG00000028312	8.12	8.81	Yes
GSMG0053109	Hist2h2aa1 // Hist2h3c1	up	1.62	1.60E-02	chr3(+):96245536-96248509	UCSC	histone cluster 2, H2aa1 // histone cluster 2, H3c1	BC080809	15077 // 15267	USG00000063954 // ENSMUSG00000	10.29	10.99	Yes
GSMG0002655	---	up	1.61	2.02E-02	chr1(-):193237046-193239023	UCSC	---	AK047510	---	---	7.39	8.08	No
GSMG0019899	Grk5	up	1.61	4.60E-02	chr19(+):60889747-61092553	UCSC	G protein-coupled receptor kinase 5	NM_018869	14773	ENSMUSG00000003228	8.02	8.71	Yes
GSMG0010447	C530050E15Rik	up	1.61	1.39E-02	chr13(-):24631918-24638568	UCSC	RIKEN cDNA C530050E15 gene	AK049793	320489	ENSMUSG00000097838	6.76	7.45	Yes
GSMG0008725	Pqlc3	up	1.61	4.56E-02	chr12(-):16988648-17000118	UCSC	PQ loop repeat containing	NM_172574	217430	ENSMUSG00000045679	9.25	9.93	No
GSMG0006900	Rnasek // 0610010K14Rik	up	1.61	8.36E-03	chr11(-):70235205-70239852	UCSC	ribonuclease, RNase K // RIKEN cDNA 0610010K14 gene	BC012258	104457 // 52898	USG00000020831 // ENSMUSG00000	9.26	9.95	Yes
GSMG0042863	Morf4l2	up	1.61	2.00E-03	chrX(-):136732953-136743689	UCSC	mortality factor 4 like 2	AK161610	56397	ENSMUSG00000031422	9.27	9.95	No
GSMG0042026	Sh3bgrl	up	1.61	1.07E-02	chrX(+):109095407-109162470	UCSC	SH3-binding domain glutamic acid-rich protein like	NM_019989	56726	ENSMUSG00000031246	9.35	10.04	Yes
GSMG0041271	Mapkapk3	up	1.61	4.01E-02	chr9(-):107254927-107289878	UCSC	mitogen-activated protein kinase-activated protein kinase 3	AK170456	102626	ENSMUSG00000032577	8.58	9.26	No
GSMG0036317	Spcs2	up	1.61	1.45E-02	chr7(-):99837569-99858897	UCSC	signal peptidase complex subunit 2 homolog (S. cerevisiae)	AK134578	66624	ENSMUSG00000035227	10.25	10.94	No
GSMG0036216	---	up	1.61	2.05E-02	chr7(-):83178570-83200326	UCSC	---	AK020043	---	---	8.01	8.7	Yes
GSMG0033018	---	up	1.61	1.65E-02	chr6(-):51857425-51858871	UCSC	---	AK134567	---	---	7.12	7.8	Yes
GSMG0032967	---	up	1.61	2.15E-02	chr6(-):43162454-43172320	UCSC	---	AK006308	---	---	6.48	7.17	No
GSMG0032228	Slc6a6	up	1.61	4.08E-02	chr6(+):91684067-91759063	UCSC	solute carrier family 6 (neurotransmitter transporter, taurine), member 6	NM_009320	21366	ENSMUSG00000030096	9.91	10.6	No
GSMG0028306	Zmpste24	up	1.61	4.07E-02	chr4(-):121059238-121098243	UCSC	zinc metallopeptidase, STE24	NM_172700	230709	ENSMUSG00000043207	10.02	10.7	No
GSMG0026335	---	up	1.61	8.12E-03	chr4(+):21849192-21852263	UCSC	---	AK045855	---	---	8.7	9.39	No
GSMG0025985	---	up	1.61	8.44E-03	chr3(-):119735463-119737515	UCSC	---	AK084691	---	---	8.41	9.1	No
GSMG0025750	Mrps21	up	1.61	2.94E-03	chr3(-):95862648-95871499	UCSC	mitochondrial ribosomal protein S21	AK008265	66292	ENSMUSG00000054312	9.28	9.97	No
GSMG0025721	Psmb4	up	1.61	7.72E-03	chr3(-):94884093-94886958	UCSC	proteasome (prosome, macropain) subunit, beta type 4	AK137044	19172	ENSMUSG00000005779	9.67	10.35	No
GSMG0021537	Ccdc34	up	1.6	3.75E-02	chr2(+):110017816-110173360	UCSC	coiled-coil domain containing 34	AK014021	68201	ENSMUSG00000027160	7.6	8.28	No
GSMG0017540	Crisp3	up	1.6	1.37E-02	chr17(-):40221779-40242267	UCSC	cysteine-rich secretory protein 3	M92850	11572	ENSMUSG00000025433	6.28	6.96	Yes
GSMG0052862	Dynlt1c	up	1.6	1.46E-02	chr17(+):6601778-6607891	UCSC	dynein light chain Tctex-type 1C	AK168478	100040563	ENSMUSG00000000579	11.07	11.75	Yes
GSMG0014291	Desi1	up	1.6	4.06E-02	chr15(-):81992523-82016227	UCSC	desumoylating isopeptidase 1	BC022097	28075	ENSMUSG00000022472	8.52	9.2	No
GSMG0009930	Mrpl36	up	1.6	3.94E-03	chr13(+):73331008-73332183	UCSC	mitochondrial ribosomal protein L36	NM_053163	94066	ENSMUSG00000021607	8.11	8.79	No
GSMG0008953	Sos2	up	1.6	2.78E-02	chr12(-):69583761-69681852	UCSC	son of sevenless homolog 2 (Drosophila)	NM_001135559	20663	ENSMUSG00000034801	8.81	9.48	No
GSMG0051222	Gm12171	up	1.6	1.72E-02	chr11(+):46548413-46556068	UCSC	predicted gene 12171	NM_001163353	675689	ENSMUSG00000086981	6.41	7.09	Yes
GSMG0042264	Ap1s2	up	1.6	2.58E-02	chrX(+):163909015-163933666	UCSC	adaptor-related protein complex 1, sigma 2 subunit	AK045558	108012	ENSMUSG00000031367	8.58	9.25	No
GSMG0050843	Xpot	up	1.6	3.05E-02	chr10(-):121587380-121626316	UCSC	exportin, tRNA (nuclear export receptor for tRNAs)	NM_001081056	73192	ENSMUSG00000034667	9.89	10.56	No
GSMG0002686	---	up	1.6	1.35E-02	chr10(-):5023497-5029003	UCSC	---	AK039175	---	---	7.44	8.12	No
GSMG0030043	Bud31	up	1.6	1.72E-03	chr5(+):145140397-145148074	UCSC	BUD31 homolog (yeast)	NM_001008705	231889	ENSMUSG00000038722	8	8.68	No
GSMG0043299	---	up	1.6	1.12E-03	chr2(+):80838455-80838753	UCSC	---	AK206594	---	---	11.57	12.24	Yes
GSMG0027207	Pafah2	up	1.6	2.76E-02	chr4(+):134396360-134427410	UCSC	platelet-activating factor acetylhydrolase 2	NM_133880	100163	ENSMUSG00000037366	6.84	7.51	Yes
GSMG0025873	I830077J02Rik	up	1.6	4.70E-02	chr3(-):105925891-105932668	UCSC	RIKEN cDNA I830077J02 gene	AK152510	433638	ENSMUSG00000074342	9.02	9.69	No
GSMG0024831	Vav3	up	1.6	2.67E-02	chr3(+):109340375-109685693	UCSC	vav 3 oncogene	NM_020505	57257	ENSMUSG00000033721	8.16	8.83	No
GSMG0022050	Romo1	up	1.59	9.66E-03	chr2(+):156144153-156145793	UCSC	reactive oxygen species modulator 1	NM_001164217	67067	ENSMUSG00000067847	7.92	8.59	No
GSMG0021035	---	up	1.59	6.08E-03	chr2(+):48961956-48967092	UCSC	---	AK154730	---	---	6.47	7.14	No
GSMG0016078	Atp5o	up	1.59	4.73E-02	chr16(-):91925223-91931630	UCSC	ATP synthase, H+ transporting, mitochondrial F1 complex, O subunit	NM_138597	28080	ENSMUSG00000022956	9.4	10.07	No
GSMG0013301	Cyc1	up	1.59	1.01E-02	chr15(+):76343521-76345934	UCSC	cytochrome c-1	AK011288	66445	ENSMUSG00000022551	9.71	10.38	No
GSMG0012468	N6amt2	up	1.59	1.47E-02	chr14(-):57549597-57571571	UCSC	N-6 adenine-specific DNA methyltransferase 2 (putative)	AK012083	68043	ENSMUSG00000021951	6.92	7.59	No
GSMG0008579	Apopt1	up	1.59	9.23E-04	chr12(+):111713268-111755055	UCSC	apoptogenic, mitochondrial 1	NM_001163388	68020	ENSMUSG00000037787	6.71	7.38	No
GSMG0007628	Sumo2	up	1.59	1.81E-02	chr11(-):115521431-115536250	UCSC	SMT3 suppressor of mif two 3 homolog 2 (yeast)	AK132213	170930	ENSMUSG00000020738	9.7	10.36	Yes
GSMG0005794	---	up	1.59	2.27E-02	chr11(+):94741780-94743033	UCSC	---	AK136078	---	---	7.88	8.55	Yes
GSMG0000489	Sp140	up	1.59	2.00E-02	chr1(+):85600703-85645037	UCSC	Sp140 nuclear body protein	NM_001013817	434484	ENSMUSG00000070031	9.72	10.39	No
GSMG0042199	---	up	1.59	3.73E-02	chrX(+):151614966-151618160	UCSC	---	AK079482	---	---	5.58	6.25	Yes
GSMG0040570	Chek1	up	1.59	2.42E-02	chr9(-):36708482-36726660	UCSC	checkpoint kinase 1	NM_007691	12649	ENSMUSG00000032113	8.04	8.71	Yes
GSMG0036433	Prkcdbp	up	1.59	5.58E-03	chr7(-):105480607-105482197	UCSC	protein kinase C, delta binding protein	BC009660	109042	ENSMUSG00000037060	8.5	9.16	Yes
GSMG0004035	---	up	1.59	4.38E-02	chr10(-):42697837-42699088	UCSC	---	AK037814	---	---	6.86	7.53	No
GSMG0035113	Mettl9	up	1.59	4.67E-02	chr7(+):121034445-121076834	UCSC	methyltransferase like 9	NM_021554	59052	ENSMUSG00000030876	10.92	11.59	No
GSMG0033308	Snrnp27	up	1.59	3.39E-02	chr6(-):86675169-86684504	UCSC	small nuclear ribonucleoprotein 27 (U4/U6.U5)	AK011937	66618	ENSMUSG00000001158	7.64	8.31	No
GSMG0030342	---	up	1.59	2.71E-02	chr5(-):32390073-32393306	UCSC	---	AK145172	---	---	6.96	7.63	Yes
GSMG0029021	Mrpl33	up	1.59	4.78E-03	chr5(+):31596934-31664364	UCSC	mitochondrial ribosomal protein L33	AK038023	100503002	ENSMUSG00000029142	8.48	9.15	No
GSMG0028781	---	up	1.59	1.04E-02	chr5(+):3525430-3530998	UCSC	---	AK030810	---	---	7.92	8.59	No
GSMG0003209	Cstb	up	1.59	1.59E-02	chr10(+):78425670-78427621	UCSC	cystatin B	NM_007793	13014	ENSMUSG00000005054	8.36	9.04	No
GSMG0027473	2610002J02Rik	up	1.59	2.78E-04	chr4(+):155249964-155256687	UCSC	RIKEN cDNA 2610002J02 gene	NR_033802	67513	ENSMUSG00000073684	7.66	8.33	No
GSMG0003160	Gm5779	up	1.59	1.52E-02	chr10(+):75344464-75353031	UCSC	predicted gene 5779	BC082775	544707	---	7.42	8.09	Yes
GSMG0019904	---	up	1.58	6.78E-03	chr19(+):61092891-61095966	UCSC	---	BC058625	---	---	7.11	7.77	No
GSMG0019691	Pgam1	up	1.58	4.33E-02	chr19(+):41911871-41918665	UCSC	phosphoglycerate mutase 1	NM_023418	18648	ENSMUSG00000011752	10.58	11.24	No
GSMG0015507	Med15	up	1.58	4.37E-04	chr16(-):17651208-17722949	UCSC	mediator complex subunit 15	AK169904	94112	ENSMUSG00000012114	9.58	10.24	Yes
GSMG0001576	Asnsd1	up	1.58	4.64E-03	chr1(-):53325507-53352754	UCSC	asparagine synthetase domain containing 1	AK034746	70396	ENSMUSG00000026095	9.83	10.5	No
GSMG0013096	---	up	1.58	6.68E-03	chr15(+):35478825-35481562	UCSC	---	AK079692	---	---	7.27	7.93	No
GSMG0012628	---	up	1.58	4.66E-02	chr14(-):70759690-70761292	UCSC	---	AK134498	---	---	7.4	8.06	Yes
GSMG0007282	Atp5g1	up	1.58	2.38E-02	chr11(-):96072793-96075694	UCSC	ATP synthase, H+ transporting, mitochondrial F0 complex, subunit c1 (subun	NM_007506	11951	ENSMUSG00000006057	8.63	9.29	No
GSMG0005131	Rnf130	up	1.58	4.62E-03	chr11(+):50025331-50121008	UCSC	ring finger protein 130	AK166468	59044	ENSMUSG00000020376	10.45	11.11	Yes
GSMG0042730	Rps4x	up	1.58	4.79E-02	chrX(-):102184943-102189326	UCSC	ribosomal protein S4, X-linked	AK135369	20102	ENSMUSG00000031320	11.01	11.67	Yes
GSMG0042702	Ophn1	up	1.58	4.77E-04	chrX(-):98557515-98891026	UCSC	oligophrenin 1	AK036038	94190	ENSMUSG00000031214	5.92	6.58	Yes
GSMG0042646	Gyk	up	1.58	2.81E-02	chrX(-):85701937-85776820	UCSC	glycerol kinase	NM_212444	14933	ENSMUSG00000025059	7.2	7.85	Yes
GSMG0042006	Cox7b	up	1.58	7.38E-03	chrX(+):106015700-106022448	UCSC	cytochrome c oxidase subunit VIIb	NM_025379	66142	ENSMUSG00000031231	10.05	10.71	No
GSMG0040485	Dpy19l1	up	1.58	2.48E-02	chr9(-):24411799-24503140	UCSC	dpy-19-like 1 (C. elegans)	NM_172920	244745	ENSMUSG00000043067	7.2	7.86	Yes
GSMG0040257	Ackr2	up	1.58	2.54E-02	chr9(+):121898354-121911071	UCSC	atypical chemokine receptor 2	NM_021609	59289	ENSMUSG00000044534	7.82	8.49	No
GSMG0038776	Amfr	up	1.58	8.94E-03	chr8(-):93971589-94012843	UCSC	autocrine motility factor receptor	NM_011787	23802	ENSMUSG00000031751	9.06	9.72	No
GSMG0004141	Sgpl1	up	1.58	3.42E-02	chr10(-):61098644-61147665	UCSC	sphingosine phosphate lyase 1	NM_009163	20397	ENSMUSG00000020097	9.79	10.45	No
GSMG0033156	Rmnd5a	up	1.58	8.40E-03	chr6(-):71388634-71440638	UCSC	required for meiotic nuclear division 5 homolog A (S. cerevisiae)	NM_024288	68477	ENSMUSG00000002222	9.17	9.83	No
GSMG0033075	Nt5c3	up	1.58	3.79E-02	chr6(-):56882404-56923932	UCSC	5'-nucleotidase, cytosolic III	NM_026004	107569	ENSMUSG00000029780	8.64	9.3	Yes
GSMG0025020	Tspan5	up	1.58	2.02E-02	chr3(+):138742195-138904425	UCSC	tetraspanin 5	NM_019571	56224	ENSMUSG00000028152	8.47	9.13	Yes
GSMG0024521	Ssr2	up	1.58	3.51E-02	chr3(+):88579607-88588419	UCSC	signal sequence receptor, beta	AK005340	66256	ENSMUSG00000041355	9.97	10.63	Yes
GSMG0024441	Ppid	up	1.58	9.46E-03	chr3(+):79591340-79603650	UCSC	peptidylprolyl isomerase D (cyclophilin D)	AK151091	67738	ENSMUSG00000027804	10.14	10.8	No
GSMG0018479	Apcdd1	up	1.57	3.94E-03	chr18(+):62922322-62953812	UCSC	adenomatosis polyposis coli down-regulated 1	AK172004	494504	ENSMUSG00000071847	7.92	8.58	Yes
GSMG0002069	Il24	up	1.57	2.84E-02	chr1(-):130882074-130887411	UCSC	interleukin 24	NM_053095	93672	ENSMUSG00000026420	6.97	7.62	Yes
GSMG0017687	Sema6b	up	1.57	1.59E-02	chr17(-):56123085-56140345	UCSC	sema domain, transmembrane domain (TM), and cytoplasmic domain, (sema	AK143229	20359	ENSMUSG00000001227	7.7	8.35	Yes
GSMG0001063	Igsf8	up	1.57	2.83E-02	chr1(+):172261631-172319848	UCSC	immunoglobulin superfamily, member 8	BC060087	140559	ENSMUSG00000038034	7.48	8.14	No
GSMG0041669	Ube2a	up	1.57	9.28E-03	chrX(+):36874330-36884218	UCSC	ubiquitin-conjugating enzyme E2A	AK133723	22209	ENSMUSG00000016308	8.37	9.02	No
GSMG0041106	Bcl2a1d	up	1.57	2.64E-02	chr9(-):88723288-88731873	UCSC	B cell leukemia/lymphoma 2 related protein A1d	BC053965	12047	ENSMUSG00000096581	8.66	9.32	Yes
GSMG0039371	Vwa5a	up	1.57	2.12E-02	chr9(+):38718268-38743337	UCSC	von Willebrand factor A domain containing 5A	NM_172767	67776	ENSMUSG00000023186	8.88	9.53	No
GSMG0038297	Lonrf1	up	1.57	8.94E-03	chr8(-):36216064-36249516	UCSC	LON peptidase N-terminal domain and ring finger 1	NM_001081150	244421	ENSMUSG00000039633	6.99	7.64	No
GSMG0035601	Bcl3	up	1.57	2.56E-02	chr7(-):19808462-19822755	UCSC	B cell leukemia/lymphoma 3	NM_033601	12051	ENSMUSG00000053175	8.68	9.33	No
GSMG0035355	Rplp2	up	1.57	9.63E-04	chr7(+):141447643-141451585	UCSC	ribosomal protein, large P2	AK012402	67186	ENSMUSG00000025508	10.98	11.63	Yes
GSMG0031489	---	up	1.57	3.25E-02	chr6(+):12041517-12047422	UCSC	---	AK045418	---	---	6.79	7.45	No
GSMG0029285	Dancr	up	1.57	3.93E-02	chr5(+):74093083-74094336	UCSC	differentiation antagonizing non-protein coding RNA	NR_015531	70036	ENSMUSG00000053656	8.48	9.13	No
GSMG0022780	---	up	1.57	4.98E-02	chr2(-):45053788-45057514	UCSC	---	AK052096	---	---	7.09	7.74	Yes
GSMG0021804	Atrn	up	1.56	1.03E-02	chr2(+):130906491-131030329	UCSC	attractin	NM_009730	11990	ENSMUSG00000027312	8.71	9.35	No
GSMG0020960	Dab2ip	up	1.56	2.66E-02	chr2(+):35558458-35730994	UCSC	disabled 2 interacting protein	NM_001114125	69601	ENSMUSG00000026883	7.51	8.15	No
GSMG0018946	Ticam2	up	1.56	3.34E-02	chr18(-):46558231-46574534	UCSC	toll-like receptor adaptor molecule 2	NM_173394	225471	ENSMUSG00000056130	7.64	8.28	No
GSMG0017876	Cdc42ep3	up	1.56	9.26E-03	chr17(-):79333727-79355094	UCSC	CDC42 effector protein (Rho GTPase binding) 3	AK150214	260409	ENSMUSG00000036533	9.47	10.11	No
GSMG0017220	1600002H07Rik	up	1.56	3.96E-03	chr17(-):24215058-24220851	UCSC	RIKEN cDNA 1600002H07 gene	NM_028056	72016	ENSMUSG00000024118	6.98	7.62	Yes
GSMG0015725	BC031361	up	1.56	4.12E-02	chr16(-):38084506-38089261	UCSC	cDNA sequence BC031361	AK145079	414072	---	6.17	6.81	Yes
GSMG0015043	---	up	1.56	1.35E-02	chr16(+):43494037-43496840	UCSC	---	AK143750	---	---	7.27	7.91	No
GSMG0014971	---	up	1.56	3.09E-02	chr16(+):38451981-38453493	UCSC	---	AK086460	---	---	8.79	9.42	Yes
GSMG0006045	Helz	up	1.56	5.52E-03	chr11(+):107547956-107687820	UCSC	helicase with zinc finger domain	NM_198298	78455	ENSMUSG00000020721	8.11	8.75	No
GSMG0005437	Arrb2	up	1.56	4.89E-02	chr11(+):70432580-70440828	UCSC	arrestin, beta 2	NM_145429	216869	ENSMUSG00000060216	10.29	10.92	No
GSMG0042533	Mmgt1	up	1.56	1.90E-02	chrX(-):56585512-56598069	UCSC	membrane magnesium transporter 1	BC032271	236792	ENSMUSG00000061273	8.58	9.22	No
GSMG0004907	C1d	up	1.56	1.11E-02	chr11(+):17257591-17269176	UCSC	C1D nuclear receptor co-repressor	AK035169	57316	ENSMUSG00000000581	8.41	9.05	No
GSMG0051193	Gm8580	up	1.56	4.36E-02	chr10(-):93665266-93665854	UCSC	predicted gene 8580	NR_027478	667338	ENSMUSG00000090159	9.68	10.32	No
GSMG0038011	A630001O12Rik	up	1.56	7.50E-03	chr8(+):126839231-126851833	UCSC	RIKEN cDNA A630001O12 gene	AK172442	414091	---	6.82	7.46	Yes
GSMG0037299	Hmgb2	up	1.56	2.38E-02	chr8(+):57511843-57515999	UCSC	high mobility group box 2	NM_008252	97165	ENSMUSG00000054717	11.55	12.19	No
GSMG0035757	Tmem147	up	1.56	2.02E-02	chr7(-):30727701-30729536	UCSC	transmembrane protein 147	NM_027215	69804	ENSMUSG00000006315	7.39	8.04	No
GSMG0003760	Pcmt1	up	1.56	2.54E-02	chr10(-):7629374-7663609	UCSC	protein-L-isoaspartate (D-aspartate) O-methyltransferase 1	BC040750	18537	ENSMUSG00000019795	9.43	10.07	No
GSMG0032644	Tuba3b	up	1.56	2.60E-02	chr6(+):145615963-145621481	UCSC	tubulin, alpha 3B	NM_009449	22147	ENSMUSG00000067338	9.02	9.66	Yes
GSMG0031997	---	up	1.56	2.37E-02	chr6(+):63816811-63818086	UCSC	---	AK036344	---	---	5.44	6.07	Yes
GSMG0030547	---	up	1.56	3.00E-02	chr5(-):65605723-65609380	UCSC	---	AK083707	---	---	7.93	8.57	Yes
GSMG0030374	Mfsd10	up	1.56	1.38E-02	chr5(-):34633646-34637184	UCSC	major facilitator superfamily domain containing 10	BC064775	68294	ENSMUSG00000001082	9.27	9.91	No
GSMG0030324	Eif2b4	up	1.56	1.32E-02	chr5(-):31187559-31193139	UCSC	eukaryotic translation initiation factor 2B, subunit 4 delta	NM_010122	13667	ENSMUSG00000029145	7.56	8.21	No
GSMG0030276	---	up	1.56	4.54E-03	chr5(-):25436538-25438246	UCSC	---	AK046589	---	---	7.54	8.19	No
GSMG0028883	Tmem60	up	1.56	3.52E-02	chr5(+):20882453-20886869	UCSC	transmembrane protein 60	NM_177601	212090	ENSMUSG00000045435	7.12	7.77	No
GSMG0003019	Lims1	up	1.56	2.88E-03	chr10(+):58323464-58424691	UCSC	LIM and senescent cell antigen-like domains 1	NM_026148	110829	ENSMUSG00000019920	10.3	10.94	No
GSMG0025521	---	up	1.56	2.26E-02	chr3(-):75538150-75540454	UCSC	---	AK138166	---	---	6.69	7.33	Yes
GSMG0002210	BC003331	up	1.55	1.01E-02	chr1(-):150361313-150393055	UCSC	cDNA sequence BC003331	NM_145511	226499	ENSMUSG00000006010	8.25	8.89	Yes
GSMG0018071	Riok3	up	1.55	7.82E-03	chr18(+):12128754-12157367	UCSC	RIO kinase 3	AK147074	66878	ENSMUSG00000024404	9.45	10.08	No
GSMG0000211	Bivm	up	1.55	3.50E-03	chr1(+):44119162-44144771	UCSC	basic, immunoglobulin-like variable motif containing	AF411386	246229	ENSMUSG00000041684	6.84	7.47	Yes
GSMG0016341	Pgp	up	1.55	2.17E-02	chr17(+):24470473-24471603	UCSC	phosphoglycolate phosphatase	NM_025954	67078	ENSMUSG00000043445	8.27	8.91	Yes
GSMG0014959	Lrrc58	up	1.55	4.38E-03	chr16(+):37868389-37888857	UCSC	leucine rich repeat containing 58	NM_177093	320184	ENSMUSG00000034158	8.98	9.61	Yes
GSMG0012421	Psme2	up	1.55	4.86E-03	chr14(-):55587440-55591101	UCSC	proteasome (prosome, macropain) activator subunit 2 (PA28 beta)	NM_001029855	19188	ENSMUSG00000079197	10.65	11.29	No
GSMG0007394	Dnajc7	up	1.55	4.36E-02	chr11(-):100582819-100620168	UCSC	DnaJ (Hsp40) homolog, subfamily C, member 7	NM_019795	56354	ENSMUSG00000014195	10.72	11.36	No
GSMG0006095	Slc9a3r1	up	1.55	2.13E-02	chr11(+):115163340-115181179	UCSC	solute carrier family 9 (sodium/hydrogen exchanger), member 3 regulator 1	AK168894	26941	ENSMUSG00000020733	9.86	10.49	Yes
GSMG0005754	Trim25	up	1.55	5.26E-03	chr11(+):88999402-89020293	UCSC	tripartite motif-containing 25	NM_009546	217069	ENSMUSG00000000275	8.45	9.08	No
GSMG0005050	Nudcd2	up	1.55	4.31E-02	chr11(+):40733642-40740044	UCSC	NudC domain containing 2	NM_026023	52653	ENSMUSG00000020328	8.57	9.2	No
GSMG0004916	Spred2	up	1.55	6.28E-03	chr11(+):19924372-20023926	UCSC	sprouty-related, EVH1 domain containing 2	AK157817	114716	ENSMUSG00000045671	7.95	8.58	No
GSMG0042209	Kdm5c	up	1.55	9.70E-03	chrX(+):152233227-152274354	UCSC	lysine (K)-specific demethylase 5C	NM_013668	20591	ENSMUSG00000025332	10.35	10.98	No
GSMG0041606	---	up	1.55	4.72E-02	chrX(+):13076229-13080062	UCSC	---	AK083509	---	---	8.89	9.52	No
GSMG0017871	Gm6548	up	1.55	1.64E-02	chr17(-):78850507-78852544	UCSC	predicted gene 6548	NR_003363	625054	ENSMUSG00000091549	8.58	9.21	No

FAST DB STABLE ID	Gene Symbol	Regulation	Fold-Change	P-Value	Gene Coordinates (mm10)	UCSC Link	Gene Name	Representative Transcript ID	Entrez Gene ID	EnsEMBL ID	log^2 intensity of untreated	log^2 intensity of treated	Low-specificity probe included
GSMG0036618	Ndufab1	up	1.55	3.30E-02	chr7(-):122086836-122101851	UCSC	NADH dehydrogenase (ubiquinone) 1, alpha/beta subcomplex, 1	AK008788	70316	ENSMUSG00000030869	8.15	8.78	Yes
GSMG0032220	---	up	1.55	3.15E-02	chr6(+):90905699-90906507	UCSC	---	AK077194	---	---	9.12	9.76	Yes
GSMG0003430	Ccdc41	up	1.55	2.39E-02	chr10(+):94688653-94790336	UCSC	coiled-coil domain containing 41	NM_029852	77048	ENSMUSG00000020024	7.87	8.5	No
GSMG0027093	Gm12942	up	1.55	2.65E-02	chr4(+):127126043-127129660	UCSC	predicted gene 12942	NM_001099319	100039968	ENSMUSG00000070737	7.39	8.02	No
GSMG0026652	Tlr4	up	1.55	4.10E-03	chr4(+):66827599-66930284	UCSC	toll-like receptor 4	AK014533	21898	ENSMUSG00000039005	7.9	8.53	No
GSMG0024390	Rsrc1	up	1.55	3.59E-02	chr3(+):67105470-67108774	UCSC	arginine/serine-rich coiled-coil 1	AK044911	66880	ENSMUSG00000034544	6.04	6.67	No
GSMG0023738	Pxmp4	up	1.55	4.80E-02	chr2(-):154585760-154603689	UCSC	peroxisomal membrane protein 4	AK156743	59038	ENSMUSG00000000876	7.84	8.47	Yes
GSMG0023379	Srp14	up	1.55	2.20E-02	chr2(-):118473752-118479696	UCSC	signal recognition particle 14	AK156845	20813	ENSMUSG00000009549	8.92	9.56	No
GSMG0021303	Zc3h15	up	1.54	1.61E-02	chr2(+):83644480-83664617	UCSC	zinc finger CCCH-type containing 15	AK167696	69082	ENSMUSG00000027091	9.29	9.92	No
GSMG0017923	Zfp36l2	up	1.54	8.74E-03	chr17(-):84183924-84187948	UCSC	zinc finger protein 36, C3H type-like 2	NM_001001806	12193	ENSMUSG00000045817	11.05	11.66	No
GSMG0001940	Pam	up	1.54	3.89E-02	chr1(-):97821095-98095646	UCSC	peptidylglycine alpha-amidating monooxygenase	NM_013626	18484	ENSMUSG00000026335	6.92	7.54	No
GSMG0010985	Polk	up	1.54	1.83E-02	chr13(-):96480689-96542580	UCSC	polymerase (DNA directed), kappa	BC052820	27015	ENSMUSG00000021668	7.41	8.04	No
GSMG0010728	Hnrnpa0	up	1.54	1.10E-03	chr13(-):58125879-58128556	UCSC	heterogeneous nuclear ribonucleoprotein A0	NM_029872	77134	ENSMUSG00000007836	9.82	10.45	No
GSMG0009728	Rnf144b	up	1.54	1.27E-02	chr13(+):47122720-47247991	UCSC	ring finger protein 144B	NM_146042	218215	ENSMUSG00000038068	7.17	7.79	No
GSMG0000656	D630008O14Rik	up	1.54	8.64E-03	chr1(+):106541943-106575045	UCSC	RIKEN cDNA D630008O14 gene	AK085305	320761	ENSMUSG00000054006	6.47	7.1	Yes
GSMG0006275	---	up	1.54	4.74E-03	chr11(-):4590456-4593808	UCSC	---	AK052072	---	---	7.39	8.02	Yes
GSMG0005931	Mlx	up	1.54	3.27E-02	chr11(+):101086710-101092212	UCSC	MAX-like protein X	BC080768	21428	ENSMUSG00000017801	9.2	9.82	Yes
GSMG0005895	Cdc6	up	1.54	4.11E-02	chr11(+):98907808-98923942	UCSC	cell division cycle 6	AK162669	23834	ENSMUSG00000017499	8.76	9.39	No
GSMG0042436	Lamp2	up	1.54	1.65E-02	chrX(-):38401359-38456461	UCSC	lysosomal-associated membrane protein 2	AK078193	16784	ENSMUSG00000016534	10.25	10.88	Yes
GSMG0038737	Brd7	up	1.54	1.17E-02	chr8(-):88331059-88362195	UCSC	bromodomain containing 7	NM_012047	26992	ENSMUSG00000031660	8.19	8.81	No
GSMG0038317	---	up	1.54	7.36E-03	chr8(-):37088936-37090310	UCSC	---	AK133068	---	---	7	7.62	Yes
GSMG0034390	Etfb	up	1.54	2.23E-02	chr7(+):43444072-43457799	UCSC	electron transferring flavoprotein, beta polypeptide	NR_075104	110826	ENSMUSG00000004610	8.53	9.15	No
GSMG0032910	Hipk2	up	1.54	3.36E-02	chr6(-):38697840-38876190	UCSC	homeodomain interacting protein kinase 2	NM_010433	15258	ENSMUSG00000061436	9.08	9.7	No
GSMG0031411	N4bp2l1	up	1.54	4.79E-02	chr5(-):150571643-150594530	UCSC	NEDD4 binding protein 2-like 1	NM_133898	100637	ENSMUSG00000041132	8.04	8.66	No
GSMG0031336	Ccz1	up	1.54	3.20E-02	chr5(-):143987908-144014891	UCSC	CCZ1 vacuolar protein trafficking and biogenesis associated	AK153160	231874	ENSMUSG00000029617	8.21	8.83	Yes
GSMG0002887	Tube1	up	1.54	1.21E-02	chr10(+):39134023-39152544	UCSC	epsilon-tubulin 1	NM_028006	71924	ENSMUSG00000019845	7.05	7.67	Yes
GSMG0021647	Snap23	up	1.53	3.91E-02	chr2(+):120567670-120600722	UCSC	synaptosomal-associated protein 23	AK019162	20619	ENSMUSG00000027287	8.77	9.38	Yes
GSMG0002382	Pou2f1	up	1.53	4.72E-03	chr1(-):165865154-166002637	UCSC	POU domain, class 2, transcription factor 1	NM_198932	18986	ENSMUSG00000026565	9.69	10.3	No
GSMG0019394	Fth1	up	1.53	3.41E-02	chr19(+):9980598-9985111	UCSC	ferritin heavy chain 1	NR_073181	14319	ENSMUSG00000024661	12.18	12.79	Yes
GSMG0018733	---	up	1.53	1.83E-02	chr18(-):15066879-15070503	UCSC	---	AK140481	---	---	6.13	6.75	Yes
GSMG0001805	Dock10	up	1.53	2.08E-02	chr1(-):80501073-80758554	UCSC	dedicator of cytokinesis 10	NM_175291	210293	ENSMUSG00000038608	9.84	10.45	No
GSMG0014887	Tctex1d2	up	1.53	3.44E-03	chr16(+):32419700-32429098	UCSC	Tctex1 domain containing 2	AK002589	66061	ENSMUSG00000014075	8.38	8.99	No
GSMG0012007	1700024B18Rik	up	1.53	1.52E-02	chr14(+):123987634-124015684	UCSC	RIKEN cDNA 1700024B18 gene	AK006293	69393	---	7.18	7.79	No
GSMG0000168	Pdcl3	up	1.53	2.32E-03	chr1(+):38987772-38997236	UCSC	phosducin-like 3	AK078372	68833	ENSMUSG00000026078	8.31	8.93	No
GSMG0010912	---	up	1.53	4.49E-02	chr13(-):83663950-83667663	UCSC	---	AK156916	---	---	7.99	8.61	Yes
GSMG0007019	---	up	1.53	3.45E-02	chr11(-):77057224-77059399	UCSC	---	AK083976	---	---	6.3	6.92	Yes
GSMG0005848	Copz2	up	1.53	2.39E-02	chr11(+):96849869-96861203	UCSC	coatomer protein complex, subunit zeta 2	AB047850	56358	ENSMUSG00000018672	7	7.61	No
GSMG0041816	Hmgb3	up	1.53	2.34E-02	chrX(+):71555918-71560673	UCSC	high mobility group box 3	AK141399	15354	ENSMUSG00000015217	7.57	8.18	No
GSMG0040978	Aqp9	up	1.53	2.81E-02	chr9(-):71110659-71163289	UCSC	aquaporin 9	NR_073483	64008	ENSMUSG00000032204	7.5	8.11	No
GSMG0040656	Trappc4	up	1.53	2.12E-03	chr9(-):44403759-44407548	UCSC	trafficking protein particle complex 4	NM_021789	60409	ENSMUSG00000032112	8.23	8.85	Yes
GSMG0040049	Cish	up	1.53	1.26E-02	chr9(+):107296689-107302784	UCSC	cytokine inducible SH2-containing protein	AK086040	12700	ENSMUSG00000032578	8.29	8.9	No
GSMG0039023	Galns	up	1.53	6.08E-03	chr8(-):122578237-122611487	UCSC	galactosamine (N-acetyl)-6-sulfate sulfatase	NM_016722	50917	ENSMUSG00000015027	8.73	9.35	No
GSMG0038660	Tecr	up	1.53	2.15E-02	chr8(-):83571700-83594491	UCSC	trans-2,3-enoyl-CoA reductase	NM_134118	106529	ENSMUSG00000031708	10.63	11.24	No
GSMG0038614	---	up	1.53	2.33E-02	chr8(-):73240142-73243347	UCSC	---	AK148676	---	---	6.66	7.28	No
GSMG0038366	Ankrd37	up	1.53	2.75E-02	chr8(-):45996910-45999868	UCSC	ankyrin repeat domain 37	AK020869	654824	ENSMUSG00000050914	7	7.61	No
GSMG0036577	Gde1	up	1.53	2.90E-03	chr7(-):118688558-118705760	UCSC	glycerophosphodiester phosphodiesterase 1	AK150807	56209	ENSMUSG00000033917	9.03	9.64	No
GSMG0036549	Psma1	up	1.53	2.80E-03	chr7(-):114264550-114276116	UCSC	proteasome (prosome, macropain) subunit, alpha type 1	NM_011965	26440	ENSMUSG00000030751	10.37	10.98	No
GSMG0036512	---	up	1.53	1.52E-02	chr7(-):110277782-110281528	UCSC	---	AK041454	---	---	7.83	8.45	Yes
GSMG0033280	Sfxn5	up	1.53	1.77E-02	chr6(-):85213051-85333438	UCSC	sideroflexin 5	NM_178639	94282	ENSMUSG00000033720	8.08	8.69	No
GSMG0032367	Zfand4	up	1.53	4.53E-02	chr6(+):116264220-116330301	UCSC	zinc finger, AN1-type domain 4	AK089032	67492	ENSMUSG00000042213	6.67	7.28	No
GSMG0031683	1810058I24Rik	up	1.53	1.20E-02	chr6(+):35252652-35263490	UCSC	RIKEN cDNA 1810058I24 gene	AK082166	67705	ENSMUSG00000073155	9.8	10.42	Yes
GSMG0029918	Polr2j	up	1.53	1.93E-02	chr5(+):136116690-136122947	UCSC	polymerase (RNA) II (DNA directed) polypeptide J	AK158610	20022	ENSMUSG00000039771	7.78	8.4	Yes
GSMG0029908	Mdh2	up	1.53	3.42E-03	chr5(+):135778631-135790395	UCSC	malate dehydrogenase 2, NAD (mitochondrial)	AK135162	17448	ENSMUSG00000019179	10.39	11	No
GSMG0003085	Dnajc12	up	1.53	1.53E-02	chr10(+):63382443-63408840	UCSC	DnaJ (Hsp40) homolog, subfamily C, member 12	NM_001253685	30045	ENSMUSG00000036764	7.64	8.25	No
GSMG0024873	4930432M17Rik	up	1.53	3.64E-02	chr3(+):121670761-121682982	UCSC	RIKEN cDNA 4930432M17 gene	NM_001033814	619318	ENSMUSG00000074248	7.11	7.72	Yes
GSMG0024535	Gba	up	1.53	6.48E-03	chr3(+):89202927-89208681	UCSC	glucosidase, beta, acid	AK082767	14466	ENSMUSG00000028048	9.62	10.23	No
GSMG0024364	P2ry1	up	1.53	3.08E-03	chr3(+):61002793-61008982	UCSC	purinergic receptor P2Y, G-protein coupled 1	AK030759	18441	ENSMUSG00000027765	6.85	7.47	No
GSMG0023771	Scand1	up	1.53	1.28E-02	chr2(-):156311847-156312704	UCSC	SCAN domain-containing 1	NM_020255	19018	ENSMUSG00000046229	10.07	10.68	Yes
GSMG0022862	Rbms1	up	1.53	1.73E-02	chr2(-):60751953-60963205	UCSC	RNA binding motif, single stranded interacting protein 1	NM_020296	56878	ENSMUSG00000026970	9.37	9.99	No
GSMG0022811	Cacnb4	up	1.53	1.30E-02	chr2(-):52428320-52676584	UCSC	calcium channel, voltage-dependent, beta 4 subunit	NM_001037099	12298	ENSMUSG00000017412	6.16	6.78	Yes
GSMG0022509	---	up	1.53	4.61E-02	chr2(-):24874532-24876329	UCSC	---	AK158801	---	---	6.28	6.9	No
GSMG0021042	---	up	1.52	2.97E-02	chr2(+):49216093-49217708	UCSC	---	AK037989	---	---	7.26	7.87	No
GSMG0020463	Cuedc2	up	1.52	2.21E-04	chr19(-):46329812-46338660	UCSC	CUE domain containing 2	NM_024192	67116	ENSMUSG00000036748	7.99	8.59	No
GSMG0018721	Npc1	up	1.52	3.64E-02	chr18(-):12189694-12236386	UCSC	Niemann Pick type C1	NM_008720	18145	ENSMUSG00000024413	7.71	8.32	Yes
GSMG0018329	Tcerg1	up	1.52	9.83E-04	chr18(+):42511487-42575785	UCSC	transcription elongation regulator 1 (CA150)	NM_001039474	56070	ENSMUSG00000024498	9.25	9.85	No
GSMG0015166	Gbe1	up	1.52	1.67E-02	chr16(+):70313949-70569720	UCSC	glucan (1,4-alpha-), branching enzyme 1	NM_028803	74185	ENSMUSG00000022707	8.36	8.97	No
GSMG0015067	Gm608	up	1.52	2.39E-02	chr16(+):44173397-44227463	UCSC	predicted gene 608	NM_001029889	207806	ENSMUSG00000068284	8.65	9.25	Yes
GSMG0007328	Ikzf3	up	1.52	2.61E-02	chr11(-):98464693-98546033	UCSC	IKAROS family zinc finger 3	NM_011771	22780	ENSMUSG00000018168	10.13	10.73	No
GSMG0005565	Git1	up	1.52	3.74E-02	chr11(+):77493412-77507774	UCSC	G protein-coupled receptor kinase-interactor 1	NM_001004144	216963	ENSMUSG00000011877	8.36	8.96	No
GSMG0052575	Gm5643	up	1.52	1.85E-02	chrX(+):142297505-142299237	UCSC	predicted gene 5643	NR_002883	434858	ENSMUSG00000083311	11.2	11.81	No
GSMG0041750	Hprt	up	1.52	1.74E-02	chrX(+):52988078-53021660	UCSC	hypoxanthine guanine phosphoribosyl transferase	NM_013556	15452	ENSMUSG00000025630	10.24	10.85	No
GSMG0041239	Nudt16	up	1.52	3.34E-02	chr9(-):105129338-105131806	UCSC	nudix (nucleoside diphosphate linked moiety X)-type motif 16	NM_029385	75686	ENSMUSG00000032565	8	8.61	No
GSMG0039749	Bnip2	up	1.52	1.35E-02	chr9(+):69989466-70011659	UCSC	BCL2/adenovirus E1B interacting protein 2	NM_016787	12175	ENSMUSG00000011958	9.72	10.32	Yes
GSMG0039257	Sep-07	up	1.52	5.98E-03	chr9(+):25252439-25308571	UCSC	septin 7	NM_009859	235072	ENSMUSG00000001833	10.39	10.99	No
GSMG0037380	Cope	up	1.52	1.36E-03	chr8(+):70302768-70312993	UCSC	coatomer protein complex, subunit epsilon	AK140753	59042	ENSMUSG00000055681	9.52	10.12	No
GSMG0035979	Svip	up	1.52	1.96E-02	chr7(-):51997162-52006018	UCSC	small VCP/p97-interacting protein	NM_001160345	75744	ENSMUSG00000074093	7.91	8.51	No
GSMG0033093	A530053G22Rik	up	1.52	5.44E-03	chr6(-):60279043-60403708	UCSC	RIKEN cDNA A530053G22 gene	AK080087	208079	ENSMUSG00000046764	6.07	6.67	Yes
GSMG0032169	Gfpt1	up	1.52	3.01E-02	chr6(+):87042844-87092207	UCSC	glutamine fructose-6-phosphate transaminase 1	NM_013528	14583	ENSMUSG00000029992	8.91	9.51	Yes
GSMG0031223	---	up	1.52	2.74E-03	chr5(-):136545787-136547206	UCSC	---	AK047012	---	---	5.98	6.58	No
GSMG0028886	Fgl2	up	1.52	3.80E-02	chr5(+):21372640-21378386	UCSC	fibrinogen-like protein 2	NM_008013	14190	ENSMUSG00000039899	7.45	8.05	Yes
GSMG0003305	Aes	up	1.52	4.09E-02	chr10(+):81559444-81566371	UCSC	amino-terminal enhancer of split	NR_074087	14797	ENSMUSG00000054452	9.85	10.46	No
GSMG0026870	Hspb11	up	1.52	2.09E-02	chr4(+):107253898-107279888	UCSC	heat shock protein family B (small), member 11	AK013636	72938	ENSMUSG00000063172	6.33	6.93	No
GSMG0025481	---	up	1.52	2.61E-02	chr3(-):65829116-65872282	UCSC	---	AK144610	---	---	7.7	8.3	Yes
GSMG0024468	---	up	1.52	3.46E-02	chr3(+):84852673-84854489	UCSC	---	AK041401	---	---	6.63	7.23	Yes
GSMG0021995	Kif3b	up	1.51	3.34E-02	chr2(+):153291416-153333387	UCSC	kinesin family member 3B	NM_008444	16569	ENSMUSG00000027475	8.48	9.07	No
GSMG0020933	Fam129b	up	1.51	2.30E-02	chr2(+):32876134-32925253	UCSC	family with sequence similarity 129, member B	NM_146119	227737	ENSMUSG00000026796	7.23	7.82	No
GSMG0020543	Dclre1a	up	1.51	7.56E-03	chr19(-):56529161-56548222	UCSC	DNA cross-link repair 1A, PSO2 homolog (S. cerevisiae)	NM_018831	55947	ENSMUSG00000025077	7.31	7.9	No
GSMG0019822	Acsl5	up	1.51	2.69E-02	chr19(+):55253368-55297720	UCSC	acyl-CoA synthetase long-chain family member 5	AK034942	433256	ENSMUSG00000024981	8.75	9.34	No
GSMG0019679	Lcor	up	1.51	3.16E-03	chr19(+):41482637-41562246	UCSC	ligand dependent nuclear receptor corepressor	AK155007	212391	ENSMUSG00000025019	8.55	9.14	Yes
GSMG0018776	Zscan30	up	1.51	1.14E-03	chr18(-):23966680-23987228	UCSC	zinc finger and SCAN domain containing 30	AK049082	328918	ENSMUSG00000024274	6.47	7.07	No
GSMG0018695	Mpp7	up	1.51	2.48E-02	chr18(-):7347962-7626867	UCSC	membrane protein, palmitoylated 7 (MAGUK p55 subfamily member 7)	NM_001081287	75739	ENSMUSG00000057440	7.95	8.54	No
GSMG0018315	Pabpc2	up	1.51	3.44E-02	chr18(+):39773495-39776082	UCSC	poly(A) binding protein, cytoplasmic 2	NM_011033	18459	ENSMUSG00000051732	8.11	8.71	Yes
GSMG0017692	Plin3	up	1.51	4.45E-02	chr17(-):56277476-56290513	UCSC	perilipin 3	AK132313	66905	ENSMUSG00000024197	8.55	9.15	No
GSMG0016114	Psmg1	up	1.51	1.13E-02	chr16(-):95979935-95990917	UCSC	proteasome (prosome, macropain) assembly chaperone 1	AK088118	56088	ENSMUSG00000022913	8.29	8.89	Yes
GSMG0015706	Fam162a	up	1.51	1.84E-03	chr16(-):36043761-36071524	UCSC	family with sequence similarity 162, member A	BC010826	70186	ENSMUSG00000003955	8.5	9.1	No
GSMG0013884	Zfp706	up	1.51	4.39E-02	chr15(-):36997029-37007402	UCSC	zinc finger protein 706	NM_026521	68036	ENSMUSG00000062397	9.92	10.51	Yes
GSMG0013535	---	up	1.51	5.55E-04	chr15(+):91757561-91758999	UCSC	---	AK084227	---	---	8.08	8.67	No
GSMG0012635	---	up	1.51	4.89E-02	chr14(-):72631258-72633097	UCSC	---	AK054012	---	---	7.61	8.21	No
GSMG0001018	Tmco1	up	1.51	4.07E-02	chr1(+):167308386-167333978	UCSC	transmembrane and coiled-coil domains 1	NM_001039483	68944	ENSMUSG00000052428	8.7	9.3	No
GSMG0008437	Psmc1	up	1.51	2.58E-02	chr12(+):100110151-100123405	UCSC	protease (prosome, macropain) 26S subunit, ATPase 1	AK133926	19179	ENSMUSG00000021178	9.06	9.66	No
GSMG0007399	Rab5c	up	1.51	1.88E-03	chr11(-):100715011-100738200	UCSC	RAB5C, member RAS oncogene family	AK089199	19345	ENSMUSG00000019173	9.98	10.57	No
GSMG0006503	Dock2	up	1.51	3.18E-03	chr11(-):34226827-34783905	UCSC	dedicator of cyto-kinesis 2	NM_033374	94176	ENSMUSG00000020143	10.41	11.01	No
GSMG0041923	Zxdb	up	1.51	2.47E-02	chrX(+):94724569-94730191	UCSC	zinc finger, X-linked, duplicated B	NM_001081473	668166	ENSMUSG00000073062	7.94	8.53	No
GSMG0037248	Ufsp2	up	1.51	6.46E-03	chr8(+):45975527-45996958	UCSC	UFM1-specific peptidase 2	NM_138668	192169	ENSMUSG00000031634	8.56	9.15	Yes
GSMG0030984	Prkab1	up	1.51	6.52E-03	chr5(-):116013588-116024448	UCSC	protein kinase, AMP-activated, beta 1 non-catalytic subunit	AK153552	19079	ENSMUSG00000029513	10.16	10.75	Yes
GSMG0030413	Clnk	up	1.51	2.92E-02	chr5(-):38706457-38876812	UCSC	cytokine-dependent hematopoietic cell linker	AB021220	27278	ENSMUSG00000039315	6.28	6.88	Yes
GSMG0023664	Cst3	up	1.51	1.26E-03	chr2(-):148871720-148875497	UCSC	cystatin C	AK146333	13010	ENSMUSG00000027447	12.14	12.74	No
GSMG0021557	---	up	1.5	3.60E-03	chr2(+):112326186-112327420	UCSC	---	AK081855	---	---	8.47	9.06	Yes
GSMG0016519	Zfp414 // Pram1	up	1.5	1.13E-02	chr17(+):33629092-33645705	UCSC	zinc finger protein 414 // PML-RAR alpha-regulated adaptor molecule 1	AK076291	328801 // 378460	USG00000032739 // ENSMUSG00000	8.61	9.2	No
GSMG0013949	---	up	1.5	2.69E-02	chr15(-):50740464-50742375	UCSC	---	AK019930	---	---	6.6	7.19	No
GSMG0011039	Mrps36	up	1.5	4.73E-02	chr13(-):100735940-100744659	UCSC	mitochondrial ribosomal protein S36	NM_025369	66128	ENSMUSG00000061474	7.97	8.56	No
GSMG0007738	Fam195b	up	1.5	1.60E-02	chr11(-):120542890-120549727	UCSC	family with sequence similarity 195, member B	NM_001033231	192173	ENSMUSG00000061111	8.47	9.05	Yes
GSMG0007311	Pip4k2b	up	1.5	4.23E-02	chr11(-):97715158-97744754	UCSC	phosphatidylinositol-5-phosphate 4-kinase, type II, beta	NM_054051	108083	ENSMUSG00000018547	8.6	9.19	No
GSMG0043689	4930485B16Rik	up	1.5	3.09E-02	chr10(-):93016535-93045119	UCSC	RIKEN cDNA 4930485B16 gene	AK039960	380654	ENSMUSG00000020014	6.32	6.91	No
GSMG0038592	Cyp4f18	up	1.5	5.96E-03	chr8(-):71988482-72009628	UCSC	cytochrome P450, family 4, subfamily f, polypeptide 18	AK007863	72054	ENSMUSG00000003484	9.18	9.77	Yes
GSMG0030541	Ugdh	up	1.5	1.47E-02	chr5(-):65413221-65435878	UCSC	UDP-glucose dehydrogenase	AK144673	22235	ENSMUSG00000029201	9.16	9.74	No
GSMG0003045	4632428N05Rik	up	1.5	3.12E-03	chr10(+):60346851-60430294	UCSC	RIKEN cDNA 4632428N05 gene	AK162635	74048	ENSMUSG00000020101	9.79	10.38	No
GSMG0024848	Sass6	up	1.5	3.68E-02	chr3(+):116594980-116630984	UCSC	spindle assembly 6 homolog (C. elegans)	NM_028349	72776	ENSMUSG00000027959	7.1	7.69	Yes
GSMG0023755	Eif6	up	1.5	2.99E-02	chr2(-):155819837-155826925	UCSC	eukaryotic translation initiation factor 6	NM_010579	16418	ENSMUSG00000027613	9.69	10.28	No
GSMG0043055	Ddx3y	down	52.34	1.39E-04	chrY(-):1260715-1286613	UCSC	DEAD (Asp-Glu-Ala-Asp) box polypeptide 3, Y-linked	NM_012008	26900	ENSMUSG00000069045	11.01	5.3	No
GSMG0043046	Eif2s3y	down	46.2	1.39E-06	chrY(+):1010594-1028847	UCSC	eukaryotic translation initiation factor 2, subunit 3, structural gene Y-linked	AK049742	26908	ENSMUSG00000069049	10.13	4.6	No
GSMG0043044	Kdm5d	down	10.27	5.84E-05	chrY(+):897787-956779	UCSC	lysine (K)-specific demethylase 5D	BC060077	20592	ENSMUSG00000056673	8.54	5.18	No
GSMG0043054	---	down	5.91	2.65E-04	chrY(-):1212111-1215972	UCSC	---	AK153586	---	---	8.82	6.26	No
GSMG0043074	---	down	5.54	2.19E-05	chrY(-):85024230-85104498	UCSC	---	AK154753	---	---	8.88	6.41	Yes
GSMG0036319	---	down	5.52	1.59E-04	chr7(-):99900192-99904156	UCSC	---	AK161464	---	---	8.02	5.56	Yes
GSMG0036373	Usp17ld	down	4.45	3.44E-03	chr7(-):103249763-103252511	UCSC	ubiquitin specific peptidase 17-like D	BC148743	384701	ENSMUSG00000057321	8.29	6.14	No
GSMG0021364	Olfr1274-ps	down	4.35	2.38E-03	chr2(+):90400640-90401674	UCSC	olfactory receptor 1274, pseudogene	NM_146263	258330	ENSMUSG00000082980	9.23	7.1	Yes
GSMG0015218	Mir125b-2	down	3.94	3.56E-03	chr16(+):77646273-77649426	UCSC	microRNA 125b-2	AK047945	723952	ENSMUSG00000065472	7.5	5.52	Yes
GSMG0035626	Gm4763	down	3.66	4.16E-03	chr7(-):24722496-24724290	UCSC	predicted gene 4763	NM_177593	210155	ENSMUSG00000058717	10.22	8.34	Yes
GSMG0036225	---	down	3.5	3.00E-03	chr7(-):83874362-83877920	UCSC	---	AK035917	---	---	7.34	5.53	Yes
GSMG0043697	---	down	3.26	1.35E-02	chr11(+):62513757-62516029	UCSC	---	AK077687	---	---	7.18	5.48	Yes
GSMG0037185	---	down	3.26	4.49E-02	chr8(+):34820584-34821951	UCSC	---	AK012530	---	---	10.9	9.2	No
GSMG0051970	---	down	3.22	2.48E-03	chr4(-):144777204-144786583	UCSC	---	NM_001126316	---	---	8.82	7.14	Yes
GSMG0052020	---	down	3.11	2.28E-03	chr5(-):108842947-108852510	UCSC	---	NM_001104621	---	---	7.8	6.16	No
GSMG0006355	1700046C09Rik	down	3.07	4.19E-02	chr11(-):14560336-14599225	UCSC	RIKEN cDNA 1700046C09 gene	NR_045918	73307	ENSMUSG00000085561	7.28	5.66	Yes
GSMG0035782	Wtip	down	3.05	1.02E-03	chr7(-):34109543-34133268	UCSC	WT1-interacting protein	NM_207212	101543	ENSMUSG00000036459	7.6	5.99	No
GSMG0052157	Scgb1b30	down	3.05	4.90E-03	chr7(+):34095431-34100837	UCSC	secretoglobin, family 1B, member 30	NM_001099330	100043868	ENSMUSG00000078752	7.78	6.17	Yes
GSMG0049040	Mir150	down	3.04	5.00E-03	chr7(+):45121757-45121821	UCSC	microRNA 150	NR_029560	387168	ENSMUSG00000065495	8.72	7.12	No
GSMG0023107	Olfr1156	down	2.93	7.00E-04	chr2(-):87949190-87950265	UCSC	olfactory receptor 1156	NM_146817	258814	ENSMUSG00000075144	6.54	4.99	Yes
GSMG0041718	---	down	2.9	8.83E-04	chrX(+):44789876-44793880	UCSC	---	AK141448	---	---	7.18	5.65	Yes
GSMG0025415	---	down	2.83	6.06E-03	chr3(-):55724050-55728423	UCSC	---	AK084877	---	---	7.47	5.97	Yes
GSMG0025073	---	down	2.77	1.81E-05	chr3(+):146016173-146019209	UCSC	---	AK036201	---	---	7.92	6.45	No
GSMG0004870	---	down	2.72	1.66E-02	chr11(+):6573314-6574675	UCSC	---	AK077821	---	---	8.95	7.51	Yes
GSMG0021052	---	down	2.69	6.42E-04	chr2(+):49845934-49850270	UCSC	---	AK142046	---	---	7.43	6	No
GSMG0037079	Defb13	down	2.68	2.40E-03	chr8(+):21946762-21948850	UCSC	defensin beta 13	AJ575427	246083	ENSMUSG00000044222	9.16	7.74	Yes
GSMG0034398	Klk14	down	2.68	2.08E-03	chr7(+):43690415-43695535	UCSC	kallikrein related-peptidase 14	NM_174866	317653	ENSMUSG00000044737	9.63	8.21	Yes
GSMG0052336	Olfr566	down	2.66	2.31E-02	chr7(-):102856321-102857271	UCSC	olfactory receptor 566	NM_001011536	258168	ENSMUSG00000060888	7.62	6.21	No
GSMG0029493	---	down	2.62	2.33E-05	chr5(+):102427418-102429414	UCSC	---	AK046390	---	---	8.6	7.22	Yes
GSMG0003472	Gm20110	down	2.61	2.04E-02	chr10(+):99609170-99658134	UCSC	predicted gene, 20110	AK039362	100504202	---	8.16	6.77	Yes
GSMG0023246	BC016548	down	2.61	6.52E-03	chr2(-):103383987-103389042	UCSC	cDNA sequence BC016548	BC016548	---	ENSMUSG00000070794	7.4	6.01	Yes
GSMG0018188	---	down	2.6	2.26E-02	chr18(+):32944313-32948486	UCSC	---	AK038046	---	---	9.56	8.18	No
GSMG0000601	---	down	2.6	4.41E-04	chr1(+):93221503-93223106	UCSC	---	AK035048	---	---	7.62	6.25	Yes
GSMG0024211	---	down	2.58	1.22E-03	chr3(+):34740644-34741887	UCSC	---	AK048856	---	---	7.18	5.81	Yes
GSMG0042957	1700042B14Rik	down	2.57	1.14E-02	chrX(-):155124917-155147849	UCSC	RIKEN cDNA 1700042B14 gene	NM_001081671	73347	ENSMUSG00000057402	8.39	7.03	Yes
GSMG0052551	Rhox2f	down	2.56	9.57E-04	chrX(+):37571421-37576159	UCSC	reproductive homeobox 2F	NM_001085356	434764	ENSMUSG00000095741	8.04	6.68	Yes
GSMG0051730	Gm14139	down	2.53	9.48E-03	chr2(+):150181755-150193279	UCSC	predicted gene 14139	NM_001145863	100271882	ENSMUSG00000079009	8.55	7.21	Yes
GSMG0009198	---	down	2.53	1.02E-03	chr12(-):99376596-99377249	UCSC	---	AK078354	---	---	9.13	7.79	No
GSMG0017659	---	down	2.52	4.88E-02	chr17(-):51745529-51748684	UCSC	---	AK088368	---	---	11.04	9.71	No
GSMG0018540	---	down	2.51	4.17E-02	chr18(+):67937752-67940402	UCSC	---	AK045980	---	---	8.12	6.8	No
GSMG0010772	Cts3	down	2.51	2.54E-03	chr13(-):61564631-61570127	UCSC	cathepsin 3	NM_054092	117066	ENSMUSG00000074870	7.29	5.96	Yes
GSMG0019840	---	down	2.5	2.36E-03	chr19(+):55895962-55898589	UCSC	---	BC131679	---	---	8	6.68	No
GSMG0043709	Pde4d	down	2.5	1.27E-02	chr13(+):108860061-109955969	UCSC	phosphodiesterase 4D, cAMP specific	NM_011056	100862401	ENSMUSG00000021699	10.6	9.28	No
GSMG0017494	Gm10499	down	2.49	1.58E-02	chr17(-):36141749-36145744	UCSC	predicted gene 10499	AK161769	69717	ENSMUSG00000073403	8.67	7.36	No
GSMG0000995	---	down	2.48	5.38E-03	chr1(+):165325833-165326745	UCSC	---	BC019732	---	---	9.69	8.37	Yes
GSMG0034424	Klk1	down	2.45	1.79E-02	chr7(+):44225436-44229617	UCSC	kallikrein 1	AK002278	16612	ENSMUSG00000063903	7.31	6.01	Yes
GSMG0002320	Fasl	down	2.44	3.26E-02	chr1(-):161780689-161788495	UCSC	Fas ligand (TNF superfamily, member 6)	AK040139	14103	ENSMUSG00000000817	8.63	7.34	No
GSMG0027712	Ccl27a	down	2.44	4.93E-02	chr4(-):41769472-41774176	UCSC	chemokine (C-C motif) ligand 27A	NM_001048179	20301	ENSMUSG00000073888	7.31	6.02	Yes
GSMG0036428	Olfr686	down	2.43	4.68E-03	chr7(-):105203359-105204376	UCSC	olfactory receptor 686	BC132006	259072	ENSMUSG00000048425	7.61	6.33	Yes
GSMG0002362	Xcl1	down	2.42	2.98E-02	chr1(-):164931645-164935523	UCSC	chemokine (C motif) ligand 1	AK079820	16963	ENSMUSG00000026573	7.63	6.36	No
GSMG0039738	---	down	2.42	8.86E-03	chr9(+):69168041-69170385	UCSC	---	AK084224	---	---	6.97	5.7	No
GSMG0030174	---	down	2.42	4.66E-03	chr5(-):9481780-9484212	UCSC	---	AK033895	---	---	7.98	6.71	Yes
GSMG0023233	---	down	2.41	2.59E-02	chr2(-):102185351-102186057	UCSC	---	AK006976	---	---	7.25	5.98	Yes
GSMG0009196	---	down	2.39	2.77E-02	chr12(-):99359871-99362028	UCSC	---	AK012244	---	---	8.97	7.72	No
GSMG0040678	Cd3g	down	2.39	3.95E-02	chr9(-):44969572-44980432	UCSC	CD3 antigen, gamma polypeptide	NM_009850	12502	ENSMUSG00000002033	9.41	8.15	No
GSMG0017373	---	down	2.38	1.50E-03	chr17(-):32325904-32328459	UCSC	---	AK041970	---	---	7.81	6.56	No
GSMG0006341	4930512M02Rik	down	2.38	7.64E-03	chr11(-):11589223-11627839	UCSC	RIKEN cDNA 4930512M02 gene	AK015778	---	ENSMUSG00000062511	9.44	8.19	Yes
GSMG0024943	---	down	2.38	3.26E-02	chr3(+):131191889-131192966	UCSC	---	AK020111	---	---	8.54	7.29	Yes
GSMG0002783	C920009B18Rik	down	2.38	4.87E-02	chr10(+):22306700-22321765	UCSC	RIKEN cDNA C920009B18 gene	AK040102	606736	ENSMUSG00000097352	9.57	8.32	No
GSMG0008183	---	down	2.37	1.96E-02	chr12(+):73656262-73658189	UCSC	---	AK036797	---	---	8.93	7.68	No
GSMG0015740	Gap43	down	2.36	1.98E-03	chr16(-):42248444-42340651	UCSC	growth associated protein 43	AK031908	14432	ENSMUSG00000047261	8.75	7.51	Yes
GSMG0015541	---	down	2.36	1.72E-03	chr16(-):20293484-20294145	UCSC	---	AK050895	---	---	9.33	8.09	No
GSMG0001011	---	down	2.36	2.64E-03	chr1(+):166301442-166303422	UCSC	---	AK049921	---	---	7.02	5.78	No
GSMG0027403	Mir34a	down	2.36	9.65E-04	chr4(+):150068454-150070999	UCSC	microRNA 34a	AK047585	723848	ENSMUSG00000065493	6.78	5.55	Yes
GSMG0034416	Klk1b27	down	2.35	1.70E-03	chr7(+):44052288-44056712	UCSC	kallikrein 1-related peptidase b27	NM_020268	16619	ENSMUSG00000063177	7.1	5.87	Yes
GSMG0034546	Siglech	down	2.34	1.84E-02	chr7(+):55768177-55778925	UCSC	sialic acid binding Ig-like lectin H	AK045719	233274	ENSMUSG00000051504	8.3	7.07	No
GSMG0010991	Nsa2	down	2.33	2.75E-02	chr13(-):97129427-97137926	UCSC	NSA2 ribosome biogenesis homolog (S. cerevisiae)	NM_021552	59050	ENSMUSG00000060739	10.86	9.64	No

FAST DB STABLE ID	Gene Symbol	Regulation	Fold-Change	P-Value	Gene Coordinates (mm10)	UCSC Link	Gene Name	Representative Transcript ID	Entrez Gene ID	EnsEMBL ID	log^2 intensity of untreated	log^2 intensity of treated	Low-specificity probe included
GSMG0040493	---	down	2.33	2.07E-02	chr9(-):25248128-25252417	UCSC	---	AK046843	---	---	6.95	5.73	Yes
GSMG0040492	Herpud2	down	2.33	1.86E-05	chr9(-):25108130-25151820	UCSC	HERPUD family member 2	NM_020586	80517	ENSMUSG00000008429	10.5	9.28	No
GSMG0011486	Socs4	down	2.32	4.64E-03	chr14(+):47277012-47291591	UCSC	suppressor of cytokine signaling 4	NM_080843	67296	ENSMUSG00000048379	9.44	8.23	Yes
GSMG0018736	E430002N23Rik	down	2.31	2.52E-03	chr18(-):15308858-15312701	UCSC	RIKEN cDNA E430002N23 gene	AK088053	319440	---	7.74	6.53	Yes
GSMG0018352	---	down	2.31	4.21E-02	chr18(+):45482102-45484391	UCSC	---	AK085879	---	---	6.41	5.2	Yes
GSMG0015062	---	down	2.31	4.98E-04	chr16(+):43940447-43942750	UCSC	---	AK047832	---	---	8.89	7.68	Yes
GSMG0026623	---	down	2.31	1.12E-03	chr4(+):62412345-62414130	UCSC	---	AK087776	---	---	8.83	7.62	No
GSMG0023069	P2rx3	down	2.31	1.78E-03	chr2(-):85030221-85033195	UCSC	purinergic receptor P2X, ligand-gated ion channel, 3	AK019679	228139	ENSMUSG00000027071	10.38	9.17	No
GSMG0033226	---	down	2.3	7.84E-03	chr6(-):82050709-82052615	UCSC	---	AK132926	---	---	7.28	6.08	No
GSMG0020873	Odf2	down	2.29	6.60E-03	chr2(+):29793679-29896014	UCSC	outer dense fiber of sperm tails 2	AK086843	18286	ENSMUSG00000026790	7.82	6.63	Yes
GSMG0018183	---	down	2.29	1.33E-02	chr18(+):32398464-32404424	UCSC	---	AK132539	---	---	6.54	5.34	Yes
GSMG0015336	---	down	2.29	1.15E-02	chr16(+):94404295-94405887	UCSC	---	AK143009	---	---	8.69	7.5	No
GSMG0013033	March11	down	2.28	1.80E-03	chr15(+):26309047-26409573	UCSC	membrane-associated ring finger (C3HC4) 11	NM_177597	211147	ENSMUSG00000022269	8.17	6.98	No
GSMG0038713	---	down	2.28	1.39E-02	chr8(-):85675086-85675811	UCSC	---	AK081835	---	---	6.72	5.53	No
GSMG0052085	Vmn1r24	down	2.28	6.80E-03	chr6(-):57955641-57956531	UCSC	vomeronasal 1 receptor 24	NM_134173	171191	ENSMUSG00000093451	7.44	6.25	No
GSMG0003623	---	down	2.28	2.69E-02	chr10(+):122939206-122940123	UCSC	---	AK046716	---	---	7.77	6.59	No
GSMG0022930	---	down	2.28	5.39E-04	chr2(-):68373246-68375222	UCSC	---	AK134421	---	---	9.68	8.49	Yes
GSMG0021485	---	down	2.26	1.65E-02	chr2(+):103696551-103697584	UCSC	---	AK042022	---	---	7.51	6.33	No
GSMG0033372	4930471M09Rik	down	2.26	2.03E-02	chr6(-):91430460-91440855	UCSC	RIKEN cDNA 4930471M09 gene	AK015554	75787	---	6.28	5.1	Yes
GSMG0030120	1700028E10Rik	down	2.26	1.53E-02	chr5(+):151368675-151414083	UCSC	RIKEN cDNA 1700028E10 gene	NR_045700	625175	ENSMUSG00000097321	7.39	6.22	Yes
GSMG0038279	---	down	2.25	5.78E-03	chr8(-):34583025-34584493	UCSC	---	AK016354	---	---	7.93	6.76	Yes
GSMG0025424	---	down	2.25	6.08E-03	chr3(-):56051706-56054576	UCSC	---	AK046973	---	---	8.01	6.84	No
GSMG0020813	Gm13539	down	2.24	2.54E-02	chr2(+):25784615-25787022	UCSC	predicted gene 13539	AK076911	620858	ENSMUSG00000085484	7.38	6.21	Yes
GSMG0040286	Ccr5	down	2.24	2.80E-02	chr9(+):124121542-124127442	UCSC	chemokine (C-C motif) receptor 5	AK155632	12774	ENSMUSG00000079227	9.52	8.36	No
GSMG0026285	Gem	down	2.24	4.20E-02	chr4(+):11704445-11714993	UCSC	GTP binding protein (gene overexpressed in skeletal muscle)	AK158393	14579	ENSMUSG00000028214	11.65	10.49	No
GSMG0032048	Cd8a	down	2.23	7.74E-03	chr6(+):71373427-71379173	UCSC	CD8 antigen, alpha chain	NM_009857	12525	ENSMUSG00000053977	10.15	8.99	No
GSMG0010665	---	down	2.22	4.40E-02	chr13(-):52087607-52096669	UCSC	---	AK029587	---	---	8.14	6.99	No
GSMG0040248	Vipr1	down	2.22	3.06E-03	chr9(+):121642715-121681274	UCSC	vasoactive intestinal peptide receptor 1	BC156777	22354	ENSMUSG00000032528	8.01	6.86	No
GSMG0035506	Zscan18	down	2.21	1.24E-02	chr7(-):12768092-12775658	UCSC	zinc finger and SCAN domain containing 18	NM_001017955	232875	ENSMUSG00000070822	7.93	6.79	No
GSMG0021350	Olfr1140	down	2.2	8.02E-03	chr2(+):87746161-87747180	UCSC	olfactory receptor 1140	NM_146642	258635	ENSMUSG00000068817	6.96	5.82	No
GSMG0039834	---	down	2.2	4.23E-02	chr9(+):77943703-77946916	UCSC	---	AK038263	---	---	8.24	7.11	No
GSMG0037084	Thsd1	down	2.2	1.03E-02	chr8(+):22227303-22261334	UCSC	thrombospondin, type I, domain 1	NM_019576	56229	ENSMUSG00000031480	9.4	8.26	Yes
GSMG0033943	---	down	2.19	1.50E-02	chr7(+):3981155-3982877	UCSC	---	AK018932	---	---	7.9	6.76	No
GSMG0037447	Klf2	down	2.18	1.06E-03	chr8(+):72319033-72321654	UCSC	Kruppel-like factor 2 (lung)	AK131962	16598	ENSMUSG00000055148	11.29	10.17	Yes
GSMG0031653	---	down	2.17	6.76E-03	chr6(+):33283427-33285700	UCSC	---	AK049535	---	---	8.02	6.9	No
GSMG0017068	Ldhal6b	down	2.16	2.40E-02	chr17(-):5417325-5418767	UCSC	lactate dehydrogenase A-like 6B	NM_175349	106557	---	7.72	6.61	Yes
GSMG0005015	---	down	2.16	1.74E-03	chr11(+):32481917-32484406	UCSC	---	AK047124	---	---	8.25	7.14	No
GSMG0042229	4930503H13Rik	down	2.16	3.04E-04	chrX(+):156539483-156540316	UCSC	RIKEN cDNA 4930503H13 gene	AK015689	74941	ENSMUSG00000082612	6.75	5.64	Yes
GSMG0026364	Bach2	down	2.16	8.16E-03	chr4(+):32417435-32586108	UCSC	BTB and CNC homology 2	NM_001109661	12014	ENSMUSG00000040270	10.76	9.65	No
GSMG0016669	---	down	2.15	2.22E-02	chr17(+):40115645-40116041	UCSC	---	AK217333	---	---	9.97	8.87	Yes
GSMG0036927	---	down	2.15	5.81E-04	chr7(-):144368048-144406934	UCSC	---	AK042115	---	---	6.99	5.88	Yes
GSMG0036423	Olfr672	down	2.15	4.84E-02	chr7(-):104995827-104996906	UCSC	olfactory receptor 672	BC160235	258755	ENSMUSG00000051172	6.95	5.84	No
GSMG0027165	Snhg12	down	2.15	2.28E-02	chr4(+):132308674-132311024	UCSC	small nucleolar RNA host gene 12	AK009175	100039864	ENSMUSG00000086290	8.74	7.63	Yes
GSMG0023985	2210418O10Rik	down	2.15	7.70E-03	chr2(+):176226568-176261871	UCSC	RIKEN cDNA 2210418O10 gene	BC080301	100504263	ENSMUSG00000078894	7.52	6.42	Yes
GSMG0028150	---	down	2.14	1.97E-02	chr4(-):106841156-106847922	UCSC	---	AK137110	---	---	8.6	7.5	Yes
GSMG0041515	---	down	2.13	4.54E-02	chrM(+):9459-9804	UCSC	---	AK139029	---	---	8.91	7.82	Yes
GSMG0040713	Nnmt	down	2.13	2.58E-02	chr9(-):48591877-48605125	UCSC	nicotinamide N-methyltransferase	U89377	18113	ENSMUSG00000032271	8.5	7.41	Yes
GSMG0001004	---	down	2.12	1.83E-02	chr1(+):165797393-165800928	UCSC	---	AK038168	---	---	8.66	7.57	Yes
GSMG0024939	---	down	2.12	5.62E-03	chr3(+):131121166-131123598	UCSC	---	AK037590	---	---	8.49	7.41	No
GSMG0015889	---	down	2.11	3.06E-02	chr16(-):63835448-63838626	UCSC	---	AK083601	---	---	6.65	5.57	Yes
GSMG0015509	---	down	2.11	6.40E-03	chr16(-):17699939-17701158	UCSC	---	AK032900	---	---	8.13	7.05	No
GSMG0014676	---	down	2.11	6.12E-03	chr16(+):11779729-11781500	UCSC	---	AK014704	---	---	7.55	6.47	Yes
GSMG0006578	Itk	down	2.11	4.62E-02	chr11(-):46324349-46389515	UCSC	IL2 inducible T cell kinase	NM_010583	16428	ENSMUSG00000020395	11.46	10.39	No
GSMG0051291	Gm11937	down	2.1	2.84E-03	chr11(-):99609794-99610189	UCSC	predicted gene 11937	NM_001099346	100041488	ENSMUSG00000058725	8.93	7.86	Yes
GSMG0041166	---	down	2.1	1.29E-02	chr9(-):97068183-97073429	UCSC	---	BC066854	---	---	8.72	7.66	No
GSMG0034644	---	down	2.1	3.40E-02	chr7(+):75560614-75563794	UCSC	---	AK085623	---	---	7.44	6.37	Yes
GSMG0033805	---	down	2.1	2.70E-02	chr6(-):134752638-134753783	UCSC	---	AK048124	---	---	8.77	7.69	No
GSMG0011607	---	down	2.09	7.42E-03	chr14(+):55643603-55646352	UCSC	---	AK149247	---	---	7.06	6	No
GSMG0039358	---	down	2.09	2.11E-02	chr9(+):37808616-37810102	UCSC	---	AK157877	---	---	6.7	5.64	No
GSMG0031407	Zar1l	down	2.09	5.18E-03	chr5(-):150507069-150518159	UCSC	zygote arrest 1-like	NM_001159693	545824	ENSMUSG00000056586	7.93	6.87	Yes
GSMG0027360	---	down	2.09	3.90E-03	chr4(+):145838051-145841632	UCSC	---	AK038044	---	---	7.51	6.45	No
GSMG0026288	---	down	2.09	3.36E-03	chr4(+):11969837-11973934	UCSC	---	AK040963	---	---	7.78	6.71	Yes
GSMG0020481	Nt5c2	down	2.08	1.08E-02	chr19(-):46886830-47015189	UCSC	5'-nucleotidase, cytosolic II	NM_001164363	76952	ENSMUSG00000025041	9.31	8.26	No
GSMG0004068	---	down	2.08	3.80E-02	chr10(-):49351065-49354256	UCSC	---	AK051556	---	---	7.13	6.08	Yes
GSMG0002834	---	down	2.08	2.12E-02	chr10(+):28489238-28492586	UCSC	---	AK044847	---	---	5.88	4.82	Yes
GSMG0021344	Olfr1120	down	2.07	2.16E-03	chr2(+):87357426-87358443	UCSC	olfactory receptor 1120	BC119143	259031	ENSMUSG00000070853	7.85	6.8	Yes
GSMG0018836	Hspa9	down	2.07	4.58E-03	chr18(-):34937417-34954353	UCSC	heat shock protein 9	NM_010481	15526	ENSMUSG00000024359	10.71	9.66	No
GSMG0035634	---	down	2.07	3.24E-02	chr7(-):25087352-25088542	UCSC	---	AK080501	---	---	8.76	7.71	No
GSMG0035200	Srcap	down	2.07	4.44E-04	chr7(+):127565469-127567054	UCSC	Snf2-related CREBBP activator protein	AK081131	100043597	ENSMUSG00000090663	7.31	6.26	Yes
GSMG0034890	---	down	2.07	1.86E-02	chr7(+):100811352-100812637	UCSC	---	AK157011	---	---	7.48	6.44	Yes
GSMG0034421	Klk1b3	down	2.07	3.14E-02	chr7(+):44198191-44202351	UCSC	kallikrein 1-related peptidase b3	NM_008693	18050	ENSMUSG00000066515	6.97	5.92	Yes
GSMG0037976	Rab4a	down	2.06	8.16E-03	chr8(+):123805996-123835290	UCSC	RAB4A, member RAS oncogene family	NM_009003	19341	ENSMUSG00000019478	8.2	7.15	No
GSMG0002830	---	down	2.06	3.21E-02	chr10(+):28235420-28237040	UCSC	---	AK087080	---	---	7.75	6.71	Yes
GSMG0008153	4930404H11Rik	down	2.05	3.70E-03	chr12(+):71540607-71556133	UCSC	RIKEN cDNA 4930404H11 gene	NR_045941	73815	---	6.77	5.74	No
GSMG0005840	---	down	2.05	8.48E-03	chr11(+):96637370-96638483	UCSC	---	AK137900	---	---	7.33	6.29	No
GSMG0029035	---	down	2.05	1.88E-02	chr5(+):32636558-32638280	UCSC	---	AK082417	---	---	6.53	5.5	Yes
GSMG0015716	---	down	2.04	3.04E-02	chr16(-):36638898-36642763	UCSC	---	AK154117	---	---	8.79	7.76	No
GSMG0011080	---	down	2.04	3.13E-02	chr13(-):104030529-104033486	UCSC	---	AK137742	---	---	7.64	6.61	No
GSMG0008036	---	down	2.04	5.32E-03	chr12(+):44512121-44514808	UCSC	---	AK051190	---	---	7.85	6.82	No
GSMG0040282	Ccr9	down	2.04	2.01E-02	chr9(+):123678096-123783457	UCSC	chemokine (C-C motif) receptor 9	AK019478	12769	ENSMUSG00000029530	7.82	6.79	No
GSMG0030345	---	down	2.04	1.20E-02	chr5(-):32713072-32713988	UCSC	---	AK078320	---	---	8	6.97	No
GSMG0009608	---	down	2.03	4.66E-02	chr13(+):31811161-31812476	UCSC	---	AK016066	---	---	6.39	5.37	Yes
GSMG0007472	Map3k14 // 1700028N14Ril	down	2.03	1.34E-03	chr11(-):103219764-103267746	UCSC	mitogen-activated protein kinase kinase kinase 14 // RIKEN cDNA 1700028N	AK036200	53859	USG00000020941 // ENSMUSG00000	9.06	8.04	No
GSMG0041831	Xlr3b	down	2.03	3.62E-02	chrX(+):73192179-73202929	UCSC	X-linked lymphocyte-regulated 3B	NM_001081643	574437	ENSMUSG00000073125	6.42	5.4	No
GSMG0052156	---	down	2.03	3.46E-03	chr7(+):31375592-31376916	UCSC	---	NM_001256073	---	---	11.45	10.43	Yes
GSMG0011688	Fam167a	down	2.02	4.74E-02	chr14(+):63436394-63465501	UCSC	family with sequence similarity 167, member A	NM_177628	219148	ENSMUSG00000035095	8.48	7.47	No
GSMG0032537	Klrd1	down	2.02	2.28E-02	chr6(+):129591782-129598775	UCSC	killer cell lectin-like receptor, subfamily D, member 1	AK136548	16643	ENSMUSG00000030165	8.01	6.99	Yes
GSMG0032021	A430010J10Rik	down	2.02	3.75E-02	chr6(+):67115672-67168426	UCSC	RIKEN cDNA A430010J10 gene	AK039826	319665	ENSMUSG00000048665	8.52	7.51	Yes
GSMG0031364	---	down	2.02	2.57E-02	chr5(-):146203253-146204789	UCSC	---	BC050222	---	---	7.13	6.12	Yes
GSMG0010726	---	down	2.01	2.99E-02	chr13(-):58000227-58003996	UCSC	---	AK033046	---	---	7.19	6.18	Yes
GSMG0009355	Gpr132	down	2.01	4.33E-02	chr12(-):112850873-112860952	UCSC	G protein-coupled receptor 132	AK154509	56696	ENSMUSG00000021298	10.56	9.55	No
GSMG0006408	---	down	2.01	2.63E-02	chr11(-):22222151-22225834	UCSC	---	AK043918	---	---	8.12	7.11	Yes
GSMG0028604	---	down	2.01	4.52E-03	chr4(-):142071610-142073923	UCSC	---	AK030556	---	---	6.72	5.72	No
GSMG0016786	---	down	2	2.86E-02	chr17(+):51842861-51846116	UCSC	---	AK158910	---	---	8.12	7.12	No
GSMG0055107	Gm21464	down	2	2.33E-02	chr14(-):69363504-69503365	UCSC	predicted gene, 21464	AK031288	100862085	ENSMUSG00000093954	8.39	7.39	No
GSMG0036728	---	down	2	2.74E-03	chr7(-):128408968-128410993	UCSC	---	AK079512	---	---	7.52	6.52	No
GSMG0016001	---	down	1.99	6.90E-03	chr16(-):86139304-86150677	UCSC	---	AK005715	---	---	7.46	6.47	Yes
GSMG0009005	---	down	1.99	7.17E-04	chr12(-):75521314-75523887	UCSC	---	AK140806	---	---	9.96	8.97	No
GSMG0006648	---	down	1.99	1.76E-02	chr11(-):52267044-52269514	UCSC	---	AK137764	---	---	7.9	6.91	No
GSMG0040537	---	down	1.99	4.86E-03	chr9(-):32477333-32480377	UCSC	---	AK038103	---	---	8.32	7.33	No
GSMG0035154	Il21r	down	1.99	1.65E-02	chr7(+):125603428-125633570	UCSC	interleukin 21 receptor	NM_021887	60504	ENSMUSG00000030745	11.06	10.07	No
GSMG0003051	Prf1	down	1.99	2.71E-02	chr10(+):61297836-61304263	UCSC	perforin 1 (pore forming protein)	NM_011073	18646	ENSMUSG00000037202	7.93	6.93	No
GSMG0022392	---	down	1.99	2.08E-03	chr2(-):7713657-7781412	UCSC	---	AK139593	---	---	8.58	7.59	Yes
GSMG0016442	Clpsl2	down	1.98	1.39E-02	chr17(+):28549411-28552618	UCSC	colipase-like 2	BC147117	328788	ENSMUSG00000024224	8.78	7.79	Yes
GSMG0005906	Gm11567	down	1.98	4.02E-04	chr11(+):99879186-99880229	UCSC	predicted gene 11567	AK003994	670533	ENSMUSG00000059845	9.03	8.05	Yes
GSMG0041873	---	down	1.98	4.18E-02	chrX(+):77518854-77522195	UCSC	---	AK033910	---	---	7.95	6.97	Yes
GSMG0022929	---	down	1.98	3.82E-03	chr2(-):68330693-68333790	UCSC	---	AK081333	---	---	7.53	6.54	Yes
GSMG0020875	Urm1	down	1.97	3.80E-02	chr2(+):29827383-29844996	UCSC	ubiquitin related modifier 1 homolog (S. cerevisiae)	AK049849	68205	ENSMUSG00000069020	9.26	8.27	No
GSMG0012466	---	down	1.97	6.86E-03	chr14(-):57417271-57419483	UCSC	---	AK139567	---	---	7.19	6.21	Yes
GSMG0009185	Ptpn21	down	1.97	2.48E-03	chr12(-):98676741-98737405	UCSC	protein tyrosine phosphatase, non-receptor type 21	NM_001146199	24000	ENSMUSG00000021009	8.38	7.41	No
GSMG0006788	---	down	1.97	4.15E-02	chr11(-):62423616-62426473	UCSC	---	AK051935	---	---	8.98	8	No
GSMG0036590	Thumpd1	down	1.97	5.98E-03	chr7(-):119715096-119720774	UCSC	THUMP domain containing 1	AK152912	233802	ENSMUSG00000030942	8.74	7.76	Yes
GSMG0021588	---	down	1.96	8.84E-03	chr2(+):115807022-115811563	UCSC	---	AK136023	---	---	8.2	7.23	Yes
GSMG0020370	---	down	1.96	2.70E-02	chr19(-):40472755-40474430	UCSC	---	AK017539	---	---	6.53	5.56	Yes
GSMG0001534	Fhl2	down	1.96	2.14E-03	chr1(-):43123074-43196762	UCSC	four and a half LIM domains 2	AK052936	14200	ENSMUSG00000008136	9.26	8.29	Yes
GSMG0027182	---	down	1.96	2.85E-02	chr4(+):133140021-133141528	UCSC	---	AK086498	---	---	8.19	7.21	Yes
GSMG0018604	---	down	1.95	8.62E-03	chr18(+):75027961-75029335	UCSC	---	AK136399	---	---	7.71	6.75	No
GSMG0008394	---	down	1.95	1.42E-02	chr12(+):89986532-89988117	UCSC	---	AK043962	---	---	6.67	5.71	Yes
GSMG0032826	AB041803	down	1.95	4.48E-02	chr6(-):31165525-31218433	UCSC	cDNA sequence AB041803	AB041803	---	ENSMUSG00000044471	11.42	10.46	Yes
GSMG0023987	Gm14410	down	1.95	1.64E-02	chr2(-):177124003-177206445	UCSC	predicted gene 14410	AK078446	---	ENSMUSG00000078870	8.4	7.43	Yes
GSMG0002113	Adora1	down	1.94	1.63E-04	chr1(-):134199229-134235460	UCSC	adenosine A1 receptor	NM_001039510	11539	ENSMUSG00000042429	8.67	7.71	Yes
GSMG0016600	Psors1c2	down	1.94	1.71E-02	chr17(+):35533200-35534647	UCSC	psoriasis susceptibility 1 candidate 2 (human)	NM_020576	57390	ENSMUSG00000024409	9.27	8.32	Yes
GSMG0036547	---	down	1.94	3.63E-02	chr7(-):114111566-114113820	UCSC	---	AK037636	---	---	9.71	8.75	Yes
GSMG0024941	---	down	1.94	3.02E-02	chr3(+):131156974-131160248	UCSC	---	AK041666	---	---	8.26	7.31	No
GSMG0017127	Unc93a	down	1.93	2.30E-02	chr17(-):13108618-13131791	UCSC	unc-93 homolog A (C. elegans)	NM_199252	381058	ENSMUSG00000067049	7.95	7	Yes
GSMG0033979	Zfp667	down	1.93	4.56E-03	chr7(+):6286580-6307883	UCSC	zinc finger protein 667	NM_001024928	384763	ENSMUSG00000054893	8.14	7.19	No
GSMG0029612	---	down	1.93	5.20E-04	chr5(+):111392060-111395732	UCSC	---	AK138421	---	---	7.66	6.71	No
GSMG0023688	Gm14152	down	1.93	1.19E-02	chr2(-):151380650-151432404	UCSC	predicted gene 14152	AK006486	100125594	ENSMUSG00000087495	7.63	6.69	Yes
GSMG0018049	---	down	1.92	3.82E-02	chr18(+):9972993-9974015	UCSC	---	AK039978	---	---	8.3	7.36	Yes
GSMG0043476	Vmn2r117	down	1.92	6.62E-03	chr17(-):23459675-23479597	UCSC	vomeronasal 2, receptor 117	NM_001104581	619788	ENSMUSG00000091407	7.83	6.89	Yes
GSMG0001640	---	down	1.92	3.19E-04	chr1(-):60543939-60545713	UCSC	---	AK142022	---	---	6.34	5.4	No
GSMG0051106	1700016L21Rik	down	1.92	4.77E-02	chr1(+):80445931-80475660	UCSC	RIKEN cDNA 1700016L21 gene	AK006047	72208	---	6.52	5.58	Yes
GSMG0040633	---	down	1.92	2.81E-02	chr9(-):43097185-43101365	UCSC	---	AK172550	---	---	6.76	5.82	No
GSMG0033450	---	down	1.92	2.40E-03	chr6(-):98992681-98997596	UCSC	---	AK037473	---	---	10.07	9.13	No
GSMG0027427	---	down	1.92	1.58E-02	chr4(+):151557356-151558521	UCSC	---	AK015242	---	---	8.85	7.91	Yes
GSMG0019801	---	down	1.91	1.20E-02	chr19(+):53038798-53040214	UCSC	---	AK133125	---	---	7.97	7.03	No
GSMG0008103	Pnn	down	1.91	1.19E-02	chr12(+):59066919-59074017	UCSC	pinin	NM_008891	18949	ENSMUSG00000020994	9.11	8.17	No
GSMG0005619	Rnf135	down	1.91	6.48E-03	chr11(+):80183870-80199753	UCSC	ring finger protein 135	AK010429	71956	ENSMUSG00000020707	7.65	6.72	Yes
GSMG0047566	Mir9-1	down	1.91	3.65E-02	chr3(+):88215598-88215686	UCSC	microRNA 9-1	NR_029817	387133	---	7.6	6.66	No
GSMG0034145	---	down	1.91	2.24E-03	chr7(+):24268794-24270165	UCSC	---	AK153767	---	---	8.08	7.15	No
GSMG0024940	---	down	1.91	1.41E-02	chr3(+):131136785-131139042	UCSC	---	AK037608	---	---	8.13	7.19	No
GSMG0023782	Ndrg3	down	1.91	2.47E-02	chr2(-):156927342-156992111	UCSC	N-myc downstream regulated gene 3	NM_180956	29812	ENSMUSG00000027634	10.63	9.7	No
GSMG0002789	Gm10824	down	1.91	4.68E-03	chr10(+):22815747-22823330	UCSC	predicted gene 10824	AK033378	100038582	---	7.37	6.43	Yes
GSMG0019120	---	down	1.9	2.59E-02	chr18(-):72296241-72297521	UCSC	---	AK043048	---	---	7.24	6.31	No
GSMG0012653	Nudt15	down	1.9	5.46E-03	chr14(-):73518880-73548246	UCSC	nudix (nucleoside diphosphate linked moiety X)-type motif 15	AK040749	214254	ENSMUSG00000033405	9.82	8.89	No
GSMG0008053	---	down	1.9	4.19E-02	chr12(+):51970858-51972361	UCSC	---	AK082671	---	---	7.09	6.16	Yes
GSMG0007466	2410004I01Rik	down	1.9	2.23E-02	chr11(-):102958307-102964155	UCSC	RIKEN cDNA 2410004I01 gene	NR_037963	69739	ENSMUSG00000085338	7.35	6.42	Yes
GSMG0042425	Rhox1	down	1.9	7.40E-04	chrX(-):37213804-37222258	UCSC	reproductive homeobox 1	NM_001025084	385343	ENSMUSG00000071773	10.55	9.62	Yes
GSMG0041866	---	down	1.9	9.82E-04	chrX(+):75573452-75575266	UCSC	---	BC042783	---	---	7.99	7.07	Yes
GSMG0004781	Dgka	down	1.9	3.76E-02	chr10(-):128720136-128744855	UCSC	diacylglycerol kinase, alpha	JN832677	13139	ENSMUSG00000025357	11.74	10.82	No
GSMG0036887	Gm4559	down	1.9	3.80E-03	chr7(-):142273764-142274363	UCSC	predicted gene 4559	NM_001199309	100043627	ENSMUSG00000056885	9.65	8.72	Yes
GSMG0029207	---	down	1.9	1.30E-02	chr5(+):64804014-64805735	UCSC	---	AK049070	---	---	9.45	8.52	No
GSMG0028649	Clcn6	down	1.9	3.72E-02	chr4(-):148004265-148038813	UCSC	chloride channel 6	AK165362	26372	ENSMUSG00000029016	9.26	8.33	No
GSMG0028053	Ifne	down	1.9	7.04E-04	chr4(-):88879538-88880201	UCSC	interferon epsilon	NM_177348	230405	ENSMUSG00000045364	8.38	7.45	Yes
GSMG0016002	---	down	1.89	1.38E-03	chr16(-):86770227-86806518	UCSC	---	AK085322	---	---	6.83	5.91	No
GSMG0008615	Zfp386	down	1.89	3.88E-02	chr12(+):116047724-116063360	UCSC	zinc finger protein 386 (Kruppel-like)	AK220544	56220	ENSMUSG00000042063	9.16	8.24	No
GSMG0007306	4933428G20Rik	down	1.89	5.56E-03	chr11(-):97490783-97500341	UCSC	RIKEN cDNA 4933428G20 gene	AK016967	100141474	ENSMUSG00000047988	8.77	7.85	Yes
GSMG0006409	---	down	1.89	1.21E-02	chr11(-):22229043-22231957	UCSC	---	AK031188	---	---	10.58	9.66	Yes
GSMG0039419	D630033O11Rik	down	1.89	2.37E-04	chr9(+):43243598-43280075	UCSC	RIKEN cDNA D630033O11 gene	AK143856	235302	ENSMUSG00000095385	8.85	7.93	Yes
GSMG0000289	Gm15834	down	1.89	1.92E-02	chr1(+):58505143-58523230	UCSC	predicted gene 15834	AK053027	100504455	ENSMUSG00000085054	8.61	7.7	Yes
GSMG0022161	Gm11458	down	1.88	3.11E-02	chr2(+):164911354-164919953	UCSC	predicted gene 11458	AK141411	629777	ENSMUSG00000085436	7.99	7.08	Yes
GSMG0019332	Gpha2	down	1.88	4.82E-03	chr19(+):6226401-6227767	UCSC	glycoprotein hormone alpha 2	NM_130453	170458	ENSMUSG00000024784	7.83	6.92	Yes
GSMG0018689	Epc1	down	1.88	2.02E-03	chr18(-):6435953-6516108	UCSC	enhancer of polycomb homolog 1 (Drosophila)	NM_007935	13831	ENSMUSG00000024240	10.98	10.07	No
GSMG0014916	---	down	1.88	2.15E-02	chr16(+):33763948-33765631	UCSC	---	AK081900	---	---	9.17	8.25	No
GSMG0013621	Aqp2	down	1.88	4.56E-02	chr15(+):99579055-99584545	UCSC	aquaporin 2	AK142567	11827	ENSMUSG00000023013	8.14	7.23	No
GSMG0007055	---	down	1.88	1.51E-02	chr11(-):78707316-78741105	UCSC	---	AK136065	---	---	7.71	6.8	Yes
GSMG0005004	Nsg2	down	1.88	9.00E-03	chr11(+):32000427-32059204	UCSC	neuron specific gene family member 2	AK088499	18197	ENSMUSG00000020297	8.66	7.75	No
GSMG0038124	Tdrp	down	1.88	2.30E-03	chr8(-):13952009-13974777	UCSC	testis development related protein	NM_173744	72148	ENSMUSG00000050052	8.66	7.75	Yes
GSMG0036748	---	down	1.88	1.06E-02	chr7(-):130653128-130654392	UCSC	---	AK015786	---	---	9.02	8.11	Yes
GSMG0052294	Zfp850	down	1.88	1.05E-02	chr7(-):27984471-28014115	UCSC	zinc finger protein 850	NM_001254951	100043772	ENSMUSG00000096916	7.29	6.38	No
GSMG0052029	1700085B03Rik	down	1.88	9.40E-03	chr5(-):127256860-127281623	UCSC	RIKEN cDNA 1700085B03 gene	NR_038129	67351	ENSMUSG00000087104	6.68	5.77	Yes
GSMG0023712	Defb25	down	1.88	8.32E-03	chr2(-):152622356-152623053	UCSC	defensin beta 25	NM_001039122	654459	ENSMUSG00000074678	7.84	6.93	Yes
GSMG0051674	Olfr1008	down	1.87	4.96E-03	chr2(+):85689431-85690372	UCSC	olfactory receptor 1008	NM_146866	258866	ENSMUSG00000050603	7.44	6.54	Yes
GSMG0014563	---	down	1.87	1.02E-02	chr15(-):102607618-102608784	UCSC	---	AK017951	---	---	8.45	7.55	No
GSMG0010325	---	down	1.87	1.98E-02	chr13(-):13953217-13954339	UCSC	---	AK134220	---	---	6.89	5.99	Yes
GSMG0000047	Gm9947	down	1.87	3.28E-02	chr1(+):14756194-14776931	UCSC	predicted gene 9947	AK080352	---	ENSMUSG00000054493	8.08	7.18	Yes
GSMG0036642	Xpo6	down	1.87	6.98E-03	chr7(-):126164033-126168403	UCSC	exportin 6	AK088433	74204	ENSMUSG00000000131	7.54	6.64	No
GSMG0028803	---	down	1.87	2.26E-02	chr5(+):8669519-8673134	UCSC	---	AK035214	---	---	7.09	6.19	Yes
GSMG0028224	Nsun4	down	1.87	1.02E-02	chr4(-):116031772-116053878	UCSC	NOL1/NOP2/Sun domain family, member 4	NM_028142	72181	ENSMUSG00000028706	9.07	8.17	No
GSMG0016670	Gm5494	down	1.86	4.07E-02	chr17(+):40561507-40579549	UCSC	predicted gene 5494	AB306983	433107	ENSMUSG00000092043	9.86	8.97	Yes
GSMG0012881	Gpr183	down	1.86	6.48E-03	chr14(-):121952331-121965195	UCSC	G protein-coupled receptor 183	NM_183031	321019	ENSMUSG00000051212	12.23	11.33	No
GSMG0010596	---	down	1.86	9.18E-03	chr13(-):44010592-44018524	UCSC	---	AK146979	---	---	7.53	6.64	Yes
GSMG0010239	---	down	1.86	2.45E-02	chr13(+):114096297-114099563	UCSC	---	AK080903	---	---	7.79	6.9	No
GSMG0007781	---	down	1.86	1.88E-03	chr12(+):3732827-3734168	UCSC	---	AK158689	---	---	7.58	6.69	No
GSMG0042045	---	down	1.86	1.00E-03	chrX(+):112635302-112637000	UCSC	---	AK132044	---	---	8.51	7.61	Yes
GSMG0039333	Pate2	down	1.86	1.48E-03	chr9(+):35570283-35690318	UCSC	prostate and testis expressed 2	AK144787	330921	ENSMUSG00000074452	8.95	8.06	No

FAST DB STABLE ID	Gene Symbol	Regulation	Fold-Change	P-Value	Gene Coordinates (mm10)	UCSC Link	Gene Name	Representative Transcript ID	Entrez Gene ID	EnsEMBL ID	log^2 intensity of untreated	log^2 intensity of treated	Low-specificity probe included
GSMG0036266	4632427E13Rik	down	1.86	4.08E-03	chr7(-):92737658-92741460	UCSC	RIKEN cDNA 4632427E13 gene	AK084903	666737	ENSMUSG00000074024	9.62	8.72	No
GSMG0031378	---	down	1.86	2.70E-03	chr5(-):147428830-147429887	UCSC	---	AK147100	---	---	8.35	7.46	No
GSMG0028280	Olfr1333	down	1.86	2.46E-02	chr4(-):118829485-118830442	UCSC	olfactory receptor 1333	BC128000	258265	ENSMUSG00000073770	8.69	7.79	Yes
GSMG0015256	---	down	1.85	3.92E-02	chr16(+):85900747-85903992	UCSC	---	AK031656	---	---	7.86	6.97	Yes
GSMG0012880	---	down	1.85	4.23E-02	chr14(-):121936637-121939166	UCSC	---	AK154231	---	---	8.5	7.62	No
GSMG0012834	---	down	1.85	2.94E-03	chr14(-):106241684-106267936	UCSC	---	AK076329	---	---	6.15	5.26	No
GSMG0009510	Pom121l2	down	1.85	4.31E-02	chr13(+):21981191-21988734	UCSC	POM121 membrane glycoprotein-like 2 (rat)	AK133022	195236	ENSMUSG00000016982	7.71	6.83	Yes
GSMG0007926	---	down	1.85	1.24E-02	chr12(+):29843960-29845875	UCSC	---	AK047353	---	---	7.78	6.89	Yes
GSMG0042031	Gm6377	down	1.85	3.07E-02	chrX(+):109196756-109200445	UCSC	predicted gene 6377	NM_001037917	622976	ENSMUSG00000048621	10.7	9.81	No
GSMG0034609	---	down	1.85	3.75E-02	chr7(+):68276007-68279992	UCSC	---	AK089641	---	---	8.86	7.97	No
GSMG0003555	---	down	1.85	1.84E-02	chr10(+):116173155-116176059	UCSC	---	AK036092	---	---	8	7.11	Yes
GSMG0030372	---	down	1.85	1.08E-02	chr5(-):34335124-34337183	UCSC	---	AK078028	---	---	7.37	6.48	No
GSMG0051843	---	down	1.85	1.77E-02	chr2(+):176289943-176301588	UCSC	---	NR_040256	---	---	7.23	6.34	No
GSMG0017010	---	down	1.84	2.42E-02	chr17(+):86178607-86180253	UCSC	---	AK087230	---	---	8.12	7.24	No
GSMG0043474	Vmn2r116	down	1.84	3.74E-02	chr17(+):23384803-23401864	UCSC	vomeronasal 2, receptor 116	NM_001104580	619697	ENSMUSG00000090966	7.53	6.65	Yes
GSMG0000477	---	down	1.84	2.15E-02	chr1(+):82748387-82750085	UCSC	---	AK084632	---	---	8.43	7.55	No
GSMG0042418	Rpl39	down	1.84	2.38E-03	chrX(-):37082520-37085190	UCSC	ribosomal protein L39	NM_026055	67248	ENSMUSG00000079641	13.05	12.17	No
GSMG0034415	Klk1b11	down	1.84	3.63E-02	chr7(+):43995879-43999874	UCSC	kallikrein 1-related peptidase b11	NM_010640	16613	ENSMUSG00000044485	7.54	6.66	Yes
GSMG0029930	Ufsp1	down	1.84	1.42E-03	chr5(+):137294648-137295664	UCSC	UFM1-specific peptidase 1	BC087958	70240	ENSMUSG00000051502	7.77	6.89	Yes
GSMG0029782	A930024E05Rik	down	1.84	1.40E-02	chr5(+):122989354-122998339	UCSC	RIKEN cDNA A930024E05 gene	AK042918	109202	ENSMUSG00000056735	8.27	7.39	No
GSMG0023671	---	down	1.84	3.26E-02	chr2(-):150305528-150306207	UCSC	---	AK014002	---	---	7.54	6.66	No
GSMG0001977	---	down	1.83	1.74E-02	chr1(-):115668988-115684620	UCSC	---	AK043174	---	---	7.28	6.41	Yes
GSMG0016540	H2-DMb1	down	1.83	3.06E-03	chr17(+):34153190-34160229	UCSC	histocompatibility 2, class II, locus Mb1	NM_010387	14999	ENSMUSG00000079547	10.23	9.35	No
GSMG0012605	---	down	1.83	3.89E-02	chr14(-):70046106-70050846	UCSC	---	AK139460	---	---	7.31	6.44	No
GSMG0051427	Ang2	down	1.83	4.84E-03	chr14(-):51195486-51195923	UCSC	angiogenin, ribonuclease A family, member 2	NM_007449	11731	ENSMUSG00000047894	6.83	5.96	Yes
GSMG0010974	Aggf1	down	1.83	2.98E-03	chr13(-):95350690-95375358	UCSC	angiogenic factor with G patch and FHA domains 1	NM_025630	66549	ENSMUSG00000021681	9.91	9.04	No
GSMG0042723	Gm614	down	1.83	3.85E-02	chrX(-):101261376-101263993	UCSC	predicted gene 614	AK131750	245536	ENSMUSG00000090141	9.18	8.31	Yes
GSMG0042288	---	down	1.83	1.42E-03	chrX(+):169037738-169040432	UCSC	---	AK030523	---	---	7.56	6.68	No
GSMG0004559	---	down	1.83	1.31E-02	chr10(-):108545756-108547937	UCSC	---	AK086086	---	---	6.29	5.42	Yes
GSMG0039923	---	down	1.83	3.72E-02	chr9(+):94873272-94876513	UCSC	---	AK030836	---	---	8.52	7.65	No
GSMG0000332	Icos	down	1.83	2.47E-02	chr1(+):60977914-61000318	UCSC	inducible T cell co-stimulator	NM_017480	54167	ENSMUSG00000026009	9.52	8.64	No
GSMG0029526	---	down	1.83	1.54E-02	chr5(+):105584022-105586591	UCSC	---	AK077342	---	---	7.46	6.59	Yes
GSMG0027819	---	down	1.83	2.30E-03	chr4(-):55129708-55279483	UCSC	---	AK144643	---	---	7.52	6.65	No
GSMG0026257	---	down	1.83	1.87E-02	chr4(+):5734263-5735750	UCSC	---	AK084230	---	---	7.14	6.27	Yes
GSMG0016835	---	down	1.82	1.40E-03	chr17(+):57066155-57068110	UCSC	---	AK149380	---	---	7.69	6.82	No
GSMG0001617	---	down	1.82	3.32E-02	chr1(-):58579222-58581977	UCSC	---	AK138413	---	---	7.56	6.69	No
GSMG0011212	---	down	1.82	2.10E-02	chr14(+):8063260-8065245	UCSC	---	AK038810	---	---	7.73	6.87	No
GSMG0039589	---	down	1.82	1.37E-02	chr9(+):57584845-57586110	UCSC	---	AK159069	---	---	8.16	7.3	No
GSMG0032704	1700019G24Rik	down	1.82	1.29E-02	chr6(-):5963897-5977393	UCSC	RIKEN cDNA 1700019G24 gene	NR_040255	72249	ENSMUSG00000085416	8.57	7.7	Yes
GSMG0028913	---	down	1.82	2.44E-02	chr5(+):24129272-24132512	UCSC	---	AK051462	---	---	7.61	6.75	No
GSMG0043300	4930578G10Rik	down	1.82	1.71E-02	chr4(+):42735545-42767288	UCSC	RIKEN cDNA 4930578G10 gene	AK006581	---	ENSMUSG00000054885	8.69	7.83	No
GSMG0003346	Btbd11	down	1.82	1.01E-04	chr10(+):85386814-85660291	UCSC	BTB (POZ) domain containing 11	NM_028709	74007	ENSMUSG00000020042	7.59	6.73	Yes
GSMG0025596	---	down	1.82	1.84E-02	chr3(-):86856585-86857279	UCSC	---	AK148922	---	---	7.55	6.69	No
GSMG0023196	---	down	1.82	2.57E-04	chr2(-):92994518-92997582	UCSC	---	AK080575	---	---	8.03	7.16	No
GSMG0018005	Bambi	down	1.81	2.42E-02	chr18(+):3507933-3516404	UCSC	BMP and activin membrane-bound inhibitor	AK045192	68010	ENSMUSG00000024232	8.44	7.59	No
GSMG0009764	S1pr3	down	1.81	1.00E-02	chr13(+):51408618-51422797	UCSC	sphingosine-1-phosphate receptor 3	NM_010101	13610	ENSMUSG00000067586	10.31	9.45	No
GSMG0008865	Dtd2	down	1.81	4.18E-03	chr12(-):51988310-52006503	UCSC	D-tyrosyl-tRNA deacylase 2	AK016300	328092	ENSMUSG00000020956	8.17	7.31	No
GSMG0008831	---	down	1.81	7.72E-03	chr12(-):43810543-43844193	UCSC	---	AY512922	---	---	7.43	6.58	Yes
GSMG0051245	Gm11565	down	1.81	6.78E-03	chr11(+):99914751-99915671	UCSC	predicted gene 11565	NM_001126323	670550	ENSMUSG00000078256	6.39	5.54	Yes
GSMG0041319	Fbxw28	down	1.81	2.44E-03	chr9(-):109322886-109339659	UCSC	F-box and WD-40 domain protein 28	NM_001177419	668758	ENSMUSG00000079284	6.86	6.01	Yes
GSMG0037405	A230052G05Rik	down	1.81	6.03E-04	chr8(+):70863125-70865428	UCSC	RIKEN cDNA A230052G05 gene	AK079546	442796	ENSMUSG00000020887	7.27	6.42	Yes
GSMG0036699	1700008J07Rik	down	1.81	1.04E-03	chr7(-):127510438-127512869	UCSC	RIKEN cDNA 1700008J07 gene	NR_024331	629159	---	7.55	6.7	Yes
GSMG0029620	---	down	1.81	2.71E-02	chr5(+):112286126-112287449	UCSC	---	AK137193	---	---	8.61	7.75	No
GSMG0021631	---	down	1.8	3.42E-02	chr2(+):119576850-119578139	UCSC	---	AK044158	---	---	7.77	6.92	Yes
GSMG0018241	4930471G03Rik	down	1.8	4.30E-02	chr18(+):36106193-36109954	UCSC	RIKEN cDNA 4930471G03 gene	AK015550	74966	---	8.06	7.21	Yes
GSMG0016418	---	down	1.8	3.44E-02	chr17(+):27007188-27008365	UCSC	---	AK043439	---	---	7.88	7.03	Yes
GSMG0013066	---	down	1.8	2.14E-02	chr15(+):32049950-32051355	UCSC	---	AK034792	---	---	8.18	7.32	No
GSMG0011034	Marveld2	down	1.8	4.45E-02	chr13(-):100595957-100616971	UCSC	MARVEL (membrane-associating) domain containing 2	NM_001038602	218518	ENSMUSG00000021636	7.94	7.09	No
GSMG0009572	Prl3b1	down	1.8	6.62E-03	chr13(+):27242428-27249740	UCSC	prolactin family 3, subfamily b, member 1	AK005542	18776	ENSMUSG00000038891	6.75	5.91	Yes
GSMG0004531	---	down	1.8	5.80E-03	chr10(-):105298054-105299743	UCSC	---	AK053556	---	---	7.14	6.29	Yes
GSMG0037785	---	down	1.8	5.32E-03	chr8(+):108035362-108037138	UCSC	---	AK046498	---	---	6.86	6.02	No
GSMG0036077	---	down	1.8	1.91E-02	chr7(-):66615098-66619148	UCSC	---	AK144264	---	---	9.27	8.42	No
GSMG0035132	---	down	1.8	2.74E-03	chr7(+):122463438-122466160	UCSC	---	AK134188	---	---	8.27	7.43	No
GSMG0032053	---	down	1.8	1.44E-03	chr6(+):71554573-71556233	UCSC	---	AK138835	---	---	7.98	7.13	No
GSMG0028168	---	down	1.8	2.93E-02	chr4(-):108144211-108144977	UCSC	---	AK085433	---	---	6.41	5.56	Yes
GSMG0003198	Krtap12-1	down	1.8	3.02E-03	chr10(+):77720586-77721256	UCSC	keratin associated protein 12-1	NM_010670	16694	ENSMUSG00000069583	8.82	7.97	No
GSMG0026981	---	down	1.8	4.47E-02	chr4(+):117699074-117704020	UCSC	---	AK047342	---	---	12.64	11.8	Yes
GSMG0020490	---	down	1.79	1.37E-02	chr19(-):47252243-47254334	UCSC	---	AK044464	---	---	8.78	7.94	No
GSMG0014914	---	down	1.79	1.12E-02	chr16(+):33703054-33706374	UCSC	---	AK053934	---	---	8.49	7.65	No
GSMG0042585	Xlr3a // Xlr3c	down	1.79	8.28E-03	chrX(-):73086294-73265390	UCSC	X-linked lymphocyte-regulated 3A // X-linked lymphocyte-regulated 3C	BC127146	22445 // 22446	USG00000057836 // ENSMUSG00000	8.23	7.39	Yes
GSMG0041351	---	down	1.79	4.76E-02	chr9(-):111544004-111548941	UCSC	---	AK084079	---	---	8.4	7.57	Yes
GSMG0036340	---	down	1.79	1.68E-02	chr7(-):101525376-101538331	UCSC	---	AK158044	---	---	7.44	6.6	Yes
GSMG0035278	---	down	1.79	1.11E-02	chr7(+):134952120-134957878	UCSC	---	AK047297	---	---	7.47	6.63	Yes
GSMG0009489	---	down	1.78	7.42E-03	chr13(+):20499888-20501229	UCSC	---	BC029724	---	---	9.19	8.36	No
GSMG0039149	---	down	1.78	1.14E-02	chr9(+):13652678-13653860	UCSC	---	AK084324	---	---	8.6	7.76	No
GSMG0038532	Zfp866 // Zfp963	down	1.78	4.58E-02	chr8(-):69741639-69791180	UCSC	zinc finger protein 866 // zinc finger protein 963	AK043654	330788 // 620419	USG00000043090 // ENSMUSG00000	7.33	6.5	Yes
GSMG0052176	---	down	1.78	1.89E-02	chr7(+):98494222-98501831	UCSC	---	NM_001113379	---	---	8.06	7.23	No
GSMG0031912	---	down	1.78	4.03E-02	chr6(+):54309276-54313427	UCSC	---	AK047300	---	---	6.6	5.77	Yes
GSMG0029322	---	down	1.78	1.97E-02	chr5(+):78005749-78007476	UCSC	---	AK034550	---	---	7.33	6.5	No
GSMG0051814	Olfr1222	down	1.78	4.12E-03	chr2(-):89124794-89125729	UCSC	olfactory receptor 1222	NM_001011860	258177	ENSMUSG00000075101	6.86	6.03	Yes
GSMG0016233	Airn	down	1.77	2.79E-02	chr17(+):12748295-12751993	UCSC	antisense Igf2r RNA	AK032756	104103	ENSMUSG00000078247	8	7.18	Yes
GSMG0001083	---	down	1.77	2.96E-02	chr1(+):173631856-173633289	UCSC	---	AK156630	---	---	8.2	7.38	Yes
GSMG0010808	Gm10324	down	1.77	1.98E-02	chr13(+):66113453-66122836	UCSC	predicted gene 10324	NM_001177832	628709	ENSMUSG00000095909	8.17	7.35	Yes
GSMG0009277	---	down	1.77	1.82E-02	chr12(-):106094823-106098978	UCSC	---	AK135920	---	---	8.19	7.37	Yes
GSMG0039395	Olfr986	down	1.77	3.66E-02	chr9(+):40187079-40188094	UCSC	olfactory receptor 986	NM_146615	258608	ENSMUSG00000051095	7.05	6.22	Yes
GSMG0032262	---	down	1.77	2.28E-02	chr6(+):98121796-98248611	UCSC	---	AK160945	---	---	8.1	7.28	No
GSMG0028019	---	down	1.77	3.01E-02	chr4(-):86829143-86831994	UCSC	---	AK076247	---	---	7.75	6.92	Yes
GSMG0026358	---	down	1.77	2.78E-02	chr4(+):28926096-28929038	UCSC	---	AK009054	---	---	7.23	6.4	Yes
GSMG0003050	Tbata	down	1.77	6.86E-03	chr10(+):61171963-61188841	UCSC	thymus, brain and testes associated	NM_001017433	65971	ENSMUSG00000020096	7.2	6.37	Yes
GSMG0014705	---	down	1.76	3.86E-02	chr16(+):14317326-14318227	UCSC	---	AK076861	---	---	7.71	6.89	No
GSMG0011894	---	down	1.76	1.22E-03	chr14(+):103424905-103449867	UCSC	---	AK139344	---	---	8.94	8.13	No
GSMG0008258	---	down	1.76	4.32E-02	chr12(+):80115861-80119084	UCSC	---	AK005005	---	---	7.94	7.13	No
GSMG0007536	---	down	1.76	2.17E-02	chr11(-):107218136-107220553	UCSC	---	AK046794	---	---	9.14	8.33	Yes
GSMG0007356	Krtap3-3	down	1.76	2.53E-02	chr11(-):99550132-99550868	UCSC	keratin associated protein 3-3	AK009410	66380	ENSMUSG00000069722	7.8	6.98	No
GSMG0005839	---	down	1.76	1.48E-02	chr11(+):96628359-96636968	UCSC	---	AK039825	---	---	7.94	7.13	No
GSMG0042204	---	down	1.76	3.49E-02	chrX(+):152052781-152057419	UCSC	---	AK053658	---	---	8.41	7.6	No
GSMG0038854	---	down	1.76	4.02E-02	chr8(-):105809914-105812733	UCSC	---	AK054358	---	---	8.4	7.58	No
GSMG0033967	Zfp580	down	1.76	1.49E-02	chr7(+):5051532-5053722	UCSC	zinc finger protein 580	NM_026900	68992	ENSMUSG00000055633	8.13	7.32	Yes
GSMG0027164	---	down	1.76	8.02E-03	chr4(+):132276527-132279059	UCSC	---	AK137290	---	---	8.04	7.23	Yes
GSMG0013985	---	down	1.75	3.39E-02	chr15(-):55636390-55637192	UCSC	---	AK004311	---	---	10.08	9.27	No
GSMG0005324	Ubb	down	1.75	2.28E-03	chr11(+):62551471-62553213	UCSC	ubiquitin B	AK007273	22187	ENSMUSG00000019505	12.69	11.88	Yes
GSMG0043171	Ighd	down	1.75	3.44E-02	chr12(-):113409439-113409645	UCSC	immunoglobulin heavy constant delta	V00790	380797	---	9.63	8.83	Yes
GSMG0006567	---	down	1.75	4.67E-02	chr11(-):44892712-44893075	UCSC	---	AK043298	---	---	8.44	7.63	No
GSMG0006056	1700096J18Rik	down	1.75	8.78E-03	chr11(+):109346865-109353651	UCSC	RIKEN cDNA 1700096J18 gene	NR_027883	67927	---	8.1	7.29	Yes
GSMG0042872	C330013F16Rik	down	1.75	6.44E-03	chrX(-):139240226-139357738	UCSC	RIKEN cDNA C330013F16 gene	AK133643	78526	ENSMUSG00000086807	7.83	7.02	Yes
GSMG0039629	---	down	1.75	1.72E-02	chr9(+):59791491-59797675	UCSC	---	AK142882	---	---	7.97	7.16	No
GSMG0032522	---	down	1.75	4.70E-02	chr6(+):128843786-128846959	UCSC	---	AK089724	---	---	6.84	6.03	Yes
GSMG0030613	Txk	down	1.75	3.77E-02	chr5(-):72695978-72752777	UCSC	TXK tyrosine kinase	NM_001122754	22165	ENSMUSG00000054892	9.39	8.58	No
GSMG0028078	Mysm1	down	1.75	2.78E-02	chr4(-):94942040-94979102	UCSC	myb-like, SWIRM and MPN domains 1	NM_177239	320713	ENSMUSG00000062627	10.28	9.48	Yes
GSMG0027174	Eya3	down	1.75	2.81E-02	chr4(+):132676706-132679315	UCSC	eyes absent 3 homolog (Drosophila)	AK078716	14050	ENSMUSG00000028886	7.6	6.79	Yes
GSMG0023387	Dnajc17	down	1.75	1.62E-03	chr2(-):119172507-119208795	UCSC	DnaJ (Hsp40) homolog, subfamily C, member 17	NM_139139	69408	ENSMUSG00000034278	9.5	8.69	No
GSMG0020238	---	down	1.74	1.56E-02	chr19(-):27884623-27886985	UCSC	---	AK084929	---	---	7.51	6.71	No
GSMG0019791	Gsto2	down	1.74	4.42E-02	chr19(+):47865544-47886324	UCSC	glutathione S-transferase omega 2	AK077086	68214	ENSMUSG00000025069	7.61	6.82	Yes
GSMG0014881	---	down	1.74	2.39E-02	chr16(+):32303127-32304494	UCSC	---	AK017980	---	---	7.17	6.37	No
GSMG0013986	Sntb1	down	1.74	4.65E-02	chr15(-):55639154-55906952	UCSC	syntrophin, basic 1	U89997	20649	ENSMUSG00000060429	8.34	7.53	No
GSMG0013037	---	down	1.74	1.35E-02	chr15(+):27477162-27478738	UCSC	---	AK052900	---	---	8.43	7.63	No
GSMG0010405	Vmn1r189	down	1.74	1.13E-02	chr13(-):22101628-22102764	UCSC	vomeronasal 1 receptor 189	BC150715	252906	ENSMUSG00000061222	6.96	6.15	No
GSMG0001005	---	down	1.74	1.61E-02	chr1(+):165801315-165806324	UCSC	---	AK156059	---	---	8.61	7.8	No
GSMG0041998	---	down	1.74	8.50E-03	chrX(+):104508412-104512227	UCSC	---	AK052864	---	---	9.38	8.58	No
GSMG0052407	Defb39	down	1.74	4.93E-02	chr8(-):19052826-19064810	UCSC	defensin beta 39	NM_183038	360214	ENSMUSG00000061847	7.87	7.07	No
GSMG0058610	---	down	1.74	2.82E-03	chr7(+):136658013-136658039	UCSC	---	DQ726947	---	---	9.93	9.13	Yes
GSMG0032802	Impdh1	down	1.74	1.20E-03	chr6(-):29200437-29216351	UCSC	inosine 5'-phosphate dehydrogenase 1	AK047537	23917	ENSMUSG00000003500	9.38	8.58	No
GSMG0057842	---	down	1.74	3.17E-02	chr1(-):78967186-78967210	UCSC	---	AB347138	---	---	7.18	6.38	No
GSMG0031924	---	down	1.74	4.83E-02	chr6(+):54902422-54905728	UCSC	---	AK040700	---	---	7.71	6.92	No
GSMG0029765	---	down	1.74	4.21E-02	chr5(+):122165597-122167937	UCSC	---	AK084900	---	---	9.34	8.54	No
GSMG0028789	Akap9	down	1.74	1.01E-02	chr5(+):3928052-4080204	UCSC	A kinase (PRKA) anchor protein (yotiao) 9	NM_194462	100986	ENSMUSG00000040407	8.5	7.7	No
GSMG0027149	---	down	1.74	1.18E-02	chr4(+):130709699-130710490	UCSC	---	AK012752	---	---	8.25	7.45	No
GSMG0026225	Fpgt	down	1.74	2.17E-02	chr3(-):155084921-155093379	UCSC	fucose-1-phosphate guanylyltransferase	NM_029330	75540	ENSMUSG00000053870	8.28	7.48	No
GSMG0000844	)6G13Rik // Mir181b-1 // Mi	down	1.73	7.22E-04	chr1(+):137966455-137969853	UCSC	RIKEN cDNA A430106G13 gene // microRNA 181b-1 // microRNA 181a-1	AK053260	723890 // 735252	5458 // ENSMUSG00000065565 // ENS	6.98	6.19	No
GSMG0050983	Rbm20	down	1.73	1.36E-02	chr19(+):53677305-53867080	UCSC	RNA binding motif protein 20	NM_001170847	73713	ENSMUSG00000043639	9.41	8.62	Yes
GSMG0019384	Tut1	down	1.73	2.19E-02	chr19(+):8953840-8966209	UCSC	terminal uridylyl transferase 1, U6 snRNA-specific	BC025499	70044	ENSMUSG00000071645	8.64	7.85	No
GSMG0013975	Tnfrsf11b	down	1.73	4.37E-02	chr15(-):54250619-54278484	UCSC	tumor necrosis factor receptor superfamily, member 11b (osteoprotegerin)	NM_008764	18383	ENSMUSG00000063727	8.47	7.68	Yes
GSMG0012858	Dzip1	down	1.73	1.81E-02	chr14(-):118875520-118925387	UCSC	DAZ interacting protein 1	AK141247	66573	ENSMUSG00000042156	8.36	7.58	No
GSMG0009561	9330162012Rik	down	1.73	1.37E-02	chr13(+):24937401-24941417	UCSC	cDNA RIKEN 9330162012 gene	AK034200	---	ENSMUSG00000086448	7.87	7.08	No
GSMG0007535	Pitpnc1	down	1.73	2.62E-03	chr11(-):107207892-107470748	UCSC	phosphatidylinositol transfer protein, cytoplasmic 1	NM_145823	71795	ENSMUSG00000040430	10.07	9.27	No
GSMG0000712	---	down	1.73	2.40E-02	chr1(+):127431375-127433882	UCSC	---	AK037781	---	---	7.18	6.4	Yes
GSMG0029172	---	down	1.73	2.80E-04	chr5(+):53905165-53909218	UCSC	---	AK084184	---	---	6.98	6.19	Yes
GSMG0024543	---	down	1.73	2.48E-02	chr3(+):89337850-89347834	UCSC	---	AK132440	---	---	8.04	7.25	Yes
GSMG0023934	---	down	1.73	8.46E-03	chr2(-):168930984-168932568	UCSC	---	AK138895	---	---	8.23	7.44	Yes
GSMG0019786	Gm19557	down	1.72	2.99E-02	chr19(+):47512981-47516578	UCSC	predicted gene, 19557	AK158434	100503104	ENSMUSG00000097990	7.34	6.56	Yes
GSMG0017924	Thada	down	1.72	1.42E-02	chr17(-):84190056-84466208	UCSC	thyroid adenoma associated	NM_183021	240174	ENSMUSG00000024251	9.71	8.93	No
GSMG0017012	---	down	1.72	2.10E-02	chr17(+):86216464-86495611	UCSC	---	AK137218	---	---	7.41	6.64	No
GSMG0012379	---	down	1.72	1.10E-03	chr14(-):52254395-52257725	UCSC	---	AK048353	---	---	8.39	7.6	No
GSMG0011520	Olfr745	down	1.72	4.03E-02	chr14(+):50642195-50643369	UCSC	olfactory receptor 745	NM_146299	258296	ENSMUSG00000050028	6.25	5.47	Yes
GSMG0008868	---	down	1.72	3.72E-03	chr12(-):52475739-52480610	UCSC	---	AK138501	---	---	7.15	6.36	Yes
GSMG0008189	---	down	1.72	2.08E-02	chr12(+):73985363-73989065	UCSC	---	AK036274	---	---	7.6	6.82	No
GSMG0007733	---	down	1.72	4.19E-04	chr11(-):120429914-120432237	UCSC	---	AK136500	---	---	7.9	7.12	No
GSMG0007406	---	down	1.72	4.20E-02	chr11(-):100843276-100846824	UCSC	---	AK143598	---	---	9.2	8.42	No
GSMG0005575	---	down	1.72	5.44E-03	chr11(+):77989483-77990653	UCSC	---	AK134465	---	---	8.56	7.78	No
GSMG0039422	---	down	1.72	1.56E-03	chr9(+):43771586-43772772	UCSC	---	AK140510	---	---	7.41	6.62	Yes
GSMG0033413	Lrig1	down	1.72	2.99E-02	chr6(-):94604529-94700145	UCSC	leucine-rich repeats and immunoglobulin-like domains 1	NM_008377	16206	ENSMUSG00000030029	8.08	7.29	No
GSMG0033108	Il12rb2	down	1.72	1.01E-02	chr6(-):67291318-67376139	UCSC	interleukin 12 receptor, beta 2	U64199	16162	ENSMUSG00000018341	7.48	6.7	No
GSMG0032482	Nop2	down	1.72	2.05E-02	chr6(+):125131883-125144753	UCSC	NOP2 nucleolar protein	NM_138747	110109	ENSMUSG00000038279	9.11	8.33	No
GSMG0030256	Gm10472	down	1.72	2.52E-02	chr5(-):24449608-24451710	UCSC	predicted gene 10472	AK153723	---	ENSMUSG00000073129	7.85	7.07	Yes
GSMG0029394	Alb	down	1.72	4.56E-03	chr5(+):90460889-90476603	UCSC	albumin	NM_009654	11657	ENSMUSG00000029368	8.41	7.63	Yes
GSMG0024312	4931419H13Rik	down	1.72	1.82E-03	chr3(+):55055240-55084002	UCSC	RIKEN cDNA 4931419H13 gene	AK016464	70970	---	7.49	6.71	Yes
GSMG0000226	C230029F24Rik	down	1.72	4.72E-02	chr1(+):49244615-49340431	UCSC	RIKEN cDNA C230029F24 gene	AK082253	442837	ENSMUSG00000051616	7.26	6.48	No
GSMG0019689	Frat1	down	1.71	1.48E-03	chr19(+):41829970-41832583	UCSC	frequently rearranged in advanced T cell lymphomas	NM_008043	14296	ENSMUSG00000067199	8.88	8.1	No
GSMG0016979	---	down	1.71	2.62E-04	chr17(+):83463951-83466020	UCSC	---	AK031194	---	---	9.66	8.88	Yes
GSMG0012698	Tnfsf11	down	1.71	1.94E-03	chr14(-):78277445-78308044	UCSC	tumor necrosis factor (ligand) superfamily, member 11	AK159997	21943	ENSMUSG00000022015	8.52	7.75	Yes
GSMG0012373	Olfr221	down	1.71	1.76E-02	chr14(-):52035082-52036414	UCSC	olfactory receptor 221	BC151091	258420	ENSMUSG00000044286	7.33	6.56	No
GSMG0011877	---	down	1.71	2.76E-02	chr14(+):99154760-99158461	UCSC	---	AK083407	---	---	7.55	6.77	No
GSMG0010291	---	down	1.71	3.04E-04	chr13(-):8833099-8834372	UCSC	---	AK139420	---	---	7.9	7.12	No
GSMG0009416	---	down	1.71	3.49E-02	chr13(+):9107703-9110299	UCSC	---	AK141795	---	---	9.38	8.61	No
GSMG0004910	---	down	1.71	5.58E-03	chr11(+):18095985-18103387	UCSC	---	AK077028	---	---	7.16	6.38	Yes
GSMG0038924	---	down	1.71	1.37E-02	chr8(-):111217167-111219921	UCSC	---	AK138012	---	---	8.04	7.26	No
GSMG0051188	Gm3238	down	1.71	3.77E-04	chr10(-):77770633-77771325	UCSC	predicted gene 3238	NM_001101630	100041261	ENSMUSG00000095817	8.78	8	Yes
GSMG0035197	---	down	1.71	4.96E-02	chr7(+):127506331-127508037	UCSC	---	AK154441	---	---	7.26	6.48	No
GSMG0052061	Vmn1r54	down	1.71	2.12E-02	chr6(+):90269106-90270053	UCSC	vomeronasal 1 receptor 54	NM_053224	113851	ENSMUSG00000047203	7.15	6.38	Yes
GSMG0029770	Gpn3	down	1.71	1.62E-02	chr5(+):122371875-122382895	UCSC	GPN-loop GTPase 3	AK042405	68080	ENSMUSG00000029464	9.3	8.52	No
GSMG0003408	---	down	1.71	5.61E-04	chr10(+):93317402-93322725	UCSC	---	AK043075	---	---	7.3	6.53	No
GSMG0023465	---	down	1.71	1.07E-02	chr2(-):125207529-125217513	UCSC	---	AK085539	---	---	7.6	6.82	No
GSMG0021436	2810002D19Rik	down	1.7	5.80E-03	chr2(+):94406151-94412470	UCSC	RIKEN cDNA 2810002D19 gene	AK148028	66457	ENSMUSG00000045464	7.21	6.45	No
GSMG0020669	Gm10851	down	1.7	2.41E-02	chr2(+):11521706-11530834	UCSC	predicted gene 10851	AK153745	100038528	---	6.79	6.02	No
GSMG0017957	Calm2	down	1.7	1.42E-02	chr17(-):87433401-87446935	UCSC	calmodulin 2	NM_007589	12314	ENSMUSG00000036438	11.8	11.04	No
GSMG0017350	Tff2	down	1.7	6.02E-03	chr17(-):31141053-31144284	UCSC	trefoil factor 2 (spasmolytic protein 1)	AK003093	21785	ENSMUSG00000024028	7.61	6.85	Yes
GSMG0017089	Rnaset2a	down	1.7	1.30E-03	chr17(-):8128316-8148097	UCSC	ribonuclease T2A	BC089534	100037283	ENSMUSG00000095687	11.41	10.64	Yes
GSMG0015824	Cep97	down	1.7	9.98E-03	chr16(-):55899888-55934856	UCSC	centrosomal protein 97	NM_028815	74201	ENSMUSG00000022604	9.53	8.77	No
GSMG0006517	---	down	1.7	2.08E-02	chr11(-):35904629-35907915	UCSC	---	AK136623	---	---	7.83	7.06	Yes
GSMG0039469	---	down	1.7	2.81E-02	chr9(+):46028235-46033470	UCSC	---	AK084915	---	---	9.46	8.69	Yes
GSMG0035914	Fgf21	down	1.7	1.50E-03	chr7(-):45613886-45615490	UCSC	fibroblast growth factor 21	AK007574	56636	ENSMUSG00000030827	9.61	8.85	Yes
GSMG0034223	Zfp60	down	1.7	3.10E-02	chr7(+):27731388-27751689	UCSC	zinc finger protein 60	AK149305	22718	ENSMUSG00000037640	7.38	6.61	No
GSMG0029486	---	down	1.7	4.53E-02	chr5(+):101264497-101268395	UCSC	---	AK079779	---	---	6.94	6.17	Yes
GSMG0028402	Lck	down	1.7	3.41E-02	chr4(-):129548344-129573641	UCSC	lymphocyte protein tyrosine kinase	NM_001162433	16818	ENSMUSG00000000409	11.09	10.33	No
GSMG0027747	---	down	1.7	4.30E-02	chr4(-):44144286-44146530	UCSC	---	AK035020	---	---	7.73	6.97	Yes
GSMG0025031	---	down	1.7	4.40E-02	chr3(+):141491536-141491993	UCSC	---	AK042906	---	---	8.16	7.39	Yes

FAST DB STABLE ID	Gene Symbol	Regulation	Fold-Change	P-Value	Gene Coordinates (mm10)	UCSC Link	Gene Name	Representative Transcript ID	Entrez Gene ID	EnsEMBL ID	log^2 intensity of untreated	log^2 intensity of treated	Low-specificity probe included
GSMG0020728	1810010K12Rik	down	1.69	2.76E-03	chr2(+):19452470-19454995	UCSC	RIKEN cDNA 1810010K12 gene	AK007425	---	ENSMUSG00000056329	9.58	8.82	Yes
GSMG0019554	Smarca2	down	1.69	2.19E-02	chr19(+):26605160-26778321	UCSC	SWI/SNF related, matrix associated, actin dependent regulator of chromatin,	NM_011416	67155	ENSMUSG00000024921	8.95	8.19	No
GSMG0018843	Spata24	down	1.69	1.56E-03	chr18(-):35656690-35662186	UCSC	spermatogenesis associated 24	NM_029485	71242	ENSMUSG00000024352	7.79	7.03	Yes
GSMG0015697	---	down	1.69	9.40E-03	chr16(-):35352660-35357576	UCSC	---	AK048116	---	---	7.76	7.01	Yes
GSMG0014741	B830017H08Rik	down	1.69	4.00E-02	chr16(+):17833118-17835019	UCSC	RIKEN cDNA B830017H08 gene	NR_027959	433004	---	8.27	7.51	Yes
GSMG0011762	Egr3	down	1.69	3.05E-02	chr14(+):70077445-70080157	UCSC	early growth response 3	NM_018781	13655	ENSMUSG00000033730	8.93	8.18	No
GSMG0001323	---	down	1.69	2.21E-04	chr1(-):13177462-13179714	UCSC	---	AK034603	---	---	8.52	7.76	No
GSMG0010954	---	down	1.69	5.98E-03	chr13(-):93155515-93158666	UCSC	---	AK051768	---	---	9.89	9.13	No
GSMG0009798	---	down	1.69	3.12E-02	chr13(+):55109795-55112319	UCSC	---	AK014495	---	---	7.33	6.57	No
GSMG0031747	Trbv1 // Trbv13-2	down	1.69	4.33E-02	chr6(+):40891227-41548356	UCSC	T cell receptor beta, variable 1 // T cell receptor beta, variable 13-2	AK138971	100124675 // 621968	USG00000076461 // ENSMUSG00000	8.43	7.68	No
GSMG0007742	Arhgdia	down	1.68	2.32E-05	chr11(-):120577235-120581620	UCSC	Rho GDP dissociation inhibitor (GDI) alpha	NM_133796	192662	ENSMUSG00000025132	11.95	11.2	No
GSMG0052724	Rps27a	down	1.68	2.84E-03	chr11(-):29545842-29548040	UCSC	ribosomal protein S27A	NM_024277	78294	ENSMUSG00000020460	11.63	10.88	No
GSMG0004653	Dyrk2	down	1.68	8.20E-04	chr10(-):118858084-118869149	UCSC	dual-specificity tyrosine-(Y)-phosphorylation regulated kinase 2	AK149371	69181	ENSMUSG00000028630	8.69	7.94	No
GSMG0039739	---	down	1.68	3.95E-02	chr9(+):69180086-69180817	UCSC	---	AK084450	---	---	7.02	6.27	Yes
GSMG0037067	Defa25	down	1.68	3.81E-02	chr8(+):21084442-21085285	UCSC	defensin, alpha, 25	NM_007849	13236	ENSMUSG00000094687	9.29	8.54	No
GSMG0031846	Gimap4	down	1.68	1.97E-02	chr6(+):48684578-48692066	UCSC	GTPase, IMAP family member 4	NM_175048	107526	ENSMUSG00000054435	12.06	11.31	No
GSMG0029268	Nipal1	down	1.68	1.96E-02	chr5(+):72647795-72671078	UCSC	NIPA-like domain containing 1	NM_001081205	70701	ENSMUSG00000067219	7.89	7.14	No
GSMG0025691	Lce1f	down	1.68	2.93E-02	chr3(-):92718685-92720351	UCSC	late cornified envelope 1F	AK003518	67828	ENSMUSG00000042124	7.17	6.43	Yes
GSMG0025136	---	down	1.68	5.10E-03	chr3(+):158072280-158074869	UCSC	---	AK155156	---	---	7.24	6.5	No
GSMG0024717	---	down	1.68	2.79E-02	chr3(+):99144602-99148954	UCSC	---	AK083626	---	---	7.97	7.22	No
GSMG0023970	Ppp4r1l-ps	down	1.68	2.08E-03	chr2(-):173579347-173659541	UCSC	protein phosphatase 4, regulatory subunit 1-like, pseudogene	AK164611	100043911	ENSMUSG00000055897	7.26	6.51	Yes
GSMG0023454	Bambi-ps1	down	1.68	7.90E-03	chr2(-):122466583-122467797	UCSC	BMP and activin membrane-bound inhibitor, pseudogene (Xenopus laevis)	NR_027919	81913	ENSMUSG00000081219	8.19	7.44	No
GSMG0022363	---	down	1.68	1.69E-02	chr2(-):6255946-6257268	UCSC	---	AK043615	---	---	7.45	6.7	Yes
GSMG0019418	A430093F15Rik	down	1.67	3.40E-02	chr19(+):10748037-10756771	UCSC	RIKEN cDNA A430093F15 gene	AK137926	403202	ENSMUSG00000067577	6.98	6.24	No
GSMG0018760	---	down	1.67	3.64E-02	chr18(-):20876125-20880777	UCSC	---	AK038081	---	---	7.93	7.19	No
GSMG0016995	Ppm1b	down	1.67	7.96E-03	chr17(+):84956739-85023991	UCSC	protein phosphatase 1B, magnesium dependent, beta isoform	AK076923	19043	ENSMUSG00000061130	10.79	10.04	No
GSMG0013856	---	down	1.67	4.06E-02	chr15(-):34481707-34483829	UCSC	---	AK040056	---	---	7.69	6.95	Yes
GSMG0012854	Sox21	down	1.67	1.04E-03	chr14(-):118233243-118236431	UCSC	SRY-box containing gene 21	BC069176	223227	ENSMUSG00000061517	8.87	8.12	Yes
GSMG0051404	---	down	1.67	1.83E-02	chr14(+):53665939-53668335	UCSC	---	NM_001205242	---	---	7.14	6.4	Yes
GSMG0052751	---	down	1.67	1.26E-03	chr13(+):120239110-120241367	UCSC	---	AF067063	---	---	7.3	6.55	Yes
GSMG0009731	---	down	1.67	1.83E-02	chr13(+):48031772-48042471	UCSC	---	AK076387	---	---	6.97	6.23	Yes
GSMG0009191	---	down	1.67	6.64E-03	chr12(-):99190085-99191480	UCSC	---	BC029185	---	---	10.14	9.41	No
GSMG0005110	Zfp62	down	1.67	3.46E-02	chr11(+):49178061-49218815	UCSC	zinc finger protein 62	L36315	22720	ENSMUSG00000046311	10.97	10.23	No
GSMG0041150	Gm16794	down	1.67	1.50E-02	chr9(-):95942049-95959393	UCSC	predicted gene, 16794	AK040392	100504734	ENSMUSG00000097777	7.15	6.41	Yes
GSMG0039193	---	down	1.67	4.28E-02	chr9(+):20385233-20387728	UCSC	---	AK076610	---	---	7.42	6.68	Yes
GSMG0037927	Gm22	down	1.67	4.68E-03	chr8(+):122269569-122272646	UCSC	predicted gene 22	AK122564	195209	ENSMUSG00000043903	8.34	7.6	Yes
GSMG0037355	Zfp930	down	1.67	2.49E-02	chr8(+):69209046-69230539	UCSC	zinc finger protein 930	NM_001013379	234358	ENSMUSG00000059897	10.1	9.36	No
GSMG0033746	Klrk1	down	1.67	4.50E-02	chr6(-):129594491-130061533	UCSC	killer cell lectin-like receptor subfamily K, member 1	AF054819	27007	ENSMUSG00000030149	7.11	6.37	No
GSMG0031330	---	down	1.67	1.98E-02	chr5(-):143511485-143513335	UCSC	---	AK155775	---	---	8.15	7.42	No
GSMG0028637	---	down	1.67	3.22E-02	chr4(-):145350132-145353103	UCSC	---	AK082378	---	---	7.69	6.95	No
GSMG0026209	---	down	1.67	4.68E-02	chr3(-):153309370-153312747	UCSC	---	AK041114	---	---	7.95	7.21	No
GSMG0050948	Safb	down	1.66	7.54E-03	chr17(+):56584982-56606294	UCSC	scaffold attachment factor B	NM_001163300	224903	ENSMUSG00000071054	9.87	9.14	No
GSMG0014477	---	down	1.66	4.16E-02	chr15(-):98760138-98762052	UCSC	---	AK089904	---	---	8.12	7.4	Yes
GSMG0051391	Fam170b	down	1.66	7.64E-03	chr14(+):32833962-32836789	UCSC	family with sequence similarity 170, member B	NM_001164485	105511	ENSMUSG00000078127	9.82	9.09	Yes
GSMG0000981	Selp	down	1.66	7.46E-03	chr1(+):164115264-164150026	UCSC	selectin, platelet	NM_011347	20344	ENSMUSG00000026580	8.6	7.87	Yes
GSMG0008023	---	down	1.66	3.60E-02	chr12(+):41161081-41163199	UCSC	---	AK044816	---	---	8.17	7.44	Yes
GSMG0007505	---	down	1.66	5.10E-03	chr11(-):106177690-106180098	UCSC	---	AK035456	---	---	7.9	7.18	No
GSMG0041769	Brs3	down	1.66	1.05E-02	chrX(+):57043073-57048758	UCSC	bombesin-like receptor 3	AK038616	12209	ENSMUSG00000031130	7.72	6.99	Yes
GSMG0041184	Gm1123	down	1.66	2.44E-03	chr9(-):99006793-99035717	UCSC	predicted gene 1123	AB262662	382097	ENSMUSG00000044860	8.43	7.7	Yes
GSMG0040564	9230110F15Rik	down	1.66	1.26E-02	chr9(-):35838329-35844152	UCSC	RIKEN cDNA 9230110F15 gene	AK020329	77080	ENSMUSG00000047980	6.24	5.51	Yes
GSMG0040519	---	down	1.66	4.73E-02	chr9(-):29696078-29698013	UCSC	---	AK159021	---	---	7.55	6.81	Yes
GSMG0040318	---	down	1.66	8.28E-03	chr9(-):7861446-7864292	UCSC	---	AK080183	---	---	10.6	9.87	Yes
GSMG0040234	Rpsa	down	1.66	1.44E-02	chr9(+):120127766-120132369	UCSC	ribosomal protein SA	NM_011029	16785	ENSMUSG00000032518	10.18	9.44	No
GSMG0036966	---	down	1.66	6.14E-03	chr8(+):4527035-4529608	UCSC	---	AK043951	---	---	7.66	6.93	No
GSMG0035790	---	down	1.66	3.82E-03	chr7(-):34362701-34365331	UCSC	---	AK135571	---	---	8.53	7.8	No
GSMG0034336	---	down	1.66	1.78E-02	chr7(+):34389731-34393612	UCSC	---	AK044863	---	---	8.86	8.13	No
GSMG0032394	Cacna2d4	down	1.66	3.42E-03	chr6(+):119236526-119352407	UCSC	calcium channel, voltage-dependent, alpha 2/delta subunit 4	NM_001033382	319734	ENSMUSG00000041460	8.03	7.3	Yes
GSMG0030395	D5Ertd579e	down	1.66	4.14E-02	chr5(-):36600492-36696025	UCSC	DNA segment, Chr 5, ERATO Doi 579, expressed	NM_001081232	320661	ENSMUSG00000029190	8.72	7.99	Yes
GSMG0029243	---	down	1.66	4.00E-03	chr5(+):66969429-66971585	UCSC	---	AK143041	---	---	7.27	6.54	Yes
GSMG0028550	Pla2g2f	down	1.66	1.24E-02	chr4(-):138750533-138757598	UCSC	phospholipase A2, group IIF	NM_012045	26971	ENSMUSG00000028749	8.15	7.42	Yes
GSMG0026817	---	down	1.66	1.68E-03	chr4(+):102975870-102977440	UCSC	---	AK047704	---	---	6.47	5.74	Yes
GSMG0026256	---	down	1.66	1.06E-02	chr4(+):5645706-5648864	UCSC	---	AK084183	---	---	9.41	8.68	Yes
GSMG0024500	Sh2d2a	down	1.66	4.71E-02	chr3(+):87846749-87855722	UCSC	SH2 domain protein 2A	NM_021309	27371	ENSMUSG00000028071	9.31	8.57	No
GSMG0023429	Lcmt2	down	1.66	5.32E-03	chr2(-):121128307-121140379	UCSC	leucine carboxyl methyltransferase 2	AK038626	329504	ENSMUSG00000074890	9.28	8.55	No
GSMG0022162	---	down	1.65	3.78E-03	chr2(+):164911888-164912933	UCSC	---	AK132110	---	---	7.84	7.11	Yes
GSMG0016936	Crim1	down	1.65	2.79E-02	chr17(+):78200248-78376592	UCSC	cysteine rich transmembrane BMP regulator 1 (chordin like)	NM_015800	50766	ENSMUSG00000024074	9.51	8.79	No
GSMG0016885	C030034I22Rik	down	1.65	3.80E-02	chr17(+):69416447-69421632	UCSC	RIKEN cDNA C030034I22 gene	AK147961	77533	ENSMUSG00000073374	7.85	7.13	No
GSMG0050949	Fem1a	down	1.65	5.24E-03	chr17(+):56256793-56263608	UCSC	feminization 1 homolog a (C. elegans)	NM_010192	14154	ENSMUSG00000043683	8.7	7.98	No
GSMG0015374	---	down	1.65	3.56E-03	chr16(-):4170776-4173636	UCSC	---	AK157787	---	---	9.03	8.32	No
GSMG0014041	---	down	1.65	3.21E-02	chr15(-):63946904-63948177	UCSC	---	AK041551	---	---	10.08	9.36	No
GSMG0007689	Tha1	down	1.65	1.58E-02	chr11(-):117867949-117873526	UCSC	threonine aldolase 1	NM_027919	71776	ENSMUSG00000017713	7.59	6.87	Yes
GSMG0041248	Glyctk	down	1.65	2.41E-02	chr9(-):106152858-106158138	UCSC	glycerate kinase	NM_174846	235582	ENSMUSG00000020258	8.09	7.36	Yes
GSMG0037911	Irf8	down	1.65	1.65E-02	chr8(+):120736356-120756694	UCSC	interferon regulatory factor 8	AK036488	15900	ENSMUSG00000041515	10.61	9.88	No
GSMG0037555	Fbxw9	down	1.65	6.40E-03	chr8(+):85060052-85067124	UCSC	F-box and WD-40 domain protein 9	AK156709	68628	ENSMUSG00000008167	8.82	8.09	Yes
GSMG0037240	Sorbs2	down	1.65	2.17E-02	chr8(+):45507788-45827906	UCSC	sorbin and SH3 domain containing 2	NM_172752	100861925	ENSMUSG00000031626	6.68	5.95	Yes
GSMG0037037	Gm10699	down	1.65	4.51E-02	chr8(+):14090461-14092590	UCSC	predicted gene 10699	AK134595	---	ENSMUSG00000074461	7.78	7.06	Yes
GSMG0036929	Ppfia1	down	1.65	1.13E-02	chr7(-):144476755-144553729	UCSC	protein tyrosine phosphatase, receptor type, f polypeptide (PTPRF), interacti	NM_001195086	233977	ENSMUSG00000037519	9.02	8.3	No
GSMG0003618	---	down	1.65	1.06E-02	chr10(+):122689124-122691645	UCSC	---	AK137773	---	---	8.63	7.91	No
GSMG0029868	---	down	1.65	3.32E-02	chr5(+):130102590-130107012	UCSC	---	AK136829	---	---	8.02	7.3	No
GSMG0029091	Jakmip1	down	1.65	3.49E-02	chr5(+):37047429-37125298	UCSC	janus kinase and microtubule interacting protein 1	NM_178394	76071	ENSMUSG00000063646	9.25	8.54	No
GSMG0028742	Ski	down	1.65	2.13E-02	chr4(-):155154076-155222535	UCSC	ski sarcoma viral oncogene homolog (avian)	NM_011385	20481	ENSMUSG00000029050	10.8	10.08	Yes
GSMG0024665	---	down	1.65	4.66E-03	chr3(+):95855964-95861485	UCSC	---	AK133135	---	---	8.88	8.16	Yes
GSMG0024507	Gpatch4	down	1.65	4.34E-02	chr3(+):88043107-88055993	UCSC	G patch domain containing 4	NM_025663	66614	ENSMUSG00000028069	7.65	6.93	Yes
GSMG0002840	---	down	1.65	5.00E-02	chr10(+):28812042-28813434	UCSC	---	AK030930	---	---	7.24	6.52	Yes
GSMG0020100	Cd6	down	1.64	4.16E-02	chr19(-):10789339-10830060	UCSC	CD6 antigen	NM_009852	12511	ENSMUSG00000024670	9.83	9.12	Yes
GSMG0019777	---	down	1.64	1.24E-02	chr19(+):46882357-46883435	UCSC	---	AK020893	---	---	8.34	7.63	Yes
GSMG0019027	Grpel2	down	1.64	1.34E-02	chr18(-):61710346-61726331	UCSC	GrpE-like 2, mitochondrial	AK082983	17714	ENSMUSG00000024580	10	9.29	No
GSMG0018215	---	down	1.64	1.76E-02	chr18(+):34764549-34766268	UCSC	---	AK144047	---	---	7.19	6.47	No
GSMG0017374	Wiz	down	1.64	2.29E-02	chr17(-):32354050-32389439	UCSC	widely-interspaced zinc finger motifs	NR_033525	22404	ENSMUSG00000024050	7.77	7.06	No
GSMG0014961	Gpr156	down	1.64	1.22E-02	chr16(+):37916451-38007527	UCSC	G protein-coupled receptor 156	AK141990	239845	ENSMUSG00000046961	7.36	6.65	Yes
GSMG0014906	---	down	1.64	1.24E-02	chr16(+):33259366-33261583	UCSC	---	AK078981	---	---	7.45	6.74	No
GSMG0013902	---	down	1.64	1.88E-03	chr15(-):39003288-39006186	UCSC	---	AK087598	---	---	7.45	6.74	Yes
GSMG0011559	---	down	1.64	3.44E-03	chr14(+):53538189-54169705	UCSC	---	AK137945	---	---	7.74	7.03	No
GSMG0009001	Gphb5	down	1.64	1.74E-03	chr12(-):75411720-75416782	UCSC	glycoprotein hormone beta 5	AK078727	217674	ENSMUSG00000048982	8.91	8.2	Yes
GSMG0008351	2310044G17Rik	down	1.64	3.20E-02	chr12(+):86947042-86965362	UCSC	RIKEN cDNA 2310044G17 gene	NR_033623	217732	ENSMUSG00000034157	9.09	8.38	No
GSMG0000836	---	down	1.64	2.51E-02	chr1(+):137898594-137899729	UCSC	---	AK040011	---	---	8.52	7.81	No
GSMG0040397	Olfr828	down	1.64	2.19E-02	chr9(-):18815201-18816563	UCSC	olfactory receptor 828	BC146590	258598	ENSMUSG00000078116	6.2	5.49	No
GSMG0051092	5830418K08Rik	down	1.64	1.26E-02	chr9(-):15316914-15357788	UCSC	RIKEN cDNA 5830418K08 gene	NM_176976	319675	ENSMUSG00000046111	9.01	8.29	Yes
GSMG0039547	Dnaja4	down	1.64	3.69E-02	chr9(+):54699384-54716317	UCSC	DnaJ (Hsp40) homolog, subfamily A, member 4	NM_021422	58233	ENSMUSG00000032285	8.42	7.71	Yes
GSMG0037844	Wwox	down	1.64	5.16E-03	chr8(+):114439652-115352712	UCSC	WW domain-containing oxidoreductase	NM_019573	80707	ENSMUSG00000004637	8.74	8.03	No
GSMG0033858	St8sia1	down	1.64	3.03E-02	chr6(-):142821541-142964452	UCSC	ST8 alpha-N-acetyl-neuraminide alpha-2,8-sialyltransferase 1	NM_011374	20449	ENSMUSG00000030283	9.07	8.35	No
GSMG0003704	---	down	1.64	1.47E-02	chr10(+):128645884-128649811	UCSC	---	AK047988	---	---	9.15	8.43	Yes
GSMG0030482	---	down	1.64	1.68E-03	chr5(-):49083874-49086770	UCSC	---	AK142091	---	---	7.62	6.9	No
GSMG0027445	Gm833	down	1.64	1.88E-03	chr4(+):152697540-152700922	UCSC	predicted gene 833	NR_033138	330004	ENSMUSG00000073699	9.38	8.66	Yes
GSMG0023684	---	down	1.64	5.02E-03	chr2(-):150852072-150853529	UCSC	---	AK159029	---	---	7.4	6.68	No
GSMG0023007	---	down	1.64	6.80E-03	chr2(-):76587266-76590617	UCSC	---	AK046927	---	---	6.25	5.54	Yes
GSMG0022495	---	down	1.64	2.48E-02	chr2(-):23033259-23037249	UCSC	---	AK144027	---	---	9.32	8.61	Yes
GSMG0022075	---	down	1.63	1.37E-02	chr2(+):157938463-157942411	UCSC	---	AK045808	---	---	7.71	7.01	Yes
GSMG0018542	---	down	1.63	9.04E-04	chr18(+):67951974-67954524	UCSC	---	AK029200	---	---	8.55	7.84	Yes
GSMG0017316	---	down	1.63	4.04E-03	chr17(-):28855136-28859505	UCSC	---	AK029928	---	---	7.18	6.48	Yes
GSMG0014433	---	down	1.63	1.36E-02	chr15(-):95747247-95751591	UCSC	---	BC038648	---	---	7.84	7.14	Yes
GSMG0013857	Hrsp12	down	1.63	1.31E-02	chr15(-):34484021-34495246	UCSC	heat-responsive protein 12	NM_008287	15473	ENSMUSG00000022323	8.35	7.65	No
GSMG0012319	Atg14	down	1.63	1.84E-02	chr14(-):47540894-47568435	UCSC	autophagy related 14	NM_172599	100504663	ENSMUSG00000037526	8.13	7.42	No
GSMG0011406	---	down	1.63	2.35E-02	chr14(+):32299336-32300292	UCSC	---	AK135594	---	---	6.93	6.22	Yes
GSMG0008993	D830013O20Rik	down	1.63	3.38E-02	chr12(-):73364075-73409563	UCSC	RIKEN cDNA D830013O20 gene	NR_046013	380765	ENSMUSG00000056359	7.18	6.48	Yes
GSMG0008808	Bzw2	down	1.63	4.20E-02	chr12(-):36091835-36156825	UCSC	basic leucine zipper and W2 domains 2	NM_025840	66912	ENSMUSG00000020547	10.29	9.59	No
GSMG0042811	Satl1	down	1.63	1.15E-02	chrX(-):112384305-112406779	UCSC	spermidine/spermine N1-acetyl transferase-like 1	NM_028655	73809	ENSMUSG00000025527	8.13	7.43	Yes
GSMG0040443	---	down	1.63	1.38E-02	chr9(-):21318079-21320619	UCSC	---	AK041135	---	---	7.29	6.59	No
GSMG0034736	---	down	1.63	2.20E-02	chr7(+):84338093-84347871	UCSC	---	AK035526	---	---	8.29	7.59	No
GSMG0034412	Klk1b1	down	1.63	3.96E-02	chr7(+):43966750-43971318	UCSC	kallikrein 1-related peptidase b1	AK089370	16623	ENSMUSG00000063133	6.9	6.2	Yes
GSMG0032789	Grm8	down	1.63	1.58E-02	chr6(-):27275121-28135137	UCSC	glutamate receptor, metabotropic 8	NM_008174	14823	ENSMUSG00000024211	6.69	5.99	Yes
GSMG0027780	Gm568	down	1.63	1.62E-02	chr4(-):47008319-47010781	UCSC	predicted gene 568	BC028561	---	ENSMUSG00000048824	7.89	7.19	Yes
GSMG0025503	B3galnt1	down	1.63	2.50E-02	chr3(-):69574159-69598862	UCSC	UDP-GalNAc:betaGlcNAc beta 1,3-galactosaminyltransferase, polypeptide 1	AK088407	26879	ENSMUSG00000043300	8.19	7.48	Yes
GSMG0023937	---	down	1.63	5.48E-03	chr2(-):169145075-169148287	UCSC	---	AK089748	---	---	8	7.29	Yes
GSMG0023123	Olfr1225	down	1.63	2.08E-02	chr2(-):89170219-89171245	UCSC	olfactory receptor 1225	NM_146891	258893	ENSMUSG00000075098	6.9	6.2	Yes
GSMG0020052	---	down	1.62	3.13E-02	chr19(-):7479778-7482340	UCSC	---	AK134314	---	---	7.43	6.73	Yes
GSMG0016422	---	down	1.62	1.06E-03	chr17(+):27201031-27203071	UCSC	---	AK139599	---	---	8.55	7.86	No
GSMG0014968	---	down	1.62	2.98E-02	chr16(+):38217181-38223996	UCSC	---	AK051712	---	---	7.82	7.13	No
GSMG0014565	Calcoco1	down	1.62	3.40E-03	chr15(-):102706779-102722178	UCSC	calcium binding and coiled coil domain 1	NM_026192	67488	ENSMUSG00000023055	9.34	8.64	No
GSMG0014341	7530416G11Rik	down	1.62	1.32E-02	chr15(-):85492736-85503228	UCSC	RIKEN cDNA 7530416G11 gene	AK078701	328577	ENSMUSG00000096607	8.6	7.91	No
GSMG0000174	Map4k4	down	1.62	1.01E-02	chr1(+):39900913-40026310	UCSC	mitogen-activated protein kinase kinase kinase kinase 4	NM_008696	26921	ENSMUSG00000026074	9.47	8.78	No
GSMG0011658	---	down	1.62	1.78E-02	chr14(+):60726450-60728136	UCSC	---	AK084129	---	---	8.7	8.01	No
GSMG0043189	Hmgcs1	down	1.62	7.72E-03	chr13(+):119690446-119708260	UCSC	3-hydroxy-3-methylglutaryl-Coenzyme A synthase 1	AK031297	208715	ENSMUSG00000093930	8.91	8.21	No
GSMG0007701	Gm11747	down	1.62	1.86E-02	chr11(-):118444200-118454997	UCSC	predicted gene 11747	AK033110	100126224	ENSMUSG00000086514	8.26	7.56	Yes
GSMG0041849	Avpr2	down	1.62	1.70E-02	chrX(+):73891841-73895502	UCSC	arginine vasopressin receptor 2	AK042836	12000	ENSMUSG00000031390	8.86	8.16	Yes
GSMG0040626	Sc5d	down	1.62	4.48E-02	chr9(-):42254139-42264300	UCSC	sterol-C5-desaturase (fungal ERG3, delta-5-desaturase) homolog (S. cerevis	NM_172769	235293	ENSMUSG00000032018	9.28	8.59	No
GSMG0038579	Ano8	down	1.62	3.86E-03	chr8(-):71476017-71486067	UCSC	anoctamin 8	NM_001164679	382014	ENSMUSG00000034863	8.06	7.36	No
GSMG0037771	---	down	1.62	4.32E-02	chr8(+):107175977-107178739	UCSC	---	BC023100	---	---	10.18	9.48	Yes
GSMG0036318	---	down	1.62	1.64E-02	chr7(-):99870666-99889359	UCSC	---	AK040058	---	---	7.1	6.41	Yes
GSMG0033329	Isy1	down	1.62	3.51E-02	chr6(-):87814280-87838759	UCSC	ISY1 splicing factor homolog (S. cerevisiae)	AK173116	57905	ENSMUSG00000030056	9.7	9.01	Yes
GSMG0028993	Slc35f6	down	1.62	4.61E-02	chr5(+):30647939-30659729	UCSC	solute carrier family 35, member F6	NM_175675	74919	ENSMUSG00000029175	9.05	8.35	Yes
GSMG0060114	Gm12429	down	1.62	6.40E-03	chr4(-):42848071-42853888	UCSC	predicted gene 12429	NM_001277167	433698	ENSMUSG00000078721	8.72	8.03	Yes
GSMG0018606	---	down	1.61	4.73E-02	chr18(+):75389919-75392208	UCSC	---	AK086531	---	---	8.41	7.72	No
GSMG0017319	Pxt1	down	1.61	8.48E-03	chr17(-):28933986-28942265	UCSC	peroxisomal, testis specific 1	AK005593	69307	ENSMUSG00000045378	9.13	8.45	Yes
GSMG0017087	---	down	1.61	2.39E-02	chr17(-):7938405-7942031	UCSC	---	AK078733	---	---	7.22	6.53	No
GSMG0016785	Gm6934	down	1.61	1.67E-02	chr17(+):51832817-51834723	UCSC	predicted gene 6934	AK153670	628919	---	9.13	8.45	No
GSMG0015752	Zdhhc23	down	1.61	2.18E-03	chr16(-):43965037-43979792	UCSC	zinc finger, DHHC domain containing 23	AK147553	332175	ENSMUSG00000036304	8.04	7.35	No
GSMG0001569	---	down	1.61	3.71E-02	chr1(-):52702270-52704762	UCSC	---	AK138105	---	---	7.11	6.42	No
GSMG0014098	Ago2	down	1.61	9.68E-03	chr15(-):73101625-73184947	UCSC	argonaute RISC catalytic subunit 2	NM_153178	239528	ENSMUSG00000036698	11.04	10.35	No
GSMG0013395	---	down	1.61	1.59E-02	chr15(+):80615948-80617981	UCSC	---	AK134048	---	---	8.24	7.56	No
GSMG0007480	Wnt9b	down	1.61	1.01E-02	chr11(-):103727363-103749822	UCSC	wingless-type MMTV integration site 9B	AK052708	22412	ENSMUSG00000018486	9.12	8.43	Yes
GSMG0006897	Acadvl	down	1.61	4.58E-04	chr11(-):70010183-70015428	UCSC	acyl-Coenzyme A dehydrogenase, very long chain	NM_017366	11370	ENSMUSG00000018574	9.12	8.43	No
GSMG0050881	Rab37	down	1.61	3.02E-02	chr11(+):115091429-115162240	UCSC	RAB37, member of RAS oncogene family	NM_001163753	58222	ENSMUSG00000020732	9.03	8.34	No
GSMG0005596	Gm11194	down	1.61	2.60E-02	chr11(+):78394483-78405657	UCSC	predicted gene 11194	AK041943	790911	ENSMUSG00000085747	8.7	8.01	Yes
GSMG0041989	Tsx	down	1.61	2.23E-02	chrX(+):103414063-103424585	UCSC	testis specific X-linked gene	NM_009440	22127	ENSMUSG00000031329	7.73	7.05	Yes
GSMG0041519	---	down	1.61	4.03E-02	chrM(+):15356-16294	UCSC	---	AK131592	---	---	11.32	10.63	Yes
GSMG0039281	---	down	1.61	5.98E-03	chr9(+):27991147-27992074	UCSC	---	AK021009	---	---	7.83	7.14	No
GSMG0037189	Mfhas1	down	1.61	1.40E-02	chr8(+):35587798-35679449	UCSC	malignant fibrous histiocytoma amplified sequence 1	NM_001081279	52065	ENSMUSG00000070056	9.45	8.77	No
GSMG0037033	Champ1	down	1.61	1.69E-02	chr8(+):13869641-13881639	UCSC	chromosome alignment maintaining phosphoprotein 1	NM_181854	101994	ENSMUSG00000047710	9.25	8.56	No
GSMG0036316	Neu3	down	1.61	2.62E-02	chr7(-):99811434-99828417	UCSC	neuraminidase 3	NM_016720	50877	ENSMUSG00000035239	7.67	6.98	Yes
GSMG0035935	Sergef	down	1.61	3.12E-02	chr7(-):46443159-46639807	UCSC	secretion regulating guanine nucleotide exchange factor	NM_013789	27414	ENSMUSG00000030839	8.77	8.09	No
GSMG0034750	---	down	1.61	3.62E-03	chr7(+):88063060-88066294	UCSC	---	AK033883	---	---	6.08	5.39	No
GSMG0003958	Nkain2	down	1.61	4.82E-03	chr10(-):31689319-32890495	UCSC	Na+/K+ transporting ATPase interacting 2	NM_001013411	432450	ENSMUSG00000069671	8.23	7.55	Yes
GSMG0032047	Cd8b1	down	1.61	3.91E-02	chr6(+):71322787-71337484	UCSC	CD8 antigen, beta chain 1	AK154055	12526	ENSMUSG00000053044	10.45	9.76	No
GSMG0028958	---	down	1.61	1.51E-02	chr5(+):28168476-28168840	UCSC	---	AF019615	---	---	7.7	7.02	No
GSMG0041523	---	down	1.61	2.91E-02	chr4(+):42470141-42472815	UCSC	---	AK138571	---	---	7.63	6.94	Yes
GSMG0051952	Gm12839	down	1.61	1.10E-02	chr4(-):115582038-115583127	UCSC	predicted gene 12839	NR_033575	631037	ENSMUSG00000083457	8.27	7.58	Yes
GSMG0025962	S1pr1	down	1.61	4.39E-02	chr3(-):115710433-115715055	UCSC	sphingosine-1-phosphate receptor 1	NM_007901	13609	ENSMUSG00000045092	12.34	11.66	No
GSMG0025197	Pde7a	down	1.61	3.42E-02	chr3(-):19224681-19311322	UCSC	phosphodiesterase 7A	NM_001122759	18583	ENSMUSG00000069094	9.53	8.84	No
GSMG0023809	Gm826	down	1.61	2.54E-02	chr2(-):160311399-160327496	UCSC	predicted gene 826	AK142710	329554	ENSMUSG00000074623	8.41	7.72	Yes
GSMG0022892	---	down	1.61	2.00E-02	chr2(-):64042243-64043493	UCSC	---	AK052002	---	---	6.14	5.46	Yes
GSMG0020568	---	down	1.6	1.01E-02	chr19(-):59882447-59885341	UCSC	---	AK155811	---	---	7.15	6.47	Yes
GSMG0019680	---	down	1.6	1.69E-02	chr19(+):41488044-41491010	UCSC	---	AK086255	---	---	8.4	7.71	Yes
GSMG0019391	---	down	1.6	4.25E-02	chr19(+):9135156-9279175	UCSC	---	AK048878	---	---	7.13	6.46	Yes
GSMG0017772	Txndc2	down	1.6	3.41E-02	chr17(-):65637505-65642204	UCSC	thioredoxin domain containing 2 (spermatozoa)	NM_001146002	213272	ENSMUSG00000050612	7.32	6.64	Yes
GSMG0016578	Abhd16a	down	1.6	1.61E-02	chr17(+):35089291-35102987	UCSC	abhydrolase domain containing 16A	NM_178592	193742	ENSMUSG00000007036	9.79	9.12	No
GSMG0013988	---	down	1.6	1.97E-02	chr15(-):55729723-55734027	UCSC	---	AK080316	---	---	7.06	6.38	Yes
GSMG0013450	AW121686	down	1.6	1.55E-02	chr15(+):82982868-82989134	UCSC	expressed sequence AW121686	AK140580	106026	ENSMUSG00000097301	7.54	6.86	Yes
GSMG0013038	Tiaf2	down	1.6	4.85E-02	chr15(+):27479006-27480902	UCSC	TGF-beta1-induced anti-apoptotic factor 2	AF075717	223431	---	7.24	6.57	Yes
GSMG0008300	---	down	1.6	4.68E-03	chr12(+):83678131-83681272	UCSC	---	AK143392	---	---	7.66	6.98	No
GSMG0006804	---	down	1.6	2.69E-02	chr11(-):63897395-63900351	UCSC	---	AK136300	---	---	6.92	6.24	Yes
GSMG0005727	A430104N18Rik	down	1.6	1.22E-03	chr11(+):87755577-87757267	UCSC	RIKEN cDNA A430104N18 gene	AK020764	---	ENSMUSG00000084796	9.58	8.9	No
GSMG0005621	---	down	1.6	2.88E-02	chr11(+):80211012-80213645	UCSC	---	AK149228	---	---	7.96	7.28	No
GSMG0041436	---	down	1.6	4.21E-02	chr9(-):119262623-119264170	UCSC	---	AK087903	---	---	7.69	7	No
GSMG0040795	Scaper	down	1.6	2.41E-02	chr9(-):55549883-55938122	UCSC	S phase cyclin A-associated protein in the ER	NM_001081341	244891	ENSMUSG00000034007	8.49	7.81	Yes
GSMG0039512	Cryab	down	1.6	4.32E-03	chr9(+):50751325-50756633	UCSC	crystallin, alpha B	BC094033	12955	ENSMUSG00000032060	8.22	7.54	Yes

FAST DB STABLE ID	Gene Symbol	Regulation	Fold-Change	P-Value	Gene Coordinates (mm10)	UCSC Link	Gene Name	Representative Transcript ID	Entrez Gene ID	EnsEMBL ID	log^2 intensity of untreated	log^2 intensity of treated	Low-specificity probe included
GSMG0039488	---	down	1.6	3.16E-02	chr9(+):47771141-47773375	UCSC	---	AK157591	---	---	6.9	6.22	No
GSMG0038618	---	down	1.6	3.92E-02	chr8(-):75992506-76099419	UCSC	---	EF591873	---	---	5.98	5.3	Yes
GSMG0038486	Cpe	down	1.6	4.27E-02	chr8(-):64592558-64693055	UCSC	carboxypeptidase E	AK160097	12876	ENSMUSG00000037852	6.99	6.31	Yes
GSMG0035753	Upk1a	down	1.6	4.36E-02	chr7(-):30603092-30612735	UCSC	uroplakin 1A	AK004014	109637	ENSMUSG00000006313	7.52	6.84	Yes
GSMG0043097	Prss3	down	1.6	2.35E-02	chr6(-):41373759-41377614	UCSC	protease, serine, 3	NM_011645	22073	ENSMUSG00000071519	9.22	8.54	Yes
GSMG0030787	---	down	1.6	2.71E-02	chr5(-):100410673-100414294	UCSC	---	AK089503	---	---	8.53	7.85	No
GSMG0030648	---	down	1.6	9.44E-03	chr5(-):76233119-76233780	UCSC	---	AK139981	---	---	8.1	7.42	No
GSMG0029807	---	down	1.6	7.50E-03	chr5(+):124003591-124005450	UCSC	---	AK081038	---	---	8.92	8.24	No
GSMG0057044	---	down	1.6	6.66E-03	chr4(+):62231252-62231281	UCSC	---	DQ720093	---	---	7.82	7.14	Yes
GSMG0025586	Fam160a1	down	1.6	1.54E-03	chr3(-):85660059-85746209	UCSC	family with sequence similarity 160, member A1	NM_172682	229488	ENSMUSG00000051000	7.23	6.56	Yes
GSMG0025569	---	down	1.6	1.43E-02	chr3(-):83952455-83954254	UCSC	---	AK138061	---	---	8.74	8.07	No
GSMG0021692	---	down	1.59	2.02E-02	chr2(+):122240540-122244446	UCSC	---	AK143491	---	---	8.21	7.55	No
GSMG0002562	Ephx1	down	1.59	2.42E-02	chr1(-):180989558-181017541	UCSC	epoxide hydrolase 1, microsomal	BC061493	13849	ENSMUSG00000038776	10.75	10.08	No
GSMG0019378	1810009A15Rik	down	1.59	9.14E-03	chr19(+):8888892-8890740	UCSC	RIKEN cDNA 1810009A15 gene	AK007390	66276	ENSMUSG00000071653	9.88	9.22	No
GSMG0019271	Ppp1ca	down	1.59	1.16E-03	chr19(+):4192155-4195424	UCSC	protein phosphatase 1, catalytic subunit, alpha isoform	AK090070	19045	ENSMUSG00000040385	12.32	11.65	No
GSMG0018935	---	down	1.59	4.77E-02	chr18(-):46235484-46237696	UCSC	---	AK131923	---	---	8.23	7.56	No
GSMG0017698	Safb2	down	1.59	9.16E-03	chr17(-):56562941-56584583	UCSC	scaffold attachment factor B2	NM_001029979	224902	ENSMUSG00000042625	10.67	10	No
GSMG0016723	Dlk2	down	1.59	2.06E-02	chr17(+):46297421-46303271	UCSC	delta-like 2 homolog (Drosophila)	NM_207666	106565	ENSMUSG00000047428	8.94	8.28	Yes
GSMG0014014	---	down	1.59	1.07E-02	chr15(-):59247366-59253581	UCSC	---	AK029886	---	---	8	7.33	No
GSMG0050926	Akap11	down	1.59	5.08E-03	chr14(-):78492246-78536860	UCSC	A kinase (PRKA) anchor protein 11	NM_001164503	219181	ENSMUSG00000022016	9.72	9.05	No
GSMG0012670	---	down	1.59	6.74E-03	chr14(-):75713470-75716036	UCSC	---	AK086265	---	---	8.42	7.75	Yes
GSMG0011483	Samd4	down	1.59	2.39E-02	chr14(+):46928449-46931300	UCSC	sterile alpha motif domain containing 4	AK031765	74480	ENSMUSG00000021838	7.2	6.54	Yes
GSMG0000137	Arid5a	down	1.59	4.32E-02	chr1(+):36307733-36324029	UCSC	AT rich interactive domain 5A (MRF1-like)	NR_033310	214855	ENSMUSG00000037447	10.19	9.52	No
GSMG0008715	Trib2	down	1.59	4.12E-02	chr12(-):15791727-15816852	UCSC	tribbles homolog 2 (Drosophila)	AK144192	217410	ENSMUSG00000020601	9.87	9.2	No
GSMG0008484	6230426M11Rik	down	1.59	1.00E-02	chr12(+):104752184-104752783	UCSC	RIKEN cDNA 6230426M11 gene	AK134511	100504301	---	7.5	6.83	No
GSMG0000706	Gpr39	down	1.59	5.80E-03	chr1(+):125676982-125873862	UCSC	G protein-coupled receptor 39	BC085285	71111	ENSMUSG00000026343	9.13	8.46	Yes
GSMG0000493	Cab39	down	1.59	4.96E-02	chr1(+):85793447-85851577	UCSC	calcium binding protein 39	NM_133781	12283	ENSMUSG00000036707	10.08	9.41	No
GSMG0005114	Olfr10	down	1.59	7.76E-03	chr11(+):49317548-49318490	UCSC	olfactory receptor 10	BC148303	18307	ENSMUSG00000057168	7.11	6.44	No
GSMG0042152	Prps1	down	1.59	1.17E-02	chrX(+):140456603-140476139	UCSC	phosphoribosyl pyrophosphate synthetase 1	NM_021463	19139	ENSMUSG00000031432	11	10.33	No
GSMG0004834	Tbc1d10a	down	1.59	2.89E-02	chr11(+):4186820-4217400	UCSC	TBC1 domain family, member 10a	AK220243	103724	ENSMUSG00000034412	9.5	8.83	Yes
GSMG0038855	Cenpt	down	1.59	8.94E-03	chr8(-):105844675-105852055	UCSC	centromere protein T	AK089172	320394	ENSMUSG00000036672	7.88	7.21	No
GSMG0004382	Zfp938	down	1.59	1.99E-02	chr10(-):82224854-82241281	UCSC	zinc finger protein 938	AK045871	237411	ENSMUSG00000062931	9.42	8.75	No
GSMG0033104	Vmn1r32	down	1.59	3.89E-02	chr6(-):66552183-66559710	UCSC	vomeronasal 1 receptor 32	AK029205	171188	ENSMUSG00000062905	6.11	5.44	Yes
GSMG0032562	---	down	1.59	3.82E-05	chr6(+):134238976-134243265	UCSC	---	AK157524	---	---	8.46	7.79	No
GSMG0031384	Slc7a1	down	1.59	3.95E-02	chr5(-):148327412-148399904	UCSC	solute carrier family 7 (cationic amino acid transporter, y+ system), member	NM_007513	11987	ENSMUSG00000041313	9.92	9.25	No
GSMG0030181	---	down	1.59	2.64E-02	chr5(-):11485335-11501453	UCSC	---	AK015330	---	---	7.93	7.26	Yes
GSMG0003427	---	down	1.59	4.66E-02	chr10(+):94397471-94398810	UCSC	---	AK035442	---	---	7.66	6.99	No
GSMG0027472	Mxra8	down	1.59	2.39E-02	chr4(+):155839680-155844101	UCSC	matrix-remodelling associated 8	NM_024263	74761	ENSMUSG00000029070	8	7.33	Yes
GSMG0026408	---	down	1.59	2.41E-02	chr4(+):34577170-34580704	UCSC	---	AK140993	---	---	9.19	8.52	No
GSMG0002873	---	down	1.59	4.72E-03	chr10(+):36769385-36771093	UCSC	---	AK048797	---	---	6.85	6.19	Yes
GSMG0022226	Pfdn4	down	1.58	7.46E-03	chr2(+):170496428-170519070	UCSC	prefoldin 4	NM_001013369	109054	ENSMUSG00000052033	9.46	8.8	No
GSMG0021225	---	down	1.58	5.68E-03	chr2(+):72982797-72983952	UCSC	---	AK084892	---	---	6.87	6.21	Yes
GSMG0020260	A930007I19Rik	down	1.58	9.03E-04	chr19(-):29464750-29523035	UCSC	RIKEN cDNA A930007I19 gene	AK132712	77779	ENSMUSG00000097855	8.35	7.69	No
GSMG0019616	Fas	down	1.58	1.94E-02	chr19(+):34290659-34327770	UCSC	Fas (TNF receptor superfamily member 6)	NM_007987	14102	ENSMUSG00000024778	8.29	7.64	Yes
GSMG0019254	Suv420h1	down	1.58	4.88E-03	chr19(+):3767420-3818303	UCSC	suppressor of variegation 4-20 homolog 1 (Drosophila)	NM_144871	225888	ENSMUSG00000045098	10.67	10.01	No
GSMG0018260	Ik	down	1.58	8.16E-04	chr18(+):36744656-36757639	UCSC	IK cytokine	NM_011879	24010	ENSMUSG00000024474	9.5	8.84	No
GSMG0012673	---	down	1.58	3.77E-02	chr14(-):75945363-75948165	UCSC	---	AK084057	---	---	7.76	7.1	No
GSMG0011983	---	down	1.58	4.20E-02	chr14(+):121995511-121998042	UCSC	---	AK037884	---	---	8.91	8.25	Yes
GSMG0010847	Srd5a1	down	1.58	4.76E-02	chr13(-):69573450-69611463	UCSC	steroid 5 alpha-reductase 1	NM_175283	78925	ENSMUSG00000021594	6.94	6.28	Yes
GSMG0010231	---	down	1.58	2.42E-03	chr13(+):113943143-113944716	UCSC	---	AK033262	---	---	7.61	6.95	No
GSMG0008693	---	down	1.58	5.10E-03	chr12(-):10219860-10222330	UCSC	---	AK157193	---	---	7.19	6.53	Yes
GSMG0007869	B430203G13Rik	down	1.58	4.49E-02	chr12(+):17924294-17925668	UCSC	RIKEN cDNA B430203G13 gene	AK046603	---	ENSMUSG00000067356	7.36	6.7	Yes
GSMG0007104	Nle1	down	1.58	2.52E-02	chr11(-):82900768-82908395	UCSC	notchless homolog 1 (Drosophila)	NM_145431	217011	ENSMUSG00000020692	9.13	8.47	No
GSMG0042419	Nkrf	down	1.58	3.92E-02	chrX(-):36887544-36902903	UCSC	NF-kappaB repressing factor	NM_029891	77286	ENSMUSG00000044149	8.92	8.26	No
GSMG0041136	1190002N15Rik	down	1.58	1.79E-02	chr9(-):94517864-94538081	UCSC	RIKEN cDNA 1190002N15 gene	NM_001033145	68861	ENSMUSG00000045414	8.22	7.56	Yes
GSMG0040837	Islr2	down	1.58	1.69E-02	chr9(-):58196298-58204319	UCSC	immunoglobulin superfamily containing leucine-rich repeat 2	NM_001161541	320563	ENSMUSG00000051243	9.61	8.95	Yes
GSMG0040516	---	down	1.58	7.70E-03	chr9(-):29539504-29543650	UCSC	---	AK032679	---	---	6.84	6.18	No
GSMG0039860	Irak1bp1	down	1.58	3.68E-03	chr9(+):82829803-82847688	UCSC	interleukin-1 receptor-associated kinase 1 binding protein 1	NM_022986	65099	ENSMUSG00000032251	7.61	6.95	No
GSMG0037977	---	down	1.58	1.74E-02	chr8(+):123835446-123836721	UCSC	---	AK144863	---	---	8.02	7.36	No
GSMG0043615	Syce1l	down	1.58	1.32E-02	chr8(+):113643213-113655533	UCSC	synaptonemal complex central element protein 1 like	NM_029139	668110	ENSMUSG00000033409	9.49	8.83	Yes
GSMG0037810	Ftsjd1	down	1.58	7.17E-04	chr8(+):110217960-110224489	UCSC	FtsJ methyltransferase domain containing 1	NM_146215	234728	ENSMUSG00000046441	9.56	8.9	Yes
GSMG0036346	Folr1	down	1.58	9.42E-03	chr7(-):101858332-101870788	UCSC	folate receptor 1 (adult)	NM_008034	14275	ENSMUSG00000001827	8.31	7.65	Yes
GSMG0036228	Abhd17c	down	1.58	3.22E-02	chr7(-):84109358-84151893	UCSC	abhydrolase domain containing 17C	NM_133722	70178	ENSMUSG00000038459	9.18	8.52	No
GSMG0004083	Gopc	down	1.58	3.28E-02	chr10(-):52337024-52382124	UCSC	golgi associated PDZ and coiled-coil motif containing	NM_053187	94221	ENSMUSG00000019861	8.49	7.83	No
GSMG0034244	Rps16	down	1.58	2.96E-02	chr7(+):28350689-28352698	UCSC	ribosomal protein S16	NM_013647	20055	ENSMUSG00000037563	12.59	11.94	Yes
GSMG0033524	Gt(ROSA)26Sor	down	1.58	1.79E-02	chr6(-):113067431-113077244	UCSC	gene trap ROSA 26, Philippe Soriano	NR_027010	14910	ENSMUSG00000086429	7.55	6.88	Yes
GSMG0003360	---	down	1.58	2.39E-02	chr10(+):87230759-87239243	UCSC	---	AK016129	---	---	5.97	5.31	Yes
GSMG0027846	Gm12526	down	1.58	1.49E-02	chr4(-):57756340-57763903	UCSC	predicted gene 12526	AK153685	100038668	ENSMUSG00000073846	9.68	9.02	Yes
GSMG0027262	Usp48	down	1.58	1.88E-02	chr4(+):137593751-137658536	UCSC	ubiquitin specific peptidase 48	NM_130879	170707	ENSMUSG00000043411	11.13	10.46	No
GSMG0027135	---	down	1.58	3.30E-02	chr4(+):129829079-129833771	UCSC	---	AK153822	---	---	9.5	8.84	Yes
GSMG0025554	---	down	1.58	2.51E-02	chr3(-):81921268-81924464	UCSC	---	AK045605	---	---	9.4	8.74	Yes
GSMG0016582	Bag6	down	1.57	1.47E-02	chr17(+):35135178-35147322	UCSC	BCL2-associated athanogene 6	NM_057171	224727	ENSMUSG00000024392	10.12	9.46	Yes
GSMG0015535	Olfr170	down	1.57	1.55E-02	chr16(-):19605696-19606703	UCSC	olfactory receptor 170	BC132587	258959	ENSMUSG00000062245	6.83	6.19	No
GSMG0015466	Ifitm7	down	1.57	2.60E-03	chr16(-):13981702-13986867	UCSC	interferon induced transmembrane protein 7	NM_001270718	74482	ENSMUSG00000065968	7.43	6.78	No
GSMG0001605	4930558J18Rik	down	1.57	2.10E-02	chr1(-):57359222-57377544	UCSC	RIKEN cDNA 4930558J18 gene	NR_037999	75323	ENSMUSG00000097519	8.26	7.61	Yes
GSMG0011956	---	down	1.57	4.26E-02	chr14(+):119098964-119115050	UCSC	---	AK147121	---	---	7.1	6.45	Yes
GSMG0010884	---	down	1.57	2.68E-02	chr13(-):74460135-74462685	UCSC	---	AK080840	---	---	7.65	7	Yes
GSMG0010210	Il6st	down	1.57	1.59E-02	chr13(+):112464070-112506860	UCSC	interleukin 6 signal transducer	NM_010560	16195	ENSMUSG00000021756	10.51	9.85	No
GSMG0008630	---	down	1.57	2.20E-02	chr12(+):117602039-117603762	UCSC	---	AK020886	---	---	7.33	6.69	No
GSMG0007540	---	down	1.57	1.03E-02	chr11(-):107268591-107270347	UCSC	---	AK086322	---	---	8.19	7.54	Yes
GSMG0007531	---	down	1.57	3.30E-02	chr11(-):107090946-107093582	UCSC	---	BC037679	---	---	8.45	7.81	Yes
GSMG0042291	---	down	1.57	4.80E-02	chrX(+):169076787-169079054	UCSC	---	AK156406	---	---	6.36	5.72	Yes
GSMG0040043	Rrp9	down	1.57	1.84E-03	chr9(+):106477306-106485416	UCSC	RRP9, small subunit (SSU) processome component, homolog (yeast)	AK076113	27966	ENSMUSG00000041506	9.16	8.5	Yes
GSMG0039303	Kcnj1	down	1.57	3.77E-02	chr9(+):32372418-32399197	UCSC	potassium inwardly-rectifying channel, subfamily J, member 1	NM_019659	56379	ENSMUSG00000041248	7.25	6.6	No
GSMG0038819	Tk2	down	1.57	4.25E-02	chr8(-):104226691-104248560	UCSC	thymidine kinase 2, mitochondrial	NR_045642	57813	ENSMUSG00000035824	10.29	9.63	No
GSMG0038170	Gm14851	down	1.57	5.84E-03	chr8(-):21094971-21096050	UCSC	predicted gene 14851	NM_001177482	634825	ENSMUSG00000061958	8.22	7.56	Yes
GSMG0004277	---	down	1.57	4.19E-02	chr10(-):76856380-76863349	UCSC	---	AK148675	---	---	7.15	6.5	No
GSMG0035866	Mybpc2	down	1.57	6.24E-03	chr7(-):44501699-44524669	UCSC	myosin binding protein C, fast-type	NM_146189	233199	ENSMUSG00000038670	7.7	7.05	Yes
GSMG0003933	Rnf146	down	1.57	2.51E-02	chr10(-):29344178-29362442	UCSC	ring finger protein 146	NM_026518	68031	ENSMUSG00000038876	10.98	10.33	No
GSMG0033257	1700124L16Rik	down	1.57	3.89E-02	chr6(-):83761759-83762510	UCSC	RIKEN cDNA 1700124L16 gene	AK005768	67934	---	6.48	5.83	Yes
GSMG0032833	---	down	1.57	1.88E-02	chr6(-):31795669-31798498	UCSC	---	AK041456	---	---	6.49	5.84	Yes
GSMG0043537	Zfp783	down	1.57	1.97E-02	chr6(+):47943175-47954549	UCSC	zinc finger protein 783	NR_027963	232785	ENSMUSG00000072653	7.84	7.19	Yes
GSMG0026433	---	down	1.57	3.66E-03	chr4(+):41177452-41179633	UCSC	---	AK009532	---	---	8.3	7.65	No
GSMG0025775	Nudt17	down	1.57	5.66E-03	chr3(-):96705892-96708565	UCSC	nudix (nucleoside diphosphate linked moiety X)-type motif 17	NM_030094	78373	ENSMUSG00000028100	8.8	8.14	Yes
GSMG0024040	Arfrp1	down	1.57	9.96E-03	chr2(-):181357690-181365404	UCSC	ADP-ribosylation factor related protein 1	NR_028585	76688	ENSMUSG00000038671	9.49	8.84	No
GSMG0023609	Esf1	down	1.57	7.24E-04	chr2(-):140119881-140170566	UCSC	ESF1, nucleolar pre-rRNA processing protein, homolog (S. cerevisiae)	NM_001081090	66580	ENSMUSG00000045624	8.79	8.13	No
GSMG0001981	---	down	1.56	4.22E-02	chr1(-):118294778-118297580	UCSC	---	AK047464	---	---	8.17	7.53	No
GSMG0015227	---	down	1.56	2.31E-02	chr16(+):78318306-78319166	UCSC	---	AK051593	---	---	6.97	6.33	Yes
GSMG0012997	---	down	1.56	3.82E-02	chr15(+):16790593-16793385	UCSC	---	AK082213	---	---	8.06	7.42	Yes
GSMG0012849	Dct	down	1.56	4.63E-02	chr14(-):118012792-118052246	UCSC	dopachrome tautomerase	NM_010024	13190	ENSMUSG00000022129	7.61	6.97	No
GSMG0051411	Fhit	down	1.56	3.21E-02	chr14(-):9550095-9870182	UCSC	fragile histidine triad gene	NM_010210	14198	ENSMUSG00000060579	8.31	7.67	No
GSMG0010326	Ggps1	down	1.56	3.44E-02	chr13(-):13982170-14063415	UCSC	geranylgeranyl diphosphate synthase 1	BC006798	14593	ENSMUSG00000021302	8.89	8.25	No
GSMG0010117	Mrps27	down	1.56	6.57E-04	chr13(+):99344786-99415569	UCSC	mitochondrial ribosomal protein S27	NM_173757	218506	ENSMUSG00000041632	7.76	7.12	No
GSMG0008566	---	down	1.56	1.78E-02	chr12(+):111079469-111081424	UCSC	---	AY512946	---	---	7.41	6.77	No
GSMG0006413	---	down	1.56	4.96E-02	chr11(-):22729897-22732987	UCSC	---	AK078949	---	---	7.33	6.68	Yes
GSMG0042779	5330434G04Rik	down	1.56	1.62E-02	chrX(-):105348282-105391778	UCSC	RIKEN cDNA 5330434G04 gene	AK134610	100043213	ENSMUSG00000087620	8.66	8.01	Yes
GSMG0041397	---	down	1.56	2.14E-02	chr9(-):116105682-116107368	UCSC	---	AK039962	---	---	8.47	7.83	No
GSMG0040202	---	down	1.56	1.14E-02	chr9(+):117873143-117881202	UCSC	---	AK139159	---	---	8.58	7.94	Yes
GSMG0037314	Sh3rf1	down	1.56	2.57E-02	chr8(+):61223872-61396072	UCSC	SH3 domain containing ring finger 1	NM_021506	59009	ENSMUSG00000031642	7.67	7.03	Yes
GSMG0004212	---	down	1.56	3.68E-02	chr10(-):68272761-68275224	UCSC	---	AK034735	---	---	9.46	8.81	No
GSMG0035578	Fosb	down	1.56	3.83E-02	chr7(-):19302696-19310045	UCSC	FBJ osteosarcoma oncogene B	NM_008036	14282	ENSMUSG00000003545	11.53	10.89	No
GSMG0034912	---	down	1.56	3.30E-02	chr7(+):102213817-102216375	UCSC	---	AK054383	---	---	6.5	5.86	Yes
GSMG0033453	---	down	1.56	3.70E-02	chr6(-):99044122-99046516	UCSC	---	AK037790	---	---	9.62	8.98	No
GSMG0032041	Eif2ak3	down	1.56	9.58E-03	chr6(+):70847839-70852074	UCSC	eukaryotic translation initiation factor 2 alpha kinase 3	AK037707	13666	ENSMUSG00000031668	10.47	9.83	No
GSMG0030933	2900026A02Rik	down	1.56	7.06E-03	chr5(-):113086323-113163352	UCSC	RIKEN cDNA 2900026A02 gene	NM_172884	243219	ENSMUSG00000051339	9.19	8.55	Yes
GSMG0030899	Gm15787	down	1.56	2.62E-03	chr5(-):110167508-110176505	UCSC	predicted gene 15787	AK053084	100504007	ENSMUSG00000086247	7.55	6.91	No
GSMG0030886	Dgkq	down	1.56	1.86E-02	chr5(-):108646690-108669672	UCSC	diacylglycerol kinase, theta	AK043933	110524	ENSMUSG00000004815	8.3	7.65	No
GSMG0029661	Gm19344	down	1.56	3.92E-02	chr5(+):114780594-114784570	UCSC	predicted gene, 19344	AK134353	100502733	---	7.73	7.08	No
GSMG0029649	---	down	1.56	1.20E-03	chr5(+):114115825-114117681	UCSC	---	AK143232	---	---	8.32	7.68	Yes
GSMG0027691	Aqp3	down	1.56	9.48E-03	chr4(-):41092724-41098185	UCSC	aquaporin 3	AK145678	11828	ENSMUSG00000028435	8.11	7.47	Yes
GSMG0026717	Acer2	down	1.56	3.58E-03	chr4(+):86874414-86934821	UCSC	alkaline ceramidase 2	AK085306	230379	ENSMUSG00000038007	8.17	7.53	Yes
GSMG0026220	Tyw3	down	1.56	3.74E-02	chr3(-):154576520-154597098	UCSC	tRNA-yW synthesizing protein 3 homolog (S. cerevisiae)	NM_172474	209584	ENSMUSG00000047583	7.43	6.79	Yes
GSMG0051661	Obp2b	down	1.55	2.03E-02	chr2(+):25737009-25740097	UCSC	odorant binding protein 2B	NM_001099301	383678	ENSMUSG00000079539	8.54	7.91	Yes
GSMG0020721	Bmi1	down	1.55	6.16E-03	chr2(+):18677018-18686629	UCSC	Bmi1 polycomb ring finger oncogene	NM_007552	12151	ENSMUSG00000026739	10.14	9.51	No
GSMG0020212	---	down	1.55	4.32E-02	chr19(-):24521743-24524478	UCSC	---	AK141407	---	---	7.15	6.52	Yes
GSMG0019446	Pfpl	down	1.55	1.33E-02	chr19(+):12427905-12432110	UCSC	pore forming protein-like	NM_019540	56093	ENSMUSG00000040065	7.57	6.93	Yes
GSMG0019339	Pygm	down	1.55	2.41E-02	chr19(+):6384429-6398458	UCSC	muscle glycogen phosphorylase	NM_011224	19309	ENSMUSG00000032648	8.23	7.6	No
GSMG0002259	Lhx4	down	1.55	1.17E-02	chr1(-):155701692-155742027	UCSC	LIM homeobox protein 4	NM_010712	16872	ENSMUSG00000026468	8.24	7.61	Yes
GSMG0017332	Tmem217	down	1.55	2.96E-03	chr17(-):29526026-29549595	UCSC	transmembrane protein 217	AK016810	71138	ENSMUSG00000079580	8.19	7.56	No
GSMG0015514	Prodh	down	1.55	3.12E-03	chr16(-):18060375-18089190	UCSC	proline dehydrogenase	AK085938	19125	ENSMUSG00000003526	7.85	7.22	Yes
GSMG0014404	---	down	1.55	1.28E-02	chr15(-):93249159-93251314	UCSC	---	AK038245	---	---	8.89	8.26	No
GSMG0014096	---	down	1.55	2.01E-02	chr15(-):73097001-73099706	UCSC	---	AK148725	---	---	11.29	10.65	No
GSMG0013974	---	down	1.55	2.48E-02	chr15(-):53798003-53799926	UCSC	---	AK039383	---	---	5.24	4.6	Yes
GSMG0013957	Eif3h	down	1.55	1.30E-03	chr15(-):51786558-51865503	UCSC	eukaryotic translation initiation factor 3, subunit H	AK168123	68135	ENSMUSG00000022312	11.56	10.92	Yes
GSMG0009014	---	down	1.55	1.41E-02	chr12(-):76344525-76345381	UCSC	---	AK020759	---	---	8.2	7.57	No
GSMG0006404	---	down	1.55	2.43E-02	chr11(-):22111981-22115824	UCSC	---	AK052379	---	---	7.61	6.98	No
GSMG0006191	---	down	1.55	2.44E-03	chr11(+):119616516-119620180	UCSC	---	AK081250	---	---	7.34	6.71	Yes
GSMG0052611	Cypt14	down	1.55	4.60E-03	chrX(-):39862919-39863604	UCSC	cysteine-rich perinuclear theca 14	NM_001191032	100504400	ENSMUSG00000095240	7.13	6.5	No
GSMG0042098	Armcx4	down	1.55	2.10E-02	chrX(+):134686519-134697775	UCSC	armadillo repeat containing, X-linked 4	NM_001202500	100503043	ENSMUSG00000049804	7.62	7	No
GSMG0041306	Arih2	down	1.55	3.12E-02	chr9(-):108602942-108653681	UCSC	ariadne homolog 2 (Drosophila)	AK167104	23807	ENSMUSG00000064145	10.38	9.75	No
GSMG0037985	Cog2	down	1.55	2.36E-02	chr8(+):124520767-124552005	UCSC	component of oligomeric golgi complex 2	NM_029746	76332	ENSMUSG00000031979	8.42	7.79	No
GSMG0034649	---	down	1.55	1.41E-02	chr7(+):75707885-75709182	UCSC	---	AK053961	---	---	9.48	8.85	Yes
GSMG0034033	---	down	1.55	1.24E-03	chr7(+):13038031-13039851	UCSC	---	AK149506	---	---	7.86	7.22	No
GSMG0000333	---	down	1.55	1.57E-02	chr1(+):60989479-60991963	UCSC	---	AK137839	---	---	7.38	6.74	No
GSMG0033608	---	down	1.55	1.57E-02	chr6(-):119643701-119649463	UCSC	---	AK165064	---	---	7.72	7.09	Yes
GSMG0003826	---	down	1.55	1.80E-02	chr10(-):13687206-13689807	UCSC	---	AK032614	---	---	7.56	6.92	Yes
GSMG0032857	---	down	1.55	1.94E-02	chr6(-):35029274-35032444	UCSC	---	AK047612	---	---	7.26	6.62	Yes
GSMG0031697	---	down	1.55	1.99E-02	chr6(+):37941016-37942406	UCSC	---	AK018532	---	---	7.7	7.06	No
GSMG0030492	Gpr125	down	1.55	4.32E-02	chr5(-):49959951-50058996	UCSC	G protein-coupled receptor 125	NM_133911	70693	ENSMUSG00000029090	7.16	6.53	Yes
GSMG0029514	D930016D06Rik	down	1.55	2.27E-02	chr5(+):104525735-104554211	UCSC	RIKEN cDNA D930016D06 gene	NR_030673	100662	ENSMUSG00000097392	8.15	7.52	Yes
GSMG0026119	Rap1gds1	down	1.55	4.52E-03	chr3(-):138925906-139075203	UCSC	RAP1, GTP-GDP dissociation stimulator 1	NM_145544	229877	ENSMUSG00000028149	9.63	8.99	No
GSMG0025782	---	down	1.55	1.37E-02	chr3(-):96998722-97000772	UCSC	---	AK020347	---	---	7.28	6.65	No
GSMG0000254	---	down	1.55	4.69E-02	chr1(+):55068347-55071059	UCSC	---	AK076312	---	---	7.83	7.2	Yes
GSMG0024425	---	down	1.55	1.23E-02	chr3(+):69753114-69756373	UCSC	---	AK035458	---	---	7.49	6.85	Yes
GSMG0021883	Otor	down	1.54	4.00E-03	chr2(+):143078393-143081713	UCSC	otoraplin	AJ243939	57329	ENSMUSG00000027416	7.52	6.89	No
GSMG0018263	Zmat2	down	1.54	1.51E-02	chr18(+):36793923-36799660	UCSC	zinc finger, matrin type 2	NM_025594	66492	ENSMUSG00000001383	9.13	8.5	Yes
GSMG0002052	---	down	1.54	3.82E-02	chr1(-):127978905-127981691	UCSC	---	AK037130	---	---	6.86	6.24	No
GSMG0017529	Olfr114	down	1.54	4.88E-03	chr17(-):37589382-37590381	UCSC	olfactory receptor 114	BC132573	258284	ENSMUSG00000062629	7.4	6.78	No
GSMG0017330	---	down	1.54	4.77E-02	chr17(-):29432310-29435458	UCSC	---	AK153778	---	---	7.08	6.46	No
GSMG0015717	Slc15a2	down	1.54	4.22E-02	chr16(-):36750164-36784962	UCSC	solute carrier family 15 (H+/peptide transporter), member 2	NM_021301	57738	ENSMUSG00000022899	7.09	6.47	No
GSMG0015282	Sod1	down	1.54	9.40E-03	chr16(+):90220742-90226331	UCSC	superoxide dismutase 1, soluble	NM_011434	20655	ENSMUSG00000022982	11.26	10.64	No
GSMG0014115	Arc	down	1.54	2.15E-02	chr15(-):74669081-74672572	UCSC	activity regulated cytoskeletal-associated protein	NM_018790	11838	ENSMUSG00000022602	8.12	7.5	No
GSMG0042316	Usp27x	down	1.54	5.41E-04	chrX(-):7371276-7375827	UCSC	ubiquitin specific peptidase 27, X chromosome	AK028249	54651	ENSMUSG00000046269	8	7.37	Yes
GSMG0004868	Ccm2	down	1.54	5.58E-03	chr11(+):6546887-6596761	UCSC	cerebral cavernous malformation 2	NM_146014	216527	ENSMUSG00000000378	10.43	9.8	No
GSMG0041263	---	down	1.54	1.36E-02	chr9(-):106668619-106672275	UCSC	---	AK052727	---	---	7.79	7.16	Yes
GSMG0038551	Klhl26	down	1.54	2.24E-02	chr8(-):70450219-70476957	UCSC	kelch-like 26	AK036321	234378	ENSMUSG00000055707	8.6	7.97	No
GSMG0037486	Zfp827	down	1.54	2.30E-03	chr8(+):79028437-79193765	UCSC	zinc finger protein 827	NM_178267	622675	ENSMUSG00000071064	8.44	7.82	No
GSMG0049843	---	down	1.54	9.30E-03	chr8(+):78710989-78714857	UCSC	---	AK037335	---	---	6.92	6.3	Yes
GSMG0004268	Pcnt	down	1.54	4.20E-02	chr10(-):76351254-76442912	UCSC	pericentrin (kendrin)	NM_008787	18541	ENSMUSG00000001151	8.81	8.19	No
GSMG0031215	Sh2b2	down	1.54	3.72E-02	chr5(-):136218147-136244903	UCSC	SH2B adaptor protein 2	NM_018825	23921	ENSMUSG00000005057	9.15	8.52	Yes
GSMG0031092	Eif2b1	down	1.54	4.38E-03	chr5(-):124570214-124579058	UCSC	eukaryotic translation initiation factor 2B, subunit 1 (alpha)	AK133695	209354	ENSMUSG00000029388	9.43	8.81	Yes
GSMG0028671	---	down	1.54	1.61E-02	chr4(-):149392418-149395964	UCSC	---	AK082244	---	---	7.98	7.35	No
GSMG0028642	4933438K21Rik	down	1.54	3.14E-02	chr4(-):147063366-147068437	UCSC	RIKEN cDNA 4933438K21 gene	AK017111	71270	ENSMUSG00000093617	9.79	9.16	Yes
GSMG0028598	Cela2a	down	1.54	1.99E-02	chr4(-):141814963-141826004	UCSC	chymotrypsin-like elastase family, member 2A	X04573	13706	ENSMUSG00000058579	8.1	7.48	No
GSMG0027534	---	down	1.54	4.52E-03	chr4(-):6767189-6769396	UCSC	---	AK037853	---	---	7.96	7.33	No
GSMG0027401	---	down	1.54	9.18E-03	chr4(+):149589321-149592413	UCSC	---	AK036293	---	---	6.74	6.12	No
GSMG0026429	---	down	1.54	7.36E-03	chr4(+):41005691-41008130	UCSC	---	AK140823	---	---	8.1	7.48	No
GSMG0026234	Srsf11	down	1.54	3.36E-03	chr3(-):158010493-158036639	UCSC	serine/arginine-rich splicing factor 11	NM_001093752	69207	ENSMUSG00000055436	11.22	10.59	Yes
GSMG0025939	Prpf38b	down	1.54	1.56E-02	chr3(-):108902807-108911728	UCSC	PRP38 pre-mRNA processing factor 38 (yeast) domain containing B	AK159267	66921	ENSMUSG00000027881	10.73	10.11	No
GSMG0023702	Trib3	down	1.54	1.61E-02	chr2(-):152337425-152344060	UCSC	tribbles homolog 3 (Drosophila)	NM_175093	228775	ENSMUSG00000032715	8.46	7.83	Yes
GSMG0019933	---	down	1.53	4.90E-02	chr19(-):4298747-4300524	UCSC	---	AK090222	---	---	8.06	7.45	No
GSMG0019559	Vldlr	down	1.53	1.33E-02	chr19(+):27216483-27254231	UCSC	very low density lipoprotein receptor	NM_013703	22359	ENSMUSG00000024924	7.4	6.78	Yes
GSMG0019476	C130060C02Rik	down	1.53	1.53E-02	chr19(+):15985074-16010912	UCSC	RIKEN cDNA C130060C02 gene	NR_045355	100502653	ENSMUSG00000097557	7.64	7.02	Yes
GSMG0018234	---	down	1.53	3.58E-02	chr18(+):35599227-35600008	UCSC	---	AK043980	---	---	10.35	9.73	No
GSMG0012479	Phf11a	down	1.53	2.71E-02	chr14(-):59276914-59297522	UCSC	PHD finger protein 11A	AK077176	219131	ENSMUSG00000044703	7.02	6.4	No
GSMG0011277	Zswim8	down	1.53	5.50E-03	chr14(+):20707551-20723618	UCSC	zinc finger SWIM-type containing 8	AK147398	268721	ENSMUSG00000021819	9.23	8.62	Yes

FAST DB STABLE ID	Gene Symbol	Regulation	Fold-Change	P-Value	Gene Coordinates (mm10)	UCSC Link	Gene Name	Representative Transcript ID	Entrez Gene ID	EnsEMBL ID	log^{2} intensity of untreated	log^{2} intensity of treated	Low-specificity probe included
GSMG0043708	Slc38a9	down	1.53	9.44E-04	chr13(+):112660766-112738752	UCSC	solute carrier family 38, member 9	NM_178746	268706	ENSMUSG00000047789	8.13	7.51	No
GSMG0009786	Simc1	down	1.53	8.34E-03	chr13(+):54503805-54551298	UCSC	SUMO-interacting motifs containing 1	NM_176987	319719	ENSMUSG00000043183	9.17	8.55	No
GSMG0000920	---	down	1.53	4.24E-02	chr1(+):156455288-156457852	UCSC	---	AK155161	---	---	6.71	6.1	Yes
GSMG0043684	Slc25a19	down	1.53	4.72E-03	chr11(-):115614182-115628295	UCSC	solute carrier family 25 (mitochondrial thiamine pyrophosphate carrier), meml	NM_026071	67283	ENSMUSG00000020744	8.75	8.14	No
GSMG0006359	2810442I21Rik	down	1.53	2.02E-02	chr11(-):16935157-16951283	UCSC	RIKEN cDNA 2810442I21 gene	AK013290	72735	ENSMUSG00000087060	7.37	6.75	No
GSMG0041398	---	down	1.53	1.87E-02	chr9(-):116143768-116147444	UCSC	---	AK158205	---	---	9.79	9.17	No
GSMG0052462	Olfr914	down	1.53	3.29E-02	chr9(+):38606467-38607417	UCSC	olfactory receptor 914	NM_146786	258782	ENSMUSG00000047050	7.24	6.63	Yes
GSMG0003944	---	down	1.53	3.34E-02	chr10(-):30612108-30614552	UCSC	---	AK047572	---	---	7.86	7.25	No
GSMG0003941	Trmt11	down	1.53	1.22E-02	chr10(-):30534225-30600749	UCSC	tRNA methyltransferase 11	NM_028604	73681	ENSMUSG00000019792	7.7	7.08	Yes
GSMG0033806	Gpr19	down	1.53	1.38E-02	chr6(-):134869092-134897925	UCSC	G protein-coupled receptor 19	NR_072990	14760	ENSMUSG00000032641	7.87	7.25	Yes
GSMG0052098	---	down	1.53	2.12E-02	chr6(-):123385262-123418061	UCSC	---	NM_001104634	---	---	5.7	5.08	Yes
GSMG0030722	Gm19619	down	1.53	1.75E-02	chr5(-):91257571-91283077	UCSC	predicted gene, 19619	AK050091	100503265	---	7.84	7.23	Yes
GSMG0028031	---	down	1.53	3.81E-02	chr4(-):87813644-87816618	UCSC	---	AK048577	---	---	6.86	6.25	No
GSMG0027705	Enho	down	1.53	1.32E-02	chr4(-):41638144-41640303	UCSC	energy homeostasis associated	AK009710	69638	ENSMUSG00000028445	8.73	8.12	Yes
GSMG0022508	Ehmt1	down	1.53	1.62E-02	chr2(-):24790769-24919614	UCSC	euchromatic histone methyltransferase 1	NM_172545	77683	ENSMUSG00000036893	9.66	9.05	No
GSMG0021553	Katnbl1 // Slc12a6	down	1.52	1.56E-03	chr2(+):112261153-112414237	UCSC	katanin p80 subunit B like 1 // solute carrier family 12, member 6	BC062099	107723 // 72425	USG00000027130 // ENSMUSG00000	7.66	7.05	No
GSMG0002625	---	down	1.52	6.28E-03	chr1(-):189913339-189915959	UCSC	---	AK089469	---	---	8.87	8.27	No
GSMG0021482	Abtb2	down	1.52	1.15E-02	chr2(+):103566310-103718423	UCSC	ankyrin repeat and BTB (POZ) domain containing 2	NM_178890	99382	ENSMUSG00000032724	8.96	8.36	No
GSMG0019340	Rasgrp2	down	1.52	1.07E-02	chr19(+):6400113-6415223	UCSC	RAS, guanyl releasing protein 2	NM_011242	19395	ENSMUSG00000032946	10.46	9.85	No
GSMG0001801	Fam124b	down	1.52	3.83E-02	chr1(-):80198699-80213945	UCSC	family with sequence similarity 124, member B	NM_173425	241128	ENSMUSG00000043230	7.92	7.32	Yes
GSMG0014126	Cyp11b2	down	1.52	1.84E-02	chr15(-):74851010-74856318	UCSC	cytochrome P450, family 11, subfamily b, polypeptide 2	NM_009991	13072	ENSMUSG00000022589	7.75	7.14	Yes
GSMG0012646	---	down	1.52	1.46E-02	chr14(-):73259203-73261092	UCSC	---	AK041268	---	---	8.05	7.45	No
GSMG0012237	Ccser2	down	1.52	7.18E-03	chr14(-):36874936-36968764	UCSC	coiled-coil serine rich 2	NM_027045	72972	ENSMUSG00000058690	10.51	9.91	No
GSMG0011391	Tnnc1	down	1.52	3.99E-02	chr14(+):31208312-31211711	UCSC	troponin C, cardiac/slow skeletal	NM_009393	21924	ENSMUSG00000091898	7.06	6.46	Yes
GSMG0010335	Mrpl32	down	1.52	1.33E-02	chr13(-):14609913-14613072	UCSC	mitochondrial ribosomal protein L32	AK078324	75398	ENSMUSG00000015672	9.65	9.04	No
GSMG0009595	Mboat1	down	1.52	1.46E-02	chr13(+):30136490-30246694	UCSC	membrane bound O-acyltransferase domain containing 1	NM_153546	218121	ENSMUSG00000038732	7.93	7.33	No
GSMG0009398	Gm5444	down	1.52	1.13E-02	chr13(+):4771649-4836485	UCSC	predicted gene 5444	AK030184	432723	ENSMUSG00000053499	7.43	6.83	No
GSMG0007451	---	down	1.52	2.83E-02	chr11(-):102511793-102515047	UCSC	---	AK137401	---	---	8.39	7.79	No
GSMG0007326	Pgap3	down	1.52	1.31E-02	chr11(-):98388672-98400490	UCSC	post-GPI attachment to proteins 3	NM_001033537	320655	ENSMUSG00000038208	7.73	7.13	Yes
GSMG0042309	---	down	1.52	5.44E-03	chrX(-):6243215-6246439	UCSC	---	AK132784	---	---	7.02	6.42	Yes
GSMG0039964	Faim	down	1.52	4.90E-02	chr9(+):98986373-99002018	UCSC	Fas apoptotic inhibitory molecule	NM_011810	23873	ENSMUSG00000032463	9.02	8.42	No
GSMG0039939	---	down	1.52	3.38E-02	chr9(+):96028624-96030850	UCSC	---	AK081047	---	---	7.78	7.18	Yes
GSMG0035885	Prr12	down	1.52	4.11E-02	chr7(-):45027707-45052881	UCSC	proline rich 12	NM_175022	233210	ENSMUSG00000046574	8.71	8.11	Yes
GSMG0035202	Rnf40	down	1.52	6.42E-03	chr7(+):127588698-127603957	UCSC	ring finger protein 40	NM_172281	233900	ENSMUSG00000030816	9	8.4	No
GSMG0004014	Gtf3c6	down	1.52	4.91E-02	chr10(-):40248381-40257671	UCSC	general transcription factor IIIC, polypeptide 6, alpha	AK162053	67371	ENSMUSG00000019837	8.58	7.98	No
GSMG0031288	Snx8	down	1.52	1.18E-02	chr5(-):140340302-140389255	UCSC	sorting nexin 8	BC037599	231834	ENSMUSG00000029560	10.05	9.45	No
GSMG0030274	---	down	1.52	4.36E-03	chr5(-):25264948-25267255	UCSC	---	AK041834	---	---	8.07	7.47	No
GSMG0026766	Inadl	down	1.52	5.98E-03	chr4(+):98395789-98719603	UCSC	InaD-like (Drosophila)	NM_172696	12695	ENSMUSG00000061859	8.68	8.08	No
GSMG0026426	Spink4	down	1.52	1.60E-02	chr4(+):40920052-40931400	UCSC	serine peptidase inhibitor, Kazal type 4	NM_011463	20731	ENSMUSG00000028415	8.45	7.85	Yes
GSMG0025313	Mccc1	down	1.52	4.78E-02	chr3(-):35959311-36000678	UCSC	methylcrotonoyl-Coenzyme A carboxylase 1 (alpha)	NM_023644	72039	ENSMUSG00000027709	8.73	8.12	No
GSMG0020523	---	down	1.51	4.42E-02	chr19(-):53457475-53459085	UCSC	---	AK041903	---	---	8.54	7.95	No
GSMG0020141	Olfr1497	down	1.51	1.75E-02	chr19(-):13794665-13795609	UCSC	olfactory receptor 1497	NM_146741	258736	ENSMUSG00000044040	7.04	6.44	Yes
GSMG0002367	---	down	1.51	1.63E-02	chr1(-):165230371-165235125	UCSC	---	AK134351	---	---	8.41	7.81	Yes
GSMG0018165	---	down	1.51	8.76E-03	chr18(+):31844998-31846079	UCSC	---	AK138057	---	---	9.79	9.19	No
GSMG0017930	Lrpprc	down	1.51	2.70E-02	chr17(-):84705249-84790790	UCSC	leucine-rich PPR-motif containing	NM_028233	72416	ENSMUSG00000024120	8.96	8.37	No
GSMG0017647	---	down	1.51	1.50E-02	chr17(-):50807155-50808268	UCSC	---	AK051637	---	---	7.9	7.31	Yes
GSMG0050827	Tns1	down	1.51	1.02E-02	chr1(-):73910231-74124447	UCSC	tensin 1	NM_027884	21961	ENSMUSG00000055322	7.77	7.17	No
GSMG0014693	1810007I06Rik	down	1.51	1.52E-02	chr16(+):13729835-13739471	UCSC	RIKEN cDNA 1810007I06 gene	AK007362	76684	ENSMUSG00000086550	9.33	8.74	Yes
GSMG0014674	Snx29	down	1.51	3.69E-02	chr16(+):11322904-11755473	UCSC	sorting nexin 29	NM_028964	74478	ENSMUSG00000071669	10.48	9.89	Yes
GSMG0014182	---	down	1.51	2.11E-02	chr15(-):76858065-76860750	UCSC	---	AK133633	---	---	7.22	6.63	No
GSMG0014144	Eef1d	down	1.51	3.54E-02	chr15(-):75894796-75909340	UCSC	eukaryotic translation elongation factor 1 delta (guanine nucleotide exchange	NM_029663	66656	ENSMUSG00000055762	9.56	8.96	Yes
GSMG0012845	---	down	1.51	3.34E-03	chr14(-):114940227-114945948	UCSC	---	AK007101	---	---	6.5	5.91	Yes
GSMG0012563	---	down	1.51	3.19E-02	chr14(-):66252745-66256368	UCSC	---	AK137768	---	---	9.39	8.81	Yes
GSMG0010631	Ptpdc1	down	1.51	2.47E-02	chr13(-):48577870-48625665	UCSC	protein tyrosine phosphatase domain containing 1	AK030415	218232	ENSMUSG00000038042	7.74	7.15	No
GSMG0000739	---	down	1.51	3.08E-03	chr1(+):130473743-130475313	UCSC	---	AK144079	---	---	6.91	6.32	Yes
GSMG0006329	Tbrg4	down	1.51	2.43E-02	chr11(-):6615599-6626067	UCSC	transforming growth factor beta regulated gene 4	NM_134011	21379	ENSMUSG00000000384	10.12	9.53	No
GSMG0006169	Gm10099	down	1.51	3.69E-02	chr11(+):118029251-118032431	UCSC	predicted gene 10099	AK084985	791367	---	7.58	6.98	Yes
GSMG0006092	Gpr142	down	1.51	1.48E-02	chr11(+):114798917-114806755	UCSC	G protein-coupled receptor 142	BC146544	217302	ENSMUSG00000034677	6.58	5.99	Yes
GSMG0006068	1700023C21Rik	down	1.51	2.91E-02	chr11(+):109845874-109848476	UCSC	RIKEN cDNA 1700023C21 gene	AK006264	73257	ENSMUSG00000087393	7.75	7.16	No
GSMG0042100	Armcx1	down	1.51	1.09E-02	chrX(+):134717938-134721910	UCSC	armadillo repeat containing, X-linked 1	NM_030066	78248	ENSMUSG00000033460	6.88	6.29	Yes
GSMG0041037	---	down	1.51	2.47E-02	chr9(-):77249180-77252134	UCSC	---	AK132372	---	---	8.43	7.84	Yes
GSMG0051105	Gm8883	down	1.51	3.48E-02	chr1(+):71888112-71891204	UCSC	predicted gene 8883	NR_027659	667931	---	8	7.41	No
GSMG0035730	Zfp82	down	1.51	2.24E-02	chr7(-):30056036-30072823	UCSC	zinc finger protein 82	NM_177889	330502	ENSMUSG00000058447	6.75	6.16	No
GSMG0034813	---	down	1.51	4.96E-02	chr7(+):93006981-93104789	UCSC	---	AK032083	---	---	7.03	6.43	No
GSMG0051180	Supt4b	down	1.51	4.35E-02	chr10(-):31413726-31414435	UCSC	suppressor of Ty 4B	NM_011509	100041294	---	7.49	6.9	No
GSMG0030299	---	down	1.51	5.30E-03	chr5(-):29276002-29280297	UCSC	---	AK147752	---	---	7.07	6.48	Yes
GSMG0029624	---	down	1.51	4.15E-02	chr5(+):112367212-112368472	UCSC	---	AK016815	---	---	7.66	7.07	No
GSMG0028884	A630072M18Rik	down	1.51	2.42E-02	chr5(+):20950988-20956398	UCSC	RIKEN cDNA A630072M18 gene	NR_030699	320770	---	8.23	7.63	No
GSMG0028559	Pqlc2	down	1.51	5.44E-03	chr4(-):139294030-139310700	UCSC	PQ loop repeat containing 2	AK081661	212555	ENSMUSG00000028744	8.48	7.88	Yes
GSMG0027431	Klhl21	down	1.51	4.40E-02	chr4(+):152008890-152017679	UCSC	kelch-like 21	AK154493	242785	ENSMUSG00000073700	8.19	7.59	No
GSMG0025693	Lce1h	down	1.51	3.25E-02	chr3(-):92763215-92765066	UCSC	late cornified envelope 1H	AK010064	67718	ENSMUSG00000049593	9.47	8.87	Yes
GSMG0002909	Slc22a16	down	1.51	3.50E-02	chr10(+):40570336-40604132	UCSC	solute carrier family 22 (organic cation transporter), member 16	DQ164804	70840	ENSMUSG00000019834	7.07	6.47	Yes
GSMG0024323	---	down	1.51	1.11E-02	chr3(+):55483788-55487180	UCSC	---	AK047591	---	---	7.25	6.65	Yes
GSMG0023882	Ncoa5	down	1.51	2.38E-03	chr2(-):165000358-165034817	UCSC	nuclear receptor coactivator 5	AK173226	228869	ENSMUSG00000039804	8.78	8.19	Yes
GSMG0022545	Sohlh1	down	1.51	4.54E-02	chr2(-):25843005-25847248	UCSC	spermatogenesis and oogenesis specific basic helix-loop-helix 1	NM_001001714	227631	ENSMUSG00000059625	8.5	7.91	Yes
GSMG0022530	---	down	1.51	1.35E-02	chr2(-):25462811-25465902	UCSC	---	AK030988	---	---	7.57	6.97	Yes
GSMG0020579	Eif3a	down	1.5	1.07E-02	chr19(-):60761116-60790701	UCSC	eukaryotic translation initiation factor 3, subunit A	NM_010123	13669	ENSMUSG00000024991	9.23	8.64	Yes
GSMG0017694	Ptprs	down	1.5	4.73E-02	chr17(-):56412426-56476480	UCSC	protein tyrosine phosphatase, receptor type, S	NM_011218	19280	ENSMUSG00000013236	8.54	7.95	No
GSMG0014935	---	down	1.5	4.96E-02	chr16(+):35781603-35783486	UCSC	---	AK143480	---	---	7.33	6.74	Yes
GSMG0014618	Rbfox1	down	1.5	6.72E-03	chr16(+):5884793-7412479	UCSC	RNA binding protein, fox-1 homolog (C. elegans) 1	NM_021477	268859	ENSMUSG00000008658	8.66	8.07	Yes
GSMG0043658	Ankrd23	down	1.5	3.64E-03	chr1(-):36530189-36539358	UCSC	ankyrin repeat domain 23	BC022973	78321	ENSMUSG00000067653	8.12	7.53	Yes
GSMG0011089	Rgs7bp	down	1.5	3.77E-02	chr13(-):104947153-105054930	UCSC	regulator of G-protein signalling 7 binding protein	NM_029879	52882	ENSMUSG00000021719	7.93	7.35	Yes
GSMG0011067	---	down	1.5	1.90E-02	chr13(-):103263868-103265303	UCSC	---	AK084371	---	---	7.21	6.62	Yes
GSMG0009661	Snrnp48	down	1.5	3.85E-02	chr13(+):38204939-38227663	UCSC	small nuclear ribonucleoprotein 48 (U11/U12)	NM_026382	67797	ENSMUSG00000021431	9.33	8.74	Yes
GSMG0006879	Polr2a	down	1.5	9.38E-03	chr11(-):69734110-69758634	UCSC	polymerase (RNA) II (DNA directed) polypeptide A	U37500	20020	ENSMUSG00000005198	10.29	9.71	No
GSMG0005665	Taf15	down	1.5	3.70E-02	chr11(+):83473086-83506742	UCSC	TAF15 RNA polymerase II, TATA box binding protein (TBP)-associated fact	AK051568	70439	ENSMUSG00000020680	10.65	10.07	No
GSMG0041336	Ptpn23	down	1.5	1.36E-02	chr9(-):110385088-110408210	UCSC	protein tyrosine phosphatase, non-receptor type 23	NM_001081043	104831	ENSMUSG00000036057	9.27	8.68	No
GSMG0036881	Mob2	down	1.5	4.06E-02	chr7(-):142008523-142061066	UCSC	MOB kinase activator 2	AK135483	101513	ENSMUSG00000025147	9.42	8.83	No
GSMG0035023	Zfp143	down	1.5	4.41E-02	chr7(+):110061215-110095392	UCSC	zinc finger protein 143	NM_009281	20841	ENSMUSG00000061079	7.96	7.38	No
GSMG0052153	---	down	1.5	2.50E-03	chr7(+):27153714-27158564	UCSC	---	NM_001100184	---	---	7.91	7.32	Yes
GSMG0003922	---	down	1.5	2.79E-02	chr10(-):26357303-26360033	UCSC	---	AK047379	---	---	6.58	5.99	Yes
GSMG0025892	Cym	down	1.5	1.40E-02	chr3(-):107211295-107221732	UCSC	chymosin	NM_001111143	229697	ENSMUSG00000046213	7.77	7.18	Yes
GSMG0025819	Atp1a1	down	1.5	6.74E-03	chr3(-):101576219-101604707	UCSC	ATPase, Na+/K+ transporting, alpha 1 polypeptide	NM_144900	11928	ENSMUSG00000033161	10.7	10.12	No

Table S3A. Overlapping human RAEB1 genes upregulated post ABT-737 treatment of HR-MDS mice

Probe Set ID	Gene Symbol	Chromosomal Location	Adj. p-value in MDS RAEB1 vs controls	Fold change in MDS RAEB1 vs controls
215721_at	IGHG1	chr14q32.33	1.55E-08	-1.6364373
224598_at	MGAT4B	chr5q35	6.61E-06	1.92411
200853_at	H2AFZ	chr4q24	7.38E-06	-1.4377519
228361_at	E2F2	chr1p36	9.91E-06	-2.296033
205011_at	VWA5A	chr11q24.1	4.65E-05	1.9165103
203066_at	CHST15	chr10q26	5.54E-05	-5.07447
213911_s_at	H2AFZ	chr4q24	1.18E-04	-1.3124048
205640_at	ALDH3B1	chr11q13	2.39E-04	1.814213
226671_at	LAMP2	chrXq24	2.85E-04	2.2320046
229356_x_at	INO80	chr15q15.1	3.10E-04	-1.298237
203042_at	LAMP2	chrXq24	8.37E-04	1.7357126
226810_at	OGFRL1	chr6q13	9.34E-04	-1.6472691
218699_at	RAB7L1	chr1q32	9.83E-04	1.664379
201315_x_at	IFITM2	chr11p15.5	1.15E-03	1.7209625
1552680_a_at	CASC5	chr15q14	1.31E-03	-1.7190827
210102_at	VWA5A	chr11q24.1	1.38E-03	1.8063465
212093_s_at	MTUS1	chr8p22	1.80E-03	-1.3051406
211004_s_at	ALDH3B1	chr11q13	1.93E-03	1.3285067
205034_at	CCNE2	chr8q22.1	2.36E-03	-1.820539
1554696_s_at	TYMS	chr18p11.32	2.49E-03	-1.8673539
225707_at	ARL6IP6	chr2q23.3	2.71E-03	-1.425871
218983_at	C1RL	chr12p13.31	2.99E-03	2.0180602
209891_at	SPC25	chr2q31.1	3.23E-03	-2.1531157
214637_at	OSM	chr22q12.2	3.72E-03	-1.3015238
211743_s_at	PRG2	chr11q12	3.78E-03	-4.9228597
209130_at	SNAP23	chr15q14	3.79E-03	1.5148033
211814_s_at	CCNE2	chr8q22.1	4.02E-03	-1.5061644
223009_at	LAMTOR1	chr11q13.4	4.49E-03	1.5385216
209879_at	SELPLG	chr12q24	4.79E-03	1.6893362
212801_at	CIT///MIR1178	chr12q24///chr12q24.23	4.98E-03	-1.3512969
218322_s_at	ACSL5	chr10q25.1-q25.2	5.75E-03	1.4210818
206200_s_at	ANXA11	chr10q23	5.94E-03	1.2997527
225469_at	LYRM5	chr12p12.1	6.27E-03	1.3965974
209890_at	TSPAN5	chr4q23	6.46E-03	1.7231414
201087_at	PXN	chr12q24.31	6.72E-03	1.6160686
205967_at	HIST1H4A///HIST1H4B/	chr12p12.3///chr1q21///chr1q2	7.14E-03	-1.2961758
202788_at	MAPKAPK3	chr3p21.3	7.46E-03	1.46537
217684_at	TYMS	chr18p11.32	7.93E-03	-1.3347185
218039_at	NUSAP1	chr15q15.1	8.08E-03	-1.6779078
201140_s_at	RAB5C	chr17q21.2	8.18E-03	1.3126237
214075_at	NENF	chr1q32.3	8.22E-03	1.3216177
202185_at	PLOD3	chr7q22	8.25E-03	1.3487327
225601_at	HMGB3	chrXq28	8.92E-03	-1.5650258
203041_s_at	LAMP2	chrXq24	9.02E-03	1.5419428
201463_s_at	TALDO1	chr11p15.5-p15.4	9.40E-03	1.2362212
221840_at	PTPRE	chr10q26	9.71E-03	-2.0405433
203554_x_at	PTTG1	chr5q35.1	1.00E-02	-1.570125
201349_at	SLC9A3R1	chr17q25.1	1.15E-02	1.3760096
228606_at	TCTEX1D2	chr3q29	1.17E-02	-1.5617791
209930_s_at	NFE2	chr12q13	1.25E-02	1.8532964
229164_s_at	ABTB1	chr3q21	1.26E-02	1.3906038
202589_at	TYMS	chr18p11.32	1.33E-02	-1.4749583
216210_x_at	TRIOBP	chr22q13.1	1.34E-02	1.2567328
217794_at	PRR13	chr12q12	1.35E-02	1.2779747
244874_at	CHST15	chr10q26	1.46E-02	-1.3251104
228754_at	SLC6A6	chr3p25.1	1.58E-02	1.4470309
214581_x_at	TNFRSF21	chr6p21.1	1.63E-02	-1.5264643
201601_x_at	IFITM1///IFITM2	chr11p15.5	1.67E-02	1.9916847
209276_s_at	GLRX	chr5q14	1.73E-02	-1.6827419
238515_at	NUDT16	chr3q22.1	1.75E-02	1.216054
220189_s_at	MGAT4B	chr5q35	1.76E-02	1.499337
230860_at	CEP19	chr3q29	1.76E-02	1.7053223
208805_at	KIAA0391///PSMA6	chr14q13///chr14q13.2	1.88E-02	-1.2259935
201291_s_at	TOP2A	chr17q21-q22	1.89E-02	-1.8839478
223396_at	TMEM60	chr7q11.23	1.90E-02	1.3709334
201292_at	TOP2A	chr17q21-q22	1.90E-02	-1.8747588
226636_at	PLD1	chr3q26	1.93E-02	2.0031781
239704_at	RNF144B	chr6p22.3	1.94E-02	-1.3744094
219213_at	JAM2	chr21q21.2	2.00E-02	-1.6419456
208808_s_at	HMGB2	chr4q31	2.03E-02	-1.2164421
224806_at	TRIM25	chr17q23.2	2.04E-02	1.4453377
219345_at	BOLA1	chr1q21	2.11E-02	1.4743003
209804_at	DCLRE1A	chr10q25.1	2.11E-02	1.5185239
206662_at	GLRX	chr5q14	2.12E-02	-1.348599

Probe Set ID	Gene Symbol	Chromosomal Location	Adj. p-value in MDS RAEB1 vs controls	Fold change in MDS RAEB1 vs controls
201055_s_at	HNRNPA0	chr5q31	2.14E-02	-1.3281915
209221_s_at	OSBPL2	chr20q13.3	2.41E-02	1.3833718
201061_s_at	STOM	chr9q34.1	2.45E-02	1.5238088
204174_at	ALOX5AP	chr13q12	2.52E-02	-1.7078526
207157_s_at	GNG5	chr1p22	2.56E-02	1.2661464
218700_s_at	RAB7L1	chr1q32	2.63E-02	1.4486791
202117_at	ARHGAP1	chr11p11.2	2.68E-02	1.2605814
219978_s_at	NUSAP1	chr15q15.1	2.72E-02	-1.8081737
225387_at	TSPAN5	chr4q23	2.72E-02	1.6109072
204186_s_at	PPID	chr4q31.3	2.73E-02	1.4319415
203744_at	HMGB3	chrXq28	2.78E-02	-1.4225464
217513_at	MILR1	chr17q23.3	2.78E-02	-1.3625568
226453_at	RNASEH2C	chr11q13.1	2.80E-02	-1.282316
1553158_at	CEP19	chr3q29	2.86E-02	1.1749215
220811_at	PRG3	chr11q12	2.86E-02	-1.4608735
241074_at	IGHG1	chr14q32.33	2.90E-02	-1.3180652
60471_at	RIN3	chr14q32.12	2.94E-02	1.4030824
235054_at	NUDT16	chr3q22.1	3.26E-02	1.3617315
226392_at	RASA2	chr3q22-q23	3.28E-02	-1.3711581
200974_at	ACTA2	chr10q23.3	3.32E-02	1.5947651
209222_s_at	OSBPL2	chr20q13.3	3.36E-02	1.3409412
215723_s_at	PLD1	chr3q26	3.42E-02	1.4424802
203214_x_at	CDK1	chr10q21.1	3.46E-02	-1.5577782
229423_at	CHEK1	chr11q24.2	3.49E-02	1.2734343
1553043_a_at	CD300LF	chr17q25.1	3.49E-02	1.5126432
208080_at	AURKA	chr20q13	3.50E-02	-1.1045762
229083_at	HNRNPA0		3.67E-02	-1.474962
208780_x_at	VAPA	chr18p11.22	3.67E-02	1.1893046
1555989_at	DAAM1	chr14q23.1	3.69E-02	1.7623738
207042_at	E2F2	chr1p36	3.90E-02	-1.1869624
229836_s_at	NUDT4	chr12q21	3.93E-02	-1.1769974
226692_at	HYPK///MIR1282///SER	chr15q15.3	3.96E-02	1.2763637
205312_at	SPI1	chr11p11.2	4.01E-02	1.1553155
209288_s_at	CDC42EP3	chr2p21	4.06E-02	1.6809378
217967_s_at	FAM129A	chr1q25	4.08E-02	1.5568438
213226_at	CCNA2	chr4q27	4.12E-02	-1.4719989
214472_at	HIST1H2AD///HIST1H3/	chr6p21.3///chr6p22.1///chr6p	4.14E-02	1.9298923
205180_s_at	ADAM8	chr10q26.3	4.15E-02	1.6934518
214539_at	SERPINB10	chr18q21.3	4.20E-02	-1.5908092
219959_at	MOCOS	chr18q12	4.24E-02	1.2008828
217169_at	IGHA1///IGHG1///IGHN	chr14q32.33	4.28E-02	-1.2491151
243570_at	SPCS2	chr11q13.4	4.38E-02	-1.272085
203175_at	RHOG	chr11p15.5-p15.4	4.40E-02	1.4218543
1569827_at	ATG7	chr3p25.3	4.45E-02	1.2997227
1569289_at	BIVM	chr13q33.1	4.50E-02	1.0935669
219457_s_at	RIN3	chr14q32.12	4.57E-02	1.3690063
202252_at	RAB13	chr1q21.2	4.62E-02	1.2472285
241994_at	XDH	chr2p23.1	4.64E-02	-1.1984246
203388_at	ARRB2	chr17p13	4.64E-02	1.3192865
216598_s_at	CCL2	chr17q11.2-q12	4.64E-02	-1.8957943
228341_at	NUDT16	chr3q22.1	4.64E-02	1.4293232
177_at	PLD1	chr3q26	4.65E-02	1.3124759
205932_s_at	MSX1	chr4p16.2	4.66E-02	-1.2350941
1555529_at	RNH1	chr11p15.5	4.70E-02	-1.2054332
229753_at	POU2F1	chr1q24.2	4.70E-02	-1.171224
215779_s_at	HIST1H2BC///HIST1H2B	chr6p21.3///chr6p22.1///chr8q	4.70E-02	1.9316245
1554091_a_at	TIRAP	chr11q24.2	4.83E-02	-1.272938
210559_s_at	CDK1	chr10q21.1	4.86E-02	-1.5590221
203370_s_at	PDLIM7	chr5q35.3	4.86E-02	1.1719533
203967_at	CDC6	chr17q21.3	4.88E-02	-1.7062597
225579_at	PQLC3	chr2p25.1	4.89E-02	1.4525129
225252_at	SRXN1	chr20p13	4.92E-02	1.4977945

Supplementary S3B. Overlapping human RAEB1 genes downregulated post ABT-737 treatment of HR-MDS mice

Probe Set ID	Gene Symbol	Chromosomal Location	Adj. p-value in MDS RAEB1 vs controls	Fold change in MDS RAEB1 vs controls
211596_s_at	LRIG1	chr3p14	7.81E-10	-7.319766
221234_s_at	BACH2	chr6q15	1.01E-09	-8.655297
227173_s_at	BACH2	chr6q15	2.20E-08	-2.566293
206492_at	FHIT	chr3p14.2	1.30E-04	-1.8293651
218696_at	EIF2AK3	chr2p12	5.47E-04	-1.7064792
215500_at	SNX29	chr16p13.13-p13.12	1.37E-03	-1.2144035
205316_at	SLC15A2	chr3q13.33	1.37E-03	2.140229
202970_at	DYRK2	chr12q15	1.44E-03	-1.2543201
229044_at	LOC101060409///NUDT17	chr1q21.1	1.97E-03	1.2629476
235918_x_at	CEP97	chr3q12.3	2.47E-03	-1.5556768
221140_s_at	GPR132	chr14q32.3	3.31E-03	-1.2219541
203068_at	KLHL21	chr1p36.31	3.37E-03	1.5680406
39248_at	AQP3	chr9p13	3.41E-03	2.2085671
223887_at	GPR132	chr14q32.3	3.86E-03	-1.6558092
223150_s_at	PTPN23	chr3p21.3	5.19E-03	1.3006246
204169_at	IMPDH1	chr7q31.3-q32	6.86E-03	1.3246675
228113_at	RAB37	chr17q25.1	7.93E-03	1.6525658
223592_s_at	RNF135	chr17q11.2	8.61E-03	1.496068
236247_at	NSUN4	chr1p34	1.01E-02	1.2475783
223591_at	RNF135	chr17q11.2	1.16E-02	1.4932797
213225_at	PPM1B	chr2p21	1.41E-02	-1.3777004
211982_x_at	XPO6	chr16p11.2	1.51E-02	1.2533848
204642_at	S1PR1	chr1p21	1.65E-02	-1.6023331
204675_at	SRD5A1	chr5p15	1.67E-02	1.5194131
227379_at	MBOAT1	chr6p22.3	1.75E-02	1.5789479
200846_s_at	PPP1CA	chr11q13	1.76E-02	-1.4108629
212275_s_at	SRCAP	chr16p11.2	1.79E-02	1.1690745
202321_at	GGPS1	chr1q43	1.84E-02	1.4565029
204890_s_at	LCK	chr1p34.3	1.89E-02	-1.2869132
203950_s_at	CLCN6	chr1p36	1.98E-02	1.2969456
207445_s_at	CCR9	chr3p21.3	2.05E-02	-1.2206492
221246_x_at	TNS1	chr2q35-q36	2.34E-02	1.3437831
202322_s_at	GGPS1	chr1q43	2.40E-02	1.2884352
204276_at	TK2	chr16q22-q23.1	2.69E-02	1.4356428
210959_s_at	SRD5A1	chr5p15	2.70E-02	1.413591
218534_s_at	AGGF1	chr5q13.3	2.75E-02	1.5003092
237230_at	GPHA2	chr11q13.1	3.05E-02	-1.1451286
226438_at	SNTB1	chr8q23-q24	3.29E-02	1.4518427
38766_at	LOC100862671///SRCAP	chr16p///chr16p11.2	3.67E-02	-1.1561667
211423_s_at	SC5D	chr11q23.3	3.68E-02	-1.3812652
242687_at	FAM160A1	chr4q31.3	3.68E-02	-1.1685913
1555781_at	PQLC2	chr1p36.13	3.78E-02	1.2015547
200623_s_at	CALM1///CALM2///CALM3	chr14q32.11///chr19q13.2-q13.3///chr2p21	3.85E-02	1.2910935
221748_s_at	TNS1	chr2q35-q36	3.87E-02	1.7998209
204891_s_at	LCK	chr1p34.3	3.87E-02	-1.5767831
206272_at	RAB4A///SPHAR	chr1q42-q43///chr1q42.13	3.92E-02	1.5093559
204057_at	IRF8	chr16q24.1	3.96E-02	-2.6401112
223868_s_at	WWOX	chr16q23.3-q24.1	4.06E-02	-1.2512114
1552628_a_at	HERPUD2	chr7p14.2	4.06E-02	1.3884828
204472_at	GEM	chr8q13-q21	4.06E-02	-1.8895009
242341_x_at	GLYCTK	chr3p21.1	4.36E-02	-1.1604922
1553366_s_at	ANKRD23///ANKRD39	chr2q11.2	4.80E-02	-1.2471164
204277_s_at	TK2	chr16q22-q23.1	4.85E-02	1.2179564

Supplementary Table S4A. Pathway analysis using GO

Term Type	GO ID with Link	Go Term	Nb Genes in Term	Nb Regulated Genes (Up / Down)	P-Value
cellular_component	GO:0070062	extracellular exosome	2702	259 (209/50)	1.03E-14
cellular_component	GO:0070469	respiratory chain	58	17 (17/0)	3.52E-05
cellular_component	GO:0005743	mitochondrial inner membrane	389	49 (41/8)	1.30E-04
cellular_component	GO:0016020	membrane	6996	481 (355/126)	2.48E-04
cellular_component	GO:0005739	mitochondrion	1731	144 (117/27)	9.50E-04
cellular_component	GO:0005747	mitochondrial respiratory chain complex I	49	13 (13/0)	1.71E-03
biological_process	GO:0055114	oxidation-reduction process	677	72 (61/11)	3.34E-03
cellular_component	GO:0000228	nuclear chromosome	53	13 (12/1)	3.42E-03
cellular_component	GO:0005634	nucleus	6051	410 (275/135)	6.54E-03
cellular_component	GO:0005737	cytoplasm	6653	445 (314/131)	7.69E-03
cellular_component	GO:0005654	nucleoplasm	1943	151 (112/39)	8.55E-03
cellular_component	GO:0030529	intracellular ribonucleoprotein complex	322	35 (23/12)	2.98E-02
cellular_component	GO:0005753	mitochondrial proton-transporting ATP synthase complex	20	7 (7/0)	3.28E-02
cellular_component	GO:0042470	melanosome	102	16 (15/1)	3.29E-02
cellular_component	GO:0005751	mitochondrial respiratory chain complex IV	14	6 (6/0)	3.31E-02
cellular_component	GO:0015629	actin cytoskeleton	202	25 (20/5)	3.35E-02

Supplementary Table S4B. Pathway analysis using KEGG

Link to KEGG Pathway	Pathway Description (KEGG)	Nb Genes in Pathway	Nb Regulated Genes (Up / Down)	P-Value (All)	P-Value (Up)	P-Value (Down)	Min P-Value
mmu00190	Oxidative phosphorylation	139	32 (32/0)	6.15E-07	6.12E-11	NA	6.12E-11
mmu05012	Parkinson's disease	149	31 (30/1)	4.55E-06	6.70E-09	NA	6.70E-09
mmu05010	Alzheimer's disease	177	35 (32/3)	3.00E-06	1.58E-08	NA	1.58E-08
mmu05016	Huntington's disease	198	34 (32/2)	7.69E-05	2.09E-07	NA	2.09E-07
mmu04932	Non-alcoholic fatty liver disease (NAFLD)	157	29 (26/3)	1.02E-04	4.17E-06	NA	4.17E-06
mmu01100	Metabolic pathways	1278	86 (86/0)	NA	2.29E-02	NA	2.29E-02
mmu04260	Cardiac muscle contraction	77	15 (12/3)	2.58E-02	3.42E-02	NA	2.58E-02
mmu04380	Osteoclast differentiation	126	20 (14/6)	3.00E-02	NA	NA	3.00E-02
mmu03050	Proteasome	45	9 (9/0)	NA	3.46E-02	NA	3.46E-02

Supplementary Table S5A. EPIGENETIC GENES REGULATED UPON ABT-737 treatment of HR-MDS MICE as defined by the pathway analysis

FAST DB STABLE ID	Gene Symbol	Gene Name	TRIPLE (-AB	TRIPLE (+AB	Regulation	Fold-Change	P-Value
GSMG0000801	Kdm5b	lysine (K)-specific demethylase 5B	8.73937	8.53116667	down	1.15524859	6.12E-02
GSMG0000867	Uchl5	ubiquitin carboxyl-terminal esterase L5	9.38678333	9.34647667	down	1.02833239	8.17E-01
GSMG0001012	Tada1	transcriptional adaptor 1	9.16585	9.11412167	down	1.0365059	4.88E-01
GSMG0001466	Kansl3	KAT8 regulatory NSL complex subunit 3	8.20276667	8.12964333	down	1.05199171	5.40E-01
GSMG0001898	Hdac4	histone deacetylase 4	8.21319167	8.18354833	down	1.02075974	8.51E-01
GSMG0002218	Rnf2	ring finger protein 2	9.13115667	8.800315	down	1.25774693	6.18E-03
GSMG0002542	Smyd3	SET and MYND domain containing 3	9.01874667	8.61073667	down	1.32685434	1.39E-01
GSMG0002624	Smyd2	SET and MYND domain containing 2	8.24851333	8.06465333	down	1.13591903	5.03E-01
GSMG0002875	Hdac2	histone deacetylase 2	9.61920333	9.4726	down	1.10696019	4.43E-01
GSMG0003275	Dot1l	DOT1-like, histone H3 methyltransferase (S. cerevisiae)	8.84743	8.59192	down	1.19375767	3.01E-01
GSMG0003322	Tdg	thymine DNA glycosylase	8.90825	8.8338	down	1.05295954	7.06E-01
GSMG0004171	Tet1	tet methylcytosine dioxygenase 1	6.86633833	6.74222167	down	1.08984024	3.73E-01
GSMG0004178	Sirt1	sirtuin 1	10.53904	10.4080833	down	1.09501958	4.97E-01
GSMG0004397	Appl2	adaptor protein, phosphotyrosine interaction, PH domain and leucine zipper containing 2	8.16846333	8.00723	down	1.11824269	1.44E-01
GSMG0006640	Phf15	PHD finger protein 15	8.97151	8.83629167	down	1.09825901	4.82E-01
GSMG0007127	Tada2a	transcriptional adaptor 2A	8.00566167	7.79627167	down	1.15619922	6.13E-02
GSMG0007333	Med24	mediator complex subunit 24	7.47807667	7.35796667	down	1.08681773	7.64E-01
GSMG0007341	Smarce1	SWI/SNF related, matrix associated, actin dependent regulator of chromatin, subfamily e, member 1	11.34	10.9378	down	1.32152159	7.99E-02
GSMG0007342	Smarce1	SWI/SNF related, matrix associated, actin dependent regulator of chromatin, subfamily e, member 1	9.56017333	8.80982833	down	1.68219506	1.38E-01
GSMG0007440	Hdac5	histone deacetylase 5	8.34755	7.91014833	down	1.35416324	2.79E-02
GSMG0007491	Kansl1	KAT8 regulatory NSL complex subunit 1	11.1487333	11.0178667	down	1.09495127	2.55E-01
GSMG0007509	Smarcd2	SWI/SNF related, matrix associated, actin dependent regulator of chromatin, subfamily d, member 2	9.96696	9.5251	down	1.35835446	8.28E-02
GSMG0007782	Dnmt3a	DNA methyltransferase 3A	9.02963333	8.69854333	down	1.25796345	8.16E-02
GSMG0008304	2410016O06Rik	RIKEN cDNA 2410016O06 gene	8.52067667	8.390925	down	1.09410536	5.76E-01
GSMG0008531	Meg3	maternally expressed 3	8.47678333	8.29056333	down	1.13777872	2.46E-01
GSMG0008602	Mta1	metastasis associated 1	9.22427333	9.10266667	down	1.08794579	5.65E-01
GSMG0009156	Alkbh1	alkB, alkylation repair homolog 1 (E. coli)	10.2158	9.87177333	down	1.26929435	4.56E-01
GSMG0009638	Cdyl	chromodomain protein, Y chromosome-like	8.62336667	8.43405667	down	1.14021825	4.67E-02
GSMG0009726	Kdm1b	lysine (K)-specific demethylase 1B	9.77930333	9.49234667	down	1.22006386	1.44E-01
GSMG0010633	Phf2	PHD finger protein 2	8.29865333	8.29842333	down	1.00015944	9.98E-01
GSMG0011295	Kat6b	K(lysine) acetyltransferase 6B	9.635285	9.401755	down	1.17570817	2.33E-01
GSMG0011523	Apex1	apurinic/apyrimidinic endonuclease 1	10.1120933	9.64325	down	1.38399942	2.29E-02
GSMG0011638	Sap18	Sin3-associated polypeptide 18	10.836	10.77525	down	1.04300784	5.54E-01
GSMG0012146	Appl1	adaptor protein, phosphotyrosine interaction, PH domain and leucine zipper containing 1	9.88224667	9.851	down	1.02189479	7.91E-01
GSMG0013625	Smarcd1	SWI/SNF related, matrix associated, actin dependent regulator of chromatin, subfamily d, member 1	9.04684667	8.76899167	down	1.21239096	1.76E-04
GSMG0014262	Cbx7	chromobox 7	8.79586333	8.55835167	down	1.17895746	2.53E-01
GSMG0014365	Hdac10	histone deacetylase 10	8.88003167	8.59996833	down	1.21424819	7.58E-02
GSMG0014468	Kansl2	KAT8 regulatory NSL complex subunit 2	9.5954	9.34235	down	1.19172388	5.80E-02
GSMG0014492	Mcrs1	microspherule protein 1	9.16921333	8.752715	down	1.33468411	4.18E-02
GSMG0014594	Naa60	N(alpha)-acetyltransferase 60, NatF catalytic subunit	9.6372	9.56808333	down	1.04907416	5.85E-01
GSMG0016156	Arid1b	AT rich interactive domain 1B (SWI-like)	8.18807667	8.13705333	down	1.03599952	8.09E-01
GSMG0016416	Phf1	PHD finger protein 1	9.634405	9.479475	down	1.1133676	4.04E-01
GSMG0016565	Ehmt2	euchromatic histone lysine N-methyltransferase 2	10.1752783	10.08228	down	1.06658455	4.29E-01
GSMG0016700	Supt3	suppressor of Ty 3	7.99313	7.94559667	down	1.03349638	6.31E-01
GSMG0016809	Chaf1a	chromatin assembly factor 1, subunit A (p150)	7.89109	7.80971167	down	1.05802839	7.48E-01
GSMG0017150	Prdm9	PR domain containing 9	8.24716	8.0026	down	1.18473139	3.55E-01
GSMG0018592	Cxxc1	CXXC finger 1 (PHD domain)	9.53132667	9.38977333	down	1.10309216	2.55E-01
GSMG0018689	Epc1	enhancer of polycomb homolog 1 (Drosophila)	10.9832	10.0691967	down	1.8842669	2.02E-03
GSMG0019254	Suv420h1	suppressor of variegation 4-20 homolog 1 (Drosophila)	10.6671167	10.0098533	down	1.57708819	4.88E-03
GSMG0019383	Mta2	metastasis-associated gene family, member 2	10.2247667	10.1025433	down	1.08841092	6.33E-01
GSMG0019554	Smarca2	SWI/SNF related, matrix associated, actin dependent regulator of chromatin, subfamily a, member 2	8.94999	8.194765	down	1.68789481	2.19E-02
GSMG0019781	Taf5	TAF5 RNA polymerase II, TATA box binding protein (TBP)-associated factor	8.36191667	8.10216	down	1.19727675	3.30E-01
GSMG0019936	Kdm2a	lysine (K)-specific demethylase 2A	10.1759333	10.16837	down	1.00525627	9.66E-01
GSMG0019983	Kat5	K(lysine) acetyltransferase 5	9.70533333	9.30457333	down	1.3202032	5.09E-02
GSMG0020483	Pcgf6	polycomb group ring finger 6	8.72817	8.58305333	down	1.10582007	2.62E-01
GSMG0021201	Hat1	histone aminotransferase 1	10.2944667	10.18171	down	1.08129237	6.25E-01
GSMG0021969	Csnk2a1	casein kinase 2, alpha 1 polypeptide	10.9903167	10.9613667	down	1.0202693	6.67E-01
GSMG0021999	Dnmt3b	DNA methyltransferase 3B	7.660425	7.52967167	down	1.09486526	3.22E-01
GSMG0022052	Phf20	PHD finger protein 20	9.35409833	8.791575	down	1.47685003	1.22E-03
GSMG0022090	Actr5	ARP5 actin-related protein 5	9.27074	8.66403	down	1.52278261	1.02E-01
GSMG0022178	Ncoa3	nuclear receptor coactivator 3	10.9624667	10.8636	down	1.07093184	4.27E-01
GSMG0022340	Suv39h2	suppressor of variegation 3-9 homolog 2 (Drosophila)	7.735335	7.71569	down	1.01371001	9.41E-01
GSMG0022508	Ehmt1	euchromatic histone methyltransferase 1	9.66375	9.04867667	down	1.53163585	1.62E-02
GSMG0022683	Phf19	PHD finger protein 19	7.975645	7.94985167	down	1.01803935	8.89E-01
GSMG0022972	Cir1	corepressor interacting with RBPJ, 1	10.2379667	10.2057333	down	1.02259391	8.32E-01
GSMG0023282	Elp4	elongation protein 4 homolog (S. cerevisiae)	8.44200333	8.43587	down	1.00426035	9.57E-01
GSMG0023975	Nespos	neuroendocrine secretory protein opposite strand	7.02274833	6.94063667	down	1.05856633	5.49E-01
GSMG0024139	Tbl1xr1	transducin (beta)-like 1X-linked receptor 1	10.8072	10.7211833	down	1.06143547	1.23E-02
GSMG0024198	Actl6a	actin-like 6A	9.59025667	9.34768333	down	1.18310108	6.65E-02
GSMG0024259	Phf17	PHD finger protein 17	8.75542333	8.47559167	down	1.21405322	6.01E-02
GSMG0025380	Setd7	SET domain containing (lysine methyltransferase) 7	9.07773667	8.83925167	down	1.17975313	1.10E-01
GSMG0025742	Setdb1	SET domain, bifurcated 1	9.49357333	9.40303667	down	1.06476619	6.85E-01
GSMG0027109	Phc2	polyhomeotic-like 2 (Drosophila)	9.44808667	9.30038333	down	1.10780452	1.46E-01
GSMG0027409	Rere	arginine glutamic acid dipeptide (RE) repeats	10.238	9.96447667	down	1.20875624	1.60E-01
GSMG0028265	Kdm4a	lysine (K)-specific demethylase 4A	9.84894667	9.53936167	down	1.23935114	3.03E-02
GSMG0028400	Hdac1	histone deacetylase 1	10.373	10.2639	down	1.07855519	4.27E-01
GSMG0028474	Arid1a	AT rich interactive domain 1A (SWI-like)	10.6915167	10.6760167	down	1.0108017	9.45E-01
GSMG0028519	Kdm1a	lysine (K)-specific demethylase 1A	9.52293333	9.32143167	down	1.14989463	3.15E-01
GSMG0028904	Kmt2e	lysine (K)-specific methyltransferase 2E	10.1586667	10.0397667	down	1.08590658	2.79E-01
GSMG0029556	Mtf2	metal response element binding transcription factor 2	9.845515	9.84012	down	1.00374653	9.64E-01
GSMG0029901	Baz1b	bromodomain adjacent to zinc finger domain, 1B	10.7283167	10.63905	down	1.06382929	2.67E-01
GSMG0030275	Kmt2c	lysine (K)-specific methyltransferase 2C	10.1560167	9.836995	down	1.24748431	7.58E-02
GSMG0030334	Supt7l	suppressor of Ty 7-like	8.912705	8.64737333	down	1.20191233	3.98E-02
GSMG0030646	Clock	circadian locomotor output cycles kaput	9.40761333	8.78237667	down	1.54246384	7.40E-02
GSMG0030947	Alkbh2	alkB, alkylation repair homolog 2 (E. coli)	8.19538333	8.12095667	down	1.05294251	7.84E-01
GSMG0031058	Kdm2b	lysine (K)-specific demethylase 2B	9.10787	8.547815	down	1.47432542	2.95E-02
GSMG0031097	Ncor2	nuclear receptor co-repressor 2	8.73793333	8.72849333	down	1.00656476	9.53E-01
GSMG0031556	Ing3	inhibitor of growth family, member 3	10.7367667	10.4536	down	1.21686293	3.10E-01
GSMG0032192	Ruvbl1	RuvB-like protein 1	10.113	9.73507667	down	1.29947001	1.45E-02
GSMG0032294	Setmar	SET domain without mariner transposase fusion	7.66226333	7.45881333	down	1.15144859	1.92E-01
GSMG0032440	Aicda	activation-induced cytidine deaminase	8.79246	8.25955333	down	1.44684128	3.75E-01
GSMG0032476	Acrbp	proacrosin binding protein	9.54625	9.39404333	down	1.11126791	3.29E-01
GSMG0032585	Atf7ip	activating transcription factor 7 interacting protein	11.0648667	11.0245333	down	1.0283514	8.26E-01
GSMG0032607	Aebp2	AE binding protein 2	10.4094	10.1659333	down	1.18383389	2.18E-01
GSMG0033531	Tada3	transcriptional adaptor 3	8.24558333	8.22300667	down	1.01577204	9.28E-01
GSMG0033558	Mbd4	methyl-CpG binding domain protein 4	8.56178	8.20992333	down	1.27620197	3.26E-02
GSMG0033640	Phc1	polyhomeotic-like 1 (Drosophila)	8.44671667	8.29344667	down	1.11208726	1.42E-03
GSMG0033956	Suv420h2	suppressor of variegation 4-20 homolog 2 (Drosophila)	9.28008	9.1862	down	1.06723656	5.15E-01
GSMG0034844	Rsf1	remodeling and spacing factor 1	9.71963	9.5255	down	1.14403406	1.26E-01
GSMG0035151	Kdm8	lysine (K)-specific demethylase 8	7.62627333	7.43750667	down	1.13978891	3.81E-01
GSMG0035416	Tfpt	TCF3 (E2A) fusion partner	8.37542833	8.29681333	down	1.05600378	6.84E-01
GSMG0035751	Kmt2b	lysine (K)-specific methyltransferase 2B	8.61911667	8.15797833	down	1.37662759	3.37E-02
GSMG0035906	Ruvbl2	RuvB-like protein 2	9.89772333	9.7907	down	1.07700379	4.78E-01
GSMG0036670	Ino80e	INO80 complex subunit E	9.48211333	8.96903667	down	1.42709034	1.57E-02
GSMG0036847	Sirt3	sirtuin 3	8.45387667	8.19552667	down	1.19610994	2.94E-02
GSMG0037097	Kat6a	K(lysine) acetyltransferase 6A	10.68125	10.3242833	down	1.28073027	1.25E-02
GSMG0037605	Fto	fat mass and obesity associated	9.38738333	9.13647333	down	1.18995746	5.12E-02
GSMG0037724	Ctcf	CCCTC-binding factor	11.7244167	11.6092667	down	1.08308765	1.15E-01
GSMG0037749	Prmt7	protein arginine N-methyltransferase 7	9.52987167	8.945405	down	1.49948456	6.20E-03
GSMG0038219	Ash2l	ash2 (absent, small, or homeotic)-like (Drosophila)	9.48895667	9.41011333	down	1.05617093	7.27E-01
GSMG0038537	Gatad2a	GATA zinc finger domain containing 2A	9.83605333	9.60494667	down	1.17373496	2.22E-01
GSMG0038643	Smarca5	SWI/SNF related, matrix associated, actin dependent regulator of chromatin, subfamily a, member 5	10.759	10.6968667	down	1.04400841	7.98E-01
GSMG0039054	Taf5l	TAF5-like RNA polymerase II, p300/CBP-associated factor (PCAF)-associated factor	9.53662	9.01506333	down	1.43550332	2.57E-02
GSMG0039215	Carm1	coactivator-associated arginine methyltransferase 1	9.38508333	9.37288667	down	1.00848992	9.26E-01
GSMG0039298	Nfrkb	nuclear factor related to kappa B binding protein	9.49504167	9.07331167	down	1.33953289	2.01E-02
GSMG0040034	Wdr82	WD repeat domain containing 82	11.0535333	10.9421333	down	1.08027603	2.65E-01
GSMG0040130	Smarcc1	SWI/SNF related, matrix associated, actin dependent regulator of chromatin, subfamily c, member 1	9.79821667	9.67701667	down	1.08763916	5.01E-01
GSMG0040672	Kmt2a	lysine (K)-specific methyltransferase 2A	10.6765333	10.6044667	down	1.05122148	8.16E-01
GSMG0041872	Tbl1x	transducin (beta)-like 1 X-linked	9.69875833	9.36659667	down	1.25889824	1.95E-02
GSMG0041966	Taf1	TAF1 RNA polymerase II, TATA box binding protein (TBP)-associated factor	10.2604667	10.0319033	down	1.1716676	3.42E-02
GSMG0042609	Mecp2	methyl CpG binding protein 2	8.69729667	8.49312333	down	1.15202604	2.66E-01
GSMG0043044	Kdm5d	lysine (K)-specific demethylase 5D	8.54351833	5.18337167	down	10.268451	5.84E-05
GSMG0043052	Uty	ubiquitously transcribed tetratricopeptide repeat gene, Y chromosome	10.14253	9.80892	down	1.26016269	1.01E-01
GSMG0043788	Zbtb21	zinc finger and BTB domain containing 21	9.83877333	9.44138333	down	1.31712293	5.52E-02
GSMG0043807	Taf6l	TAF6-like RNA polymerase II, p300/CBP-associated factor (PCAF)-associated factor	7.90154667	7.75410833	down	1.10760106	2.34E-01
GSMG0050875	Chd3	chromodomain helicase DNA binding protein 3	8.51635333	8.36237333	down	1.1126347	6.02E-01
GSMG0050962	Ash1l	ash1 (absent, small, or homeotic)-like (Drosophila)	9.40786333	9.37901	down	1.02020094	8.15E-01
GSMG0051008	Tet3	tet methylcytosine dioxygenase 3	9.05956333	8.98705833	down	1.05154093	7.35E-01
GSMG0051091	Setd2	SET domain containing 2	9.89412667	9.811235	down	1.0591388	5.27E-01
GSMG0051508	Arid1b	AT rich interactive domain 1B (SWI-like)	10.8773317	10.36494	down	1.42641291	5.02E-02
GSMG0053098	Setd1b	SET domain containing 1B	8.89619667	8.68458	down	1.15798508	2.52E-01
GSMG0000772	Rbbp5	retinoblastoma binding protein 5	8.03327833	8.13545667	up	1.07339296	7.60E-01
GSMG0001320	Ncoa2	nuclear receptor coactivator 2	9.18425333	9.52078	up	1.26271291	2.69E-01

FAST DB STABLE ID	Gene Symbol	Gene Name	TRIPLE (-AB	TRIPLE (+AB	Regulation	Fold-Change	P-Value
GSMG0001683	Kansl1l	KAT8 regulatory NSL complex subunit 1-like	8.43181667	8.67519667	up	1.18376278	5.48E-01
GSMG0003680	Baz2a	bromodomain adjacent to zinc finger domain, 2A	9.93488833	10.1317167	up	1.14617579	1.68E-01
GSMG0003697	Smarcc2	SWI/SNF related, matrix associated, actin dependent regulator of chromatin, subfamily c, member 2	9.87208333	10.1485433	up	1.21121922	2.01E-01
GSMG0004262	Smarcb1	SWI/SNF related, matrix associated, actin dependent regulator of chromatin, subfamily b, member 1	9.53202667	9.83551333	up	1.23412341	1.51E-01
GSMG0004266	Prmt2	protein arginine N-methyltransferase 2	7.73781667	7.91193333	up	1.12827337	5.10E-01
GSMG0004335	Mbd3	methyl-CpG binding domain protein 3	9.16137333	9.23886667	up	1.05518308	7.13E-01
GSMG0004376	Sirt6	sirtuin 6	8.02047	8.37924333	up	1.28233511	1.45E-01
GSMG0005208	Hint1	histidine triad nucleotide binding protein 1	9.42909667	10.13286	up	1.62874792	2.73E-02
GSMG0005616	Suz12	suppressor of zeste 12 homolog (Drosophila)	10.3997	10.6111	up	1.15781119	1.14E-01
GSMG0006179	Cbx2	chromobox 2	9.58096167	9.782465	up	1.14989596	2.45E-01
GSMG0006764	Usp22	ubiquitin specific peptidase 22	8.945015	9.36506667	up	1.33797547	6.18E-02
GSMG0006786	Ncor1	nuclear receptor co-repressor 1	11.1264833	11.3445333	up	1.16316035	1.88E-01
GSMG0006900	Rnasek	ribonuclease, RNase K	9.26364	9.94897333	up	1.60807348	8.36E-03
GSMG0007266	Kat7	K(lysine) acetyltransferase 7	10.4293667	10.5910333	up	1.11857862	3.46E-01
GSMG0007398	Kat2a	K(lysine) acetyltransferase 2A	8.733575	8.94597833	up	1.15861667	4.14E-01
GSMG0007414	Ezh1	enhancer of zeste homolog 1 (Drosophila)	9.94188667	10.0553867	up	1.08184964	4.34E-01
GSMG0007676	Jmjd6	jumonji domain containing 6	8.88046333	9.03576333	up	1.11365317	9.76E-02
GSMG0007706	Cbx8	chromobox 8	7.67510667	8.03573833	up	1.28398795	2.71E-01
GSMG0008522	Yy1	YY1 transcription factor	10.3405	10.4818667	up	1.10294945	3.44E-01
GSMG0008659	Ncoa1	nuclear receptor coactivator 1	9.03991	9.25828667	up	1.16342375	4.25E-02
GSMG0008798	Hdac9	histone deacetylase 9	8.57044	8.67948833	up	1.07851656	6.96E-01
GSMG0009226	Atxn3	ataxin 3	9.008915	9.16795833	up	1.1165465	1.66E-01
GSMG0009294	Setd3	SET domain containing 3	7.48820333	7.82992333	up	1.26726655	1.80E-01
GSMG0009707	Jarid2	jumonji, AT rich interactive domain 2	9.57801333	9.76241333	up	1.13634428	1.38E-01
GSMG0009801	Nsd1	nuclear receptor-binding SET-domain protein 1	10.4823333	10.6512	up	1.12417502	2.49E-01
GSMG0010123	Taf9	TAF9 RNA polymerase II, TATA box binding protein (TBP)-associated factor	10.5501167	10.61115	up	1.0432127	6.40E-01
GSMG0011369	Actr8	ARP8 actin-related protein 8	8.61154333	8.63086333	up	1.01348167	9.21E-01
GSMG0012378	Chd8	chromodomain helicase DNA binding protein 8	9.93940333	9.99761333	up	1.04117314	7.90E-01
GSMG0012395	Prmt5	protein arginine N-methyltransferase 5	9.40233	9.53988667	up	1.10004052	2.89E-01
GSMG0012481	1c // Phf11d // S	PHD finger protein 11C // PHD finger protein 11D // SET domain, bifurcated 2	8.35894667	8.84983667	up	1.40531155	1.46E-01
GSMG0012553	Elp3	elongator acetyltransferase complex subunit 3	9.40223833	9.50431333	up	1.07331608	4.41E-01
GSMG0013415	Ep300	E1A binding protein p300	10.5778	10.8651	up	1.22035425	2.21E-01
GSMG0014452	Hdac7	histone deacetylase 7	10.0046133	10.3054667	up	1.23187283	1.57E-01
GSMG0014572	Cbx5	chromobox 5	9.21354667	9.63326667	up	1.33766791	3.28E-03
GSMG0015331	Chaf1b	chromatin assembly factor 1, subunit B (p60)	8.05698	8.28180833	up	1.16863818	2.63E-01
GSMG0015372	Crebbp	CREB binding protein	10.5615667	10.656	up	1.06764597	4.78E-01
GSMG0015965	Nrip1	nuclear receptor interacting protein 1	8.09492333	8.15875333	up	1.04523693	6.68E-01
GSMG0016797	Kat2b	K(lysine) acetyltransferase 2B	8.70883333	8.88619667	up	1.13081532	3.26E-01
GSMG0016798	Kat2b	K(lysine) acetyltransferase 2B	7.24806333	7.39117667	up	1.10428559	7.09E-01
GSMG0016817	Uhrf1	ubiquitin-like, containing PHD and RING finger domains, 1	9.58139833	9.74329667	up	1.11875826	7.06E-01
GSMG0016981	Mta3	metastasis associated 3	9.89107833	10.1806617	up	1.22228722	6.22E-02
GSMG0017419	Ring1	ring finger protein 1	8.11150667	8.20365	up	1.06595263	7.32E-01
GSMG0018161	Sap130	Sin3A associated protein	9.32379667	9.51241667	up	1.13967305	9.87E-02
GSMG0018578	Mbd2	methyl-CpG binding domain protein 2	11.3357167	11.7346667	up	1.31854792	2.90E-02
GSMG0018593	Mbd1	methyl-CpG binding domain protein 1	8.03657667	8.56636333	up	1.4437157	5.92E-02
GSMG0018778	Ino80c	INO80 complex subunit C	8.48257333	8.51584667	up	1.02333133	8.56E-01
GSMG0018881	Hdac3	histone deacetylase 3	10.1793833	10.30955	up	1.09442013	1.02E-02
GSMG0019336	Men1	multiple endocrine neoplasia 1	8.74903833	8.83034167	up	1.05797338	7.61E-01
GSMG0019661	Hells	helicase, lymphoid specific	8.69022667	9.01739667	up	1.25455003	1.80E-01
GSMG0020453	Mgea5	meningioma expressed antigen 5 (hyaluronidase)	10.6538667	10.9195	up	1.20216368	2.68E-01
GSMG0020837	Wdr5	WD repeat domain 5	9.11200667	9.15592	up	1.03090639	5.50E-01
GSMG0021408	Phf21a	PHD finger protein 21A	9.698795	9.913725	up	1.1606476	3.37E-01
GSMG0021891	srp2bp // Pet117	cysteine and glycine-rich protein 2 binding protein // PET117 homolog (S. cerevisiae)	7.96269667	8.19948	up	1.17836243	4.81E-01
GSMG0022260	Gnas	GNAS (guanine nucleotide binding protein, alpha stimulating) complex locus	11.4728167	11.6603667	up	1.1388281	4.45E-01
GSMG0022430	Trdmt1	tRNA aspartic acid methyltransferase 1	7.43780167	7.661035	up	1.16734689	2.87E-01
GSMG0022550	Nacc2	nucleus accumbens associated 2, BEN and BTB (POZ) domain containing	7.80353667	8.19195167	up	1.30895455	1.04E-01
GSMG0022593	Gtf3c4	general transcription factor IIIC, polypeptide 4	8.27536333	8.30406833	up	1.02009605	1.40E-01
GSMG0022948	Mettl8	methyltransferase like 8	7.89734	7.98582333	up	1.06325183	6.14E-01
GSMG0022984	Atf2	activating transcription factor 2	9.64969667	10.1245533	up	1.38978014	6.40E-02
GSMG0023210	Alkbh3	alkB, alkylation repair homolog 3 (E. coli)	8.98133667	9.19723667	up	1.16142823	3.48E-02
GSMG0023392	Ino80	INO80 homolog (S. cerevisiae)	8.53070667	9.37677	up	1.79758916	1.27E-02
GSMG0023946	Zfp217	zinc finger protein 217	9.03889667	9.27078	up	1.174367	3.58E-01
GSMG0025280	Phc3	polyhomeotic-like 3 (Drosophila)	9.37393667	9.54611667	up	1.1267598	1.63E-01
GSMG0025952	Prmt6	protein arginine N-methyltransferase 6	8.51925667	8.88345	up	1.28716173	1.73E-01
GSMG0026088	Tet2	tet methylcytosine dioxygenase 2	8.91697667	9.11538	up	1.14742777	3.61E-01
GSMG0026666	Kdm4c	lysine (K)-specific demethylase 4C	9.30415	9.66306	up	1.2824566	5.57E-04
GSMG0027163	Taf12	TAF12 RNA polymerase II, TATA box binding protein (TBP)-associated factor	8.41143333	8.87561667	up	1.37953622	2.12E-02
GSMG0028394	Rbbp4	retinoblastoma binding protein 4	11.1173667	11.5002	up	1.30390009	5.05E-02
GSMG0029048	Whsc1	Wolf-Hirschhorn syndrome candidate 1 (human)	9.30778	9.77038333	up	1.37802621	1.60E-01
GSMG0029815	Setd8	SET domain containing (lysine methyltransferase) 8	9.03930333	9.85529667	up	1.76050989	2.74E-02
GSMG0030038	Trrap	transformation/transcription domain-associated protein	9.95040333	9.99562	up	1.03183813	8.01E-01
GSMG0030107	Brca2	breast cancer 2	7.096535	7.37911167	up	1.21636539	1.80E-01
GSMG0030995	Suds3	suppressor of defective silencing 3 homolog (S. cerevisiae)	9.23146333	9.31038667	up	1.05622949	4.23E-01
GSMG0031212	Lrwd1	leucine-rich repeats and WD repeat domain containing 1	8.58537333	8.85501333	up	1.20550698	1.23E-01
GSMG0032001	Smarcad1	SWI/SNF-related, matrix-associated actin-dependent regulator of chromatin, subfamily a, containing DEAD/H box 1	9.05547667	9.07621667	up	1.0144797	8.78E-01
GSMG0032221	Hdac11	histone deacetylase 11	8.23095333	8.33618667	up	1.07566835	5.45E-01
GSMG0032480	Chd4	chromodomain helicase DNA binding protein 4	10.6682333	10.8380667	up	1.12492852	1.53E-01
GSMG0032918	Jhdm1d	jumonji C domain-containing histone demethylase 1 homolog D (S. cerevisiae)	10.3737667	10.5267667	up	1.11187916	1.31E-01
GSMG0032977	Ezh2	enhancer of zeste homolog 2 (Drosophila)	9.44623667	9.946285	up	1.41426094	1.60E-02
GSMG0033238	Ino80b	INO80 complex subunit B	8.25686	8.78385667	up	1.44092642	3.37E-02
GSMG0033642	Apobec1	apolipoprotein B mRNA editing enzyme, catalytic polypeptide 1	9.80024667	10.0562467	up	1.19416319	2.67E-01
GSMG0034257	Sirt2	sirtuin 2	9.54728333	9.67832333	up	1.09508283	4.63E-01
GSMG0035208	Hsd3b7	hydroxy-delta-5-steroid dehydrogenase, 3 beta- and steroid delta-isomerase 7	8.11354667	8.19288333	up	1.05653215	5.92E-01
GSMG0035212	Kat8	K(lysine) acetyltransferase 8	8.08082	8.48025	up	1.31898668	2.10E-01
GSMG0035882	Prmt1	protein arginine N-methyltransferase 1	9.96421667	10.1058583	up	1.10315971	1.67E-01
GSMG0036251	Eed	embryonic ectoderm development	9.76618	9.77772667	up	1.00803565	9.27E-01
GSMG0037122	Whsc1l1	Wolf-Hirschhorn syndrome candidate 1-like 1 (human)	10.3883267	10.6059667	up	1.16282984	5.06E-01
GSMG0037463	Hmgxb4	HMG box domain containing 4	7.96107	8.09927667	up	1.10053625	6.20E-01
GSMG0038443	Sap30	sin3 associated polypeptide	8.30111333	8.70475667	up	1.32284436	3.16E-01
GSMG0039226	Smarca4	SWI/SNF related, matrix associated, actin dependent regulator of chromatin, subfamily a, member 4	9.08771333	9.58601	up	1.41254484	9.07E-02
GSMG0040427	Dnmt1	DNA methyltransferase (cytosine-5) 1	8.97291833	9.066945	up	1.06734507	5.14E-01
GSMG0041616	Kdm6a	lysine (K)-specific demethylase 6A	9.17924167	10.0163783	up	1.78650093	5.23E-04
GSMG0041624	Phf16	PHD finger protein 16	7.79209333	7.811	up	1.01319135	9.05E-01
GSMG0041968	Ogt	O-linked N-acetylglucosamine (GlcNAc) transferase (UDP-N-acetylglucosamine:polypeptide-N-acetylglucosaminyl transferase)	10.5727333	10.6796	up	1.07688685	4.82E-01
GSMG0042197	Phf8	PHD finger protein 8	8.75134	8.82161667	up	1.04991801	3.45E-01
GSMG0042209	Kdm5c	lysine (K)-specific demethylase 5C	10.3518333	10.9802833	up	1.54590322	9.70E-03
GSMG0042259	Rbbp7	retinoblastoma binding protein 7	11.1192333	11.4704	up	1.27559175	7.42E-02
GSMG0042325	Hdac6	histone deacetylase 6	8.120185	8.36741167	up	1.18692326	7.68E-02
GSMG0042329	Suv39h1	suppressor of variegation 3-9 homolog 1 (Drosophila)	9.29036333	9.57506667	up	1.21815974	2.33E-01
GSMG0042604	Hcfc1	host cell factor C1	9.62608667	9.80020833	up	1.12827728	2.33E-01
GSMG0042732	Hdac8	histone deacetylase 8	8.485345	8.53179833	up	1.03272299	6.89E-01
GSMG0043470	Bptf	bromodomain PHD finger transcription factor	9.61591333	9.67252	up	1.04001668	7.95E-01
GSMG0050863	Cbx4	chromobox 4	8.99782667	9.0275	up	1.02078097	9.16E-01
GSMG0050913	Kmt2d	lysine (K)-specific methyltransferase 2D	9.72160333	9.76370667	up	1.02961383	8.11E-01
GSMG0051006	Cecr2	cat eye syndrome chromosome region, candidate 2	8.16903333	8.22176	up	1.03722341	8.82E-01

Supplementary Table S5B. SPECIFIC EPIGENETIC GENES REGULATED IN HR-MDS upon treatment with ABT-737

FAST DB STABLE ID	Gene Symbo	Gene Name	Mean TRIPLE (-ABT-737)	Mean TRIPLE (+ABT-737)	Regulation	Fold-Change	P-Value
GSMG0000801	Kdm5b	lysine (K)-specific demethylase 5B	8.73937	8.531166667	down	1.15524859	6.12E-02
GSMG0001898	Hdac4	histone deacetylase 4	8.213191667	8.183548333	down	1.02075974	8.51E-01
GSMG0002875	Hdac2	histone deacetylase 2	9.619203333	9.4726	down	1.10696019	4.43E-01
GSMG0003275	Dot1l	DOT1-like, histone H3 methyltransferase (S. cerevisiae)	8.84743	8.59192	down	1.19375767	3.01E-01
GSMG0004171	Tet1	tet methylcytosine dioxygenase 1	6.866338333	6.742221667	down	1.08984024	3.73E-01
GSMG0004178	Sirt1	sirtuin 1	10.53904	10.40808333	down	1.09501958	4.97E-01
GSMG0007440	Hdac5	histone deacetylase 5	8.34755	7.910148333	down	1.35416324	2.79E-02
GSMG0007782	Dnmt3a	DNA methyltransferase 3A	9.029633333	8.698543333	down	1.25796345	8.16E-02
GSMG0009726	Kdm1b	lysine (K)-specific demethylase 1B	9.779303333	9.492346667	down	1.22006386	1.44E-01
GSMG0011295	Kat6b	K(lysine) acetyltransferase 6B	9.635285	9.401755	down	1.17570817	2.33E-01
GSMG0014365	Hdac10	histone deacetylase 10	8.880031667	8.599968333	down	1.21424819	7.58E-02
GSMG0016565	Ehmt2	euchromatic histone lysine N-methyltransferase 2	10.17527833	10.08228	down	1.06658455	4.29E-01
GSMG0019254	Suv420h1	suppressor of variegation 4-20 homolog 1 (Drosophila)	10.66711667	10.00985333	down	1.57708819	4.88E-03
GSMG0019936	Kdm2a	lysine (K)-specific demethylase 2A	10.17593333	10.16837	down	1.00525627	9.66E-01
GSMG0019983	Kat5	K(lysine) acetyltransferase 5	9.705333333	9.304573333	down	1.3202032	5.09E-02
GSMG0021999	Dnmt3b	DNA methyltransferase 3B	7.660425	7.529671667	down	1.09486526	3.22E-01
GSMG0022340	Suv39h2	suppressor of variegation 3-9 homolog 2 (Drosophila)	7.735335	7.71569	down	1.01371001	9.41E-01
GSMG0022508	Ehmt1	euchromatic histone methyltransferase 1	9.66375	9.048676667	down	1.53163585	1.62E-02
GSMG0028400	Hdac1	histone deacetylase 1	10.373	10.2639	down	1.07855519	4.27E-01
GSMG0028519	Kdm1a	lysine (K)-specific demethylase 1A	9.522933333	9.321431667	down	1.14989463	3.15E-01
GSMG0028904	Kmt2e	lysine (K)-specific methyltransferase 2E	10.15866667	10.03976667	down	1.08590658	2.79E-01
GSMG0030275	Kmt2c	lysine (K)-specific methyltransferase 2C	10.15601667	9.836995	down	1.24748431	7.58E-02
GSMG0031058	Kdm2b	lysine (K)-specific demethylase 2B	9.10787	8.547815	down	1.47432542	2.95E-02
GSMG0033558	Mbd4	methyl-CpG binding domain protein 4	8.56178	8.209923333	down	1.27620197	3.26E-02
GSMG0033956	Suv420h2	suppressor of variegation 4-20 homolog 2 (Drosophila)	9.28008	9.1862	down	1.06723656	5.15E-01
GSMG0035151	Kdm8	lysine (K)-specific demethylase 8	7.626273333	7.437506667	down	1.13978891	3.81E-01
GSMG0035751	Kmt2b	lysine (K)-specific methyltransferase 2B	8.619116667	8.157978333	down	1.37662759	3.37E-02
GSMG0036847	Sirt3	sirtuin 3	8.453876667	8.195526667	down	1.19610994	2.94E-02
GSMG0037097	Kat6a	K(lysine) acetyltransferase 6A	10.68125	10.32428333	down	1.28073027	1.25E-02
GSMG0037724	Ctcf	CCCTC-binding factor	11.72441667	11.60926667	down	1.08308765	1.15E-01
GSMG0040672	Kmt2a	lysine (K)-specific methyltransferase 2A	10.67653333	10.60446667	down	1.05122148	8.16E-01
GSMG0042609	Mecp2	methyl CpG binding protein 2	8.697296667	8.493123333	down	1.15202604	2.66E-01
GSMG0043044	Kdm5d	lysine (K)-specific demethylase 5D	8.543518333	5.183371667	down	10.268451	5.84E-05
GSMG0051008	Tet3	tet methylcytosine dioxygenase 3	9.059563333	8.987058333	down	1.05154093	7.35E-01
GSMG0001320	Ncoa2	nuclear receptor coactivator 2	9.184253333	9.52078	up	1.26271291	2.69E-01
GSMG0004335	Mbd3	methyl-CpG binding domain protein 3	9.161373333	9.238866667	up	1.05518308	7.13E-01
GSMG0004376	Sirt6	sirtuin 6	8.02047	8.379243333	up	1.28233511	1.45E-01
GSMG0006786	Ncor1	nuclear receptor co-repressor 1	11.12648333	11.34453333	up	1.16316035	1.88E-01
GSMG0007266	Kat7	K(lysine) acetyltransferase 7	10.42936667	10.59103333	up	1.11857862	3.46E-01
GSMG0007398	Kat2a	K(lysine) acetyltransferase 2A	8.733575	8.945978333	up	1.15861667	4.14E-01
GSMG0007414	Ezh1	enhancer of zeste homolog 1 (Drosophila)	9.941886667	10.05538667	up	1.08184964	4.34E-01
GSMG0007676	Jmjd6	jumonji domain containing 6	8.880463333	9.035763333	up	1.11365317	9.76E-02
GSMG0007706	Cbx8	chromobox 8	7.675106667	8.035738333	up	1.28398795	2.71E-01
GSMG0008659	Ncoa1	nuclear receptor coactivator 1	9.03991	9.258286667	up	1.16342375	4.25E-02
GSMG0008798	Hdac9	histone deacetylase 9	8.57044	8.679488333	up	1.07851656	6.96E-01
GSMG0009707	Jarid2	jumonji, AT rich interactive domain 2	9.578013333	9.762413333	up	1.13634428	1.38E-01
GSMG0012395	Prmt5	protein arginine N-methyltransferase 5	9.40233	9.539886667	up	1.10004052	2.89E-01
GSMG0013415	Ep300	E1A binding protein p300	10.5778	10.8651	up	1.22035425	2.21E-01
GSMG0014452	Hdac7	histone deacetylase 7	10.00461333	10.30546667	up	1.23187283	1.57E-01
GSMG0014572	Cbx5	chromobox 5	9.213546667	9.633266667	up	1.33766791	3.28E-03
GSMG0016797	Kat2b	K(lysine) acetyltransferase 2B	8.708833333	8.886196667	up	1.13081532	3.26E-01
GSMG0016798	Kat2b	K(lysine) acetyltransferase 2B	7.248063333	7.391176667	up	1.10428559	7.09E-01
GSMG0018578	Mbd2	methyl-CpG binding domain protein 2	11.33571667	11.73466667	up	1.31854792	2.90E-02
GSMG0018593	Mbd1	methyl-CpG binding domain protein 1	8.036576667	8.566363333	up	1.4437157	5.92E-02
GSMG0018881	Hdac3	histone deacetylase 3	10.17938333	10.30955	up	1.09442013	1.02E-02
GSMG0025952	Prmt6	protein arginine N-methyltransferase 6	8.519256667	8.88345	up	1.28716173	1.73E-01
GSMG0026088	Tet2	tet methylcytosine dioxygenase 2	8.916976667	9.11538	up	1.14742777	3.61E-01
GSMG0026666	Kdm4c	lysine (K)-specific demethylase 4C	9.30415	9.66306	up	1.2824566	5.57E-04
GSMG0032918	Jhdm1d	onji C domain-containing histone demethylase 1 homolog D (S. cerevisiae)	10.37376667	10.52676667	up	1.11187916	1.31E-01
GSMG0032977	Ezh2	enhancer of zeste homolog 2 (Drosophila)	9.446236667	9.946285	up	1.41426094	1.60E-02
GSMG0034257	Sirt2	sirtuin 2	9.547283333	9.678323333	up	1.09508283	4.63E-01
GSMG0040427	Dnmt1	DNA methyltransferase (cytosine-5) 1	8.972918333	9.066945	up	1.06734507	5.14E-01
GSMG0041616	Kdm6a	lysine (K)-specific demethylase 6A	9.179241667	10.01637833	up	1.78650093	5.23E-04
GSMG0042209	Kdm5c	lysine (K)-specific demethylase 5C	10.35183333	10.98028333	up	1.54590322	9.70E-03
GSMG0042325	Hdac6	histone deacetylase 6	8.120185	8.367411667	up	1.18692326	7.68E-02
GSMG0042329	Suv39h1	suppressor of variegation 3-9 homolog 1 (Drosophila)	9.290363333	9.575066667	up	1.21815974	2.33E-01
GSMG0042732	Hdac8	histone deacetylase 8	8.485345	8.531798333	up	1.03272299	6.89E-01
GSMG0050863	Cbx4	chromobox 4	8.997826667	9.0275	up	1.02078097	9.16E-01
GSMG0050913	Kmt2d	lysine (K)-specific methyltransferase 2D	9.721603333	9.763706667	up	1.02961383	8.11E-01
